# Comparative effectiveness of acupuncture-based multimodal rehabilitation for post-stroke cognitive impairment: a network meta-analysis of 70 randomized controlled trials

**DOI:** 10.3389/fneur.2026.1759572

**Published:** 2026-02-10

**Authors:** Tingting Yin, Peifang Li

**Affiliations:** 1 The Second Clinical Medical College, Anhui University of Chinese Medicine, Hefei City, Anhui Province, China; 2 Department of Encephalopathy, The Second Affiliated Hospital of Anhui University of Chinese Medicine, Hefei City, Anhui Province, China

**Keywords:** acupuncture, cognitive impairment, efficacy, network meta-analysis, stroke

## Abstract

**Introduction:**

This study systematically identified randomized controlled trials evaluating therapeutic strategies for post-stroke cognitive impairment (PSCI) and performed a comprehensive systematic review and network meta-analysis to compare the effectiveness of acupuncture-based multimodal rehabilitation interventions.

**Methods:**

PubMed, Embase, the Cochrane Library, Web of Science, CNKI, and Wanfang were searched from inception. A systematic review and network meta-analysis were performed using Montreal Cognitive Assessment (MoCA), Mini-Mental State Examination (MMSE), Barthel Index (BI) and Total Effective Rate (TER) as outcomes. Risk of bias was assessed using RoB 2, and certainty of evidence was graded with the CINeMA framework.

**Results:**

A total of 70 randomized controlled trials involving 6,259 participants and 25 acupuncture-based rehabilitation regimens were included. For MoCA, combined strategies such as ScA-N-SOC (SMD = 1.89, 95% CI: 1.59–2.19) and BA-N-SOC (SMD = 1.60, 95% CI: 1.26–1.93) showed notable gains. For MMSE, BA-C (SMD = 2.41, 95% CI: 1.64–3.19) and ScA-N-SOC (SMD = 1.97, 95% CI: 1.67–2.26) produced significantly greater improvements versus SOC. In BI, ScA-N-SOC (SMD = 1.50, 95% CI: 0.77–2.22) and BA-N-SOC (SMD = 1.30, 95% CI: 0.78–1.81). For TER, E-C-SOC (RR = 2.18, 95% CI: 1.24–3.83) and EA-C-SOC (RR = 1.73, 95% CI: 1.19–2.50) yielded higher response rates.

**Conclusion:**

Significant differences were observed in the comparative effectiveness of acupuncture-based multimodal interventions for post-stroke cognitive impairment (PSCI). Overall, composite strategies incorporating noninvasive brain stimulation or cognitive training produced greater improvements in cognitive function (MoCA, MMSE) as well as activities of daily living assessed by the Barthel Index (BI). Scalp acupuncture or electroacupuncture combined with noninvasive brain stimulation demonstrated the most consistent benefits for MMSE and BI outcomes, while multimodal combined interventions also showed favorable effects on MoCA performance. These findings suggest that multi-pathway, multi-target rehabilitation strategies may outperform single-modality acupuncture or conventional rehabilitation, providing evidence-based support for individualized treatment of PSCI. Further high-quality, multicenter randomized controlled trials with long-term follow-up are warranted to confirm these findings and to elucidate the underlying neurobiological mechanisms.

**Systematic review registration:**

This systematic review and network meta-analysis was registered in PROSPERO (CRD420251229557). https://www.crd.york.ac.uk/prospero/.

## 1 Introduction

Stroke is an acute and highly disabling cerebrovascular disease, which continues to pose a major threat to the health of middle-aged and elderly people (1). China’s large-scale epidemiological survey shows that the burden of stroke is heavy, with a weighted prevalence of 844.5 cases per 100,000 people, and the prevalence rate increases significantly with age. The prevalence of all age groups has increased significantly. The prevalence of people aged 50–59 is 1854.5 cases per 100,000 people, the prevalence rate of people aged 60 to 69 is 4259.1 cases per 100,000 people, and the prevalence rate of people aged 70 to 79 is as high as 6670.5 cases per 100,000 people, which reflects the decline The increase of old-related risks is accelerating. According to recent national monitoring reports, the age-standardized incidence rate is 276.2 cases per 100,000 people per year, and the age-standardized mortality rate is 149.5 cases per 100,000 people per year, highlighting the recognized pattern of high incidence, frequent recurrence and high mortality (1).

Stroke can be roughly divided into two subtypes: ischemic and hemorrhagic, and both subtypes show an upward trend in China (2). Epidemiological evidence shows that China bears the heaviest burden of stroke in the world, accounting for about 40% of the world’s new cases (2). With the rapid aging of the population and the increasing prevalence of vascular risk factors such as hypertension, diabetes and dyslipidemia, the number of stroke survivors in China has exceeded 11 million. Relapse is still a persistent challenge, with about 17.7% of survivors having a recurrence within a year, and the recurrence rate has risen to 30–40% within 5 years (3). In addition to the significant impact on long-term disability, stroke can also significantly increase health care spending. According to national data, the annual direct medical expenses of each stroke patient are estimated at 20,000–30,000 yuan, resulting in an economic burden of more than 300 billion yuan, and the trend continues to rise (3). Stroke can lead to a variety of neurological sequelae, such as cognitive disorders, motor dysfunction, emotional and mental disorders, difficulty swallowing and aphasia. Among them, post-stroke cognitive impairment (PSCI) is one of the most common complications with the most serious clinical consequences (4). There is evidence that about 38% of stroke survivors develop cognitive disorders within a year, and 7 to 41% of patients subsequently develop dementia. PSCI significantly impairs the quality of life and social functions of patients, and is closely related to the increase in long-term mortality (4).

At present, clinical treatment is still dominated by drug intervention. Drugs such as donepizil are widely used to improve neurological function and alleviate the symptoms of cognitive disorders (5). However, simple drug treatment is difficult to reverse the nerve damage that has occurred, and long-term drug use may cause a variety of adverse reactions, bringing potential adverse effects on organ function. Therefore, exploring therapeutic strategies that are safe, effective and conducive to promoting neuroplastic reconstruction has become an important research direction in the field of PSCI (6). Conventional rehabilitation (such as physical therapy, occupational therapy, etc.) is a basic measure for comprehensive management after stroke, and its safety has been widely recognized (7), but existing evidence shows that the effect of simply relying on conventional rehabilitation on improving PSCI is relatively limited, especially in attention, executive function and advanced cognitive function. Insufficient improvement in the field (8). According to traditional Chinese medicine (TCM), PSCI belongs to the category of “brain disorders,” and its clinical manifestations correspond to the TCM classifications of “stroke” accompanied by symptoms such as “forgetfulness” and “mental dullness.” The pathogenesis is characterized by root deficiency with secondary excess, in which wind, fire, phlegm, blood stasis, and deficiency serve as the primary pathogenic factors. These factors lead to obstruction of the cerebral vessels and insufficient nourishment of the sea of marrow, eventually resulting in pathological changes described in TCM as “marrow depletion, brain diminution, and impairment of mental function (9).”As one of the characteristic therapies of traditional Chinese medicine, acupuncture is increasingly used in the prevention and treatment of PSCI. Previous studies have shown that acupuncture and moxibustion treatment of PSCI has good clinical efficacy, easy operation, high safety, few adverse reactions, and good patient compliance (10–13). It may improve post-stroke motor, speech and cognitive dysfunction by regulating qi and blood circulation, improving cerebral blood flow and microcirculation, inhibiting inflammatory reactions, reducing oxidative stress and promoting nerve repair and plastic reconstruction (14).

However, there are still significant heterogeneities in the current acupuncture and moxibustion research on PSCI, including significant differences in acupoint selection principles, stimulation methods (for example, body acupuncture, electric acupuncture, scalp acupuncture, warm acupuncture), treatment frequency and course. Due to the lack of high-quality direct comparative trials, the best acupuncture treatment plan has not been determined. Network meta-analysis (NMA) can integrate direct and indirect evidence, compare and sort multiple interventions, even in limited direct comparison, thus providing stronger evidence for clinical decision-making (15). Therefore, this study adopts the NMA framework, comprehensively analyzes the evidence of randomized controlled trials, and evaluates the relative effectiveness of the multi-mode rehabilitation strategy based on acupuncture in the treatment of PSCI, aiming to provide a basis for a more individualized and optimized treatment plan.

## 2 Materials and methods

This network meta-analysis followed the methodological standards outlined in the Preferred Reporting Items for Systematic Reviews and Meta-Analyses extension for network meta-analyses (PRISMA-NMA) (Supplementary Table 1) (16). In view of the current lack of randomized controlled trials that can directly compare acupuncture and moxibustion therapy, this study adopts an indirect comparison method to sequence the multi-mode and acupuncture-centered rehabilitation strategies studied based on probability (17). To uphold methodological transparency, ensure reproducibility, and maintain analytical rigor, the study protocol was prospectively registered in the International Prospective Register of Systematic Reviews (PROSPERO; CRD420251229557).

### 2.1 Data sources and search strategy

A comprehensive literature search was undertaken across PubMed, EMBASE, the Cochrane Library, Web of Science, CNKI, and Wanfang. The search strategy integrated both free-text terms and controlled vocabulary, centered on key concepts including “Stroke,” “Cognitive Dysfunction,” “Cognitive Impairment,” “Cerebrovascular Accident,” “Acupuncture Therapy,” “Electroacupuncture,” and “Randomized Controlled Trial” (Supplementary Table 2). All databases were searched from their inception to October 1, 2025. No language restrictions were imposed to minimize selection bias and ensure full coverage of relevant evidence.

### 2.2 Selection criteria

Inclusion criteria: (1) Randomized controlled trials enrolling patients diagnosed with PSCI, characterized by cognitive deficits such as impairments in memory, attention, and executive function following documented brain tissue injury;(2) The experimental group adopted the study of multi-mode rehabilitation strategies based on acupuncture, including but not limited to manual acupuncture, electric acupuncture, scalp acupuncture or warm acupuncture;(3) Compare these interventions with trials of standard nursing (SOC), routine rehabilitation training, drug treatment or alternative acupuncture methods to ensure sufficient network connectivity to support effective indirect and mixed treatment comparisons;(4) Eligible randomized controlled trials must report at least one of the following results: Montreal Cognitive Assessment (MoCA), which is conducted using the Montreal Cognitive Assessment Scale, which is often used to detect mild cognitive impairment, and higher scores reflect greater cognitive improvements (18); simple Easy Mental State Examination (MMSE) is measured by simple mental state examination, which is an overall cognitive assessment tool, in which a higher score indicates better cognitive function (19); Basel Index (BI), which is evaluated by the Basel Index, which quantifies daily life activities and functions. Independence, of which a higher score indicates better recovery (20); Total effective rate (TER), defined as the proportion of participants classified as clinically improved according to prespecified criteria in each trial.

Exclusion criteria included: (1) A randomized controlled trial with repeated evaluation of the same group of participants at different points in time, resulting in repeated observation results; (2) Research that did not clearly report the main outcome or did not provide a sufficiently detailed outcome evaluation method; (3) Non-original research, including review articles, case reports, meeting minutes or other forms of secondary analysis; (4) Before being finally included in the study, all retrieved records were preliminarily screened according to titles and abstracts. After that, the two researchers independently reviewed the full text to ensure that only the latest randomized controlled trials that meet all the predetermined inclusion criteria are included.

### 2.3 Data extraction and quality assessment

Two researchers independently extracted data from the randomized controlled trials in accordance with the Preferred Reporting Items for Systematic Reviews and Meta-Analysis process, and any discrepancies were resolved through discussion. For each eligible study, we collected information on the first author and year of publication, sample size, age and sex distribution, geographical setting, duration of follow-up, and detailed descriptions of interventions in both the experimental and comparator arms. For continuous outcomes (MoCA, MMSE, and BI), mean changes and their corresponding standard deviations (SDs) were preferentially extracted. When only baseline and post-intervention scores were available, mean changes and SDs were calculated using established transformation formulas. In cases where the correlation coefficient (r) required for SD conversion was not reported and could not be estimated from the available data, a conservative r value of 0.5 was applied. Sensitivity analyses were subsequently undertaken to examine the robustness of effect estimates under different assumptions.

MEANchange=Endpoint Mean−Baseline Mean

$$\text{SDchange}=\sqrt{\begin{array}{l} \text{Baseline}\;{\mathrm{SD}}^{2}+\text{Endpoint}\;{\mathrm{SD}}^{2}-\\ 2R\cdot \text{Baseline}\;\mathrm{SD}\cdot \text{Endpoint}\;\mathrm{SD} \end{array}}$$

For the results of the second classification, the number of events and the total number of participants of each research group were extracted. All the methodological qualities included in the trial are evaluated using the bias risk assessment tool 2 (RoB 2), which can assess potential bias from five core areas. The evaluation content includes: the adequacy of the randomization process, including whether the sequence generation is correct and whether the distribution concealment is in place; the degree to which the intervention deviation from expectations, paying special attention to the compliance and blind implementation of participants and researchers; the impact of missing outcome data, including the degree and reasons for missed visits and its research results The potential impact of the effectiveness of the result; the reliability of the outcome measurement, ensuring that the evaluation is objective, consistent and under blind conditions; and the possibility of selective reporting, including not reporting predetermined outcomes or prioritizing statistically significant results. According to the RoB 2 standard, each area is judged to have low-risk, some problems or high-risk bias (21).

### 2.4 Statistical analysis

Use Stata/MP 17.0 for network meta-analysis. For the continuous outcome, when all studies use the same measurement scale, the mean difference (MD) and its 95% confidence interval (CI) are calculated; otherwise, the standardized mean difference (SMD) and its 95% CI are used. For dichotomous outcomes, the risk ratio (RR) with its 95% confidence interval (CI) was calculated. When incorporating the multi-arm test, the treatment group was not split; instead, all groups were jointly modeled at the research level to retain their inherent relevant structures and avoid artificial reduction of standard errors. For dichotomous outcomes with zero-event or zero-non-event cells, a continuity correction (r = r + 0.5; n = n + 1) was uniformly applied to all treatment groups to prevent infinite RR estimates. The main analysis hypothesis is consistent within the framework of random effects, and uses the restrictive maximum likelihood method (REML) to estimate the inter-study variance (τ^2^). In a network containing a closed loop, first evaluate the global inconsistency, and then use the node splitting method to evaluate the local inconsistency. For each closed loop, the inconsistency factor (IF) is calculated on the logarithm effect scale. If with zero in the 95% confidence interval is interpreted as no significant difference in the statistical sense between direct evidence and indirect evidence. The ranking of intervention strategies was estimated using SUCRA values, and cumulative ranking probability curves were generated accordingly. Publication bias and small-study effects were assessed using comparison-adjusted funnel plots when more than 10 studies were available. Robustness was examined through leave-one-out sensitivity analysis, in which each study was sequentially omitted under the random-effects consistency model to compare changes in the direction and magnitude of pooled effects. Furthermore, a univariate network meta-regression was conducted to evaluate the influence of study-level covariates on treatment effects, with regression coefficients, 95% confidence intervals, and Wald test *p*-values reported. A *p* < 0.05 was considered to indicate statistically significant effect modification by the covariate.

### 2.5 GRADE assessment

The certainty of evidence for the network meta-analysis estimates was evaluated using the GRADE framework, supplemented by the Confidence in Network Meta-analysis (CINeMA) methodology. Because all included studies were randomized controlled trials, each comparison began with a rating of high certainty and was subsequently downgraded when concerns were identified across six domains: within-study bias, indirectness, imprecision, heterogeneity, inconsistency, and across-study bias (i.e., publication bias or small-study effects). Within study bias was assessed using the RoB 2 tool on a domain specific basis. The CINeMA contribution matrix was applied to weight these risk of bias judgments according to each study’s relative contribution to the network estimates, thereby generating comparison level evaluations. Indirectness was examined in relation to assumptions of transitivity and exchangeability, drawing on prespecified effect modifiers such as baseline severity, intervention intensity, and follow up duration to judge the comparability of direct and indirect evidence with respect to participants, interventions, comparators, and outcome measurements. Imprecision was assessed using minimally important difference (MID) thresholds. For dichotomous outcomes, an RR of 1.25 served as the threshold for a clinically meaningful effect, whereas the MID for continuous outcomes was set at an SMD of 0.5. The 95% confidence interval was evaluated to determine whether it crossed both the line of no effect and the MID threshold. Heterogeneity was judged by considering the magnitude of the between-study variance (τ^2^) obtained from random-effects models and the location of the prediction interval relative to the MID. In networks containing closed loops, inconsistency was examined using CINeMA’s internal tools that compare direct and indirect evidence, incorporating node-splitting/SIDE procedures and design-by-treatment interaction models. Across-study bias was assessed by reviewing trial registration status, gray literature searches, and evidence of small-study effects derived from comparison-adjusted funnel plots. Each domain was categorized as having no concerns, some concerns, or major concerns. Following GRADE recommendations, evidence was downgraded by one level when some concerns were present and by two levels when major concerns were identified. Final certainty ratings were classified as high, moderate, low, or very low.

## 3 Results

### 3.1 Systematic review and characteristics of the included studies

**Figure 1:**
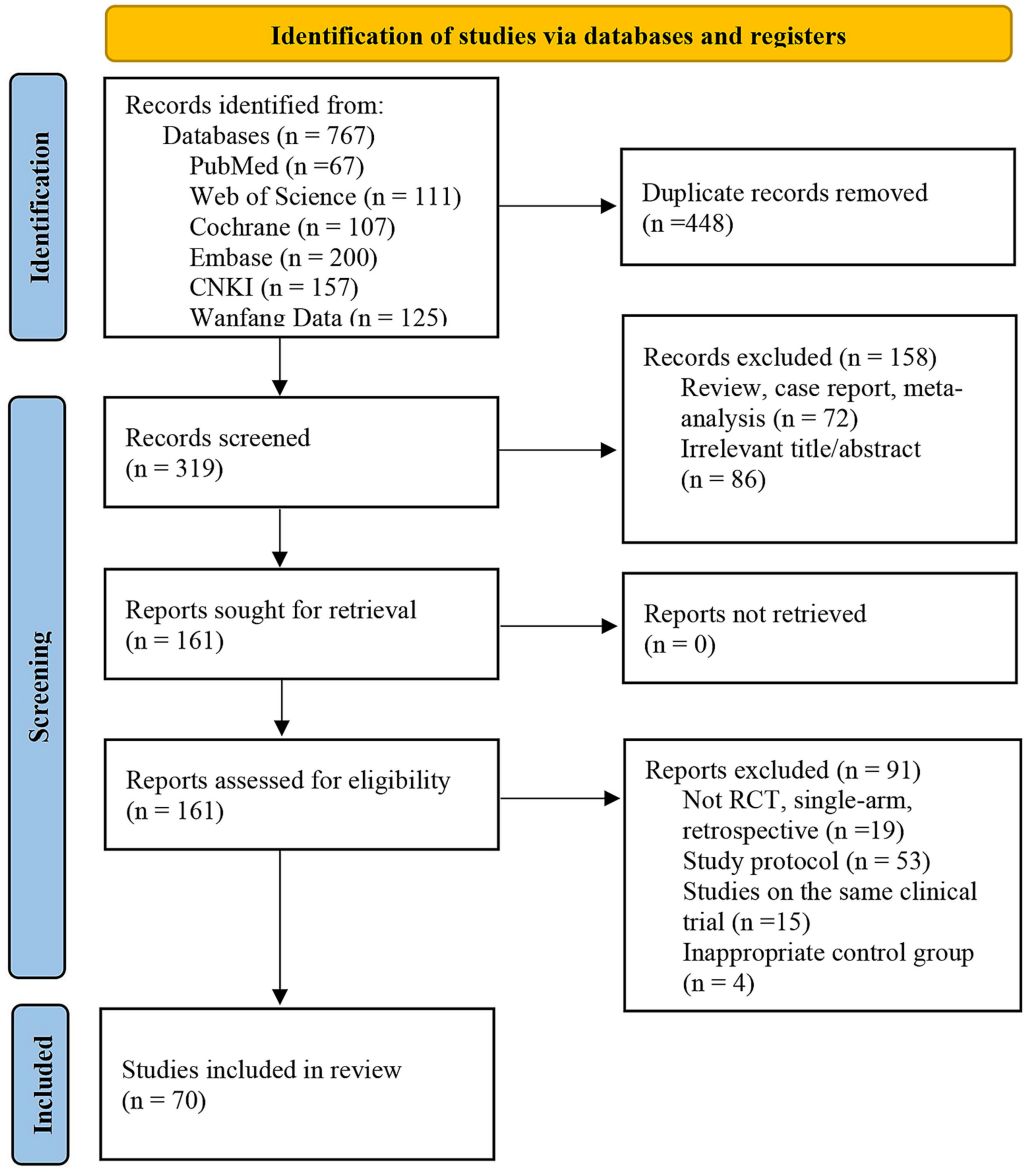
Flow diagram of the literature search and study selection process conducted in accordance with PRISMA guidelines.

The initial search identified 767 records. After removing duplicates and screening titles and abstracts, 161 articles were retrieved for full-text review. Ultimately, 70 randomized controlled trials met the eligibility criteria (Figure 1). These studies collectively enrolled 6,259 participants and evaluated 25 distinct therapeutic strategies, including Body acupuncture-standard care (BA-SOC); Body acupuncture-cognitive training-standard care (BA-C-SOC); Body acupuncture-non-invasive brain stimulation-standard care (BA-N-SOC); Body acupuncture-moxibustion-standard care (BA-M-SOC); Body acupuncture-cognitive training (BA-C); Body acupuncture alone (BA); Body acupuncture plus bleeding therapy (B-SOC); Electroacupuncture-standard care (EA-SOC); Electroacupuncture-NIBS-standard care (EA-N-SOC); Electroacupuncture-cognitive training-standard care (EA-C-SOC); Electroacupuncture-moxibustion-cognitive training-standard care (EA-M-C-SOC); Ear acupuncture-standard care (E-SOC); Scalp acupuncture-standard care (ScA-SOC); Scalp acupuncture-cognitive training-standard care (ScA-C-SOC); Scalp acupuncture-NIBS-standard care (ScA-N-SOC); Scalp acupuncture-body acupuncture-standard care (ScA-BA-SOC); Scalp acupuncture-bleeding therapy-standard care (ScA-B-SOC); Scalp acupuncture with simultaneous cognitive training-standard care (ScAsim-C-SOC); Scalp acupuncture-ear acupuncture-cognitive training-standard care (ScA-E-C-SOC); Eye acupuncture-NIBS-standard care (EyeAcu-N-SOC); standard care alone (SOC); Cognitive training-standard care (C-SOC); NIBS-standard care (N-SOC); Moxibustion-cognitive training-standard care (M-C-SOC); EarAcu-Cognitive training-Standard of care (E-C-SOC) interventions. Study-level characteristics for all included trials are summarized in Supplementary Table 3.

Quality assessments using the RoB 2.0 tool show that the overall risk of bias in 70 trials is low. Fifty-five studies were rated as “low risk,” 15 were rated as “some problems,” and only 0 were rated as “high risk.” Most studies provide clearly described randomization methods, standardized interventions, complete result reports and objective evaluation procedures, reflecting the overall good methodological rigor. Regarding the randomization process, most experiments use reliable methods, such as random number tables or computer-generated sequences, and report the balance of baseline characteristics between groups. A few studies mentioned random distribution, but did not specify the allocation procedure; some early published literature seemed to allocate subjects according to the order of admission or treatment time, which may have introduced selective bias.

In cases of deviation from the expected interventions, the follow-up of most trials is satisfactory. Only a few trials do not clearly indicate whether the treatment operator adopts the blind method; however, in view of the nature of acupuncture-related interventions, its potential impact on the main outcome is considered negligible.

It is not common for the end data to be missing. Most of the tests have a high completion rate, a low dropout rate, and the reasons for dropout are recorded. Although a few studies lack detailed follow-up information, no signs of selective exclusion or selective reporting have been found, indicating good overall data integrity.

The outcome measurements were generally reliable. Validated instruments such as MoCA, MMSE, BI and TER were consistently used as primary outcome measures, ensuring standardized and objective evaluations across studies.

Taken together, the overall risk of bias across domains was low, and the methodological quality of the included studies was considered acceptable, lending confidence to the pooled estimates. Detailed domain-level assessments are presented in Supplementary Figure 1.

### 3.2 Network meta-analyses

**Figure 2:**
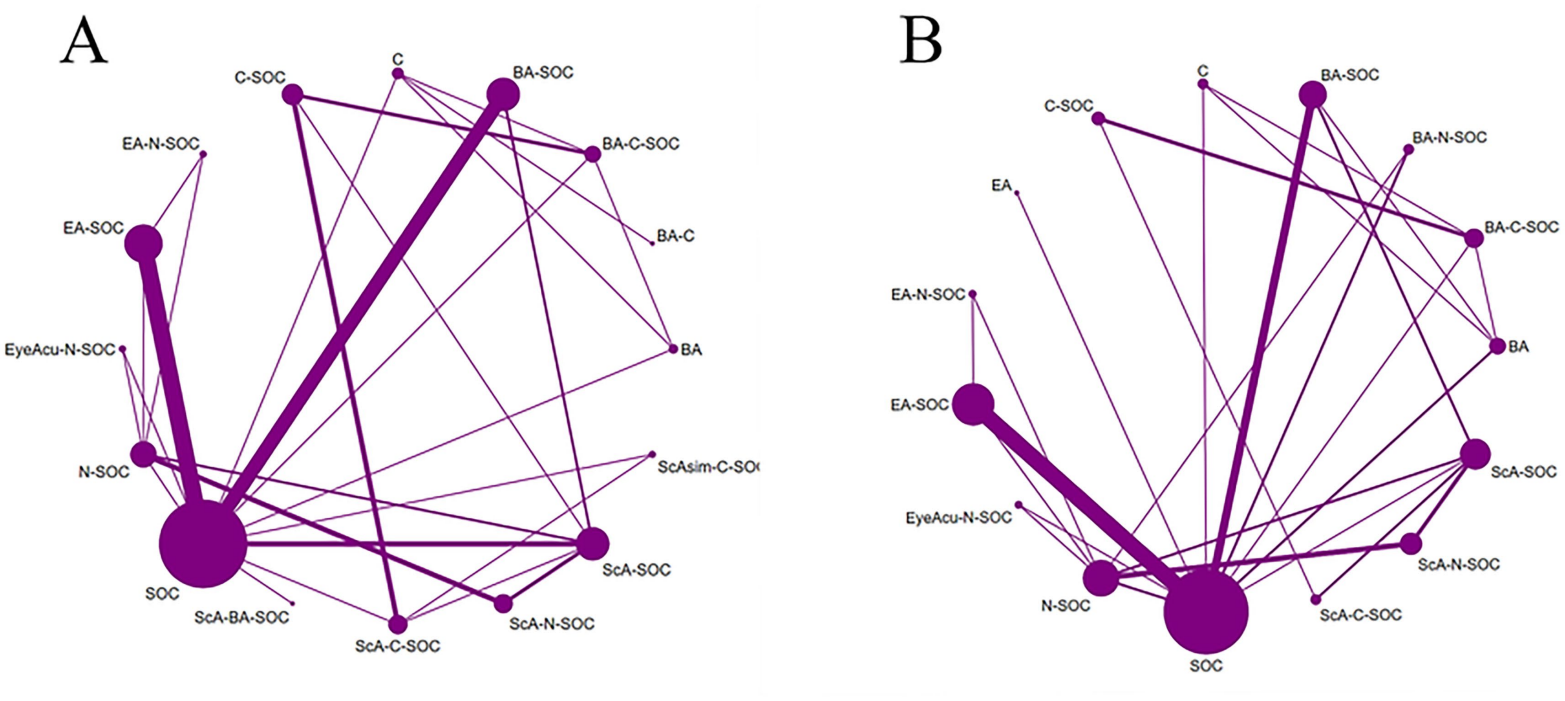
Network plots of acupuncture-based multimodal rehabilitation for PSCI. **(A)** MMSE outcomes. **(B)** MoCA outcomes.

**Figure 3:**
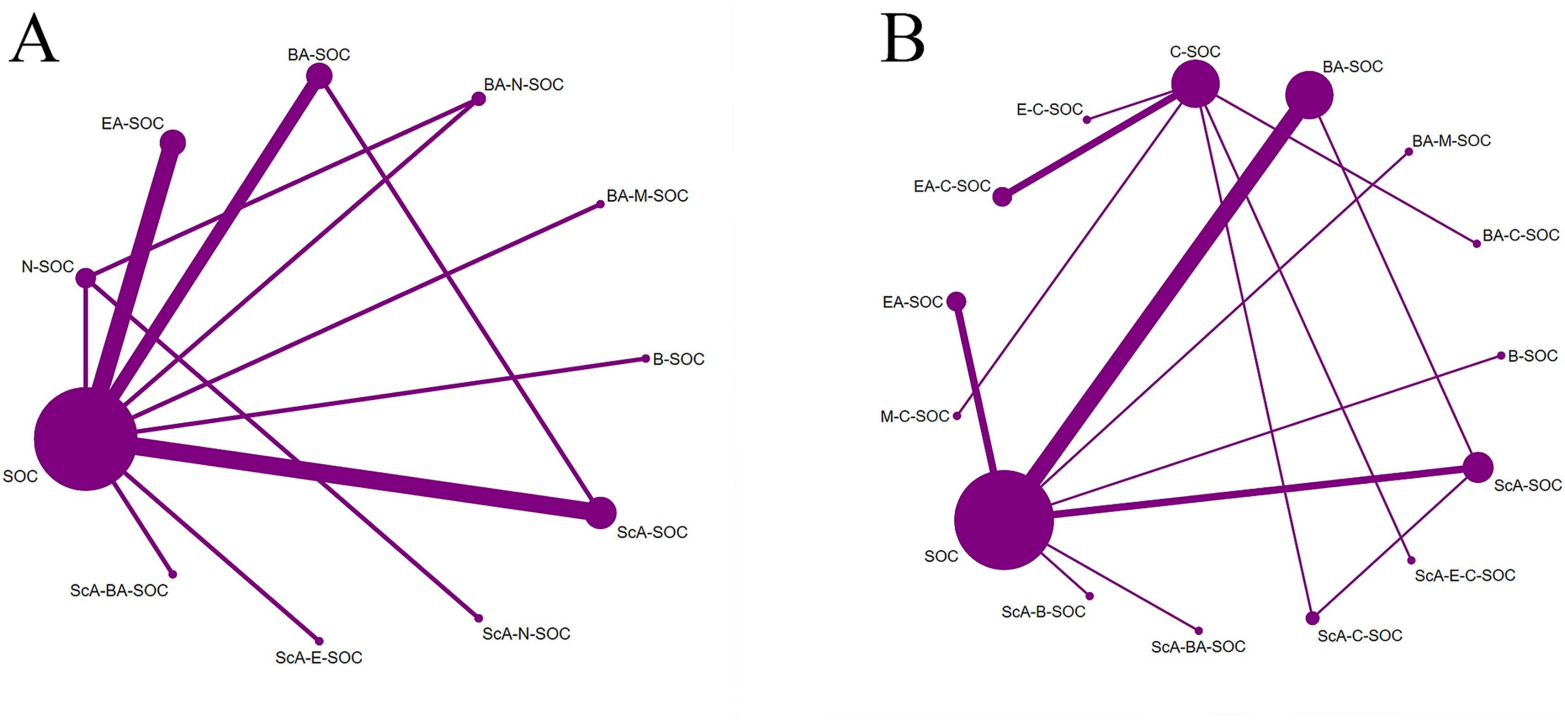
Network plots of acupuncture-based multimodal rehabilitation for PSCI. **(A)** BI outcomes. **(B)** TER outcomes.

**Figure 4:**
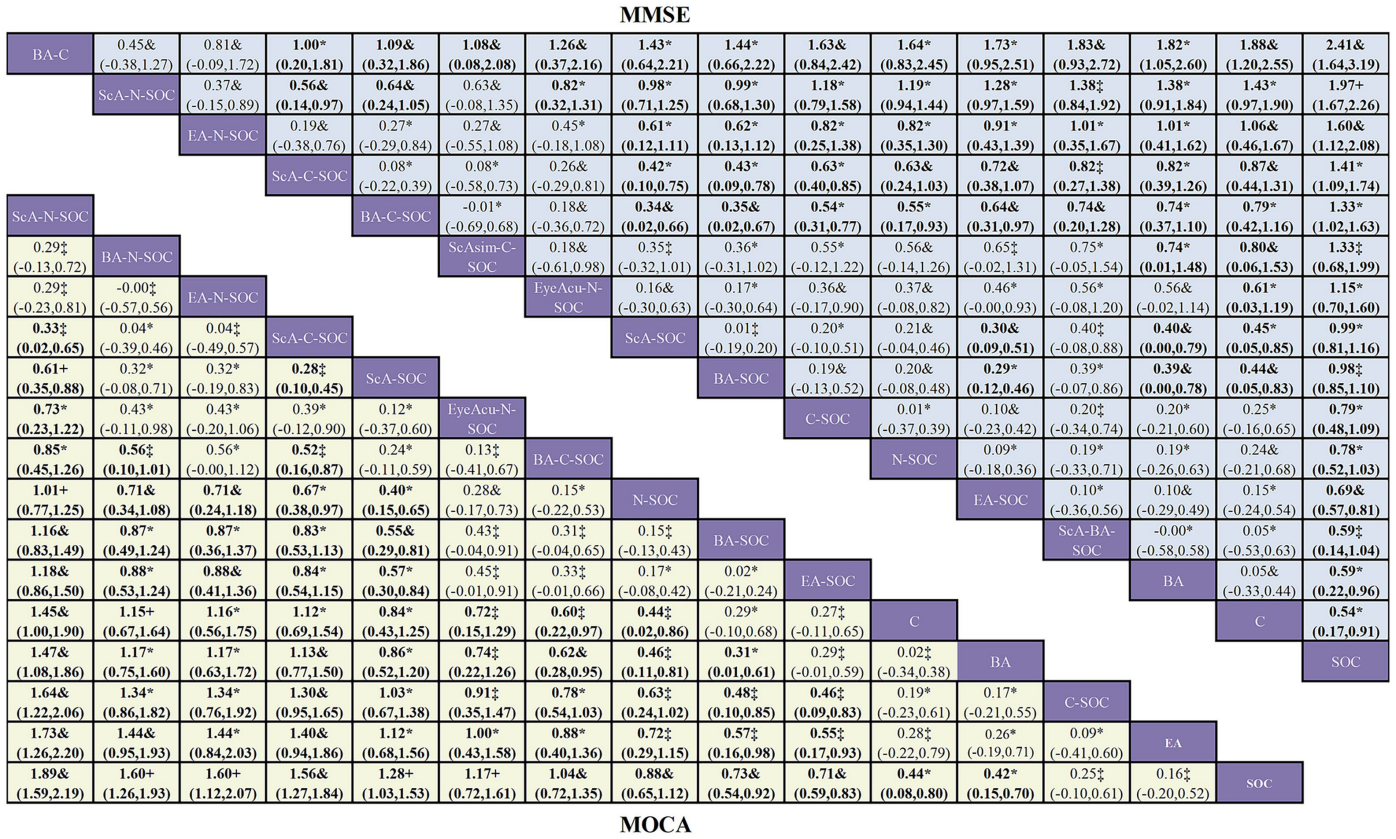
Network meta-analysis of acupuncture-based interventions for post-stroke cognitive impairment and certainty of evidence. The matrix presents the league table of the network meta-analysis, summarizing all pairwise comparisons between acupuncture-related treatment strategies. The upper triangular section reports SMDs with corresponding 95% CIs for MMSE outcomes, whereas the lower triangular section displays SMDs and 95% CIs for MoCA outcomes. An SMD > 0 indicates that the row intervention performs better than the column intervention; conversely, an SMD < 0 favors the column intervention. Values shown in bold denote statistically significant differences, with 95% CIs not crossing zero. Certainty of evidence was graded using the CINeMA (Confidence in Network Meta-analysis) framework: High^+^ indicates high certainty, Moderate& denotes moderate certainty, Low* reflects low certainty, and Very low‡ represents very low certainty.

**Figure 5:**
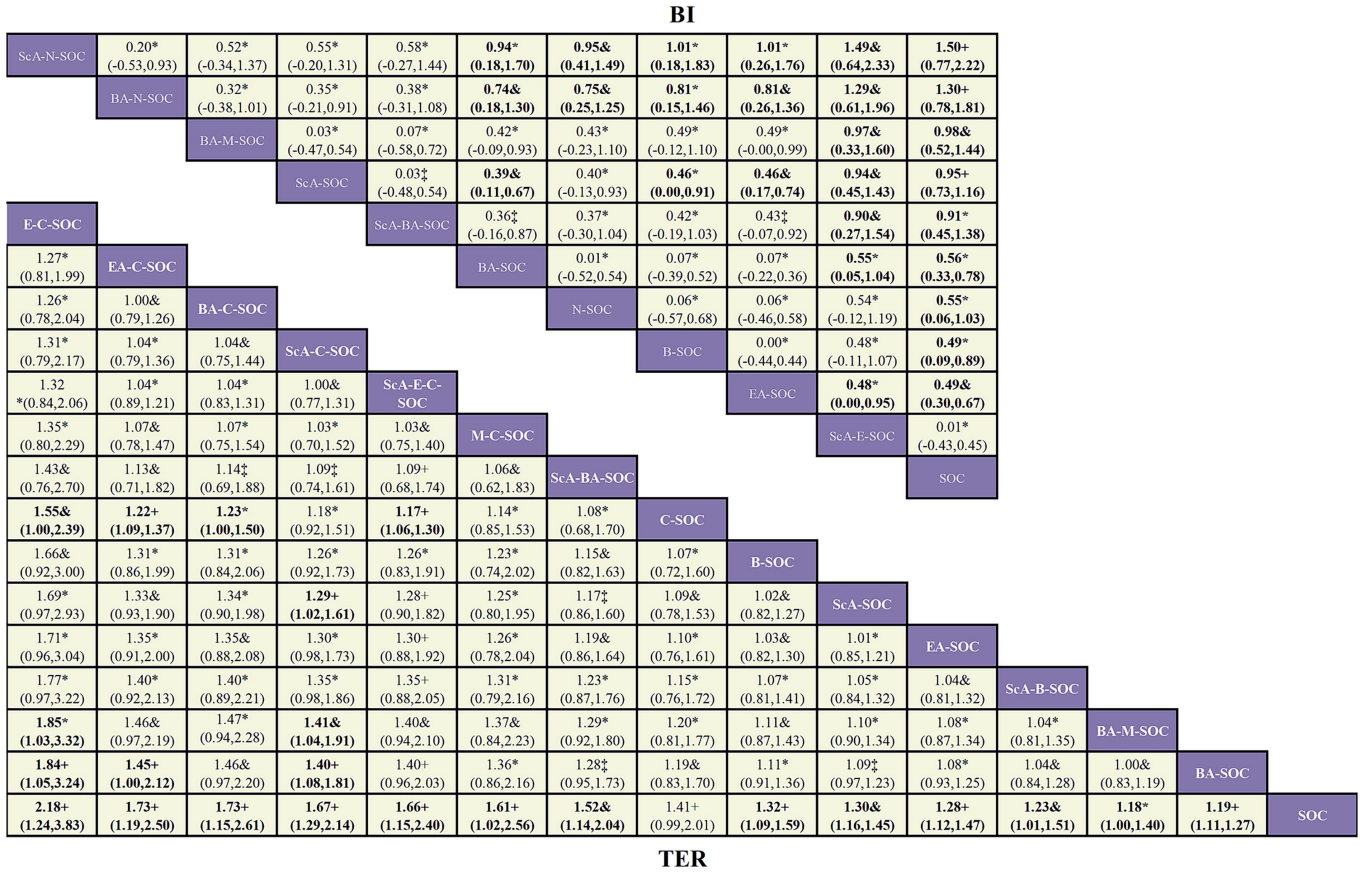
Network meta-analysis of acupuncture interventions for activities-of-daily-living outcomes in patients with post-stroke cognitive impairment and certainty of evidence. The matrix displays the league table summarizing all pairwise comparisons across acupuncture-based treatment strategies for functional independence. The upper triangular section reports standardized mean differences (SMDs) with corresponding 95% confidence intervals (CIs) for Barthel Index (BI) outcomes, whereas the lower triangular section presents pairwise comparisons of total effective rate (TER) expressed as the risk ratio (RRs) with corresponding 95% CIs. An RR > 1 indicates that the row intervention is superior to the column intervention, whereas an RR < 1 indicates the opposite. Bold values indicate statistical significance, with 95% CIs not crossing the null value. Certainty of evidence was graded according to the CINeMA (Confidence in Network Meta-analysis) framework: High+ indicates high certainty, Moderate& denotes moderate certainty, Low* reflects low certainty, and Very low‡ represents very low certainty.

In this study, the MoCA and MMSE served as the primary end points, whereas BI and TER were defined as secondary outcomes (Figures 2–3). All outcome networks constituted closed loops, enabling the evaluation of global inconsistency. The global inconsistency tests produced *p* values > 0.05 across all models (Supplementary Table 4), indicating that the assumption of network coherence was upheld. Local inconsistency was further examined using node-splitting analyses, and all comparisons likewise demonstrated *p* > 0.05 (Supplementary Tables 5–8). In addition, loop-specific inconsistency assessments showed that the 95% confidence intervals of inconsistency factors all crossed zero, confirming good concordance between direct and indirect evidence and supporting the overall coherence of the network. For the MoCA outcome, 38 randomized controlled trials involving 15 intervention strategies were included. Compared with standard of care (SOC), several combined acupuncture approaches demonstrated significantly greater improvements in MoCA scores, including ScA-N-SOC (SMD = 1.89, 95% CI: 1.59–2.19), BA-N-SOC (SMD = 1.60, 95% CI: 1.26–1.93), EA-N-SOC (SMD = 1.60, 95% CI: 1.12–2.07), and ScA-C-SOC (SMD = 1.56, 95% CI: 1.27–1.84); For MMSE, 48 studies encompassing 18 intervention strategies were analyzed. Relative to SOC, several combined acupuncture modalities produced significantly superior improvements in MMSE performance, including BA-C (SMD = 2.41, 95% CI: 1.64–3.19), ScA-N-SOC (SMD = 1.97, 95% CI: 1.67–2.26), EA-N-SOC (SMD = 1.60, 95% CI: 1.12–2.08), and ScA-C-SOC (SMD = 1.41, 95% CI: 1.09–1.74). These findings reinforce the enhanced efficacy of acupuncture-based multimodal rehabilitation in promoting global cognitive recovery after stroke; For BI, 18 studies involving 11 intervention strategies were incorporated. Compared with SOC, several acupuncture-inclusive combinations generated significantly larger gains in BI scores, such as ScA-N-SOC (SMD = 1.50, 95% CI: 0.77–2.22), BA-N-SOC (SMD = 1.30, 95% CI: 0.78–1.81), BA-M-SOC (SMD = 0.98, 95% CI: 0.52–1.44), and ScA-SOC (SMD = 0.95, 95% CI: 0.73–1.16). These improvements suggest enhanced functional independence and daily self-care capacity in patients with post-stroke cognitive impairment; For TER, 27 studies across 15 intervention strategies were analyzed. Compared with SOC, several combined acupuncture regimens exhibited substantially higher total effective rates, including E-C-SOC (RR = 2.18, 95% CI: 1.24–3.83), EA-C-SOC (RR = 1.73, 95% CI: 1.19–2.50), BA-C-SOC (RR = 1.73, 95% CI: 1.15–2.61), and ScA-C-SOC (RR = 1.67, 95% CI: 1.29–2.14). These results suggest that multimodal acupuncture and integrated traditional-Western therapeutic approaches may confer significant advantages in improving overall clinical effectiveness in patients with post-stroke cognitive impairment (Figures 4–5).

### 3.3 SUCRA rankings

Surface under the cumulative ranking curve (SUCRA) values were used to estimate the probability that each intervention represented the most effective regimen (Supplementary Tables 9–12; Supplementary Figures 2–5). For MoCA results, the SUCRA value of ScA-N-SOC is the highest (98.2%), indicating that it is most likely to be the best intervention, followed by BA-N-SOC (86.1%), EA-N-SOC (85.5%) and ScA-C-SOC (84.2%); For MMSE, BA-C ranked first (SUCRA = 98.6%), ScA-N-SOC (93.4%), EA-N-SOC (82.2%), and ScA-C-SOC (75.8%) ranked in order Name. For BI results, ScA-N-SOC showed the highest SUCRA probability (93.9%), indicating that it has the greatest potential for enhancing functional independence, followed by BA-N-SOC (88.6%), BA-M-SOC (71.9%), and ScA-SOC (71%); For TER results, E-C-SOC obtained the highest SUCRA value (92.4%), indicating that it is most likely to provide the most favorable therapeutic effect, followed by EA-C-SOC (78.3%), BA-C-SOC (77.2%), and ScA-C-SOC (74.5%).

### 3.4 Sensitivity analyses, meta-regression, and publication bias

We used the retention method to conduct a sensitivity analysis of all outcome indicators (MoCA, MMSE, BI, and TER) to evaluate the impact of a single trial on the overall network estimate. In each iteration, eliminate one study and re-analyze the remaining data using a consistency-based random effect network meta-analysis model. The elimination of any trial did not substantially change the direction or magnitude of the effect estimate of acupuncture intervention compared with SOC. The change in the effect quantity is extremely small, the 95% confidence interval basically overlaps, and the statistical significance is maintained, indicating that the research results are stable in all outcome indicators.

In order to further explore the potential determinants of heterogeneity, we conducted a single-variate network meta-regression analysis with geographical area and follow-up time as covariables (Supplementary Tables 17–24). The results show that these two covariates are not statistically related to the relative efficacy of any acupuncture and moxibustion scheme relative to SOC, indicating that these factors have no significant impact on the intervention effect.

We checked the publication bias of MoCA, MMSE, BI, and TER through the funnel diagram (Supplementary Figures 6–9). The funnel diagram shows that the distribution is roughly symmetrical, and no obvious small sample effects or extreme anomalies are found, indicating that the publication bias included in the study is less likely.

### 3.5 GRADE assessment

Using the CINeMA framework to evaluate the evidence certainty of the main outcome, the distribution of the evidence certainty level of different outcomes varies significantly, ranging from high to low. For MoCA, among 105 pairwise comparisons, 7 (6.7%) were graded as high certainty,23 (21.9%) as moderate, 45 (42.9%) as low, and 30 (28.6%) as very low; For MMSE, of 120 comparisons, 1 (0.8%) achieved high certainty, 44 (36.7%) were rated as moderate, 65 (54.2%) as low, and 10 (8.3%) as very low; For BI, among 55 comparisons, 3 (5.5%) were assessed as high certainty, 13 (23.6%) as moderate,36 (65.5%) as low, and 3 (5.5%) as very low; For TER, among 105 comparisons, 20 (19.0%) were graded as high certainty, 31 (29.5%) as moderate, 49 (46.7%) as low, and 5 (4.8%) as very low. Complete CINeMA ratings for each outcome are provided in Supplementary Tables 30–34; Supplementary Figures 12–16.

## 4 Discussion

### 4.1 Significance and innovation of the study

PSCI is a common and clinically significant complication that markedly worsens long-term outcomes. Although acupuncture has been widely used in stroke rehabilitation, the relative efficacy of different acupuncture and moxibustion therapies is still unclear due to the lack of direct, head-to-head randomized controlled trials. For the first time, this study adopted a network meta-analysis framework to conduct a comprehensive PSCI evaluation of 25 acupuncture-centered multi-mode interventions. Our analysis is based on 70 randomized controlled trials, including a total of 6,259 subjects, providing a reliable evidence basis for integrating direct and indirect comparisons, thus supporting the development of more individualized and evidence-based rehabilitation strategies for PSCI patients.

### 4.2 Main findings

Network meta-analysis clarified the relative ranking of treatment effects across multiple clinical outcome measures.

First, in terms of improvement in Montreal Cognitive Assessment (MoCA) scores, scalp acupuncture combined with non-invasive brain stimulation (ScA-N-SOC) demonstrated the most favorable effect. This finding suggests that acupuncture strategies targeting cortical functional regions, when integrated with non-invasive brain stimulation, may exert synergistic benefits in modulating higher-order cognitive domains, particularly attention, executive function, and information integration. Second, regarding Mini-Mental State Examination (MMSE) outcomes, both body acupuncture combined with cognitive rehabilitation training (BA-C) and scalp acupuncture combined with non-invasive brain stimulation (ScA-N-SOC) showed relatively strong efficacy. These results indicate that combining peripheral acupoint stimulation with structured cognitive training or central neuromodulation approaches may provide complementary advantages in improving global cognitive status. Third, with respect to Barthel Index (BI) outcomes, scalp acupuncture combined with non-invasive brain stimulation (ScA-N-SOC) and body acupuncture combined with non-invasive brain stimulation (BA-N-SOC) were associated with significant improvements in activities of daily living. This supports the notion that integrating acupuncture with modulation of central nervous system excitability may facilitate the translation of cognitive improvement into functional independence. Finally, in terms of total effective rate (TER), auricular acupuncture combined with cognitive rehabilitation training (E-C-SOC) ranked highest, suggesting that combined strategies emphasizing sensory input and cognitive-behavioral interventions may enhance overall clinical response.

In general, different acupuncture-based multimodal rehabilitation strategies exhibited differential advantages across specific outcome domains. These findings suggest that stratified selection of combined interventions according to targeted cognitive dimensions and functional recovery goals may better align with the clinical management needs of patients with post-stroke cognitive impairment.

### 4.3 Mechanistic interpretation

The potential mechanisms underlying the beneficial effects of acupuncture on post-stroke cognitive impairment (PSCI) may involve multilevel central nervous system modulation, including improvements in cerebral blood perfusion, enhancement of synaptic plasticity, and regulation of neuroinflammatory and oxidative stress responses. Previous preclinical and clinical studies have shown that acupuncture stimulation can upregulate the expression of brain-derived neurotrophic factor (BDNF) and synapse-related proteins, promote hippocampal neuronal repair and synaptic remodeling, and suppress pro-inflammatory cytokines such as IL-1β and TNF-*α*, thereby providing a biological basis for cognitive recovery (22).

Building on these findings, the relatively stable advantages observed for acupuncture combined with non-invasive brain stimulation (NIBS) in the present study may be attributed to a synergistic “peripheral-central” dual-channel neuromodulatory mechanism. On the one hand, acupuncture delivers peripheral sensory input that modulates thalamocortical circuits and cognition-related neural networks. On the other hand, NIBS techniques such as repetitive transcranial magnetic stimulation (rTMS) or transcranial direct current stimulation (tDCS) can directly influence cortical excitability and neural network plasticity, thereby amplifying the central regulatory effects initiated by acupuncture. This complementary mechanism provides a plausible explanation for the relative advantages of ScA-N-SOC and BA-N-SOC observed across multiple cognitive and functional outcomes in this study (23).

In recent years, electroencephalography (EEG)-based studies have further provided objective neurophysiological evidence supporting the central modulatory effects of acupuncture. Multiple investigations have demonstrated that acupuncture stimulation can significantly alter EEG rhythmic power and functional connectivity patterns, with consistent changes particularly observed in the *δ* and *α* frequency bands. These alterations are often accompanied by enhanced interhemispheric functional connectivity and improved small-world network efficiency, suggesting that acupuncture may systematically reshape large-scale brain functional network organization (24).

Further studies incorporating periodic-aperiodic EEG parameterization have indicated that acupuncture not only affects conventional oscillatory power but also modulates the aperiodic component (aperiodic exponent), which reflects changes in the cortical excitation-inhibition balance. These findings provide more physiologically meaningful quantitative indicators for evaluating acupuncture-induced neural modulation (25). Consistent with electrophysiological evidence, neuroimaging studies have also shown that acupuncture intervention can induce activity remodeling in multiple cognition-related brain regions in patients with mild cognitive impairment, with activation changes in certain regions significantly correlated with improvements in cognitive scale scores, thereby offering spatial-level neuroimaging support for the cognitive benefits of acupuncture (26).

More importantly, recent advances in brain-computer interface (BCI) and deep learning research have demonstrated that different acupuncture manipulation states and stimulation patterns can be reliably distinguished and decoded from EEG signals. For example, Transformer-based models have achieved high accuracy in identifying distinct acupuncture operation states, indicating that acupuncture is not a non-specific stimulus but rather an intervention input with identifiable and quantifiable central neuromodulatory signatures (27). In addition, studies employing neural manifold analysis and representation learning have shown that acupuncture stimulation can generate stable and separable dynamic trajectories within low-dimensional neural state space, with different acupuncture manipulations corresponding to significantly distinct neural manifold structures. These findings provide emerging computational neuroscience evidence supporting the quantifiable central effects of acupuncture (28).

From the perspective of traditional Chinese medicine theory, previous studies have proposed that acupuncture strategies centered on Governor Vessel-related regions may be closely associated with brain functional regulation. This concept of “regulating the spirit through the Governor Vessel” offers an important theoretical background for acupuncture-based interventions in cognitive disorders. However, given the substantial heterogeneity in acupuncture techniques and manipulation protocols across studies, the present analysis did not treat this theoretical construct as an independent efficacy conclusion. Instead, related interventions were evaluated within a unified combined-intervention framework to avoid introducing excessive clinical heterogeneity into the network comparisons.

### 4.4 Comparison with previous research

The findings of the present study are generally consistent with previous systematic reviews and meta-analyses on acupuncture for post-stroke cognitive impairment (PSCI), which have concluded that acupuncture, as a non-pharmacological intervention, exerts certain beneficial effects on post-stroke cognitive function. However, compared with earlier studies, the present analysis further extends the evidence base in terms of both methodological approach and clinically informative comparisons.

For example, a meta-analysis by Luo et al., which included 29 randomized controlled trials, demonstrated that acupuncture was superior to pharmacological treatment in improving MoCA, MMSE, and Barthel Index outcomes in patients with PSCI (29). This study provided important support for the overall effectiveness of acupuncture in PSCI; however, it relied on conventional pairwise meta-analytic methods and treated different acupuncture modalities and combined strategies as a single homogeneous intervention category, thereby failing to further distinguish the relative effectiveness among specific techniques or combination patterns.

Similarly, a systematic review by Kuang et al. suggested that acupuncture may serve as a potentially effective adjunctive therapy for PSCI, while also noting substantial clinical heterogeneity and risk of bias among the included studies, resulting in an overall low certainty of evidence (30). These limitations are, to some extent, attributable to the inability of traditional meta-analytic methods to systematically compare multiple intervention strategies within a single analytical framework.

In contrast, the present study adopted a network meta-analysis approach to simultaneously compare the relative effects of multiple acupuncture-based combined interventions within a unified evidence framework and to generate probability-based rankings of treatment efficacy. This approach more closely reflects real-world clinical practice, in which multiple therapeutic options coexist and optimal selection is required. Importantly, the objective of this study was not to reiterate whether acupuncture is effective for PSCI, but rather to further address a clinically relevant question: among various acupuncture-based combined strategies, which interventions demonstrate relative advantages across different outcome domains, thereby compensating for the limitations of traditional pairwise meta-analyses in supporting clinical decision-making.

### 4.5 Implications and unresolved issues

The findings of the present study suggest that different acupuncture-based combined interventions may exert differential therapeutic effects by engaging distinct central neural circuits. Interventions combining scalp or body acupuncture with non-invasive brain stimulation may preferentially modulate cortico-thalamic pathways and higher-order cognitive networks, thereby conferring relative advantages in improving cognitive function and functional independence. In contrast, acupuncture combined with cognitive rehabilitation training may enhance neuroplastic processes through behaviorally driven learning mechanisms, contributing more prominently to improvements in overall clinical response rates.

However, due to residual heterogeneity among the included studies in terms of acupuncture manipulation details, stimulation parameters, and rehabilitation training protocols, the specific modes of action underlying the differential effects of various combined strategies across multidimensional outcomes remain incompletely understood. Future studies integrating neuroimaging and neurophysiological techniques, such as functional magnetic resonance imaging and electroencephalography, will be necessary to further elucidate the neurobiological basis of the differential efficacy observed among acupuncture-based multimodal interventions.

### 4.6 Strengths of the present study

This network meta-analysis focusing on post-stroke cognitive impairment (PSCI) demonstrates several notable strengths across multiple dimensions. By strictly restricting inclusion to PSCI populations, a high degree of sample homogeneity was ensured, thereby enhancing the internal validity of the pooled estimates. In terms of intervention construction, a standardized framework comprising 25 acupuncture-based combination nodes was established for the first time, which substantially reduced classification bias and improved the comparability among different intervention strategies. Methodologically, multiple complementary assessment tools—including RoB 2.0, the CINeMA framework, and SUCRA probability ranking—were jointly applied to evaluate the robustness and methodological rigor of the findings from multiple perspectives. The selected outcome measures comprehensively covered key clinical domains, including cognitive function, activities of daily living, and total effective rate, thereby enhancing the overall clinical relevance and applicability of the results. Notably, this study proposed an “acupuncture-brain stimulation coordination” intervention model, offering new perspectives for integrative rehabilitation combining traditional Chinese and Western medicine and indicating potential pathways for clinical translation.

### 4.7 Limitations and future perspectives

Although this network meta-analysis integrated a substantial body of available evidence, several limitations should be acknowledged.

First, due to the inherent characteristics of acupuncture interventions, complete double-blinding is difficult to achieve, which may introduce performance bias. Accordingly, some included trials were assessed as having “some concerns” regarding deviations from intended interventions in the RoB 2 evaluation, and were subsequently downgraded in the CINeMA-based certainty of evidence assessment.

Second, given the cultural and clinical context of acupuncture practice, the majority of included studies were conducted in China, which may limit the generalizability of the findings to other populations and healthcare settings. Nevertheless, this study systematically searched four major international databases and ultimately included 70 randomized controlled trials, providing a relatively comprehensive synthesis of the available evidence.

Third, variability in acupuncture techniques and operator expertise across studies is inevitable and may contribute to heterogeneity in treatment effects. However, the large overall sample size (6,259 participants), together with consistent results from sensitivity analyses, supports the robustness and stability of the main findings.

Future research should prioritize high-quality, multicenter randomized controlled trials with larger sample sizes and extended follow-up periods. The integration of objective measures, such as neuroimaging and biological markers, will be essential for further elucidating the mechanisms underlying acupuncture-based interventions in PSCI rehabilitation and for promoting their clinical application toward greater standardization and personalizatio.

## Data Availability

The original contributions presented in the study are included in the article/Supplementary material, further inquiries can be directed to the corresponding author.
