# Supplementary material for: Comparative effectiveness of acupuncture-based multimodal rehabilitation for post-stroke cognitive impairment: a network meta-analysis of 70 randomized controlled trials
Source: Front Neurol. 2026 Feb 10;17:1759572. doi: 10.3389/fneur.2026.1759572 (PMC12929128; doi:10.3389/fneur.2026.1759572)
Supplement: Supplementary file 1 [file Table_1.docx]

**Comparative Effectiveness of Acupuncture-Based Multimodal Rehabilitation for Post-Stroke Cognitive Impairment: A Network Meta-Analysis of 70 Randomized Controlled Trials**

| **Table of Contents** | | |
| --- | --- | --- |
| Title | Content | page |
| Table S1 | PRISMA NMA Checklist of Items to Include When Reporting a Systematic Review Involving a Network Meta-analysis | 3-7 |
| Table S2 | Literature Search Strategy | 8-11 |
| Table S3 | Acupuncture Baseline Characteristics of Studies Included in the Network Meta-Analysis | 12-62 |
| Table S4 | Results of the Global Inconsistency Assessment | 63 |
| Table S5 | Node-Splitting Inconsistency Analysis for MoCA | 64-65 |
| Table S6 | Node-Splitting Inconsistency Analysis for MMSE | 66-67 |
| Table S7 | Node-Splitting Inconsistency Analysis for BI | 68 |
| Table S8 | Node-Splitting Inconsistency Analysis for TER | 69 |
| Table S9 | SUCRA-Based Ranking Results for MoCA | 70 |
| Table S10 | SUCRA-Based Ranking Results for MMSE | 71 |
| Table S11 | SUCRA-Based Ranking Results for BI | 72 |
| Table S12 | SUCRA-Based Ranking Results for TER | 73 |
| Table S13 | Sensitivity Analysis for MoCA | 74-87 |
| Table S14 | Sensitivity Analysis for MMSE | 88-106 |
| Table S15 | Sensitivity Analysis for BI | 107-110 |
| Table S16 | Sensitivity Analysis for TER | 111-125 |
| Table S17 | Meta-Regression Analysis of MoCA by Region | 126-127 |
| Table S18 | Meta-Regression Analysis of MOCA by Follow-Up Duration | 128-129 |
| Table S19 | Meta-Regression Analysis of MMSE by Region | 130-132 |
| Table S20 | Meta-Regression Analysis of MMSE by Follow-Up Duration | 132-133 |
| Table S21 | Meta-Regression Analysis of BI by Region | 134 |
| Table S22 | Meta-Regression Analysis of BI by Follow-Up Duration | 135 |
| Table S23 | Meta-Regression Analysis of TER by Region | 136-137 |
| Table S24 | Meta-Regression Analysis of TER by Follow-Up Duration | 138-139 |
| Table S25 | GRADE Assessment for MoCA | 140-144 |
| Table S26 | GRADE Assessment for MMSE | 145-150 |
| Table S27 | GRADE Assessment for BI | 151-153 |
| Table S28 | GRADE Assessment for TER | 154-158 |
| Figure S1 | Risk of Bias Assessment for Randomized Controlled Trials | 159 |
| Figure S2 | SCURA Diagram of MOCA | 160 |
| Figure S3 | SCURA Diagram of MMSE | 160 |
| Figure S4 | SCURA Diagram of BI | 161 |
| Figure S5 | SCURA Diagram of TER | 161 |
| Figure S6 | The funnel plot of MOCA | 162 |
| Figure S7 | The funnel plot of MMSE | 162 |
| Figure S8 | The funnel plot of BI | 163 |
| Figure S9 | The funnel plot of TER | 163 |

Table S1 PRISMA NMA Checklist of Items to Include When Reporting a Systematic Review Involving a Network Meta-analysis

| **Section/Topic** | **Item #** | **Checklist Item** | **Reported on Page #** |
| --- | --- | --- | --- |
| **TITLE** |  |  |  |
| Title | 1 | Identify the report as a systematic review *incorporating*  *anetwork meta-analysis (or related form of meta-analysis).* | 1 |
|  |  |  |  |
| **ABSTRACT** |  |  |  |
| Structured summary | 2 | Provide a structured summary including, as applicable:  **Background:** main objectives  **Methods:** data sources; study eligibility criteria, participants, and interventions; study appraisal; and *synthesis methods, such as network meta-analysis.*  **Results:** number of studies and participants identified; summary estimates with corresponding confidence/credible intervals; *treatment rankings may also be discussed. Authors may choose to summarize pairwise comparisons against a chosen treatment included in their analyses for brevity.*  **Discussion/Conclusions:** limitations; conclusions and implications of findings.  **Other:** systematic review registration number with registry name. | 1-2 |
|  |  |  |  |
| **INTRODUCTION** |  |  |  |
| Rationale | 3 | Describe the rationale for the review in the context of what is already known*, including mention of why a network meta-analysis has been conducted.* | 3-4 |
| Objectives | 4 | Provide an explicit statement of questions being addressed, with reference to participants, interventions, comparisons, outcomes, and study design (PICOS). | 4 |
|  |  |  |  |
| **METHODS** |  |  |  |
| Protocol and registration | 5 | Indicate whether a review protocol exists and if and where it can be accessed (e.g., Web address); and, if available, provide registration information, including registration number. | 4 |
| Eligibility criteria | 6 | Specify study characteristics (e.g., PICOS, length of follow-up) and report characteristics (e.g., years considered, language, publication status) used as criteria for eligibility, giving rationale. *Clearly describe eligible treatments included in the treatment network, and note whether any have been clustered or merged into the same node (with justification).* | 4-5 |
| Information sources | 7 | Describe all information sources (e.g., databases with dates of coverage, contact with study authors to identify additional studies) in the search and date last searched. | 4 |
| Search | 8 | Present full electronic search strategy for at least one database, including any limits used, such that it could be repeated. | 4，Supplementary Table2 |
| Study selection | 9 | State the process for selecting studies (i.e., screening, eligibility, included in systematic review, and, if applicable, included in the meta-analysis). | 4-5 |
| Data collection process | 10 | Describe method of data extraction from reports (e.g., piloted forms, independently, in duplicate) and any processes for obtaining and confirming data from investigators. | 5-6 |
| Data items | 11 | List and define all variables for which data were sought (e.g., PICOS, funding sources) and any assumptions and simplifications made. | 5 |
| **Geometry of the network** | **S1** | Describe methods used to explore the geometry of the treatment network under study and potential biases related to it. This should include how the evidence base has been graphically summarized for presentation, and what characteristics were compiled and used to describe the evidence base to readers. | 5-6 |
| Risk of bias within individual studies | 12 | Describe methods used for assessing risk of bias of individual studies (including specification of whether this was done at the study or outcome level), and how this information is to be used in any data synthesis. | 5 |
| Summary measures | 13 | State the principal summary measures (e.g., risk ratio, difference in means). *Also describe the use of additional summary measures assessed, such as treatment rankings and surface under the cumulative ranking curve (SUCRA) values, as well as modified approaches used to present summary findings from meta-analyses.* | 5-6 |
| Planned methods of analysis | 14 | Describe the methods of handling data and combining results of studies for each network meta-analysis. This should include, but not be limited to:   - *Handling of multi-arm trials;* - *Selection of variance structure;* - *Selection of prior distributions in Bayesian analyses; and* - *Assessment of model fit.* | 6 |
| **Assessment of Inconsistency** | **S2** | Describe the statistical methods used to evaluate the agreement of direct and indirect evidence in the treatment network(s) studied. Describe efforts taken to address its presence when found. | 6 |
| Risk of bias across studies | 15 | Specify any assessment of risk of bias that may affect the cumulative evidence (e.g., publication bias, selective reporting within studies). | **7** |
| Additional analyses | 16 | Describe methods of additional analyses if done, indicating which were pre-specified. This may include, but not be limited to, the following:   - Sensitivity or subgroup analyses; - Meta-regression analyses; - *Alternative formulations of the treatment network; and* - *Use of alternative prior distributions for Bayesian analyses (if applicable).* | **7** |
| **RESULTS†** |  |  |  |
| Study selection | 17 | Give numbers of studies screened, assessed for eligibility, and included in the review, with reasons for exclusions at each stage, ideally with a flow diagram. | 8-10，Figure1 |
| **Presentation of network structure** | **S3** | Provide a network graph of the included studies to enable visualization of the geometry of the treatment network. | 11，Figure2-3 |
| **Summary of network geometry** | **S4** | Provide a brief overview of characteristics of the treatment network. This may include commentary on the abundance of trials and randomized patients for the different interventions and pairwise comparisons in the network, gaps of evidence in the treatment network, and potential biases reflected by the network structure. | 11 |
| Study characteristics | 18 | For each study, present characteristics for which data were extracted (e.g., study size, PICOS, follow-up period) and provide the citations. | 8-9，Supplementary Table 3 |
| Risk of bias within studies | 19 | Present data on risk of bias of each study and, if available, any outcome level assessment. | 9,Supplementary Figure.1 |
| Results of individual studies | 20 | For all outcomes considered (benefits or harms), present, for each study: 1) simple summary data for each intervention group, and 2) effect estimates and confidence intervals. *Modified approaches may be needed to deal with information from larger networks.* | 17-18，Supplementary Table4，Supplementary Table 5-9 |
| Synthesis of results | 21 | Present results of each meta-analysis done, including confidence/credible intervals. *In larger networks, authors may focus on comparisons versus a particular comparator (e.g. placebo or standard care), with full findings presented in an appendix. League tables and forest plots may be considered to summarize pairwise comparisons.* If additional summary measures were explored (such as treatment rankings), these should also be presented. | 12-16,Figure 4–6 |
| **Exploration for inconsistency** | **S5** | Describe results from investigations of inconsistency. This may include such information as measures of model fit to compare consistency and inconsistency models, *P* values from statistical tests, or summary of inconsistency estimates from different parts of the treatment network. | 17,Supplementary Table 10–14; Supplementary Figure 2–6 |
| Risk of bias across studies | 22 | Present results of any assessment of risk of bias across studies for the evidence base being studied. | 18-19,Supplementary Figure 7-11 |
| Results of additional analyses | 23 | Give results of additional analyses, if done (e.g., sensitivity or subgroup analyses, meta-regression analyses*, alternative network geometries studied, alternative choice of prior distributions for Bayesian analyses,* and so forth). | 18-19,Supplementary Table20-29,  Supplementary Table30–34 ,Supplementary  Figure 12–16 |
|  |  |  |  |
| **DISCUSSION** |  |  |  |
| Summary of evidence | 24 | Summarize the main findings, including the strength of evidence for each main outcome; consider their relevance to key groups (e.g., healthcare providers, users, and policy-makers). | 19-20 |
| Limitations | 25 | Discuss limitations at study and outcome level (e.g., risk of bias), and at review level (e.g., incomplete retrieval of identified research, reporting bias). *Comment on the validity of the assumptions, such as transitivity and consistency. Comment on any concerns regarding network geometry (e.g., avoidance of certain comparisons).* | 20-22 |
| Conclusions | 26 | Provide a general interpretation of the results in the context of other evidence, and implications for future research. | 23 |
|  |  |  |  |
| **FUNDING** |  |  |  |
| Funding | 27 | Describe sources of funding for the systematic review and other support (e.g., supply of data); role of funders for the systematic review. This should also include information regarding whether funding has been received from manufacturers of treatments in the network and/or whether some of the authors are content experts with professional conflicts of interest that could affect use of treatments in the network. | NA |

PICOS = population, intervention, comparators, outcomes, study design.

* Text in italics indicate S wording specific to reporting of network meta-analyses that has been added to guidance from the PRISMA statement.

† Authors may wish to plan for use of appendices to present all relevant information in full detail for items in this section.

| **Table S2.Literature SElectroacupuncturerch Strategy** | |
| --- | --- |
| Pubmed | ("Stroke"[MeSH] OR "Strokes" OR "Cerebrovascular Accident" OR "Cerebrovascular Accidents" OR"Cerebral Stroke" OR "Cerebral Strokes" OR "Stroke, Cerebral" OR "Strokes, Cerebral" OR"Cerebrovascular Apoplexy" OR "Apoplexy, Cerebrovascular" OR "Vascular Accident, Brain" OR"Brain Vascular Accident" OR "Brain Vascular Accidents" OR "Vascular Accidents, Brain" OR"Cerebrovascular Stroke" OR "Cerebrovascular Strokes" OR "Stroke, Cerebrovascular" OR"Strokes, Cerebrovascular" OR "Apoplexy" OR "CVA (Cerebrovascular Accident)" OR"CVAs (Cerebrovascular Accident)" OR "Stroke, Acute" OR "Acute Stroke" OR "Acute Strokes" OR"Strokes, Acute" OR "Cerebrovascular Accident, Acute" OR "Acute Cerebrovascular Accident" OR"Acute Cerebrovascular Accidents" OR "Cerebrovascular Accidents, Acute")  AND  ("Cognitive Impairment"[MeSH] OR "Cognitive Dysfunctions" OR "Dysfunction, Cognitive" OR"Dysfunctions, Cognitive" OR "Cognitive Disorder" OR "Cognitive Disorders" OR"Disorder, Cognitive" OR "Disorders, Cognitive" OR "Cognitive Impairments" OR"Cognitive Impairment" OR "Impairment, Cognitive" OR "Impairments, Cognitive" OR"Mild Cognitive Impairment" OR "Cognitive Impairment, Mild" OR "Cognitive Impairments, Mild" OR"Impairment, Mild Cognitive" OR "Impairments, Mild Cognitive" OR "Mild Cognitive Impairments" OR"Cognitive Decline" OR "Cognitive Declines" OR "Decline, Cognitive" OR "Declines, Cognitive" OR"Mental Deterioration" OR "Deterioration, Mental" OR "Deteriorations, Mental" OR"Mental Deteriorations")  AND  ("Acupuncture Therapy"[MeSH] OR "Acupuncture TrElectroacupuncturetment" OR "Acupuncture TrElectroacupuncturetments" OR"TrElectroacupuncturetment, Acupuncture" OR "Therapy, Acupuncture" OR "Pharmacoacupuncture TrElectroacupuncturetment" OR"TrElectroacupuncturetment, Pharmacoacupuncture" OR "Pharmacoacupuncture Therapy"OR"Therapy, Pharmacoacupuncture" OR "Acupotomy" OR "Acupotomies")  AND  ("Randomized Controlled Trial"[Publication Type] OR "Randomized Controlled Trials as Topic"[MeSH] OR "RCT" OR "randomized") |
|  |  |
|  |  |
|  |  |
|  |  |
|  |  |
|  |  |
| Web of Science | TS=("stroke" OR "cerebrovascular accident" OR "acute stroke") AND TS=("cognitive impairment" OR "cognitive dysfunction" OR "mild cognitive impairment" OR "cognitive decline") AND TS=("acupuncture" OR "acupuncture therapy" OR "electroacupuncture" OR "pharmacoacupuncture" OR "acupotomy") AND TS=("randomized controlled trial" OR "randomized" OR "random*" OR "placebo" OR "trial") Filters applied: Document Type: Article, Language: English, Timespan: All yElectroacupuncturers |
|  |  |
|  |  |
|  |  |
|  |  |
|  |  |
|  |  |
|  |  |
| Cochrane | (MeSH descriptor: [Stroke] explode all trees OR stroke* OR cerebrovascular accident* OR apoplex* OR brain ischem* OR cerebral infarct* OR intracerebral hemorrhag* OR CVA*) AND (MeSH descriptor: [Cognitive Dysfunction] explode all trees OR cognit* NElectroacupunctureR/3 (impair* OR declin* OR dysfunction* OR deteriorat*) OR mild cognitive impairment OR vascular dementia OR post-stroke cognitive impairment OR PSCI) AND (MeSH descriptor: [Acupuncture Therapy] explode all trees OR acupunct* OR electroacupunct* OR electro-acupunct* OR Scalp acupuncturelp acupuncture OR hElectroacupunctured acupuncture OR warm needl* OR moxibust* OR pharmacoacupunct* OR acupotom*) AND (random* OR placebo OR sham) Filters applied: Document Type: Article, Language: English, Timespan: All yElectroacupuncturers |
|  |  |
|  |  |
|  |  |
| EmBody acupuncturese | ('stroke'/exp OR 'stroke':ti,ab OR 'cerebrovascular accident'/exp OR 'cerebrovascular accident':ti,ab OR 'brain ischemia'/exp OR 'brain ischemia':ti,ab OR 'cerebral infarction'/exp OR 'cerebral infarction':ti,ab OR'intracranial hemorrhage'/exp OR 'intracranial hemorrhage':ti,ab) AND ('cognitive impairment'/exp OR 'cognitive impairment':ti,ab OR 'mild cognitive impairment'/exp OR 'mild cognitive impairment':ti,ab OR 'dementia'/exp OR 'dementia':ti,ab OR 'vascular dementia'/exp OR 'vascular dementia':ti,ab OR 'cognitive dysfunction'/exp OR 'cognitive dysfunction':ti,ab)  AND ('acupuncture'/exp OR 'acupuncture':ti,ab OR 'electroacupuncture'/exp OR 'electroacupuncture':ti,ab OR 'Scalp acupuncturelp acupuncture'/exp OR 'Scalp acupuncturelp acupuncture':ti,ab OR 'warm acupuncture'/exp OR 'warm acupuncture':ti,ab OR 'fire needle':ti,ab) AND ('randomized controlled trial'/exp OR 'randomized controlled trial':ti,ab OR 'rct':ti,ab) |
|  |  |
|  |  |
|  |  |
|  |  |
|  |  |
|  |  |
|  |  |
| CNKI | (SU = (stroke + apoplexy + ischemic stroke + hemorrhagic stroke + acute stroke + cerebrovascular accident + cerebrovascular incident + cerebral infarction + cerebral hemorrhage))  AND  (SU = (cognitive impairment + mild cognitive impairment + cognitive decline + cognitive dysfunction + mild cognitive dysfunction + memory impairment + intellectual deterioration))  AND  (SU = (acupuncture + acupuncture therapy + electroacupuncture + body acupuncture + Scalp acupuncturelp acupuncture + acupoint injection + meridian point injection + pharmacoacupuncture + acupotomy + needle-knife therapy))  AND  (TI = (randomized controlled trial + randomization + randomized + RCT)  OR AB = (randomized controlled trial + randomization + randomized + RCT)OR KY = (randomized controlled trial + randomization + randomized + RCT)OR SU = (randomized controlled trial + randomization + randomized + RCT)) |
|  |  |
|  |  |
|  |  |
|  |  |
|  |  |
| Wanfang Data | ((TI = ("cerebrovascular accident" OR stroke OR apoplexy OR "ischemic stroke" OR "hemorrhagic stroke" OR "acute stroke" OR "cerebral infarction" OR "cerebral hemorrhage"))OR (KY = ("cerebrovascular accident" OR stroke OR apoplexy OR "ischemic stroke" OR "hemorrhagic stroke" OR "acute stroke" OR "cerebral infarction" OR "cerebral hemorrhage"))OR (AB = ("cerebrovascular accident" OR stroke OR apoplexy OR "ischemic stroke" OR "hemorrhagic stroke" OR "acute stroke" OR "cerebral infarction" OR "cerebral hemorrhage")))  AND  ((TI = ("cognitive impairment" OR "mild cognitive impairment" OR "cognitive dysfunction" OR "mild cognitive dysfunction" OR "cognitive decline" OR "cognitive deterioration" OR "memory impairment" OR MCI))OR (KY = ("cognitive impairment" OR "mild cognitive impairment" OR "cognitive dysfunction" OR "mild cognitive dysfunction" OR "cognitive decline" OR "cognitive deterioration" OR "memory impairment" OR MCI))OR (AB = ("cognitive impairment" OR "mild cognitive impairment" OR "cognitive dysfunction" OR "mild cognitive dysfunction" OR "cognitive decline" OR "cognitive deterioration" OR "memory impairment" OR MCI)))  AND  ((TI = (acupuncture OR "acupuncture therapy" OR electroacupuncture OR "Scalp acupuncturelp acupuncture" OR "body acupuncture" OR "acupoint injection" OR "meridian point injection" OR pharmacoacupuncture OR acupotomy OR "needle-knife therapy"))OR (KY = (acupuncture OR "acupuncture therapy" OR electroacupuncture OR "Scalp acupuncturelp acupuncture" OR "body acupuncture" OR "acupoint injection" OR "meridian point injection" OR pharmacoacupuncture OR acupotomy OR "needle-knife therapy"))OR (AB = (acupuncture OR "acupuncture therapy" OR electroacupuncture OR "Scalp acupuncturelp acupuncture" OR "body acupuncture" OR "acupoint injection" OR "meridian point injection" OR pharmacoacupuncture OR acupotomy OR "needle-knife therapy")))  AND  ((TI = (random* OR RCT))OR (KY = (random* OR RCT))OR (AB = (random* OR RCT))) |
|  |  |
|  |  |
|  |  |

Table 1. Studies Included in the Network Meta-Analysis

| Table S3. Acupuncture Baseline Characteristics of Studies Included in the Network Meta-Analysis | | | | | | | | |
| --- | --- | --- | --- | --- | --- | --- | --- | --- |
| First Author and Publication Year | Experimental/Control Sample Size | Age(Median/Mean, y) | Male/Female Ratio | Geographic Region | Follow-up Duration(weeks) | Experimental Intervention(s) | Control Intervention(s) |  |
| Roukeyanmu Yusufu 2022 [1] | 32/31 | 59.03 ± 7.65 / 58.05 ± 7.04 | 37 / 26 | Xinjiang, Urumqi | 8 | Body acupuncture–Standard of care(acupuncture + standard treatment group: acupuncture at Shenting, Baihui, Dazhui, Mingmen, Shenshu, Shenmen, and Xuanzhong + standard treatment [blood pressure and blood glucose control; monitoring of vital signs, hearing, and respiration; management of abnormal symptoms; and oral oxiracetam capsules]). | Standard of care (standard treatment group: blood pressure and blood glucose control; monitoring of vital signs, hearing, and respiration; targeted management of abnormal symptoms; and oral administration of oxiracetam capsules). |  |
| Jiang Xiaofen 2025 [2] | 42/42 | 60.4 ± 8.9 / 61.5 ± 7.4 | 51 / 33 | Zhejiang, Dongyang | 4 | Body acupuncture–Cognitive Training–Standard of care (acupuncture + cognitive training + symptomatic treatment group:“Tongdu-Xingshen” acupuncture applied at Yintang, Baihui, Shenting, Fengfu, and Shuigou, with auxiliary points including bilateral Xuehai and Geshu + cognitive training [memory training, attention training, calculation training, repetition and naming training, thinking training, and visuospatial structural ability training] + routine symptomatic treatment [control of blood pressure, blood lipids, and blood glucose; improvement of cerebral circulation; and neuroprotection]). | Cognitive Training–Standard of care(cognitive training + symptomatic treatment group: cognitive training [memory training, attention training, calculation training, repetition and naming training, thinking training, and visuospatial structural ability training] + routine symptomatic treatment [control of blood pressure, blood lipids, and blood glucose; improvement of cerebral circulation; and neuroprotection]). |  |
| Li Wei 2012 [3] | 48/46 | 68.29 ± 8.22/69.22 ± 7.88 | 54/40 | Beijing | 12 | Electroacupuncture–Standard of care (electroacupuncture + medication group: primary acupuncture points included Baihui and Shenting (electroacupuncture applied after “Deqi” at Baihui and Shenting), Qucha, Sishencong, and Fengchi; upper-limb points Neiguan and Hegu; lower-limb points Zusanli, Sanyinjiao, Taixi, and Zhaohai, with modifications based on syndrome differentiation. The needling directions were Baihui angled toward Xuanli, Shenting angled toward Baihui, and Qucha angled toward Chengguang + medication (nimodipine) + basic treatment [antiplatelet therapy, defibrination therapy, blood pressure control, regulation of blood glucose and blood lipids, and symptomatic management of comorbid conditions]). | Standard of care (medication group: nimodipine + basic treatment [antiplatelet therapy, defibrination therapy, blood pressure control, regulation of blood glucose and blood lipids, and symptomatic management of comorbid conditions]). |  |
| Wang Yiyan 2025 [4] | 30/30 | 63.20±5.96 / 62.93±6.01 | 38/22 | Heilongjiang | 8 | Scalp acupuncture–Standard of care (repetitive transcranial scalp acupuncture + basic treatment group: repetitive transcranial scalp acupuncture applied at Sishencong, Yintang, Taiyang (bilateral), Touwei (bilateral), Fengchi (bilateral), Waiguan (bilateral), Xuanzhong (bilateral), and the cognitive–affective area (frontal region). For the cognitive–affective area, the needle was inserted horizontally upward for 20 mm, followed by rapid twirling after achieving “Deqi,” with a rotational frequency of 200 r/min for 3 min; the remaining points were stimulated using routine acupuncture techniques. Each session involved 15 min of manipulation with a total needle-retention time of 30 min + basic treatment [blood pressure, blood glucose, and blood lipid control; antiplatelet therapy; and individualized functional rehabilitation training]). | Body acupuncture–Standard of care (non-manipulation routine acupuncture group: routine acupuncture applied at Sishencong, Yintang, Taiyang (bilateral), Touwei (bilateral), Fengchi (bilateral), Waigong (bilateral), Xuanzhong (bilateral), and the cognitive–affective area (frontal region), with the needle inserted horizontally upward for 20 mm in the cognitive–affective area, achieving “Deqi,” followed by a 30-min needle-retention period + basic treatment [blood pressure, blood glucose, and blood lipid control; antiplatelet therapy; and individualized functional rehabilitation training]). |  |
| Song Yuru 2025 [5] | 40/40 | 57.36±6.05 / 56.25±6.12 | 51/29 | Beijing | 6 | Scalp acupuncture–Cognitive Training–Standard of care (scalp acupuncture + Liuzijue Qigong + conventional Western medication group: scalp acupuncture applied to the vertex area (from Baihui to Qianding and the parallel lines 1 cun and 2 cun lateral to this line, covering parts of the precentral gyrus, postcentral gyrus, paracentral lobule, superior parietal lobule, and inferior parietal lobule); the pre-vertex area (from Baihui to Qianding and the parallel lines 1 cun and 2 cun lateral to this line, corresponding to the posterior portions of the superior and middle frontal gyri); and the frontal area (from Baihui to Shenting and the parallel lines 1 cun and 2 cun lateral to this line, corresponding to the anterior portion of the frontal lobe) + Liuzijue Qigong training: seated static practice of Liuzijue health Qigong, inhaling through the nose and exhaling through the mouth, softly chanting the six syllables “xū, hē, hū, sī, chuī, xī” during exhalation; during “xū,” the eyes are fully opened, while for the other syllables the eyes remain gently closed. Each syllable is practiced 6 times, progressing to the next only after completing the previous one, for a total of 36 repetitions per complete set of Liuzijue training + conventional Western therapy [blood glucose control, lipid lowering, blood pressure reduction, antiplatelet therapy, and secondary stroke prevention]). | Scalp acupuncture–Standard of care (scalp acupuncture + conventional Western medication group: scalp acupuncture applied to the vertex area (from Baihui to Qianding and the parallel lines 1 cun and 2 cun lateral to this line, corresponding to portions of the precentral gyrus, postcentral gyrus, paracentral lobule, superior parietal lobule, and inferior parietal lobule); the pre-vertex area (from Baihui to Qianding and the parallel lines 1 cun and 2 cun lateral to this line, corresponding to the posterior portions of the superior and middle frontal gyri); and the frontal area (from Baihui to Shenting and the parallel lines 1 cun and 2 cun lateral to this line, corresponding to the anterior portion of the frontal lobe) + conventional Western therapy [blood glucose control, lipid lowering, blood pressure reduction, antiplatelet therapy, and secondary stroke prevention]). |  |
| Li Leishen 2023 [6] | 30/30 | 65.63±3.82/65.58 ± 3.76 | 32 / 28 | Henan, Zhengzhou | 4 | Electroacupuncture–Standard of care (electroacupuncture + cognitive rehabilitation group: electroacupuncture applied at Baihui, Sishencong, Shenting, Benshen, Dazhui, Fengfu, Hegu, and Fenglong + cognitive rehabilitation training [memory training, orientation training, attention training, calculation training, language training, and game-based training]). | Standard of care (cognitive rehabilitation group: cognitive rehabilitation training [memory training, orientation training, attention training, calculation training, language training, and game-based training]). |  |
| Huang Saie 2014 [7] | 28/28 | 65±3 / 62±4 | 34/22 | Fujian | 12 | Body acupuncture–Standard of care (acupuncture + medication + rehabilitation training group: Governor Vessel acupuncture at Baihui and Shenting + medication (donepezil tablets) + rehabilitation training [cognitive function training and limb-function rehabilitation for motor impairment]). | Standard of care (medication + rehabilitation training group: medication (donepezil tablets) + rehabilitation training [cognitive function training and limb-function rehabilitation for motor impairment]). |  |
| Liu Jiao 2013 [8] | 25/25 | 53.40±8.5/53.40±8.5 | 34 / 16 | Henan, Zhengzhou | 4 | Electroacupuncture-Cognitive Training-Standard of care (electroacupuncture + cognitive function + basic treatment group: acupuncture at Shenting and Baihui + cognitive function training [attention, orientation, memory, calculation, and thinking] + basic treatment [blood pressure reduction, improvement of cerebral circulation, proper limb positioning, and motor-function rehabilitation training]). | Cognitive Training-Standard of care (cognitive function + basic treatment group: cognitive training (attention, orientation, memory, calculation, thinking) + basic treatment (blood pressure control, improving cerebral circulation, proper limb positioning, motor function rehabilitation)). |  |
| Liu Runli 2017 [9] | 32/32 | 56.9±10.3/56.4±10.1 | 41/23 | Shaanxi, Weinan | 2 | Electroacupuncture-Standard of care (electroacupuncture + conventional treatment group: electroacupuncture at Shenting and Baihui + conventional treatment [proper limb positioning, antihypertensive medication, and rehabilitation training]). | Standard of care (routine treatment group: proper limb positioning, antihypertensive medication, rehabilitation training). |  |
| Yang Mei 2022 [10] | 45/45 | 62.3±6.1 / 62.3±6.1 | 53/37 | Anhui, Tongling | 4 | Electroacupuncture-Cognitive Training-Standard of care (electroacupuncture + cognitive training + conventional treatment group: electroacupuncture at Baihui, Houding, Qianding, bilateral Benshen, and bilateral Neiguan, plus Shuigou and Shenting; for phlegm-damp obstruction type, Zusanli, Zhongwan, and Fenglong were added; for spleen-kidney deficiency type, Taixi, Sanyinjiao, and Zusanli were added; for blood stasis obstruction type, Siguans and Xuehai were added; for insufficiency of the Sea of Marrow, Xuanzhong and Dazhong were added + cognitive training [memory, orientation, attention, executive function, and calculation] + conventional treatment [blood pressure lowering, blood glucose reduction, lipid lowering, inhibition of platelet aggregation, and nimodipine tablets]). | Cognitive Training-Standard of care (cognitive training + routine treatment group: cognitive training (memory, orientation, attention, executive ability, calculation) + routine treatment (blood pressure control, glucose-lowering, lipid-lowering, antiplatelet aggregation, nimodipine tablets)). |  |
| Zhou Junying 2019 [11] | 60/60 | 61.44±8.77/62.04±8.69 | 74/46 | Henan, Puyang | 4 | Electroacupuncture-Cognitive Training-Standard of care (electroacupuncture + medication therapy + cognitive function training group: electroacupuncture at Baihui and Shenting + medication therapy [blood pressure control, blood glucose control, lipid regulation, anticoagulation, and improvement of circulation] + cognitive function training [memory, intelligence, language, observation, classification, and calculation abilities]). | Cognitive Training-Standard of care (medication + cognitive function training: medication (blood pressure and glucose control, lipid regulation, anticoagulation, improving circulation) + cognitive training (memory training, intelligence training, language training, observation, classification, and calculation training)). |  |
| Fang Zheng 2019 [12] | 41/41 | 65.18±4.29/ 65.07±4.31 | 44 / 38 | Beijing | 6 | Body acupuncture-Moxibustion-Standard of care (electroacupuncture + medication therapy + cognitive function training group: electroacupuncture at Baihui and Shenting + medication therapy [blood pressure control, blood glucose control, lipid regulation, anticoagulation, and improvement of circulation] + cognitive function training [memory, intelligence, language, observation, classification, and calculation abilities]). | Standard of care (cognitive training + routine treatment group: cognitive training (memory, orientation, attention, executive ability, calculation) + routine treatment (blood pressure control, glucose lowering, lipid lowering, antiplatelet aggregation, nimodipine tablets)). |  |
| Yang Mei 2017 [13] | 47/44 | 58.72±5.18/58.72±5.18 | 52 / 39 | Anhui, Tongling | 4 | Body acupuncture-Standard of care (acupuncture + cognitive function + conventional treatment group: acupuncture at Ezhong line, Baihui, Sishencong, bilateral Shenmen, bilateral Houxi, bilateral Zhaohai, and bilateral Xuanzhong + cognitive function training, correct limb positioning, transfer training, balance training, gait training, occupational therapy, and symptomatic medication). | Standard of care (cognitive function + routine treatment group: correct limb positioning, transfer training, balance training, gait training, occupational therapy, and symptomatic pharmacological treatment). |  |
| Wang Yanxu 2016 [14] | 30/30 | 69.83±4.50/ 71.05±3.21 | 32 / 28 | Fujian | 4 | Body acupuncture-Standard of care (acupuncture + Western medication + conventional treatment group: acupuncture at Baihui, Shenting, Sishencong, Hegu, Taichong, Sanyinjiao, Fenglong, Zusanli, and Xuehai + Western medication [nimodipine] + conventional treatment [antiplatelet therapy, plaque stabilization]). | Standard of care (Western medicine + routine treatment group: Western medicine (nimodipine) + routine treatment (antiplatelet aggregation, plaque stabilization)). |  |
| Bao Yu 2012 [15] | 30/30 | 63 ± 6 / 64 ± 6 | 40 / 20 | Heilongjiang | 8 | Body acupuncture-Standard of care (acupuncture + Western medication + conventional treatment group: acupuncture at Baihui, Sishencong, Fengchi, Shenmen, Sanyinjiao, Taixi, Xuanzhong + Western medication [donepezil hydrochloride] + conventional treatment [intracranial pressure reduction, fibrinolytic therapy, calcium channel antagonists, antiplatelet therapy, promoting blood circulation, maintaining electrolyte balance, nutritional support]). | Standard of care (Western medicine + routine treatment group: Western medicine (donepezil hydrochloride) + routine treatment (reducing intracranial pressure, fibrinolytic regulation, calcium channel blockers, antiplatelet aggregation, promoting blood circulation and removing stasis, maintaining water-electrolyte balance, nutritional support)). |  |
| Sun Yuanzheng 2011 [16] | 36/36 | 63.6 ± 5.8 / 64.1 ± 5.5 | 49 / 23 | Heilongjiang | 4 | Scalp acupuncture-Standard of care (cluster scalp acupuncture + Western medication + conventional treatment group: cluster scalp acupuncture with 5 needles inserted in frontal and parietal zones to cover lesion areas; frontal zone from Shenting to Xinhui with 1–2 cun lateral parallel lines; parietal zone from Qianding to Baihui with 1–2 cun lateral lines + Western medication [Aricept] + conventional treatment [promoting circulation, antiplatelet therapy, fibrinolytic therapy, calcium channel antagonists, intracranial pressure reduction, electrolyte balance maintenance]). | Standard of care (Western medicine + routine treatment group: Western medicine (Aricept) + routine treatment (promoting blood circulation and removing stasis, antiplatelet aggregation, fibrinolytic regulation, calcium channel blockers, reducing intracranial pressure, maintaining water-electrolyte balance)). |  |
| Xiang Rong 2019 [17] | 40/40 | 62.2 ± 4.6 / 61.9 ± 4.6 | 47 / 33 | Hebei, Cangzhou | 8 | Scalp acupuncture-Body acupuncture-Standard of care (scalp acupuncture + cervical acupuncture + body acupuncture + conventional rehabilitation group: scalp acupuncture at the Dingqian, frontal area, Sishencong, and Baihui; cervical acupuncture at Fengfu, Fengchi, Yiming, and blood-supply points; body acupuncture at Taichong, Guanyuan, and Shenshu + conventional rehabilitation [routine cognitive training and medication with nimodipine]). | Standard of care (routine rehabilitation group: routine cognitive function training with nimodipine medication). |  |
| Wei Bingxin 2023 [18] | 40/40 | 53.18 ± 8.42 / 52.31 ± 7.87 | 52 / 28 | Guangxi, Liuzhou | 4 | Scalp acupuncture-Cognitive Training-Standard of care (motor scalp acupuncture + cognitive function training group: motor scalp acupuncture on Yu’s seven zones—parietal (Baihui to Qianding and bilateral Sishencong), pre-parietal (Qianding through Xinhui, Tongtian through Chengguang, Zhengying through Muchuang) + cognitive training). | Cognitive Training-Standard of care (cognitive function training + routine treatment group: cognitive function training + routine treatment (secondary prevention of cerebral infarction, antiplatelet therapy, lipid-lowering and plaque stabilization, blood pressure and glucose control)). |  |
| Yan Hongda 2016 [19] | 30/30 | 65 / 66 | 35 / 25 | Guangdong, Zhongshan | 8 | Moxibustion-Cognitive Training-Standard of care (Leihuo moxibustion + cognitive training + basic treatment group: Leihuo moxibustion at Dazhui and Shenshu using warming–tonifying method + cognitive training [memory, calculation, intelligence] + basic treatment [antiplatelet therapy; blood pressure and glucose management]). | Cognitive Training-Standard of care (cognitive training + basic treatment group: cognitive training (memory training, calculation training, intelligence training) + basic treatment (antiplatelet therapy; blood pressure and glucose management)). |  |
| Zhang Xiaoying 2018 [20] | 38/38 | 69.5 ± 5.5 / 68.9 ± 4.9 | 40 / 26 | Shaanxi, Xi'an | 8 | Scalp acupuncture-Standard of care (Fangshi scalp acupuncture + idebenone + conventional symptomatic treatment group: Fangshi scalp acupuncture at writing area, thinking area, movement–balance area, balance area, and Fuxiang head area + idebenone + symptomatic therapy [antiplatelet, microcirculation improvement, oxygen supplementation, infection control, intracranial pressure reduction, antioxidant therapy]). | Standard of care (idebenone + routine symptomatic treatment group: idebenone + routine symptomatic treatment (antiplatelet therapy, improving microcirculation, oxygen therapy, infection control, reducing intracranial pressure, scavenging oxygen free radicals)). |  |
| Li Lanchen 2019 [21] | 34/34 | 63.17 ± 7.84 / 62.61 ± 7.76 | 39 / 29 | Heilongjiang | 12 | Scalp acupuncture-Standard of care (scalp acupuncture + medication group: scalp acupuncture at Baihui, Shenting, Sishencong, Benshen; with additional points Fengfu, Fengchi, Neiguan, Shenmen, Touwei, Hegu, Quchi, Zusanli, Wangushan, Sanyinjiao, Taichong, Taixi + medication [nimodipine]). | Standard of care (medication group: nimodipine). |  |
| Cao Hui 2019 [22] | 51/49 | 60 ± 3 / 59 ± 3 | 51 / 49 | Nantong | 6 | B-Standard of care (Jing-well bloodletting + conventional treatment group: Jing-well bloodletting at Shaoshang, Shangyang, Zhongchong, Guanchong, Shaochong + conventional treatment [intracranial pressure reduction, thrombolysis, microcirculation improvement, vasodilation, anti-inflammatory therapy, rehabilitation training—including joint ROM, sitting/standing balance, transfer training, ADL training]). | Standard of care (routine treatment: reducing intracranial pressure, thrombolysis, improving microcirculation, vasodilation, anti-inflammatory therapy, and rehabilitation—range of motion training, sitting/standing balance training, transfer training, activities of daily living training). |  |
| Wang Qin 2019 [23] | 59/59 | 68.88 ± 3.64 / 67.71 ± 3.02 | 68 / 50 | Liupanshui | 4 | Body acupuncture-Standard of care (acupuncture + Western medication + conventional treatment group: acupuncture at Baihui, Fengchi, Shenting, Fengfu, Dazhui, cervical Jiaji, Quchi, Neiguan, Fengshi, Zusanli, Yanglingquan, Sanyinjiao, Xuehai; with syndrome-based additions—Ganshu, Shenshu, Pishu, Geshu, Qihai, Fenglong, Zhongwan + Western medication [atorvastatin] + conventional treatment [intracranial pressure reduction, antiplatelet therapy, electrolyte balance maintenance]). | Standard of care (Western medicine + routine treatment group: Western medicine (atorvastatin) + routine treatment (reducing intracranial pressure, antiplatelet therapy, maintaining water–electrolyte balance)). |  |
| Gao Yan 2019 [24] | 20/20 | 67.93 ± 4.26 / 68.73 ± 3.24 | 17 / 23 | Shandong, Weihai | 12 | EarAcu-Cognitive Training-Standard of care (auricular acupressure + cognitive rehabilitation + conventional treatment group: auricular acupressure at heart, brain, kidney, subcortex, ear center, brainstem, Shenmen, sympathetic points + cognitive rehabilitation [memory, analytic judgment, language, calculation, sensory integration] + conventional treatment [BP and glucose control, lipid stabilization, routine nursing with psychological support]). | Cognitive Training-Standard of care (cognitive rehabilitation training + routine treatment group: cognitive rehabilitation training (memory training, analytical reasoning, language training, calculation ability, sensory integration) + routine treatment (basic management including blood pressure and glucose control, plaque stabilization, and routine nursing combined with psychological intervention)). |  |
| Deng Ru 2021 [25] | 40/40 | 59 ± 3 / 60 ± 3 | 41 / 39 | Shaanxi | 8 | Scalp acupuncture-BodyAcu Bleeding-Standard of care (scalp acupuncture + Jing-well bloodletting + Western medication + conventional treatment group: scalp acupuncture at Dingzhong line, ipsilateral parietotemporal anterior oblique line, ipsilateral parietotemporal posterior oblique line; Jing-well bloodletting at Shaoshang, Shangyang, Zhongchong, Guanchong, Shaochong + Western medication [nimodipine] + conventional treatment [anticoagulation, BP/glucose/lipid control, nutrition support, prevention of complications]). | Standard of care (Western medicine + routine treatment group: Western medicine (nimodipine) + routine treatment (anticoagulation, blood pressure control, glucose and lipid regulation, nutritional support, prevention and treatment of complications)). |  |
| Niu Lei 2021 [26] | 75/75 | 52.06 ± 7.98 / 51.89 ± 10.24 | 81 / 69 | Zhengzhou | 6 | Scalp acupuncture-EarAcu-Cognitive Training-Standard of care (cluster scalp acupuncture with long-retention + cognitive function training + basic treatment group: cluster scalp acupuncture (frontal, pre-parietal, and parietal regions; 5 needles per region; 6-hour retention) + cognitive training [memory, attention, calculation, executive function, agnosia/apraxia training] + basic treatment [BP/glucose/lipid control, antiplatelet therapy, supportive treatment]). | Cognitive Training-Standard of care (cognitive function training + basic treatment group: cognitive function training (memory, attention, calculation, executive function, agnosia/apraxia training) + basic treatment (antihypertensive, hypoglycemic, lipid-lowering, antiplatelet therapy and symptomatic support)). |  |
| Wang Zhenyao 2019 [27] | 104/104 | 62 ± 7 / 62 ± 7 | 123/85 | Beijing | 4 | Body acupuncture-Standard of care (acupuncture + cognitive rehabilitation group: acupuncture at Baihui, Shenting, Sishencong, Neiguan, Waiguan, Fengchi, Fengshi, Quchi, Zusanli, Xuehai, Yanglingquan + cognitive rehabilitation [attention, calculation, memory, orientation, executive function]). | Standard of care (cognitive rehabilitation training group: rehabilitation training including attention, calculation, memory, orientation, and executive function). |  |
| Yuan Hongwei 2022 [28] | 39/40 | 61 ± 8 / 59 ± 9 | 60/19 | Beijing | 4 | Electroacupuncture-Standard of care (electroacupuncture at two Du meridian points + basic treatment group: acupuncture at Baihui, Shenting (connected to electroacupuncture), Sishencong, Benshen; with insomnia adding Neiguan and Shenmen + moxibustion at Shenting, Baihui, Shendao, Fengfu, Xinshu + basic treatment [attention, memory, orientation, calculation, and unilateral neglect training]). | Standard of care（Cognitive training group: attention training, memory training, orientation training, calculation training, unilateral neglect training） |  |
| Jian Xiong 2020 [29] | 35/35 | 63.0 ± 7.23 / 65.3 ± 8.52 | 37/33 | Wuhan | 12 | Scalp acupuncture-Cognitive Training-Standard of care (scalp acupuncture + cognitive training + standard rehabilitation group: scalp acupuncture at Baihui, Sishencong, Fengchi, and Shenting + cognitive training using cards, objects, and computer tasks + standard rehabilitation [physical therapy, occupational therapy, cognitive training]). | Cognitive Training-Standard of care（Sham scalp acupuncture + cognitive training + standard rehabilitation group: sham points at Baihui–Shenting midpoint + cognitive training [cards, objects, computer-based tasks] + standard rehabilitation [physical therapy, occupational therapy, cognitive therapy]） |  |
| Wang Huiling 2017 [30] | 30/30 | 53.27 ± 11.62 / 56.73 ± 9.32 | 39/21 | Henan | 8 | Electroacupuncture-Cognitive Training-Standard of care (electroacupuncture + cognitive function training + conventional treatment group: electroacupuncture at Baihui and Zusanli + cognitive training [attention, memory, orientation, judgment, visuospatial ability] + conventional treatment [BP stabilization, improved cerebral circulation, neurotrophic therapy, plus PT/OT/physical therapy]). | Cognitive Training-Standard of care（Cognitive training + routine treatment group: cognitive training [attention, memory, orientation, judgment, and visuospatial ability training] + routine treatment [basic treatment: blood pressure stabilization, cerebral circulation improvement, neurotrophic support; rehabilitation: exercise therapy, occupational therapy, physical therapy]） |  |
| Sun Shanbin 2017 [31] | 30/30 | 65.07 ± 7.06 / 64.07 ± 6.58 | 30/30 | Anhui | 8 | Electroacupuncture-Standard of care (electroacupuncture + conventional treatment group: electroacupuncture at Shenting and Sishencong + syndrome-based point selection [Shenting 1 group, Sishencong 2 groups] + conventional treatment [basic rehabilitation training]). | Standard of care（Medication + routine treatment group: medication [oxiracetam tablets] + routine treatment [basic rehabilitation training]） |  |
| Wei Ruipeng 2019 [32] | 30/30 | 60.32 ± 7.93 / 60.38 ± 8.01 | 37/23 | Guangdong, Shenzhen | 6 | Electroacupuncture-Standard of care (Electroacupuncture combined with computer-assisted cognitive training and routine medication group: electroacupuncture at the Midline of Forehead, Midline of Vertex, and bilateral postero-temporal oblique lines; computer-assisted cognitive training including structured organizational ability training, orientation training, attention training, language ability training, memory ability training, and calculation ability training; plus routine medication.) | Standard of care（Computer-assisted cognitive training + routine medication group: computer-assisted cognitive training [structural organization training, orientation training, attention training, language training, memory training, calculation training] + routine medication） |  |
| Wu Chao 2019 [33] | 40/40 | - 64.05 ± 10.41 /   62.98 ± 11.41 | 26/54 | Nanjing | 4 | Electroacupuncture-Standard of care (Electroacupuncture plus basic treatment group: electroacupuncture at Baihui (GV20), Sishencong (EX-HN1), and related points; basic treatment including nimodipine administration, routine antiplatelet therapy, plaque stabilization, and free radical scavenging for cerebral infarction, or dehydration therapy and blood pressure control for cerebral hemorrhage.) | Standard of care（Basic treatment: all patients received nimodipine; ischemic stroke patients additionally received routine antiplatelet therapy, plaque-stabilizing treatment, and free radical scavenging; hemorrhagic stroke patients received dehydration therapy and blood pressure control） |  |
| Saren 2019 [34] | 55/55 | 67.96±10.12/68.02±9.57 | 65/45 | Hainan | 6 | Scalp acupuncture-Standard of care (Scalp cluster-needling plus routine treatment group: scalp cluster-needling at Baihui, Shenting, Sishencong, Benshen, Fengfu, Fengchi, Touwei, Yintang, Renzhong, Neiguan, Zusanli, Sanyinjiao, and Taichong; routine treatment for improving cerebral circulation, antiplatelet aggregation, lipid regulation, promoting cerebral metabolism, and blood pressure control.) | Body acupuncture-Standard of care（Routine acupuncture + routine treatment group: routine acupuncture [Waiguan, Quchi, Zusanli, Shousanli, Sanyinjiao, Hegu, Taixi, Taichong] + routine treatment [cerebral circulation improvement, antiplatelet aggregation, lipid regulation, promotion of cerebral metabolism, blood pressure reduction]） |  |
| Li Ziting 2023 [35] | 34/34 | 69±8/67±9 | 43/25 | Beijing | 4 | Scalp acupuncture-Standard of care (Ring-shaped cranial base acupuncture group: modified cranial-base acupoint needling at bilateral Fengchi, Wangu, Tianzhu, Yamen, and Baihui.) | Body acupuncture-Standard of care（Distal non-channel/non-acupoint group: shallow needling at 8 distal non-channel/non-acupoint locations [bilateral insertion point 1 (midpoint of olecranon–axilla line), point 2 (midpoint of humeral medial epicondyle–ulnar wrist line), point 3 (junction of deltoid and biceps at medial anterior upper arm), point 4 (2 cm lateral to Zusanli level)]） |  |
| Xin Mu 2024 [36] | 67/68 | 70.81±3.00/70.71±2.70 | 74/61 | Sichuan | 4 | Body acupuncture-Standard of care (Acupuncture combined with modern rehabilitation therapy group: Xingnao Kaiqiao acupuncture at Neiguan, Renzhong, Baihui, Sishencong, Fengchi, Hegu, Sanyinjiao, and Taichong; modern rehabilitation including routine therapy such as limb positioning, passive and active limb exercises, balance training, ADL rehabilitation, and cognitive training including writing, attention and hand–eye coordination, memory, calculation, and analytical ability training.) | Standard of care（Modern rehabilitation therapy group: routine rehabilitation training [limb positioning, passive and active bed exercises, balance training, activities of daily living training] + cognitive rehabilitation training [writing training, attention and hand–eye coordination training, memory training, calculation training, comprehensive analytical ability training]） |  |
| Zeng Youhua 2015 [37] | 50/50 | 66±12/68±10 | 62/38 | Hangzhou | 8 | Electroacupuncture-Standard of care (Electroacupuncture plus basic treatment group: electroacupuncture at Baihui, Sishencong, Shenting, Yintang, Hegu, and Taichong; routine care including airway clearance, secretion removal, prevention and management of aspiration pneumonia, monitoring and treatment of arrhythmias and ischemic heart disease, blood pressure and glucose regulation, appropriate use of anticoagulants/antiplatelet agents, routine acupuncture for limb recovery, and cognitive training including picture recall, word-pair association, number games, block puzzles, and diary training.) | Standard of care（Basic treatment group: routine airway maintenance, removal of respiratory secretions, prevention and management of aspiration pneumonia; monitoring and treatment of arrhythmias and ischemic heart disease; blood pressure and glucose regulation; appropriate use of anticoagulants and antiplatelet agents; routine acupuncture for limb function improvement; cognitive rehabilitation training [picture recall, word association matching, number games, block puzzles, diary training]） |  |
| Luo Jianchang 2019 [38] | 94/94 | 66.24 ± 9.12 / 65.87 ± 9.01 | 112/76 | Taizhou | 12 | Electroacupuncture-Standard of care (Electroacupuncture at the Eight Cervical Points plus routine treatment group: Eight Cervical Points located at midpoints between bilateral Fengchi and Fengfu at upper and lower levels; plus bilateral Fengchi, Fengfu, and Dazhui; routine treatment including blood pressure and glucose control, antiplatelet therapy, and plaque stabilization.) | Standard of care（Medication + routine treatment group: medication [nimodipine tablets] + routine treatment [blood pressure control, glucose control, antiplatelet aggregation, plaque stabilization]） |  |
| Zhang Qufei 2018 [39] | 40/40 | 71.00 ± 2.74 / 71.47 ± 2.83 | 39/41 | Guangdong | 8 | Scalp acupuncture-Standard of care (Baihui Bagua scalp acupuncture plus routine treatment group: Baihui Bagua scalp acupuncture is performed with Baihui as the center, selecting points in the anterior, posterior, left, right, upper-left, lower-left, upper-right, and lower-right directions. Points located 1 cun lateral to Baihui form the small Bagua, 2 cun lateral form the medium Bagua, and 3 cun lateral form the large Bagua. All three Bagua groups are alternately stimulated with penetrating needling toward Baihui (0.8–1.2 cun) and connected to electroacupuncture; routine treatment includes improving cerebral circulation, nourishing brain neurons, antiplatelet therapy, lipid stabilization, blood pressure control, blood glucose control, and supportive symptomatic treatment.) | Standard of care（Western medicine + routine treatment group: Western medicine [nimodipine tablets] + routine treatment [improving cerebral circulation, neurotrophic support, antiplatelet therapy, lipid regulation and plaque stabilization, blood pressure control, glucose control, symptomatic support]） |  |
| Ding Xiao 2016 [40] | 40/46 | 56.24 ± 8.12 / 57.87 ± 9.01 | 48/38 | Beijing | 8 | Scalp acupuncture-Standard of care (Scalp acupuncture plus rehabilitation group: scalp acupuncture applied before rehabilitation and needles removed afterward, including Baihui, Sishencong, the lesion-side anterior temporal oblique line (from Qianding to Xuanli), the lesion-side posterior temporal oblique line (from Baihui to Qubin), the lesion-side anterior temporal line (from Xuanxian to Xuanli), and the lesion-side posterior temporal line (from Shuaigu to Qubin); rehabilitation treatment includes antiplatelet therapy, control of blood pressure, blood glucose, and lipids, and rehabilitation modalities such as physical therapy and occupational therapy.) | Standard of care（Rehabilitation treatment group: antiplatelet therapy, control of blood pressure/glucose/lipids, and rehabilitation treatment [exercise therapy, occupational therapy]） |  |
| Tong Xin 2012 [41] | 34/34 | 58.4 ± 8.5 / 59.2 ± 8.9 | 45/23 | Heilongjiang | 4 | Body acupuncture-Standard of care (Acupuncture plus cognitive training and routine treatment group: acupuncture at Baihui, Sishencong, Neiguan, Shuigou, Tanzhong; adjunct points including Sanyinjiao, Jiquan, Fengchi, Quchi, Hegu, Chize, Zusanli, Taichong, Gongsun, Zhaohai, Huantiao; Baihui to Qubin, Neiguan to Waiguan, Shuigou to Yingxiang, Qianshencong to Xuanli, Tanzhong to Zhongting; all adjunct points selected on the affected side. Cognitive training includes thinking exercises, attention and hand–eye coordination training, memory training, and daily activity memory tasks; routine treatment includes improving cerebral circulation, reducing cerebral edema, protecting neurons, regulating blood pressure, and nutritional support including tube feeding and IV nutrition.) | Standard of care（Cognitive training + routine treatment group: cognitive training [thinking training, attention and hand–eye coordination training, memory training, daily living memory training] + routine treatment [improving cerebral circulation, controlling cerebral edema, protecting neurons, blood pressure regulation, nasogastric feeding guidance, intravenous nutritional support]） |  |
| Chen Shaofei 2019 [42] | 40/40 | 63.23 ± 7.15 / 62.70 ± 7.18 | 36/44 | Anhui, Hefei | 4 | Scalp acupuncture-EarAcu-Standard of care (Scalp embedded needles plus auricular seeds and medication group: embedded needles using clip-type intradermal needles placed at Baihui, Sishencong, and Zhisan points with 5-day retention; auricular seeds applied to Heart, Brain point, Liver, Kidney, Shenmen, Adrenal, and Occiput areas, alternating sides every other day; medication includes nimodipine tablets and piracetam tablets.) | Standard of care（Medication group: nimodipine tablets + piracetam tablets） |  |
| Tian Ran 2021 [43] | 30/30 | 58.13 ± 5.12 / 60.14 ± 6.33 | 31/29 | Jiangsu | 4 | Scalp acupuncture-NIBS-Standard of care (Scalp acupuncture plus hyperbaric oxygen and routine rehabilitation group: scalp acupuncture at Baihui, Sishencong, the midline of vertex, midline of forehead, anterior temporal line, and posterior temporal line; hyperbaric oxygen therapy at 0.2 MPa with 20 minutes of pressurization and two oxygen inhalation cycles per session; routine rehabilitation including attention, memory, orientation, executive function, calculation, visuospatial training, training for apraxia and agnosia, handwriting training, language and communication exercises, and lifestyle guidance.) | NIBS-Standard of care（Hyperbaric oxygen + routine rehabilitation group: hyperbaric oxygen therapy [0.2 MPa chamber pressure, 20 min compression time, stable pressure followed by two oxygen inhalation cycles] + routine rehabilitation [attention, memory, orientation, executive function, calculation, visuospatial ability, apraxia/agnosia training, writing training, language and communication, lifestyle guidance]） |  |
| Yisha Guo 2025 [44] | 17/17 | 54.71 ± 9.34 / 49.00 ± 13.44 | 25/9 | Shanghai | 12 | Electroacupuncture-Standard of care (Electroacupuncture plus cognitive rehabilitation group: electroacupuncture at Baihui and Shenting; cognitive rehabilitation including attention and hand–eye coordination training, calculation training, writing training, memory training, thinking-function training, orientation training, and language training.) | Standard of care（Cognitive rehabilitation group: attention and hand–eye coordination training, calculation training, writing training, memory training, thinking function training, orientation training, and language training） |  |
| Zhang Boyang 2024 [45] | 34/33 | 无 | 无 | Heilongjiang | 3 | Electroacupuncture-Standard of care (Electroacupuncture EA group: electroacupuncture at Sishencong combined with routine rehabilitation including physical therapy.) | Standard of care（Sham SA group: sham acupuncture [non-channel, non-acupoint locations at the midpoint between bilateral Jianyu (LI15) and Binao (LI14), and the midpoint between Jianyu (LI15) and Jianzheng (SI9), with flat-head needles fixed on sham points to simulate electrical current] + routine rehabilitation (physical therapy)） |  |
| Li Huang 2021 [46] | 60/60 | 65.1 ± 7.5 / 64.6 ± 8.4 | 62/58 | Shanghai | 16 | Electroacupuncture (EA group: electroacupuncture at Baihui (GV20), Shenting (GV24), Sishencong (EX-HN1), Shuigou (GV26), Yintang (GV29), Naohu (GV17), Fengchi (GB20), Shenmen (HT7), and Sanyinjiao (SP6)). | Standard of care（SA group: sham acupuncture） |  |
| Zhan Jie 2016 [47] | 25/25 | 60 ± 10 / 60 ± 9 | 33/17 | Guangdong | 4 | Body acupuncture-Standard of care (acupuncture + basic treatment + routine rehabilitation group: acupuncture (Baihui, Shenting) + basic treatment (Chinese medicine, Western medicine, and routine acupuncture; Chinese medicine: Jieyu Decoction, Buyang Huanwu Decoction, Zuogui Pill combined with Dihuang Yin Zi; Western medicine: blood pressure control, glucose control, lipid regulation and plaque stabilization, circulation improvement, antiplatelet aggregation; routine acupuncture: Jiquan, Chize, Neiguan, Hegu, Weizhong, Zusanli, Sanyinjiao, Taichong) + routine rehabilitation training (cognitive training, physical therapy, occupational therapy)). | Standard of care（Basic treatment + routine rehabilitation group: basic treatment [Chinese medicine, Western medicine, and routine acupuncture; Chinese medicine (Jieyu Dan, Buyang Huanwu Decoction, Zuogui Pill combined with Dihuang Decoction); Western medicine (blood pressure and glucose control, lipid regulation and plaque stabilization, circulation improvement, antiplatelet aggregation); routine acupuncture (Jiquan, Chize, Neiguan, Hegu, Weizhong, Zusanli, Sanyinjiao, Taichong)] + routine rehabilitation training (cognitive training, physical therapy, occupational therapy)） |  |
| Lifang Chen 2016 [48] | 78/71 | 62.52 ± 10.60 / 64.06 ± 10.54 | 77/72 | Zhejiang, Hangzhou | 3 | Electroacupuncture-Standard of care (acupuncture AG [scalp acupuncture + electroacupuncture] + routine rehabilitation nursing group: acupuncture sequence = scalp acupuncture 4 h → cervical acupuncture (for dysphagia) → body acupuncture → electroacupuncture; needle removal: electroacupuncture → body acupuncture → cervical acupuncture → scalp acupuncture; 2–3 needles inserted through the upper and middle lines, motor area (MS-6), and sensory area (MS-7) on the affected side; whole-body acupuncture on the affected side (Jianyu, Quchi, Shousanli, Waiguan, Hegu; Liangqiu, Zusanli, Yanglingquan, Sanyinjiao, Fenglong, Jiexi, Taichong); for dysphagia add (Fengchi, Yiming, Tianzhu, Fengfu, Gongxue, Lianquan); for cognitive impairment add (Baihui, Shenting, Benshen, Sishencong) + routine rehabilitation nursing (normal limb positioning, passive hemiplegic-side movement, bedside rehabilitation (Bobath technique, rolling, bridging), neuromuscular electrical stimulation, swallowing training for dysphagia, or cognitive training for cognitive impairment)). | Standard of care（Non-acupuncture group NAG: routine rehabilitation nursing (normal limb positioning, passive movement of the hemiplegic side, bedside rehabilitation [Bobath technique, rolling training, bridging], neuromuscular electrical stimulation, swallowing training for dysphagia, or cognitive training for cognitive impairment)） |  |
| Zhang Shasha 2022 [49] | 46/46 | 61.83 ± 6.29 / 61.26 ± 5.29 | 54/38 | Shanghai | 4 | Body acupuncture-NIBS-Standard of care (acupuncture + tDCS + routine rehabilitation group: acupuncture (Baihui, Shuigou, Sishencong, Taixi, Fenglong; Fenglong with strong reducing technique, Sanyinjiao with tonifying technique, Zusanli with meridian-following insertion) + transcranial direct current stimulation (tDCS): anode over frontal area, reference electrode over healthy-side shoulder + routine rehabilitation (passive joint movement, neurodevelopmental therapy, sit-to-stand training, bed-to-chair transfer, EMG biofeedback, ADL training, pneumatic therapy, hyperbaric oxygen; 40 min/session, physiotherapy 20 min/session)). | Standard of care（Sham tDCS + routine rehabilitation group: sham tDCS applied using the same method as real stimulation + routine rehabilitation (passive joint movement training, neurodevelopmental therapy, sit-to-stand training, bed-to-chair transfer, electromyographic biofeedback, activities of daily living training, air-pump therapy, and hyperbaric oxygen; 40 min/session for rehabilitation, 20 min/session for physical therapy)） |  |
| Chen Qiusheng 2020 [50] | 40/40 | 56.85 ± 17.12 / 56.95 ± 18.09 | 43/37 | Guangzhou | 4 | Scalp acupuncture-NIBS-Standard of care (scalp acupuncture + repetitive transcranial magnetic stimulation + basic treatment group: scalp acupuncture (contralateral anterior/posterior parietotemporal oblique lines, frontal midline; ipsilateral temporal anterior/posterior lines; Baihui, Sishencong, Yamen, Tongtian, Fengfu, Fengchi) + rTMS (dorsolateral prefrontal cortex) + basic treatment (routine medication: secondary stroke prevention, neurotrophic support, circulation improvement, cognitive enhancement; routine rehabilitation: attention, memory, thinking training)). | NIBS-Standard of care（Repetitive transcranial magnetic stimulation + basic treatment group: rTMS (dorsolateral prefrontal cortex) + basic treatment [routine medications (secondary stroke prevention, neurotrophic support, circulation improvement, cognitive enhancement) and routine rehabilitation training (attention, memory, thinking)]） |  |
| Li Jianhong 2019 [51] | 31/31 | 62.14 ± 8.22 / 57.22 ± 8.26 | 39/23 | Ningxia | 8 | Body acupuncture-Cognitive Training-Standard of care (acupuncture + computer-assisted cognitive training + Oxiracetam group: acupuncture (Sishencong, Baihui, Shenting) + computerized cognitive rehabilitation (memory, attention, executive function, visual training, calculation training) + Oxiracetam capsules). | Cognitive Training-Standard of care（Computer-assisted cognitive training + oxiracetam group: computerized cognitive rehabilitation system (memory training, attention training, executive function training, visual training, calculation training) + oxiracetam capsules） |  |
| Yao Juanjuan 2019 [52] | 40/38 | 61.27 ± 5.38 / 62.72 ± 6.48 | 41/37 | Fujian | 12 | Electroacupuncture-Standard of care (electroacupuncture + cognitive rehabilitation + routine treatment group: electroacupuncture (Shenting, Baihui, bilateral Benshen) + cognitive rehabilitation (attention, perception, memory, orientation, executive function) + routine treatment (blood pressure, glucose, lipid control; antiplatelet and anticoagulation therapy after stroke)). | Standard of care（Cognitive rehabilitation training + routine treatment group: cognitive rehabilitation training [attention impairment, perceptual impairment, memory impairment, orientation impairment, thinking–action impairment] + routine treatment (blood pressure, glucose, and lipid control, post-stroke antiplatelet and anticoagulation therapy)） |  |
| Liu Xujia 2023 [53] | 31/29 | 54.45 ± 10.58/57.55 ± 11.66 | 38/22 | Shenyang | 3 | Scalp acupuncture-NIBS-Standard of care (scalp acupuncture + repetitive transcranial magnetic stimulation (rTMS) + basic treatment group: scalp acupuncture (Baihui, Sishencong, Shenting, Touwei, Yuzhen, Fengchi) + rTMS (left dorsolateral prefrontal cortex) + basic treatment (pharmacological therapy and routine rehabilitation, including antiplatelet therapy, plaque stabilization, blood pressure and glucose control for secondary stroke prevention, symptomatic management of complications, physical therapy, occupational therapy, and cognitive impairment training)). | Scalp acupuncture-Standard of care（Scalp acupuncture + basic treatment group: scalp acupuncture [Baihui, Sishencong, Shenting, Touwei, Yuzhen, Fengchi] + basic treatment (pharmacotherapy and routine rehabilitation, antiplatelet therapy, plaque stabilization, blood pressure and glucose control as part of secondary stroke prevention, management of complications, physical therapy, occupational therapy, and cognitive dysfunction training)） |  |
| Chen Honglin 2023 [54] | 55/55 | 48.20±4.74/47.11±5.61 | 66/44 | Heilongjiang | 4 | Body acupuncture-Cognitive Training-Standard of care (acupuncture + cognitive rehabilitation + routine treatment group: acupuncture (Taixi, Zusanli, Shenmen, Fengfu, etc.) + cognitive rehabilitation (attention training, calculation training, apraxia training, memory training) + routine treatment (oral aspirin, folic acid, nimodipine, statins) + routine treatment + cognitive rehabilitation). | Cognitive Training-Standard of care（Cognitive rehabilitation training + routine treatment group: cognitive rehabilitation (attention training, calculation training, apraxia training, memory training) + routine treatment (oral aspirin, folic acid, nimodipine, and statins)） |  |
| Li Lei 2019 [55] | 40/40 | 66.9±5.9/67.4±6.1 | 40/40 | Hubei, Wuhan | 6 | Body acupuncture-Standard of care (acupuncture + Western medicine + routine treatment group: acupuncture (Shenting, Baihui, Sishencong, Qihai, Guanyuan, Hegu, Zusanli, Taichong) + Western medicine (Donepezil hydrochloride) + routine treatment (respiratory and urinary tract management, blood pressure/glucose/lipid control, prevention of complications such as hypostatic pneumonia and pressure ulcers, rehabilitation therapy, cerebral edema management for hemorrhagic stroke, vasospasm prevention, antiplatelet or anticoagulation therapy for ischemic stroke, circulation improvement, neurotrophic therapy)). | Standard of care（Western medicine + routine pharmacological treatment group: Western medicine (donepezil hydrochloride) + routine pharmacotherapy (airway and urinary tract management, blood pressure/glucose/lipid control, prevention of complications such as aspiration pneumonia and pressure ulcers, rehabilitation therapy, edema control for hemorrhagic stroke, vasospasm prevention, antiplatelet or anticoagulation therapy for ischemic stroke, circulation improvement, and neurotrophic support)） |  |
| Lida Zhong 2025 [56] | 18/18/18 | 59.89 ± 2.25 / 60.61 ± 2.89 / 65.11 ± 2.03 | 36/28 | Guangdong | 4 | Scalp acupuncture-NIBS-Standard of care/Scalp acupuncture-Standard of care (combined SA + iTBS + routine rehabilitation group: SA scalp acupuncture (scalp needling of MS1, MS2, MS3, MS5, MS10, MS11) + iTBS (intermittent theta-burst stimulation) + routine rehabilitation (standard pharmacologic therapy and routine rehabilitation training)). | NIBS-Standard of care（SA + routine rehabilitation group / iTBS + routine rehabilitation group: SA scalp acupuncture group [scalp lines MS1, MS2, MS3, MS5, MS10, MS11] + routine rehabilitation (standard pharmacological and routine rehabilitation treatments) / iTBS group (intermittent theta-burst stimulation) + routine rehabilitation (standard pharmacological and routine rehabilitation treatments)） |  |
| Zhang Chunxia 2021 [57] | 200/200 | 59 ± 15 / 63 ± 14 | 285/115 | Shenzhen | 8 | Scalp acupuncture-Standard of care (interactive scalp acupuncture + cognitive training + routine treatment group: scalp acupuncture (vertex midline, contralateral anterior parietotemporal oblique line, parietotemporal posterior oblique line; 200 rapid manipulations per minute) + cognitive training (response training, plane-recognition training, visual memory training, logical reasoning training, calculation training, eye-movement training, search ability training, attention training) + routine medication (aspirin, atorvastatin calcium tablets) and routine motor rehabilitation (proper limb positioning, joint mobility training, transfer training, balance training, gait training)). | Scalp acupuncture-Cognitive Training-Standard of care（Scalp acupuncture alone + routine treatment group: scalp acupuncture on the midline, contralateral anterior oblique line of vertex–temporal region, and posterior oblique line of vertex–temporal region with 200 rapid rotations/min / Scalp acupuncture + cognitive training + routine treatment group: the same scalp needling + reaction behavior training, planar recognition training, graphic memory training, logical reasoning training, calculation training, oculomotor training, search ability training, attention training + routine medication (aspirin, atorvastatin calcium) and routine motor rehabilitation (proper limb positioning, ROM training, transfer training, balance training, gait training)） |  |
| Shuhua Wang 2016 [58] | 40/39/40 | **65.2 ± 7.1/60.6 ± 6.7/64.4 ± 7.7** | 82/37 | Tianjin | 24 | Body acupuncture-Standard of care/Standard of care (nimodipine + acupuncture group: nimodipine + acupuncture (Baihui, Sishencong, Sibai, Fengchi, Wanggu, Tianzhu, Renzhong, Shenmen, Neiguan, Fenglong, Sanyinjiao, Taichong); horizontal insertion 1 cun at Baihui and Sishencong, oblique insertion at Sibai to 0.5 cun with gentle rotation for 30 s, perpendicular insertion 1–1.5 cun at Fengchi, Wanggu, Tianzhu with 30 s rotation, oblique upward insertion 0.3–0.5 cun at Renzhong with strong bird-pecking manipulation until tearing occurs, perpendicular insertion 0.5–1 cun at Neiguan with rotation, lifting, and mild stimulation for 1 min, perpendicular insertion 0.2–0.5 cun at Shenmen and 0.5–1 cun at Sanyinjiao with gentle 30 s rotation, perpendicular insertion 1–1.5 cun at Fenglong and Taichong with gentle rotation, lifting, and 1 min insertion; needle retention 30 min at all points). | Body acupuncture（Nimodipine + acupuncture group: nimodipine + acupuncture [Baihui, Sishencong, Sibai, Fengchi, Wangu, Tianzhu, Renzhong, Shenmen, Neiguan, Fenglong, Sanyinjiao, Taichong], with 1-cun horizontal insertion at Baihui and Sishencong; oblique 0.5-cun insertion at Sibai with gentle 30-s rotation; vertical 1–1.5-cun insertion at Fengchi, Wangu, Tianzhu with 30-s rotation; oblique 0.3–0.5-cun “bird-pecking” at Renzhong until lacrimation; vertical 0.5–1-cun insertion at Neiguan with lifting–thrusting for 1 min; vertical insertion 0.2–0.5 cun at Shenmen and 0.5–1.0 cun at Sanyinjiao with gentle 30-s rotation; vertical 1–1.5-cun insertion at Fenglong and Taichong with lifting–thrusting for 1 min; needles retained for 30 min at all points） |  |
| Cai Jiang 2016 [59] | 52/51/52/49 | - 62.33±7.72/62.37 ±7.89/61.58±9.71/60.53 ± 9.19 | 97/107 | Fujian | 12 | Body acupuncture-Cognitive Training-Standard of care/Cognitive Training/Body acupuncture (ACRG group: acupuncture (Baihui, Shenting) + RehaCom cognitive training (3–5 different programs and difficulty levels) + routine rehabilitation (basic treatment and health education) / RehaCom cognitive training RTG (3–5 cognitive programs and difficulty levels) / acupuncture ATG (Baihui, Shenting)). | Standard of care（Routine rehabilitation CG: basic treatment and health education） |  |
| Wang He Yi 2022 [60] | 32/33/34 | 58.73 ± 11.80 / 62.12 ± 12.37 | 52/47 | Liaoning | 4 | Body acupuncture-NIBS-Standard of care/NIBS-Standard of care (tDCS + basic treatment group / ocular acupuncture + tDCS + basic treatment group: tDCS (anode over affected-side dorsolateral prefrontal cortex, cathode over contralateral supraorbital region, 1.5 mA for 20 min) + basic treatment / ocular acupuncture (bilateral heart, spleen, kidney, liver zones with gentle scraping of needle handle for 1 min, needle retention 30 min) + tDCS (same parameters) + basic treatment (medications: antiplatelet therapy, plaque stabilization, control of blood pressure, lipids, glucose, improvement of cerebral circulation, neurorepair; rehabilitation: cognitive-perceptual training, exercise therapy, occupational therapy, muscle strength training, gait training, physical therapy)). | Standard of care（Basic treatment group: medications (antiplatelet therapy, plaque stabilization, blood pressure/lipid/glucose control, improved cerebral circulation, neurotrophic therapy) + rehabilitation (cognitive–perceptual training, exercise therapy, occupational therapy, muscle strength training, gait training, physical therapy)） |  |
| Yang Lin 2019 [61] | 30/30/30 | 52.14 ± 7.56 / 50.10 ± 6.21 | 44/46 | Hunan, Changsha | 4 | Electroacupuncture-NIBS-Standard of care/NIBS-Standard of care (electroacupuncture scalp needling + hyperbaric oxygen + basic treatment group: electroacupuncture scalp needling (affected-side frontal midline, vertex midline, anterior parietotemporal oblique line, posterior parietotemporal oblique line) + hyperbaric oxygen (treatment pressure 0.12 MPa, pure oxygen via mask, 20 min pressurization with oxygen inhalation starting at 5 min, 60 min stable pressure oxygen inhalation with a 5 min air break, 15 min decompression) + basic rehabilitation (routine medications including reducing edema, improving cerebral circulation, neurotrophic therapy, occupational therapy, cognitive rehabilitation training) / hyperbaric oxygen + basic treatment group: hyperbaric oxygen (same 0.12 MPa protocol) + basic rehabilitation (same routine medications and cognitive rehabilitation)). | Electroacupuncture-Standard of care（Electroacupuncture scalp needling + basic treatment group: electroacupuncture on frontal midline, vertex midline, anterior and posterior oblique lines of the vertex–temporal region + basic rehabilitation (routine medications such as anti-edema therapy, circulation improvement, neurotrophic agents, occupational therapy, and cognitive rehabilitation training)） |  |
| Liu Li 2019 [62] | 41/40/39 | 55.00 ± 13.84 / 54.68 ± 11.51 | 60/60 | Hubei | 4 | Scalp acupuncture-NIBS-Standard of care/Scalp acupuncture-Standard of care (scalp acupuncture + rTMS + routine rehabilitation group: scalp acupuncture (frontal midline, vertex midline, contralateral temporal anterior line from Xuanliao to Xuanli) + rTMS (left dorsolateral prefrontal cortex) + routine rehabilitation (pharmacotherapy for underlying diseases, hemiplegia functional training, joint mobilization, exercise therapy, occupational therapy) / scalp acupuncture (same point prescription) + routine rehabilitation (same underlying disease treatment and functional rehabilitation)). | NIBS-Standard of care（rTMS treatment (left dorsolateral prefrontal cortex) + routine rehabilitation (medications for underlying diseases and comprehensive hemiplegic limb training, joint mobilization, exercise therapy, and occupational therapy)） |  |
| Huang Fan 2008 [63] | 40/40 | 59.22 ± 10.6 / 61.05 ± 9.68 | 41/39 | Guangdong | 4 | Body acupuncture-Standard of care (acupuncture + basic treatment group: acupuncture (Baihui, Renzhong, Shenmen; additional points: kidney deficiency with marrow depletion: Shenshu, Taixi; heart-liver yin deficiency: Neiguan, Sanyinjiao; phlegm obstruction: Sanyinjiao, Fenglong; Qi stagnation and blood stasis: Zusanli, Xuanzhong) + basic treatment (hemiplegic limb rehabilitation, cognitive training, antihypertensive and glucose-lowering medication)). | Standard of care（Basic treatment group: hemiplegic limb rehabilitation training, cognitive function training, antihypertensive and glucose-regulating medications） |  |
| Zhang Xiaoli 2012 [64] | 30/30 | \| 68.80±7.25 / 67.93±10.63 \| \| --- \| | 34/26 | Jiangsu, Yizheng | 4 | Electroacupuncture-Standard of care (electroacupuncture + Western medicine + routine treatment group: electroacupuncture (Baihui, Sishencong, Shenting, Benshen, Neiguan, Shenmen, Sanyinjiao, Taixi) + Western medicine (nimodipine) + routine treatment (antiplatelet therapy, fibrinogen reduction, dehydration, promoting blood circulation and removing stasis)). | Standard of care（Western medicine + routine treatment group: Western medicine [nimodipine] + routine treatment [antiplatelet therapy, defibrase therapy, dehydration therapy, and blood-activating/stasis-resolving treatment]） |  |
| Bai Jing 2012 [65] | 30/30 | 60±6/60±6 | 34/26 | Cangzhou | 4 | Scalp acupuncture-Cognitive Training-Standard of care (scalp cluster needling + Western medicine + rehabilitation + routine treatment group: scalp cluster needling (frontal, parietal, temporal regions) + Western medicine (piracetam tablets) + rehabilitation (early proper limb positioning, joint mobility training, balance training, transfer training, gait training, occupational therapy) + routine treatment (antiplatelet therapy, blood pressure and glucose control, cognitive training including attention, memory, reasoning, and other correctional therapies)). | Cognitive Training-Standard of care（Western medicine + rehabilitation training + routine treatment group: Western medicine [piracetam tablets] + rehabilitation training [early proper positioning, range-of-motion training, balance training, transfer training, gait training, occupational therapy] + routine treatment [antiplatelet therapy, blood pressure control, glucose control, and cognitive training such as attention, memory, reasoning training, and related corrective therapy]） |  |
| Xiao Bao 2021 [66] | 72/72 | 62.4±3.93 / 61.6±4.75 | 65/79 | Northern Guangdong | 8 | Body acupuncture-Standard of care (acupuncture + routine rehabilitation group: acupuncture (Baihui, Shenting, Fengchi, Jianyu, Quchi, Waiguan, Hegu, Huantiao, Liangqiu, Zusanli, Sanyinjiao, Taichong) + cognitive training, physical therapy, activities of daily living training). | Standard of care（Routine rehabilitation group: cognitive function training, physical therapy training, and activities of daily living training） |  |
| Tian Yali 2021 [67] | 25/25 | 68.19±0.16 / 68.21±0.15 | 29/21 | Zhengzhou | 8 | Body acupuncture-Cognitive Training (acupuncture + cognitive rehabilitation group: acupuncture (Sishencong, Baihui, Fengchi, Quchi, Shenting, Neiguan, Waiguan, Xuehai) + cognitive rehabilitation (attention training, calculation training, memory training, reasoning training)). | Cognitive Training（Cognitive rehabilitation group: attention training, calculation training, memory training, and reasoning training） |  |
| Li Fei 2013 [68] | 37/36/20 | 62±12 / 61±14 | 39/34 | Anhui | 4 | Special acupuncture-Standard of care/Standard of care (meridian Jing-well needling + temporal three-needle + routine treatment group / routine treatment group: Jing-well and temporal three-needle method (Baihui, Sishencong, Shenting, Benshen; temporal three-needle at Rate Valley–Jiaosun, Xuanli–Qubin, Tianchong; plus Shanzhong, Zhongwan, Qihai, Xuehai, Zusanli, Waiguan, syndrome-based Jing-well selection) + routine treatment (blood pressure and glucose control, antiplatelet therapy, aspirin for ischemic stroke sequelae, citicoline) / routine treatment alone (same protocol)). | Placebo（Control group: no treatment） |  |
| Han Chengyan 2018 [69] | 15/15/15 | **60.1±7.9 / 61.6±9.2** | **26/19** | Zhejiang | 8 | Scalp acupuncture-Cognitive Training-Standard of care/Scalp acupuncture-Cognitive Training-Standard of care (scalp acupuncture + cognitive training + basic treatment group: scalp acupuncture (Jin’s three-needle system: Zhizhen (Shenting, bilateral Benshen), Sishen needle, temporal three-needle) + cognitive training (attention, memory, apraxia/agnosia training, calculation, executive and problem-solving, language training) + basic treatment (neurotrophic support, improving cerebral blood flow, antiplatelet therapy, plaque stabilization, motor/sensory/balance/swallowing rehabilitation) / scalp acupuncture (same Jin’s three-needle prescription) + cognitive training (attention, memory, apraxia)). | Standard of care（Agnosia training, calculation training, executive and problem-solving training, and language training + basic treatment [neurotrophic therapy, improved cerebral blood flow, antiplatelet therapy, plaque stabilization, limb motor function training, sensory training, balance training, and swallowing training] / basic treatment [neurotrophic therapy, improved cerebral blood flow, antiplatelet therapy, plaque stabilization, limb motor function training, sensory training, balance training, and swallowing training]） |  |
| Wang Yongxin 2017 [70] | 40/40/40 | **45.39±11.42 / 52.65±7.53** | **51/43** | Hebei, Cangzhou | 4 | Scalp acupuncture-Cognitive Training-Standard of care/Scalp acupuncture-Standard of care (Yu’s scalp cluster needling + cognitive rehabilitation + routine treatment group: Yu’s scalp cluster needling (parietal area, pre-parietal area, frontal area, 6–8 h retention) + cognitive rehabilitation (object-manipulation tasks, card-based tasks, progressing from simple to complex, single to multiple, part to whole) + routine treatment (symptomatic and supportive care, reducing cerebral edema, lowering intracranial pressure, maintaining fluid and acid–base balance, improving cerebral circulation, anti-infection) / Yu’s scalp cluster needling (same areas and retention) + routine treatment (same supportive protocol)). | Cognitive Training-Standard of care（Cognitive rehabilitation + routine treatment group: cognitive rehabilitation [object manipulation, card-based exercises, progression from simple to complex, single to multiple, part-to-whole training] + routine treatment [symptomatic and supportive care, reduction of cerebral edema, intracranial pressure control, maintenance of water–electrolyte and acid–base balance, improved cerebral circulation, and anti-infection treatment]） |  |

Table S4. Results of the Global Inconsistency Assessment

| **Outcome** | **Chi²** | **P-value** |
| --- | --- | --- |
| MOCA | 17.80 | 0.0585 |
| MMSE | 5.12 | 0.7442 |
| BI | 0.11 | 0.7453 |
| TER | 0.42 | 0.5165 |

Table S5. Node-Splitting Inconsistency Analysis for MoCA

| Side | Direct |  | Indirect |  | Difference |  |  | tau |
| --- | --- | --- | --- | --- | --- | --- | --- | --- |
|  | Coef. | Std. Err. | Coef. | Std. Err. | Coef. | Std. Err. | P>z |  |
| A B * | 0.3677176 | 0.4577189 | 1.46348 | 0.7456314 | -1.095762 | 0.8747101 | 0.21 | 0.4131665 |
| A D | 0.3100941 | 0.4836247 | 0.3236024 | 0.4617643 | -0.0135083 | 0.6689909 | 0.984 | 0.4283398 |
| A E * | -0.01008 | 0.4712866 | 0.2607503 | 1.151332 | -0.2708303 | 1.243759 | 0.828 | 0.4281031 |
| A L * | -0.2804545 | 0.3278878 | -1.38842 | 0.8026912 | 1.107965 | 0.8681437 | 0.202 | 0.4119925 |
| B E * | -0.3767435 | 0.4352149 | -3.557979 | 1.457816 | 3.181236 | 1.521592 | 0.037 | 0.3875767 |
| B F | -0.9546317 | 0.2613033 | 0.6360016 | 0.7145659 | -1.590633 | 0.7607978 | 0.037 | 0.387576 |
| B L * | -0.618559 | 0.4463958 | -1.910468 | 0.5736705 | 1.291909 | 0.727235 | 0.076 | 0.3983257 |
| C K | -0.9418791 | 0.4936111 | -0.3211983 | 0.5344679 | -0.6206808 | 0.7256198 | 0.392 | 0.4213231 |
| C L * | -1.453814 | 0.3410683 | -2.907032 | 0.988196 | 1.453218 | 1.042612 | 0.163 | 0.4107044 |
| D L | -0.8789377 | 0.1974735 | -0.1833307 | 0.4278736 | -0.695607 | 0.4712724 | 0.14 | 0.4047646 |
| D O | 1.046801 | 0.3405471 | 0.25404 | 0.3437121 | 0.7927615 | 0.4837966 | 0.101 | 0.3994852 |
| E L * | -0.2431281 | 0.4611859 | -1.503189 | 0.9901667 | 1.26006 | 1.093316 | 0.249 | 0.4153647 |
| F M | 0.7111255 | 0.4511938 | 2.301765 | 0.6125684 | -1.590639 | 0.7607995 | 0.037 | 0.3875769 |
| G L * | -0.1606284 | 0.4563089 | -0.8761947 | 628.2905 | 0.7155662 | 628.2906 | 0.999 | 0.418058 |
| H I * | -0.949885 | 0.5039898 | -0.5165441 | 1.052778 | -0.4333409 | 1.16547 | 0.71 | 0.4265963 |
| H K * | -0.6535209 | 0.5011843 | -1.086857 | 1.056789 | 0.4333363 | 1.165471 | 0.71 | 0.4265963 |
| I K * | 0.2963641 | 0.499172 | 0.0796945 | 0.3006801 | 0.2166696 | 0.582736 | 0.71 | 0.4265968 |
| I L | -0.8253304 | 0.1422503 | -0.6086626 | 0.5652188 | -0.2166678 | 0.5827325 | 0.71 | 0.426596 |
| J K * | -0.5051291 | 0.4859196 | 0.8344883 | 0.9921871 | -1.339617 | 1.103164 | 0.225 | 0.4149384 |
| J L * | -0.9367417 | 0.4864614 | -2.276356 | 0.9913905 | 1.339614 | 1.103164 | 0.225 | 0.4149386 |
| K L * | -0.36989 | 0.3200768 | -1.434426 | 0.2953716 | 1.064536 | 0.4357228 | 0.015 | 0.3787534 |
| K N | 0.9435116 | 0.2463202 | 1.921057 | 0.6220013 | -0.9775449 | 0.668803 | 0.144 | 0.4076825 |
| K O | -0.0998865 | 0.3349368 | 0.9577809 | 0.3148949 | -1.057667 | 0.4596373 | 0.021 | 0.3839106 |
| L O | 2.301438 | 0.4853578 | 1.157005 | 0.257563 | 1.144433 | 0.5494642 | 0.037 | 0.3877885 |
| M O | -0.4466158 | 0.2992184 | 1.144029 | 0.699448 | -1.590644 | 0.7607992 | 0.037 | 0.387577 |
| N O | -0.7551354 | 0.2889731 | -0.1291758 | 0.5325105 | -0.6259596 | 0.6059842 | 0.302 | 0.4162587 |

Table S6. Node-Splitting Inconsistency Analysis for MMSE

| Side | Direct |  | Indirect |  | Difference |  |  | tau |
| --- | --- | --- | --- | --- | --- | --- | --- | --- |
|  | Coef. | Std. Err. | Coef. | Std. Err. | Coef. | Std. Err. | P>z |  |
| A C * | 0.495168 | 1.091826 | 2.242088 | 2.854308 | -1.74692 | 3.056481 | 0.568 | 1.07378 |
| A E * | -0.050709 | 1.079722 | -0.4225023 | 89.40641 | 0.3717932 | 89.41292 | 0.997 | 1.061581 |
| A K * | -0.3385574 | 1.092193 | -2.084535 | 2.851854 | 1.745977 | 3.054598 | 0.568 | 1.073759 |
| B E * | -1.876326 | 1.11617 | -0.1016093 | 632.4419 | -1.774717 | 632.4429 | 0.998 | 1.061573 |
| C E * | -0.5458354 | 1.092092 | -2.296251 | 2.857276 | 1.750416 | 3.059253 | 0.567 | 1.073813 |
| C F | -0.7655372 | 0.6336847 | 0.10971 | 1.392239 | -0.8752472 | 1.529667 | 0.567 | 1.073814 |
| C K * | -0.8337231 | 1.092919 | -1.70908 | 1.070285 | 0.8753568 | 1.5297 | 0.567 | 1.073815 |
| D K | -1.053626 | 0.3160312 | 0.5733985 | 0.8606549 | -1.627024 | 0.9168518 | 0.076 | 1.02192 |
| D O | 1.176695 | 0.743148 | -0.4503622 | 0.5369834 | 1.627057 | 0.9168606 | 0.076 | 1.021921 |
| E K * | -0.2878879 | 1.092376 | -2.037876 | 2.856273 | 1.749988 | 3.058625 | 0.567 | 1.073806 |
| F M | 0.6070105 | 0.5529515 | 0.1806058 | 1.702202 | 0.4264047 | 1.789791 | 0.812 | 1.079047 |
| F O | -0.6057853 | 1.081709 | 1.004426 | 0.950473 | -1.610211 | 1.439585 | 0.263 | 1.057584 |
| G H * | -0.9680414 | 1.107998 | 0.7277761 | 2.397127 | -1.695817 | 2.639557 | 0.521 | 1.07491 |
| G J * | -0.7719429 | 1.107084 | -2.467738 | 2.398398 | 1.695795 | 2.639561 | 0.521 | 1.07491 |
| H J * | 0.1960984 | 1.105589 | -0.6518084 | 0.7207623 | 0.8479069 | 1.319782 | 0.521 | 1.074911 |
| H K | -1.298595 | 0.3123395 | -0.4507199 | 1.282221 | -0.8478754 | 1.319746 | 0.521 | 1.07491 |
| I J * | -0.437039 | 1.108538 | 0.1276827 | 2.332159 | -0.5647217 | 2.581387 | 0.827 | 1.079472 |
| I K * | -1.081216 | 1.109645 | -1.645932 | 2.330574 | 0.5647158 | 2.581381 | 0.827 | 1.079472 |
| J K * | -0.6441772 | 1.108684 | -0.9265377 | 0.6608429 | 0.2823605 | 1.290695 | 0.827 | 1.079473 |
| J N | 1.278809 | 0.5569055 | 0.460049 | 1.41741 | 0.81876 | 1.522812 | 0.591 | 1.075706 |
| J O | -0.0940828 | 0.7873616 | 0.306581 | 0.7489663 | -0.4006638 | 1.086682 | 0.712 | 1.076873 |
| K L * | 0.5880175 | 1.085926 | 1.123452 | 632.6825 | -0.5354348 | 632.6835 | 0.999 | 1.061573 |
| K M * | 0.4038086 | 1.125879 | 1.73982 | 0.8818518 | -1.336011 | 1.430128 | 0.35 | 1.064045 |
| K O | 0.6469151 | 0.5475103 | 1.330012 | 0.5804043 | -0.683097 | 0.7978947 | 0.392 | 1.066924 |
| K P * | 0.8076171 | 1.128642 | 3.479616 | 2.62815 | -2.671999 | 2.860245 | 0.35 | 1.064045 |
| M O | -1.19372 | 1.083348 | 0.4171754 | 0.9268097 | -1.610895 | 1.42569 | 0.259 | 1.057074 |
| M P * | 0.4038086 | 1.125879 | -2.26821 | 2.631709 | 2.672019 | 2.860251 | 0.35 | 1.064045 |
| N O | -0.7976945 | 0.6380722 | -1.937905 | 1.188664 | 1.140211 | 1.349166 | 0.398 | 1.068468 |

Table S7. Node-Splitting Inconsistency Analysis for BI

| Side | Direct |  | Indirect |  | Difference |  |  | tau |
| --- | --- | --- | --- | --- | --- | --- | --- | --- |
|  | Coef. | Std. Err. | Coef. | Std. Err. | Coef. | Std. Err. | P>z |  |
| A G * | -0.4916046 | 0.3507773 | -0.4275098 | 77.09909 | -0.0640948 | 77.09988 | 0.999 | 0.2859603 |
| B G * | -0.9807171 | 0.3698358 | -0.9831456 | 634.0083 | 0.0024285 | 634.0084 | 1 | 0.2859598 |
| C F * | -0.7501095 | 0.3825479 | -1.812254 | 640.9592 | 1.062144 | 640.9593 | 0.999 | 0.2859598 |
| C G * | -1.297267 | 0.389176 | -0.6599578 | 447.747 | -0.6373094 | 447.7471 | 0.999 | 0.2859595 |
| D G | -0.5681185 | 0.2227565 | -0.7286531 | 0.4412455 | 0.1605345 | 0.4941156 | 0.745 | 0.3101068 |
| D K | 0.2748364 | 0.3944454 | 0.4353706 | 0.2975969 | -0.1605342 | 0.4941165 | 0.745 | 0.3101073 |
| E G * | -0.5397175 | 0.1738395 | -0.9808573 | 315.5553 | 0.4411398 | 315.5554 | 0.999 | 0.2859598 |
| F G * | -0.5471579 | 0.3782764 | -0.0780295 | 282.116 | -0.4691283 | 282.1162 | 0.999 | 0.2859598 |
| F J * | 0.9509963 | 0.3957571 | -0.1124757 | 630.5051 | 1.063472 | 630.5052 | 0.999 | 0.2859597 |
| G H * | 0.9132438 | 0.3705585 | 0.9881635 | 621.4944 | -0.0749197 | 621.4945 | 1 | 0.2859584 |
| G I * | 0.0109763 | 0.3630069 | 0.9908869 | 626.4122 | -0.9799106 | 626.4123 | 0.999 | 0.2859601 |
| G K | 1.00349 | 0.1977658 | 0.8429553 | 0.4529979 | 0.1605342 | 0.4941158 | 0.745 | 0.3101073 |

Table S8. Node-Splitting Inconsistency Analysis for TER

| **Side** | **Direct** |  | **Indirect** |  | **Difference** |  |  | **tau** |
| --- | --- | --- | --- | --- | --- | --- | --- | --- |
|  | **Coef.** | **Std. Err.** | **Coef.** | **Std. Err.** | **Coef.** | **Std. Err.** | **P>z** |  |
| A J * | -0.2758476 | 0.0968956 | -0.2295579 | 29.98743 | -0.0462898 | 29.98759 | 0.999 | 5.17e-07 |
| B E * | -0.203599 | 0.1046652 | 0.1499619 | 292.2437 | -0.3535609 | 292.2438 | 0.999 | 4.70e-07 |
| C J * | -0.1670541 | 0.0846379 | -0.5422666 | 240.9122 | 0.3752125 | 240.9123 | 0.999 | 2.04e-07 |
| D J | -0.1766812 | 0.0646605 | -0.0784803 | 0.1389024 | -0.0982009 | 0.1513676 | 0.516 | 0.0325217 |
| D O | 0.1603427 | 0.1220919 | 0.0621398 | 0.089483 | 0.0982029 | 0.1513726 | 0.517 | 0.032524 |
| E F * | 0.4353181 | 0.2230081 | -0.1823381 | 418.2184 | 0.6176562 | 418.2185 | 0.999 | 3.31e-06 |
| E G * | 0.1999657 | 0.0579437 | -0.1279963 | 141.6806 | 0.327962 | 141.6806 | 0.998 | 5.23e-06 |
| E I * | 0.1335314 | 0.1503963 | -0.1299768 | 375.6186 | 0.2635082 | 375.6186 | 0.999 | 7.43e-08 |
| E M * | 0.164303 | 0.1265595 | 0.3352856 | 53.02105 | -0.1709825 | 53.0212 | 0.997 | 4.02e-07 |
| E N * | 0.1609304 | 0.0521523 | -0.1460808 | 185.0038 | 0.3070112 | 185.0038 | 0.999 | 1.67e-07 |
| H J * | -0.2464313 | 0.0694624 | -0.5108923 | 167.9534 | 0.264461 | 167.9534 | 0.999 | 4.04e-06 |
| J K * | 0.2097205 | 0.1017859 | 0.6509091 | 292.8728 | -0.4411885 | 292.8728 | 0.999 | 1.70e-07 |
| J L * | 0.4198538 | 0.1484914 | 0.5080914 | 395.1074 | -0.0882376 | 395.1075 | 1 | 3.65e-06 |
| J O | 0.2388225 | 0.0662392 | 0.3370224 | 0.1381607 | -0.0981999 | 0.1513706 | 0.517 | 0.0325218 |
| M O * | -0.2513144 | 0.1161553 | -0.0891555 | 51.1362 | -0.1621589 | 51.13634 | 0.997 | 1.30e-06 |

Table S9. SUCRA-Based Ranking Results for MoCA

| **Treatment** | **SUCRA** | **PrBest** | **MeanRank** |
| --- | --- | --- | --- |
| BA-C | 98.6 | 85.2 | 1.2 |
| ScA-N-SOC | 93.4 | 12.8 | 2 |
| EA-N-SOC | 82.2 | 1.4 | 3.7 |
| ScA-C-SOC | 75.8 | 0 | 4.6 |
| BA-C-SOC | 71 | 0 | 5.3 |
| ScAsim-C-SOC | 69.5 | 0.6 | 5.6 |
| EyeAcu-N-SOC | 60.6 | 0 | 6.9 |
| ScA-SOC | 51.6 | 0 | 8.3 |
| BA-SOC | 50.5 | 0 | 8.4 |
| C-SOC | 33.8 | 0 | 10.9 |
| N-SOC | 32.5 | 0 | 11.1 |
| EA-SOC | 24.9 | 0 | 12.3 |
| ScA-BA-SOC | 20.4 | 0 | 12.9 |
| BA | 19.5 | 0 | 13.1 |
| C | 15.8 | 0 | 13.6 |
| SOC | 0 | 0 | 16 |

Table S10. SUCRA-Based Ranking Results for MMSE

| **Treatment** | **SUCRA** | **PrBest** | **MeanRank** |
| --- | --- | --- | --- |
| BA-C | 98.6 | 85.2 | 1.2 |
| ScA-N-SOC | 93.4 | 12.8 | 2 |
| EA-N-SOC | 82.2 | 1.4 | 3.7 |
| ScA-C-SOC | 75.8 | 0 | 4.6 |
| BA-C-SOC | 71 | 0 | 5.3 |
| ScAsim-C-SOC | 69.5 | 0.6 | 5.6 |
| EyeAcu-N-SOC | 60.6 | 0 | 6.9 |
| ScA-SOC | 51.6 | 0 | 8.3 |
| BA-SOC | 50.5 | 0 | 8.4 |
| C-SOC | 33.8 | 0 | 10.9 |
| N-SOC | 32.5 | 0 | 11.1 |
| EA-SOC | 24.9 | 0 | 12.3 |
| ScA-BA-SOC | 20.4 | 0 | 12.9 |
| BA | 19.5 | 0 | 13.1 |
| C | 15.8 | 0 | 13.6 |
| SOC | 0 | 0 | 16 |

Table S11. SUCRA-Based Ranking Results for BI

| **Treatment** | **SUCRA** | **PrBest** | **MeanRank** |
| --- | --- | --- | --- |
| ScA-N-SOC | 93.9 | 65.3 | 1.6 |
| BA-N-SOC | 88.6 | 25 | 2.1 |
| BA-M-SOC | 71.9 | 5 | 3.8 |
| ScA-SOC | 71 | 1.2 | 3.9 |
| ScA-BA-SOC | 67.4 | 3.5 | 4.3 |
| BA-SOC | 39.4 | 0 | 7.1 |
| N-SOC | 39.1 | 0 | 7.1 |
| B-SOC | 34 | 0 | 7.6 |
| EA-SOC | 33 | 0 | 7.7 |
| ScA-E-SOC | 6.7 | 0 | 10.3 |
| SOC | 5 | 0 | 10.5 |

Table S12. SUCRA-Based Ranking Results for TER

| **Treatment** | **SUCRA** | **PrBest** | **MeanRank** |
| --- | --- | --- | --- |
| E-C-SOC | 92.4 | 69.3 | 2.1 |
| EA-C-SOC | 78.3 | 3.9 | 4 |
| BA-C-SOC | 77.2 | 7.8 | 4.2 |
| ScA-C-SOC | 74.5 | 4.3 | 4.6 |
| ScA-E-C-SOC | 71.2 | 1 | 5 |
| M-C-SOC | 65.4 | 5.7 | 5.8 |
| ScA-BA-SOC | 62.2 | 7.2 | 6.3 |
| C-SOC | 43.6 | 0 | 8.9 |
| B-SOC | 40.6 | 0.6 | 9.3 |
| ScA-SOC | 37.8 | 0 | 9.7 |
| EA-SOC | 35.9 | 0 | 10 |
| ScA-B-SOC | 29.4 | 0.1 | 10.9 |
| BA-M-SOC | 21.5 | 0 | 12 |
| BA-SOC | 19.4 | 0 | 12.3 |
| SOC | 0.8 | 0 | 14.9 |

Table S13. Sensitivity Analysis for MoCA

| **dropped_id** | **comparison** | **effect size** | **lower confidence interval** | **upper confidence interval** | **Connected Network Status** |
| --- | --- | --- | --- | --- | --- |
| 2 | y_A | 0.44244931 | -0.1665187 | 1.051417 | 1 |
| 2 | y_B | 1.0943061 | 0.3656072 | 1.823005 | 1 |
| 2 | y_C | 1.5990279 | 0.9492074 | 2.248848 | 1 |
| 2 | y_D | 0.78167259 | 0.4068489 | 1.156496 | 1 |
| 2 | y_E | 0.46488613 | -0.369142 | 1.298914 | 1 |
| 2 | y_F | 0.32099118 | -0.4733208 | 1.115303 | 1 |
| 2 | y_G | 0.16063749 | -0.7460242 | 1.067299 | 1 |
| 2 | y_H | 1.6692138 | 0.769361 | 2.569067 | 1 |
| 2 | y_I | 0.81129786 | 0.5416722 | 1.080923 | 1 |
| 2 | y_J | 1.1844133 | 0.3114159 | 2.057411 | 1 |
| 2 | y_K | 0.92579553 | 0.455091 | 1.3965 | 1 |
| 2 | y_M | 1.5693295 | 0.8498978 | 2.288761 | 1 |
| 2 | y_N | 1.9911426 | 1.394791 | 2.587494 | 1 |
| 2 | y_O | 1.3612541 | 0.8442561 | 1.878252 | 1 |
| 6 | y_A | 0.43896167 | -0.1452996 | 1.023223 | 1 |
| 6 | y_B | 1.104774 | 0.4078677 | 1.80168 | 1 |
| 6 | y_C | 1.6021545 | 0.9770698 | 2.227239 | 1 |
| 6 | y_D | 0.75560599 | 0.4052204 | 1.105992 | 1 |
| 6 | y_E | 0.46727234 | -0.332044 | 1.266589 | 1 |
| 6 | y_F | 0.33755564 | -0.4205472 | 1.095659 | 1 |
| 6 | y_G | 0.16063749 | -0.7071317 | 1.028407 | 1 |
| 6 | y_H | 1.6462482 | 0.7797999 | 2.512697 | 1 |
| 6 | y_I | 0.75453461 | 0.4890417 | 1.020028 | 1 |
| 6 | y_J | 1.1895503 | 0.350286 | 2.028815 | 1 |
| 6 | y_K | 0.93596782 | 0.491457 | 1.380479 | 1 |
| 6 | y_M | 1.6059971 | 0.9344563 | 2.277538 | 1 |
| 6 | y_N | 2.0140585 | 1.454982 | 2.573135 | 1 |
| 6 | y_O | 1.4055384 | 0.9473249 | 1.863752 | 1 |
| 7 | y_A | 0.44110332 | -0.1733173 | 1.055524 | 1 |
| 7 | y_B | 1.1095478 | 0.3774385 | 1.841657 | 1 |
| 7 | y_C | 1.6061212 | 0.952952 | 2.25929 | 1 |
| 7 | y_D | 0.76021895 | 0.3661194 | 1.154319 | 1 |
| 7 | y_E | 0.46952067 | -0.3702204 | 1.309262 | 1 |
| 7 | y_F | 0.34374686 | -0.4522456 | 1.139739 | 1 |
| 7 | y_G | 0.16063749 | -0.7521505 | 1.073425 | 1 |
| 7 | y_H | 1.6831103 | 0.7793807 | 2.58684 | 1 |
| 7 | y_I | 0.81365411 | 0.5424988 | 1.084809 | 1 |
| 7 | y_J | 1.1970107 | 0.3199902 | 2.074031 | 1 |
| 7 | y_K | 0.95095181 | 0.4858547 | 1.416049 | 1 |
| 7 | y_M | 1.6142202 | 0.9074522 | 2.320988 | 1 |
| 7 | y_N | 2.028755 | 1.443747 | 2.613763 | 1 |
| 7 | y_O | 1.416272 | 0.9339993 | 1.898545 | 1 |
| 8 | y_A | 0.43987669 | -0.1672645 | 1.047018 | 1 |
| 8 | y_B | 1.1076808 | 0.3831762 | 1.832186 | 1 |
| 8 | y_C | 1.6043705 | 0.9568062 | 2.251935 | 1 |
| 8 | y_D | 0.75700128 | 0.3942557 | 1.119747 | 1 |
| 8 | y_E | 0.46849837 | -0.3628984 | 1.299895 | 1 |
| 8 | y_F | 0.34135104 | -0.446358 | 1.12906 | 1 |
| 8 | y_G | 0.16063749 | -0.7432283 | 1.064503 | 1 |
| 8 | y_H | 1.667271 | 0.7699872 | 2.564555 | 1 |
| 8 | y_I | 0.788149 | 0.5086553 | 1.067643 | 1 |
| 8 | y_J | 1.1938322 | 0.3243699 | 2.063295 | 1 |
| 8 | y_K | 0.94458977 | 0.4840007 | 1.405179 | 1 |
| 8 | y_M | 1.6106582 | 0.9125849 | 2.308732 | 1 |
| 8 | y_N | 2.0229306 | 1.444162 | 2.601699 | 1 |
| 8 | y_O | 1.4118876 | 0.9375954 | 1.88618 | 1 |
| 10 | y_A | 0.44017042 | -0.1617764 | 1.042117 | 1 |
| 10 | y_B | 1.1081703 | 0.3899357 | 1.826405 | 1 |
| 10 | y_C | 1.6058418 | 0.9634018 | 2.248282 | 1 |
| 10 | y_D | 0.75753176 | 0.3976008 | 1.117463 | 1 |
| 10 | y_E | 0.46877143 | -0.355345 | 1.292888 | 1 |
| 10 | y_F | 0.34206288 | -0.4389148 | 1.123041 | 1 |
| 10 | y_G | 1.6816811 | 0.7924919 | 2.57087 | 1 |
| 10 | y_H | 0.81219669 | 0.5456117 | 1.078782 | 1 |
| 10 | y_I | 1.1963553 | 0.3337925 | 2.058918 | 1 |
| 10 | y_J | 0.94960883 | 0.4929514 | 1.406266 | 1 |
| 10 | y_L | 1.6124208 | 0.920399 | 2.304443 | 1 |
| 10 | y_M | 2.0264308 | 1.452282 | 2.60058 | 1 |
| 10 | y_N | 1.4138379 | 0.9432715 | 1.884404 | 1 |
| 11 | y_A | 0.42915789 | -0.1769276 | 1.035243 | 1 |
| 11 | y_B | 1.0629475 | 0.3327367 | 1.793158 | 1 |
| 11 | y_C | 1.607838 | 0.9618478 | 2.253828 | 1 |
| 11 | y_D | 0.76023696 | 0.3983122 | 1.122162 | 1 |
| 11 | y_E | 0.44996741 | -0.3803441 | 1.280279 | 1 |
| 11 | y_F | 0.43938157 | -0.3778146 | 1.256578 | 1 |
| 11 | y_G | 0.16063749 | -0.7407093 | 1.061984 | 1 |
| 11 | y_H | 1.6858037 | 0.7918037 | 2.579804 | 1 |
| 11 | y_I | 0.81318618 | 0.5450685 | 1.081304 | 1 |
| 11 | y_J | 1.1999392 | 0.3325935 | 2.067285 | 1 |
| 11 | y_K | 0.95677365 | 0.4973397 | 1.416208 | 1 |
| 11 | y_M | 1.6531222 | 0.950637 | 2.355608 | 1 |
| 11 | y_N | 2.0372215 | 1.45951 | 2.614933 | 1 |
| 11 | y_O | 1.4293923 | 0.955034 | 1.903751 | 1 |
| 15 | y_A | 0.44330321 | -0.1702601 | 1.056867 | 1 |
| 15 | y_B | 1.1111747 | 0.380012 | 1.842337 | 1 |
| 15 | y_C | 1.6065132 | 0.9540746 | 2.258952 | 1 |
| 15 | y_D | 0.7674848 | 0.3758047 | 1.159165 | 1 |
| 15 | y_E | 0.47080178 | -0.3678746 | 1.309478 | 1 |
| 15 | y_F | 0.3456277 | -0.4493399 | 1.140595 | 1 |
| 15 | y_G | 0.16063749 | -0.7509872 | 1.072262 | 1 |
| 15 | y_H | 1.683801 | 0.7810609 | 2.586541 | 1 |
| 15 | y_I | 0.81366489 | 0.542818 | 1.084512 | 1 |
| 15 | y_J | 1.1976929 | 0.3216569 | 2.073729 | 1 |
| 15 | y_K | 0.95231124 | 0.487791 | 1.416831 | 1 |
| 15 | y_M | 1.6169118 | 0.9111567 | 2.322667 | 1 |
| 15 | y_N | 2.0307599 | 1.446495 | 2.615025 | 1 |
| 15 | y_O | 1.4192889 | 0.9378284 | 1.900749 | 1 |
| 16 | y_A | 0.44088847 | -0.1618461 | 1.043623 | 1 |
| 16 | y_B | 1.1098332 | 0.390641 | 1.829025 | 1 |
| 16 | y_C | 1.6086419 | 0.9654078 | 2.251876 | 1 |
| 16 | y_D | 0.75874152 | 0.3983783 | 1.119105 | 1 |
| 16 | y_E | 0.46956564 | -0.3556544 | 1.294786 | 1 |
| 16 | y_F | 0.34433488 | -0.4376749 | 1.126345 | 1 |
| 16 | y_G | 0.16063749 | -0.7362818 | 1.057557 | 1 |
| 16 | y_H | 1.7088679 | 0.8171949 | 2.600541 | 1 |
| 16 | y_I | 0.8570629 | 0.5773338 | 1.136792 | 1 |
| 16 | y_J | 1.2013185 | 0.3376686 | 2.064968 | 1 |
| 16 | y_K | 0.95951736 | 0.5019417 | 1.417093 | 1 |
| 16 | y_M | 1.6164506 | 0.9234768 | 2.309424 | 1 |
| 16 | y_N | 2.0343044 | 1.45929 | 2.609319 | 1 |
| 16 | y_O | 1.418697 | 0.9474846 | 1.889909 | 1 |
| 18 | y_A | 0.44090758 | -0.1505823 | 1.032398 | 1 |
| 18 | y_B | 1.1094733 | 0.4038378 | 1.815109 | 1 |
| 18 | y_C | 1.6094064 | 0.9772206 | 2.241592 | 1 |
| 18 | y_D | 0.75884837 | 0.4045584 | 1.113138 | 1 |
| 18 | y_E | 0.46947711 | -0.3399775 | 1.278932 | 1 |
| 18 | y_F | 0.3439365 | -0.4235333 | 1.111406 | 1 |
| 18 | y_G | 0.16063749 | -0.7185429 | 1.039818 | 1 |
| 18 | y_H | 1.7165585 | 0.8398787 | 2.593238 | 1 |
| 18 | y_I | 0.87033988 | 0.5962278 | 1.144452 | 1 |
| 18 | y_J | 1.2024975 | 0.3536842 | 2.051311 | 1 |
| 18 | y_K | 0.96183168 | 0.5120918 | 1.411572 | 1 |
| 18 | y_M | 1.6168076 | 0.9368567 | 2.296758 | 1 |
| 18 | y_N | 2.0351117 | 1.469704 | 2.600519 | 1 |
| 18 | y_O | 1.4187482 | 0.9553906 | 1.882106 | 1 |
| 21 | y_A | 0.44509689 | -0.1476374 | 1.037831 | 1 |
| 21 | y_B | 1.0731701 | 0.3642683 | 1.782072 | 1 |
| 21 | y_C | 1.5892749 | 0.9554642 | 2.223086 | 1 |
| 21 | y_D | 0.81441302 | 0.449476 | 1.17935 | 1 |
| 21 | y_E | 0.4587438 | -0.3525163 | 1.270004 | 1 |
| 21 | y_F | 0.28931657 | -0.4835475 | 1.062181 | 1 |
| 21 | y_G | 0.16063749 | -0.7204303 | 1.041705 | 1 |
| 21 | y_H | 1.6491465 | 0.7709678 | 2.527325 | 1 |
| 21 | y_I | 0.80662028 | 0.5437942 | 1.069446 | 1 |
| 21 | y_J | 1.1669068 | 0.3154657 | 2.018348 | 1 |
| 21 | y_K | 0.8908055 | 0.4323972 | 1.349214 | 1 |
| 21 | y_M | 1.5074588 | 0.8087421 | 2.206176 | 1 |
| 21 | y_N | 1.9384275 | 1.357726 | 2.519129 | 1 |
| 21 | y_O | 1.285105 | 0.7837572 | 1.786453 | 1 |
| 22 | y_A | 0.42035354 | -0.1837938 | 1.024501 | 1 |
| 22 | y_B | 1.0268035 | 0.2917965 | 1.761811 | 1 |
| 22 | y_C | 1.6093461 | 0.9657954 | 2.252897 | 1 |
| 22 | y_D | 0.76224855 | 0.4016171 | 1.12288 | 1 |
| 22 | y_E | 0.4349484 | -0.3930543 | 1.262951 | 1 |
| 22 | y_F | 0.22675681 | -0.5840007 | 1.037514 | 1 |
| 22 | y_G | 0.16063749 | -0.7367699 | 1.058045 | 1 |
| 22 | y_H | 1.6884838 | 0.7977422 | 2.579226 | 1 |
| 22 | y_I | 0.81326047 | 0.546183 | 1.080338 | 1 |
| 22 | y_J | 1.2025684 | 0.3384797 | 2.066657 | 1 |
| 22 | y_K | 0.96201309 | 0.5039953 | 1.420031 | 1 |
| 22 | y_M | 1.3957884 | 0.5952669 | 2.19631 | 1 |
| 22 | y_N | 2.044959 | 1.468804 | 2.621114 | 1 |
| 22 | y_O | 1.4410154 | 0.9669511 | 1.91508 | 1 |
| 25 | y_A | 0.4381226 | -0.1232249 | 0.9994701 | 1 |
| 25 | y_B | 1.1019481 | 0.4326752 | 1.771221 | 1 |
| 25 | y_C | 1.6004138 | 0.9977585 | 2.203069 | 1 |
| 25 | y_D | 0.75435378 | 0.416292 | 1.092416 | 1 |
| 25 | y_E | 0.4661049 | -0.301055 | 1.233265 | 1 |
| 25 | y_F | 0.33388997 | -0.3945937 | 1.062374 | 1 |
| 25 | y_G | 0.16063748 | -0.6708928 | 0.9921678 | 1 |
| 25 | y_H | 1.6298499 | 0.793405 | 2.466295 | 1 |
| 25 | y_I | 0.72863818 | 0.4709723 | 0.986304 | 1 |
| 25 | y_J | 1.1860626 | 0.3769093 | 1.995216 | 1 |
| 25 | y_K | 0.92892458 | 0.5002149 | 1.357634 | 1 |
| 25 | y_M | 1.601834 | 0.956851 | 2.246817 | 1 |
| 25 | y_N | 2.0061862 | 1.466532 | 2.54584 | 1 |
| 25 | y_O | 1.3996784 | 0.9573538 | 1.842003 | 1 |
| 27 | y_A | 0.43991308 | -0.1728329 | 1.052659 | 1 |
| 27 | y_B | 1.1079518 | 0.3765984 | 1.839305 | 1 |
| 27 | y_C | 1.5617826 | 0.6748387 | 2.448727 | 1 |
| 27 | y_D | 0.75702761 | 0.3911232 | 1.122932 | 1 |
| 27 | y_E | 0.46858936 | -0.3706466 | 1.307825 | 1 |
| 27 | y_F | 0.3416793 | -0.453461 | 1.13682 | 1 |
| 27 | y_G | 0.16063748 | -0.7520129 | 1.073288 | 1 |
| 27 | y_H | 1.6794142 | 0.7747299 | 2.584099 | 1 |
| 27 | y_I | 0.81313583 | 0.5419485 | 1.084323 | 1 |
| 27 | y_J | 1.1936027 | 0.3157585 | 2.071447 | 1 |
| 27 | y_K | 0.94414911 | 0.4721068 | 1.416191 | 1 |
| 27 | y_M | 1.6107521 | 0.905411 | 2.316093 | 1 |
| 27 | y_N | 2.023058 | 1.435761 | 2.610355 | 1 |
| 27 | y_O | 1.4121706 | 0.9322473 | 1.892094 | 1 |
| 31 | y_A | 0.44705255 | -0.1662077 | 1.060313 | 1 |
| 31 | y_B | 1.1140008 | 0.3832518 | 1.84475 | 1 |
| 31 | y_C | 1.6071902 | 0.9551138 | 2.259267 | 1 |
| 31 | y_D | 0.7798379 | 0.3872243 | 1.172451 | 1 |
| 31 | y_E | 0.47300125 | -0.3651733 | 1.311176 | 1 |
| 31 | y_F | 0.34890223 | -0.4456267 | 1.143431 | 1 |
| 31 | y_G | 0.16063748 | -0.7504039 | 1.071679 | 1 |
| 31 | y_H | 1.685067 | 0.7828128 | 2.587321 | 1 |
| 31 | y_I | 0.81379668 | 0.5431041 | 1.084489 | 1 |
| 31 | y_J | 1.1988842 | 0.3233327 | 2.074436 | 1 |
| 31 | y_K | 0.95468796 | 0.4903885 | 1.418987 | 1 |
| 31 | y_M | 1.6215507 | 0.9161345 | 2.326967 | 1 |
| 31 | y_N | 2.0342918 | 1.450279 | 2.618305 | 1 |
| 31 | y_O | 1.4245206 | 0.9431513 | 1.90589 | 1 |
| 35 | y_A | 0.44049766 | -0.1732585 | 1.054254 | 1 |
| 35 | y_B | 1.1093183 | 0.3768398 | 1.841797 | 1 |
| 35 | y_C | 1.6064529 | 0.9523765 | 2.260529 | 1 |
| 35 | y_D | 0.75801025 | 0.3916841 | 1.124336 | 1 |
| 35 | y_E | 0.46923939 | -0.3714274 | 1.309906 | 1 |
| 35 | y_F | 0.3435427 | -0.4527211 | 1.139806 | 1 |
| 35 | y_G | 0.16063748 | -0.7536504 | 1.074925 | 1 |
| 35 | y_H | 1.6873549 | 0.7811418 | 2.593568 | 1 |
| 35 | y_I | 0.82100632 | 0.537479 | 1.104534 | 1 |
| 35 | y_J | 1.1976108 | 0.3194016 | 2.07582 | 1 |
| 35 | y_K | 0.95215274 | 0.4869195 | 1.417386 | 1 |
| 35 | y_M | 1.6140235 | 0.908291 | 2.319756 | 1 |
| 35 | y_N | 2.029449 | 1.445001 | 2.613897 | 1 |
| 35 | y_O | 1.4161283 | 0.9371958 | 1.895061 | 1 |
| 36 | y_A | 0.43977555 | -0.1663967 | 1.045948 | 1 |
| 36 | y_B | 1.1074173 | 0.3840779 | 1.830757 | 1 |
| 36 | y_C | 1.6040262 | 0.9574121 | 2.25064 | 1 |
| 36 | y_D | 0.75683636 | 0.3946133 | 1.119059 | 1 |
| 36 | y_E | 0.46837853 | -0.3616602 | 1.298417 | 1 |
| 36 | y_F | 0.34099757 | -0.4454632 | 1.127458 | 1 |
| 36 | y_G | 0.16063749 | -0.7417005 | 1.062975 | 1 |
| 36 | y_H | 1.663947 | 0.7677338 | 2.56016 | 1 |
| 36 | y_I | 0.78270491 | 0.5018093 | 1.063601 | 1 |
| 36 | y_J | 1.1932089 | 0.3250184 | 2.061399 | 1 |
| 36 | y_K | 0.9433425 | 0.483363 | 1.403322 | 1 |
| 36 | y_M | 1.6101081 | 0.9131463 | 2.30707 | 1 |
| 36 | y_N | 2.0218645 | 1.443879 | 2.59985 | 1 |
| 36 | y_O | 1.4111975 | 0.9375567 | 1.884838 | 1 |
| 39 | y_A | 0.4406275 | -0.1736113 | 1.054866 | 1 |
| 39 | y_B | 1.1096302 | 0.37657 | 1.84269 | 1 |
| 39 | y_C | 1.6069405 | 0.9523866 | 2.261494 | 1 |
| 39 | y_D | 0.75822695 | 0.3916389 | 1.124815 | 1 |
| 39 | y_E | 0.46938603 | -0.371957 | 1.310729 | 1 |
| 39 | y_F | 0.34396606 | -0.4529218 | 1.140854 | 1 |
| 39 | y_G | 0.16063748 | -0.7544104 | 1.075685 | 1 |
| 39 | y_H | 1.6920806 | 0.7850605 | 2.599101 | 1 |
| 39 | y_I | 0.82878706 | 0.5435891 | 1.113985 | 1 |
| 39 | y_J | 1.1984796 | 0.3196272 | 2.077332 | 1 |
| 39 | y_K | 0.95388845 | 0.4882799 | 1.419497 | 1 |
| 39 | y_M | 1.6147452 | 0.9084513 | 2.321039 | 1 |
| 39 | y_N | 2.0308564 | 1.445981 | 2.615732 | 1 |
| 39 | y_O | 1.4170089 | 0.9377334 | 1.896285 | 1 |
| 40 | y_A | 0.40229714 | -0.1665183 | 0.9711125 | 1 |
| 40 | y_B | 1.0207411 | 0.339124 | 1.702358 | 1 |
| 40 | y_C | 1.5729994 | 0.9633479 | 2.182651 | 1 |
| 40 | y_D | 0.69370576 | 0.3471288 | 1.040283 | 1 |
| 40 | y_E | 0.4269497 | -0.3501264 | 1.204026 | 1 |
| 40 | y_F | 0.22233886 | -0.5224452 | 0.9671229 | 1 |
| 40 | y_G | 0.16063748 | -0.6810126 | 1.002288 | 1 |
| 40 | y_H | 1.6156008 | 0.7696055 | 2.461596 | 1 |
| 40 | y_I | 0.79885838 | 0.5464047 | 1.051312 | 1 |
| 40 | y_J | 1.1376458 | 0.3183611 | 1.95693 | 1 |
| 40 | y_K | 0.8323374 | 0.3863198 | 1.278355 | 1 |
| 40 | y_M | 1.3992169 | 0.7175049 | 2.080929 | 1 |
| 40 | y_N | 1.850166 | 1.282102 | 2.41823 | 1 |
| 40 | y_O | 1.1574306 | 0.6519639 | 1.662897 | 1 |
| 41 | y_A | 0.4262772 | -0.1832822 | 1.035837 | 1 |
| 41 | y_B | 1.0979926 | 0.3716933 | 1.824292 | 1 |
| 41 | y_C | 1.603382 | 0.9549271 | 2.251837 | 1 |
| 41 | y_D | 0.71158046 | 0.3213058 | 1.101855 | 1 |
| 41 | y_E | 0.46070921 | -0.3723032 | 1.293722 | 1 |
| 41 | y_F | 0.33030792 | -0.4594459 | 1.120062 | 1 |
| 41 | y_G | 0.16063749 | -0.744607 | 1.065882 | 1 |
| 41 | y_H | 1.677476 | 0.7801094 | 2.574843 | 1 |
| 41 | y_I | 0.81232967 | 0.5431761 | 1.081483 | 1 |
| 41 | y_J | 1.1920977 | 0.3214105 | 2.062785 | 1 |
| 41 | y_K | 0.94113048 | 0.4794133 | 1.402848 | 1 |
| 41 | y_M | 1.5955087 | 0.8943802 | 2.296637 | 1 |
| 41 | y_N | 2.0139748 | 1.433146 | 2.594803 | 1 |
| 41 | y_O | 1.3949448 | 0.9162221 | 1.873668 | 1 |
| 45 | y_A | 0.4449274 | -0.1567241 | 1.046579 | 1 |
| 45 | y_B | 1.1189916 | 0.4009045 | 1.837079 | 1 |
| 45 | y_C | 1.5976696 | 0.9554012 | 2.239938 | 1 |
| 45 | y_D | 0.76558254 | 0.4055305 | 1.125635 | 1 |
| 45 | y_E | 0.47398005 | -0.3496878 | 1.297648 | 1 |
| 45 | y_F | 0.35689322 | -0.4241115 | 1.137898 | 1 |
| 45 | y_G | 0.16063749 | -0.7344724 | 1.055747 | 1 |
| 45 | y_H | 1.6659996 | 0.7767921 | 2.555207 | 1 |
| 45 | y_I | 0.81000086 | 0.5435097 | 1.076492 | 1 |
| 45 | y_J | 1.1818987 | 0.3193837 | 2.044414 | 1 |
| 45 | y_K | 0.92075401 | 0.4611285 | 1.380379 | 1 |
| 45 | y_M | 1.6392377 | 0.9458163 | 2.332659 | 1 |
| 45 | y_N | 2.1338163 | 1.525657 | 2.741976 | 1 |
| 45 | y_O | 1.4459916 | 0.971838 | 1.920145 | 1 |
| 48 | y_A | 0.44020072 | -0.1713511 | 1.051753 | 1 |
| 48 | y_B | 1.1085682 | 0.3787471 | 1.838389 | 1 |
| 48 | y_C | 1.6054008 | 0.9534965 | 2.257305 | 1 |
| 48 | y_D | 0.7575216 | 0.3923894 | 1.122654 | 1 |
| 48 | y_E | 0.468894 | -0.3686841 | 1.306472 | 1 |
| 48 | y_F | 0.34253237 | -0.4508801 | 1.135945 | 1 |
| 48 | y_G | 0.16063748 | -0.7501783 | 1.071453 | 1 |
| 48 | y_H | 1.6771825 | 0.7740551 | 2.58031 | 1 |
| 48 | y_I | 0.80431119 | 0.5230768 | 1.085546 | 1 |
| 48 | y_J | 1.1957189 | 0.3204288 | 2.071009 | 1 |
| 48 | y_K | 0.94836943 | 0.4847126 | 1.412026 | 1 |
| 48 | y_M | 1.6123948 | 0.9092172 | 2.315573 | 1 |
| 48 | y_N | 2.0262839 | 1.443743 | 2.608824 | 1 |
| 48 | y_O | 1.414107 | 0.9367278 | 1.891486 | 1 |
| 49 | y_A | 0.33030807 | -0.2464283 | 0.9070444 | 1 |
| 49 | y_B | 0.65850785 | -0.1374859 | 1.454502 | 1 |
| 49 | y_C | 1.6247439 | 1.015745 | 2.233743 | 1 |
| 49 | y_D | 0.78275927 | 0.4404894 | 1.125029 | 1 |
| 49 | y_E | 0.28176504 | -0.5134826 | 1.077013 | 1 |
| 49 | y_F | -0.2960951 | -1.243043 | 0.6508527 | 1 |
| 49 | y_G | 0.16063749 | -0.6807467 | 1.002022 | 1 |
| 49 | y_H | 1.7148489 | 0.8703767 | 2.559321 | 1 |
| 49 | y_I | 0.81237441 | 0.5600214 | 1.064727 | 1 |
| 49 | y_J | 1.2292525 | 0.411329 | 2.047176 | 1 |
| 49 | y_K | 1.0151295 | 0.5776366 | 1.452622 | 1 |
| 49 | y_M | 2.006427 | 1.254981 | 2.757873 | 1 |
| 49 | y_N | 2.1230986 | 1.570031 | 2.676167 | 1 |
| 49 | y_O | 1.559759 | 1.091574 | 2.027944 | 1 |
| 55 | y_A | 0.44471692 | -0.1675008 | 1.056935 | 1 |
| 55 | y_B | 1.1268616 | 0.3896385 | 1.864085 | 1 |
| 55 | y_C | 1.6052062 | 0.9531522 | 2.25726 | 1 |
| 55 | y_D | 0.75673883 | 0.3914809 | 1.121997 | 1 |
| 55 | y_E | 0.47651721 | -0.3623676 | 1.315402 | 1 |
| 55 | y_F | 0.3047607 | -0.5185084 | 1.12803 | 1 |
| 55 | y_G | 0.16063751 | -0.7504221 | 1.071697 | 1 |
| 55 | y_H | 1.6812697 | 0.7790905 | 2.583449 | 1 |
| 55 | y_I | 0.81327534 | 0.5425842 | 1.083966 | 1 |
| 55 | y_J | 1.1953771 | 0.3198891 | 2.070865 | 1 |
| 55 | y_K | 0.94768778 | 0.4839733 | 1.411402 | 1 |
| 55 | y_M | 1.5972768 | 0.8880597 | 2.306494 | 1 |
| 55 | y_N | 2.0238583 | 1.440882 | 2.606835 | 1 |
| 55 | y_O | 1.4091627 | 0.9305311 | 1.887794 | 1 |
| 57 | y_A | 0.44062813 | -0.1707096 | 1.051966 | 1 |
| 57 | y_B | 1.1095348 | 0.3799716 | 1.839098 | 1 |
| 57 | y_C | 1.6071076 | 0.9554135 | 2.258802 | 1 |
| 57 | y_D | 0.75824612 | 0.3932295 | 1.123263 | 1 |
| 57 | y_E | 0.46936042 | -0.3679177 | 1.306638 | 1 |
| 57 | y_F | 0.34385779 | -0.4492779 | 1.136993 | 1 |
| 57 | y_G | 0.16063748 | -0.749841 | 1.071116 | 1 |
| 57 | y_H | 1.6937682 | 0.7908896 | 2.596647 | 1 |
| 57 | y_I | 0.83170964 | 0.5502895 | 1.11313 | 1 |
| 57 | y_J | 1.1987331 | 0.3237246 | 2.073742 | 1 |
| 57 | y_K | 0.95438518 | 0.490866 | 1.417904 | 1 |
| 57 | y_M | 1.6148062 | 0.9118752 | 2.317737 | 1 |
| 57 | y_N | 2.0310041 | 1.44864 | 2.613369 | 1 |
| 57 | y_O | 1.4169944 | 0.9397619 | 1.894227 | 1 |
| 58 | y_A | 0.44033201 | -0.1729166 | 1.053581 | 1 |
| 58 | y_B | 1.108924 | 0.3770571 | 1.840791 | 1 |
| 58 | y_C | 1.6058249 | 0.9522488 | 2.259401 | 1 |
| 58 | y_D | 0.75773313 | 0.3916818 | 1.123784 | 1 |
| 58 | y_E | 0.46905327 | -0.3709024 | 1.309009 | 1 |
| 58 | y_F | 0.34300663 | -0.452601 | 1.138614 | 1 |
| 58 | y_G | 0.16063749 | -0.752851 | 1.074126 | 1 |
| 58 | y_H | 1.681265 | 0.7757688 | 2.586761 | 1 |
| 58 | y_I | 0.81097403 | 0.5280328 | 1.093915 | 1 |
| 58 | y_J | 1.1964933 | 0.3189563 | 2.07403 | 1 |
| 58 | y_K | 0.94992048 | 0.485051 | 1.41479 | 1 |
| 58 | y_M | 1.6131007 | 0.9079561 | 2.318245 | 1 |
| 58 | y_N | 2.0276483 | 1.443638 | 2.611658 | 1 |
| 58 | y_O | 1.4150055 | 0.9364295 | 1.893581 | 1 |
| 64 | y_A | 0.44088012 | -0.1581761 | 1.039936 | 1 |
| 64 | y_B | 1.1096844 | 0.3949267 | 1.824442 | 1 |
| 64 | y_C | 1.6088301 | 0.9692134 | 2.248447 | 1 |
| 64 | y_D | 0.75875144 | 0.4003761 | 1.117127 | 1 |
| 64 | y_E | 0.46952127 | -0.3505423 | 1.289585 | 1 |
| 64 | y_F | 0.34416141 | -0.4330919 | 1.121415 | 1 |
| 64 | y_G | 0.16063749 | -0.7304811 | 1.051756 | 1 |
| 64 | y_H | 1.7107813 | 0.8241239 | 2.597439 | 1 |
| 64 | y_I | 0.86040732 | 0.5835249 | 1.13729 | 1 |
| 64 | y_J | 1.2015955 | 0.3428043 | 2.060387 | 1 |
| 64 | y_K | 0.96005785 | 0.5050673 | 1.415048 | 1 |
| 64 | y_M | 1.616483 | 0.927769 | 2.305197 | 1 |
| 64 | y_N | 2.0344035 | 1.462537 | 2.60627 | 1 |
| 64 | y_O | 1.4186154 | 0.9499747 | 1.887256 | 1 |
| 68 | y_A | 0.43360565 | -0.1538261 | 1.021037 | 1 |
| 68 | y_B | 1.0927002 | 0.3918106 | 1.79359 | 1 |
| 68 | y_C | 1.6163958 | 0.9881154 | 2.244676 | 1 |
| 68 | y_D | 0.74652318 | 0.3942118 | 1.098835 | 1 |
| 68 | y_E | 0.46143731 | -0.3422992 | 1.265174 | 1 |
| 68 | y_F | 0.32097208 | -0.4415507 | 1.083495 | 1 |
| 68 | y_G | 0.1606375 | -0.7120581 | 1.033333 | 1 |
| 68 | y_H | 1.7006468 | 0.8303312 | 2.570962 | 1 |
| 68 | y_I | 0.81302039 | 0.552444 | 1.073597 | 1 |
| 68 | y_J | 1.2147975 | 0.3711015 | 2.058493 | 1 |
| 68 | y_K | 0.98636401 | 0.5372561 | 1.435472 | 1 |
| 68 | y_M | 1.5757074 | 0.8991292 | 2.252285 | 1 |
| 68 | y_N | 1.8823204 | 1.293714 | 2.470927 | 1 |
| 68 | y_O | 1.3693483 | 0.9059809 | 1.832716 | 1 |
| 70 | y_A | 0.44067537 | -0.1720825 | 1.053433 | 1 |
| 70 | y_B | 1.1096856 | 0.3779981 | 1.841373 | 1 |
| 70 | y_C | 1.6054502 | 0.9521372 | 2.258763 | 1 |
| 70 | y_D | 0.75831798 | 0.3919726 | 1.124663 | 1 |
| 70 | y_E | 0.46942391 | -0.369775 | 1.308623 | 1 |
| 70 | y_F | 0.34405433 | -0.4517331 | 1.139842 | 1 |
| 70 | y_G | 0.16063748 | -0.7518708 | 1.073146 | 1 |
| 70 | y_H | 1.6818115 | 0.7774726 | 2.58615 | 1 |
| 70 | y_I | 0.81345524 | 0.5423199 | 1.084591 | 1 |
| 70 | y_J | 1.1958219 | 0.3182946 | 2.073349 | 1 |
| 70 | y_K | 0.94857843 | 0.478065 | 1.419092 | 1 |
| 70 | y_M | 1.6150466 | 0.9070003 | 2.323093 | 1 |
| 70 | y_N | 2.0227064 | 1.39992 | 2.645493 | 1 |
| 70 | y_O | 1.4173221 | 0.9315432 | 1.903101 | 1 |
| 74 | y_A | 0.42834181 | -0.1769615 | 1.033645 | 1 |
| 74 | y_B | 1.0595984 | 0.3296994 | 1.789497 | 1 |
| 74 | y_C | 1.607968 | 0.9627948 | 2.253141 | 1 |
| 74 | y_D | 0.76040658 | 0.3989286 | 1.121884 | 1 |
| 74 | y_E | 0.44857688 | -0.3806736 | 1.277827 | 1 |
| 74 | y_F | 0.44631443 | -0.3723766 | 1.265005 | 1 |
| 74 | y_G | 0.16063748 | -0.7393981 | 1.060673 | 1 |
| 74 | y_H | 1.6859836 | 0.7930861 | 2.578881 | 1 |
| 74 | y_I | 0.81311341 | 0.54534 | 1.080887 | 1 |
| 74 | y_J | 1.2001566 | 0.3339074 | 2.066406 | 1 |
| 74 | y_K | 0.95720488 | 0.498335 | 1.416075 | 1 |
| 74 | y_M | 1.6559725 | 0.9539514 | 2.357994 | 1 |
| 74 | y_N | 2.0378398 | 1.460818 | 2.614862 | 1 |
| 74 | y_O | 1.4303792 | 0.9565242 | 1.904234 | 1 |
| 76 | y_A | 0.40072349 | -0.179215 | 0.980662 | 1 |
| 76 | y_B | 1.076617 | 0.3860356 | 1.767198 | 1 |
| 76 | y_C | 1.5984413 | 0.9790848 | 2.217798 | 1 |
| 76 | y_D | 0.62836795 | 0.2539237 | 1.002812 | 1 |
| 76 | y_E | 0.44507701 | -0.3463839 | 1.236538 | 1 |
| 76 | y_F | 0.3052381 | -0.4462174 | 1.056694 | 1 |
| 76 | y_G | 0.16063748 | -0.6978361 | 1.019111 | 1 |
| 76 | y_H | 1.6654165 | 0.8072594 | 2.523573 | 1 |
| 76 | y_I | 0.8070542 | 0.5502515 | 1.063857 | 1 |
| 76 | y_J | 1.182879 | 0.3512633 | 2.014495 | 1 |
| 76 | y_K | 0.92263407 | 0.4814872 | 1.363781 | 1 |
| 76 | y_M | 1.5617646 | 0.8949103 | 2.228619 | 1 |
| 76 | y_N | 1.9853975 | 1.429821 | 2.540974 | 1 |
| 76 | y_O | 1.3556747 | 0.8975637 | 1.813786 | 1 |
| 79 | y_A | 0.44963583 | -0.164215 | 1.063487 | 1 |
| 79 | y_B | 1.1301326 | 0.3965267 | 1.863739 | 1 |
| 79 | y_C | 1.5901048 | 0.9354054 | 2.244804 | 1 |
| 79 | y_D | 0.7734737 | 0.405757 | 1.14119 | 1 |
| 79 | y_E | 0.47925183 | -0.3613839 | 1.319888 | 1 |
| 79 | y_F | 0.3720467 | -0.4263238 | 1.170417 | 1 |
| 79 | y_G | 0.16063749 | -0.7533013 | 1.074576 | 1 |
| 79 | y_H | 1.6525115 | 0.7457131 | 2.55931 | 1 |
| 79 | y_I | 0.80952788 | 0.5379609 | 1.081095 | 1 |
| 79 | y_J | 1.1687095 | 0.2888455 | 2.048573 | 1 |
| 79 | y_K | 0.89446437 | 0.4149525 | 1.373976 | 1 |
| 79 | y_M | 1.6654852 | 0.9513752 | 2.379595 | 1 |
| 79 | y_N | 2.0381047 | 1.415837 | 2.660372 | 1 |
| 79 | y_O | 1.4778882 | 0.9809265 | 1.97485 | 1 |
| 82 | y_A | 0.43135944 | -0.1831459 | 1.045865 | 1 |
| 82 | y_B | 1.0719745 | 0.3181434 | 1.825806 | 1 |
| 82 | y_C | 1.6075695 | 0.9543082 | 2.260831 | 1 |
| 82 | y_D | 0.75991957 | 0.3939159 | 1.125923 | 1 |
| 82 | y_E | 0.45370591 | -0.3891759 | 1.296588 | 1 |
| 82 | y_F | 0.29067959 | -0.5453598 | 1.126719 | 1 |
| 82 | y_G | 0.16063748 | -0.7523046 | 1.07358 | 1 |
| 82 | y_H | 1.6858904 | 0.7820476 | 2.589733 | 1 |
| 82 | y_I | 0.81404768 | 0.5428541 | 1.085241 | 1 |
| 82 | y_J | 1.1995721 | 0.3224333 | 2.076711 | 1 |
| 82 | y_K | 0.95606485 | 0.4909826 | 1.421147 | 1 |
| 82 | y_M | 1.5149208 | 0.659674 | 2.370168 | 1 |
| 82 | y_N | 2.0363784 | 1.451443 | 2.621314 | 1 |
| 82 | y_O | 1.4274839 | 0.9455106 | 1.909457 | 1 |
| 84 | y_A | 0.51387679 | -0.3919873 | 1.419741 | 1 |
| 84 | y_B | 1.1393523 | 0.3561099 | 1.922595 | 1 |
| 84 | y_C | 1.6075396 | 0.9428681 | 2.272211 | 1 |
| 84 | y_D | 0.77629922 | 0.3692765 | 1.183322 | 1 |
| 84 | y_E | 0.50379788 | -0.4028494 | 1.410445 | 1 |
| 84 | y_F | 0.37017648 | -0.4639585 | 1.204311 | 1 |
| 84 | y_G | 0.16063749 | -0.7705249 | 1.0918 | 1 |
| 84 | y_H | 1.6867452 | 0.7674868 | 2.606004 | 1 |
| 84 | y_I | 0.81545621 | 0.5393968 | 1.091516 | 1 |
| 84 | y_J | 1.199682 | 0.3072101 | 2.092154 | 1 |
| 84 | y_K | 0.95632001 | 0.4829968 | 1.429643 | 1 |
| 84 | y_M | 1.6297663 | 0.9064168 | 2.353116 | 1 |
| 84 | y_N | 2.037077 | 1.441901 | 2.632253 | 1 |
| 84 | y_O | 1.4275014 | 0.9366262 | 1.918377 | 1 |
| 85 | y_A | 0.39492393 | -0.4011035 | 1.190951 | 1 |
| 85 | y_B | 2.2542385 | 0.9591902 | 3.549287 | 1 |
| 85 | y_C | 1.6252387 | 1.004987 | 2.245491 | 1 |
| 85 | y_D | 0.78933147 | 0.4369127 | 1.14175 | 1 |
| 85 | y_E | 1.2999989 | 0.1150951 | 2.484903 | 1 |
| 85 | y_F | 0.16063748 | -0.6989031 | 1.020178 | 1 |
| 85 | y_G | 1.7168559 | 0.8572106 | 2.576501 | 1 |
| 85 | y_H | 0.81417443 | 0.5570058 | 1.071343 | 1 |
| 85 | y_I | 1.2303138 | 0.3972695 | 2.063358 | 1 |
| 85 | y_J | 1.0172959 | 0.5716425 | 1.462949 | 1 |
| 85 | y_L | 2.0111375 | 1.243874 | 2.778401 | 1 |
| 85 | y_M | 2.1267001 | 1.563495 | 2.689906 | 1 |
| 85 | y_N | 1.5638091 | 1.086725 | 2.040894 | 1 |
| 87 | y_A | 0.45101965 | -0.1476319 | 1.049671 | 1 |
| 87 | y_B | 1.1327505 | 0.4176597 | 1.847841 | 1 |
| 87 | y_C | 1.6490724 | 1.006314 | 2.291831 | 1 |
| 87 | y_D | 0.77591075 | 0.4166561 | 1.135165 | 1 |
| 87 | y_E | 0.48062378 | -0.338737 | 1.299985 | 1 |
| 87 | y_F | 0.37577779 | -0.402566 | 1.154122 | 1 |
| 87 | y_G | 0.16063749 | -0.7294337 | 1.050709 | 1 |
| 87 | y_H | 1.7641856 | 0.8696081 | 2.658763 | 1 |
| 87 | y_I | 0.8230937 | 0.5572909 | 1.088897 | 1 |
| 87 | y_J | 1.1020773 | 0.5851665 | 1.618988 | 1 |
| 87 | y_L | 1.673655 | 0.9785982 | 2.368712 | 1 |
| 87 | y_M | 2.1462044 | 1.54285 | 2.749559 | 1 |
| 87 | y_N | 1.4871554 | 1.004069 | 1.970242 | 1 |
| 88 | y_A | 0.45384423 | -0.1471212 | 1.05481 | 1 |
| 88 | y_B | 1.1392654 | 0.4212073 | 1.857323 | 1 |
| 88 | y_C | 1.6583204 | 0.7112304 | 2.605411 | 1 |
| 88 | y_D | 0.78067507 | 0.4199338 | 1.141416 | 1 |
| 88 | y_E | 0.48374069 | -0.3388349 | 1.306316 | 1 |
| 88 | y_F | 0.38468231 | -0.3970009 | 1.166366 | 1 |
| 88 | y_G | 0.16063749 | -0.7329952 | 1.05427 | 1 |
| 88 | y_H | 1.7855561 | 0.8858381 | 2.685274 | 1 |
| 88 | y_I | 0.82630157 | 0.559387 | 1.093216 | 1 |
| 88 | y_J | 1.2923466 | 0.4207381 | 2.163955 | 1 |
| 88 | y_K | 1.1411901 | 0.6101788 | 1.672201 | 1 |
| 88 | y_M | 1.689516 | 0.9903265 | 2.388705 | 1 |
| 88 | y_N | 2.1772127 | 1.565235 | 2.78919 | 1 |
| 88 | y_O | 1.5062623 | 1.018442 | 1.994082 | 1 |
| 91 | y_A | 0.43713316 | -0.1747907 | 1.049057 | 1 |
| 91 | y_B | 1.1015838 | 0.3705914 | 1.832576 | 1 |
| 91 | y_C | 1.5932301 | 0.9377673 | 2.248693 | 1 |
| 91 | y_D | 0.75233035 | 0.386012 | 1.118649 | 1 |
| 91 | y_E | 0.46553366 | -0.3724437 | 1.303511 | 1 |
| 91 | y_F | 0.33296625 | -0.4623586 | 1.128291 | 1 |
| 91 | y_G | 0.16063749 | -0.7503995 | 1.071674 | 1 |
| 91 | y_H | 0.82633156 | 0.5472411 | 1.105422 | 1 |
| 91 | y_I | 1.1742061 | 0.2907885 | 2.057624 | 1 |
| 91 | y_J | 0.90543036 | 0.385125 | 1.425736 | 1 |
| 91 | y_L | 1.5951163 | 0.8854057 | 2.304827 | 1 |
| 91 | y_M | 1.9924753 | 1.38096 | 2.603991 | 1 |
| 91 | y_N | 1.3933749 | 0.9025297 | 1.88422 | 1 |
| 92 | y_A | 0.45769081 | -0.1382649 | 1.053646 | 1 |
| 92 | y_B | 1.1478355 | 0.4355413 | 1.86013 | 1 |
| 92 | y_C | 1.5755301 | 0.9382344 | 2.212826 | 1 |
| 92 | y_D | 0.78721597 | 0.4287749 | 1.145657 | 1 |
| 92 | y_E | 0.48790404 | -0.3275955 | 1.303404 | 1 |
| 92 | y_F | 0.39648298 | -0.3792796 | 1.172246 | 1 |
| 92 | y_G | 0.16063749 | -0.7249429 | 1.046218 | 1 |
| 92 | y_H | 1.6230677 | 0.7392616 | 2.506874 | 1 |
| 92 | y_I | 0.80339015 | 0.5392058 | 1.067575 | 1 |
| 92 | y_J | 1.1426446 | 0.2858485 | 1.999441 | 1 |
| 92 | y_K | 0.84239158 | 0.3704071 | 1.314376 | 1 |
| 92 | y_M | 1.711344 | 1.015427 | 2.407261 | 1 |
| 92 | y_N | 2.032942 | 1.413157 | 2.652727 | 1 |
| 92 | y_O | 1.5322383 | 1.042701 | 2.021776 | 1 |

Table S14. Sensitivity Analysis for MMSE

| **dropped_id** | **comparison** | **effect size** | **lower confidence interval** | **upper confidence interval** | **Connected Network Status** |
| --- | --- | --- | --- | --- | --- |
| 4 | y_A | 0.56295344 | -1.442087 | 2.567994 | 1 |
| 4 | y_B | 2.3885536 | -0.6005755 | 5.377683 | 1 |
| 4 | y_C | 1.28221 | -0.2209451 | 2.785365 | 1 |
| 4 | y_D | 0.86083818 | 0.2495349 | 1.472142 | 1 |
| 4 | y_E | 0.51223671 | -1.493132 | 2.517606 | 1 |
| 4 | y_F | 0.66744038 | -0.7328171 | 2.067698 | 1 |
| 4 | y_G | 1.9630599 | -0.0385403 | 3.96466 | 1 |
| 4 | y_H | 1.317873 | 0.6982304 | 1.937515 | 1 |
| 4 | y_I | 1.1940847 | -0.7654791 | 3.153648 | 1 |
| 4 | y_J | 0.86933481 | -0.2417915 | 1.980461 | 1 |
| 4 | y_L | 0.58809259 | -1.570029 | 2.746214 | 1 |
| 4 | y_M | 1.2337394 | -0.1425298 | 2.610008 | 1 |
| 4 | y_N | 2.0337315 | 0.7892655 | 3.278198 | 1 |
| 4 | y_O | 0.97371365 | 0.1859737 | 1.761454 | 1 |
| 4 | y_P | 1.2248729 | -0.8299205 | 3.279666 | 1 |
| 6 | y_A | 0.56246715 | -1.454365 | 2.579299 | 1 |
| 6 | y_B | 2.3880677 | -0.6180427 | 5.394178 | 1 |
| 6 | y_C | 1.2812419 | -0.2307148 | 2.793199 | 1 |
| 6 | y_D | 0.86804367 | 0.2273006 | 1.508787 | 1 |
| 6 | y_E | 0.51175087 | -1.505407 | 2.528909 | 1 |
| 6 | y_F | 0.66612034 | -0.7423282 | 2.074569 | 1 |
| 6 | y_G | 1.924925 | -0.0852887 | 3.935139 | 1 |
| 6 | y_H | 1.25622 | 0.6572466 | 1.855193 | 1 |
| 6 | y_I | 1.1867151 | -0.7840523 | 3.157482 | 1 |
| 6 | y_J | 0.85462924 | -0.2623237 | 1.971582 | 1 |
| 6 | y_L | 0.58809259 | -1.582593 | 2.758778 | 1 |
| 6 | y_M | 1.2323886 | -0.151824 | 2.616601 | 1 |
| 6 | y_N | 2.0240044 | 0.7723297 | 3.275679 | 1 |
| 6 | y_O | 0.97106911 | 0.1775612 | 1.764577 | 1 |
| 6 | y_P | 1.2241734 | -0.8418495 | 3.290196 | 1 |
| 7 | y_A | 0.55875963 | -1.432647 | 2.550166 | 1 |
| 7 | y_B | 2.3843607 | -0.5851145 | 5.353836 | 1 |
| 7 | y_C | 1.2738231 | -0.2192888 | 2.766935 | 1 |
| 7 | y_D | 0.77899839 | 0.1456377 | 1.412359 | 1 |
| 7 | y_E | 0.5080434 | -1.483693 | 2.49978 | 1 |
| 7 | y_F | 0.65626478 | -0.7347996 | 2.047329 | 1 |
| 7 | y_G | 1.9158978 | -0.06965 | 3.901446 | 1 |
| 7 | y_H | 1.2520984 | 0.6601761 | 1.844021 | 1 |
| 7 | y_I | 1.1797605 | -0.7666566 | 3.126178 | 1 |
| 7 | y_J | 0.84073667 | -0.2624725 | 1.943946 | 1 |
| 7 | y_L | 0.5880928 | -1.555471 | 2.731657 | 1 |
| 7 | y_M | 1.2228668 | -0.14449 | 2.590224 | 1 |
| 7 | y_N | 2.0064219 | 0.7701015 | 3.242742 | 1 |
| 7 | y_O | 0.9482427 | 0.164531 | 1.731954 | 1 |
| 7 | y_P | 1.2194359 | -0.8224002 | 3.261272 | 1 |
| 9 | y_A | 0.51868188 | -1.505007 | 2.542371 | 1 |
| 9 | y_B | 2.3442906 | -0.6627829 | 5.351364 | 1 |
| 9 | y_C | 1.1937309 | -0.3754267 | 2.762889 | 1 |
| 9 | y_D | 0.86125464 | 0.2478028 | 1.474706 | 1 |
| 9 | y_E | 0.46797343 | -1.556036 | 2.491983 | 1 |
| 9 | y_F | 0.71935281 | -0.7112914 | 2.149997 | 1 |
| 9 | y_G | 1.9263134 | -0.0792907 | 3.931917 | 1 |
| 9 | y_H | 1.2558758 | 0.6582175 | 1.853534 | 1 |
| 9 | y_I | 1.188279 | -0.7779402 | 3.154498 | 1 |
| 9 | y_J | 0.85774898 | -0.2565436 | 1.972041 | 1 |
| 9 | y_L | 0.58809259 | -1.577543 | 2.753729 | 1 |
| 9 | y_M | 1.2701358 | -0.1240828 | 2.664354 | 1 |
| 9 | y_N | 2.0281321 | 0.7794681 | 3.276796 | 1 |
| 9 | y_O | 0.97663334 | 0.1853687 | 1.767898 | 1 |
| 9 | y_P | 1.2431434 | -0.8206066 | 3.306893 | 1 |
| 10 | y_A | 0.54918256 | -1.469362 | 2.567727 | 1 |
| 10 | y_B | 2.3747859 | -0.6314366 | 5.381009 | 1 |
| 10 | y_C | 1.2546901 | -0.272488 | 2.781868 | 1 |
| 10 | y_D | 0.86002084 | 0.245575 | 1.474467 | 1 |
| 10 | y_E | 0.49846878 | -1.5204 | 2.517338 | 1 |
| 10 | y_F | 0.63064605 | -0.8077925 | 2.069085 | 1 |
| 10 | y_G | 1.9240784 | -0.0847785 | 3.932935 | 1 |
| 10 | y_H | 1.2559532 | 0.6573564 | 1.85455 | 1 |
| 10 | y_I | 1.1860025 | -0.7834308 | 3.155436 | 1 |
| 10 | y_J | 0.85320563 | -0.2627026 | 1.969114 | 1 |
| 10 | y_L | 0.58809278 | -1.581157 | 2.757343 | 1 |
| 10 | y_M | 1.2620068 | -0.1457631 | 2.669777 | 1 |
| 10 | y_N | 2.0221807 | 0.7717949 | 3.272567 | 1 |
| 10 | y_O | 0.9686761 | 0.1770078 | 1.760344 | 1 |
| 10 | y_P | 1.239054 | -0.829854 | 3.307962 | 1 |
| 12 | y_A | 0.56302572 | -1.451886 | 2.577937 | 1 |
| 12 | y_B | 2.3886265 | -0.6147165 | 5.391969 | 1 |
| 12 | y_C | 1.282357 | -0.2281752 | 2.792889 | 1 |
| 12 | y_D | 0.88107762 | 0.2412801 | 1.520875 | 1 |
| 12 | y_E | 0.51230942 | -1.502928 | 2.527547 | 1 |
| 12 | y_F | 0.66761425 | -0.7395189 | 2.074747 | 1 |
| 12 | y_G | 1.9258148 | -0.0825353 | 3.934165 | 1 |
| 12 | y_H | 1.2561246 | 0.6576844 | 1.854565 | 1 |
| 12 | y_I | 1.1876542 | -0.7812734 | 3.156582 | 1 |
| 12 | y_J | 0.85650243 | -0.259408 | 1.972413 | 1 |
| 12 | y_L | 0.58809278 | -1.580544 | 2.75673 | 1 |
| 12 | y_M | 1.2338605 | -0.1490767 | 2.616798 | 1 |
| 12 | y_N | 2.0264669 | 0.7759591 | 3.276975 | 1 |
| 12 | y_O | 0.9743711 | 0.1816232 | 1.767119 | 1 |
| 12 | y_P | 1.2249163 | -0.8392788 | 3.289111 | 1 |
| 13 | y_A | 0.56318178 | -1.433335 | 2.559698 | 1 |
| 13 | y_B | 2.3887816 | -0.5880663 | 5.36563 | 1 |
| 13 | y_C | 1.2826639 | -0.2141736 | 2.779501 | 1 |
| 13 | y_D | 0.86105536 | 0.2523218 | 1.469789 | 1 |
| 13 | y_E | 0.51246469 | -1.484382 | 2.509311 | 1 |
| 13 | y_F | 0.66806639 | -0.7263631 | 2.062496 | 1 |
| 13 | y_G | 1.9726382 | -0.0207327 | 3.966009 | 1 |
| 13 | y_H | 1.3331144 | 0.7155159 | 1.950713 | 1 |
| 13 | y_I | 1.1960618 | -0.7553404 | 3.147464 | 1 |
| 13 | y_J | 0.87327778 | -0.2332522 | 1.979808 | 1 |
| 13 | y_L | 0.58809251 | -1.560937 | 2.737122 | 1 |
| 13 | y_M | 1.2343952 | -0.1362234 | 2.605014 | 1 |
| 13 | y_N | 2.0365294 | 0.797207 | 3.275852 | 1 |
| 13 | y_O | 0.97488483 | 0.1904326 | 1.759337 | 1 |
| 13 | y_P | 1.2252178 | -0.8214681 | 3.271904 | 1 |
| 15 | y_A | 0.56294107 | -1.442721 | 2.568604 | 1 |
| 15 | y_B | 2.3885413 | -0.6014836 | 5.378566 | 1 |
| 15 | y_C | 1.2821855 | -0.2214305 | 2.785801 | 1 |
| 15 | y_D | 0.86082629 | 0.2493355 | 1.472317 | 1 |
| 15 | y_E | 0.51222436 | -1.493766 | 2.518215 | 1 |
| 15 | y_F | 0.66740621 | -0.7332764 | 2.068089 | 1 |
| 15 | y_G | 1.9625763 | -0.0396463 | 3.964799 | 1 |
| 15 | y_H | 1.3171039 | 0.697131 | 1.937077 | 1 |
| 15 | y_I | 1.1939845 | -0.7661755 | 3.154145 | 1 |
| 15 | y_J | 0.86913512 | -0.2423332 | 1.980603 | 1 |
| 15 | y_L | 0.58809259 | -1.570692 | 2.746877 | 1 |
| 15 | y_M | 1.2337028 | -0.1429787 | 2.610384 | 1 |
| 15 | y_N | 2.0335897 | 0.7887454 | 3.278434 | 1 |
| 15 | y_O | 0.97365395 | 0.1856734 | 1.761635 | 1 |
| 15 | y_P | 1.2248534 | -0.8305315 | 3.280238 | 1 |
| 18 | y_A | 0.55066005 | -1.457893 | 2.559214 | 1 |
| 18 | y_B | 2.3762628 | -0.6175526 | 5.370079 | 1 |
| 18 | y_C | 1.257641 | -0.2503512 | 2.765633 | 1 |
| 18 | y_D | 0.89666606 | 0.2684471 | 1.524885 | 1 |
| 18 | y_E | 0.49994574 | -1.508935 | 2.508826 | 1 |
| 18 | y_F | 0.63460521 | -0.7726888 | 2.041899 | 1 |
| 18 | y_G | 1.9020983 | -0.1012933 | 3.90549 | 1 |
| 18 | y_H | 1.2521302 | 0.6554781 | 1.848782 | 1 |
| 18 | y_I | 1.1659294 | -0.7979345 | 3.129793 | 1 |
| 18 | y_J | 0.81313505 | -0.3094412 | 1.935711 | 1 |
| 18 | y_L | 0.5880927 | -1.573256 | 2.749442 | 1 |
| 18 | y_M | 1.2015801 | -0.1815381 | 2.584698 | 1 |
| 18 | y_N | 1.9703292 | 0.7084178 | 3.232241 | 1 |
| 18 | y_O | 0.9000563 | 0.0677223 | 1.73239 | 1 |
| 18 | y_P | 1.2087127 | -0.8497789 | 3.267204 | 1 |
| 20 | y_A | 0.56203191 | -1.453583 | 2.577647 | 1 |
| 20 | y_B | 2.3876329 | -0.616734 | 5.392 | 1 |
| 20 | y_C | 1.2803709 | -0.230622 | 2.791364 | 1 |
| 20 | y_D | 0.85998205 | 0.2454907 | 1.474473 | 1 |
| 20 | y_E | 0.51131581 | -1.504625 | 2.527257 | 1 |
| 20 | y_F | 0.66495903 | -0.742529 | 2.072447 | 1 |
| 20 | y_G | 1.9191227 | -0.0926893 | 3.930935 | 1 |
| 20 | y_H | 1.2478954 | 0.6257005 | 1.87009 | 1 |
| 20 | y_I | 1.1850686 | -0.7846209 | 3.154758 | 1 |
| 20 | y_J | 0.85134165 | -0.2654881 | 1.968171 | 1 |
| 20 | y_L | 0.58809279 | -1.581309 | 2.757494 | 1 |
| 20 | y_M | 1.2312577 | -0.1520224 | 2.614538 | 1 |
| 20 | y_N | 2.020983 | 0.770135 | 3.271831 | 1 |
| 20 | y_O | 0.96842693 | 0.1766081 | 1.760246 | 1 |
| 20 | y_P | 1.2236077 | -0.841247 | 3.288462 | 1 |
| 24 | y_A | 0.56281353 | -1.45361 | 2.579237 | 1 |
| 24 | y_B | 2.3884144 | -0.6171077 | 5.393937 | 1 |
| 24 | y_C | 1.2819333 | -0.2297218 | 2.793588 | 1 |
| 24 | y_D | 0.87616755 | 0.2352047 | 1.51713 | 1 |
| 24 | y_E | 0.51209732 | -1.504653 | 2.528847 | 1 |
| 24 | y_F | 0.66704476 | -0.7411265 | 2.075216 | 1 |
| 24 | y_G | 1.9255395 | -0.0842786 | 3.935358 | 1 |
| 24 | y_H | 1.256257 | 0.6573969 | 1.855117 | 1 |
| 24 | y_I | 1.1873116 | -0.7830652 | 3.157688 | 1 |
| 24 | y_J | 0.85581921 | -0.2609171 | 1.972556 | 1 |
| 24 | y_L | 0.5880928 | -1.582157 | 2.758343 | 1 |
| 24 | y_M | 1.2332955 | -0.1506483 | 2.617239 | 1 |
| 24 | y_N | 2.0255549 | 0.7741206 | 3.276989 | 1 |
| 24 | y_O | 0.97313276 | 0.1797647 | 1.766501 | 1 |
| 24 | y_P | 1.2246299 | -0.8410047 | 3.290264 | 1 |
| 25 | y_A | 0.56051095 | -1.064306 | 2.185328 | 1 |
| 25 | y_B | 2.3860971 | -0.0573102 | 4.829504 | 1 |
| 25 | y_C | 1.277162 | 0.0555003 | 2.498824 | 1 |
| 25 | y_D | 0.85913112 | 0.3623146 | 1.355948 | 1 |
| 25 | y_E | 0.50977712 | -1.11545 | 2.135004 | 1 |
| 25 | y_F | 0.66187661 | -0.4789521 | 1.802705 | 1 |
| 25 | y_G | 1.7299189 | 0.0979821 | 3.361856 | 1 |
| 25 | y_H | 0.94518569 | 0.446456 | 1.443915 | 1 |
| 25 | y_I | 1.1471816 | -0.4490081 | 2.743371 | 1 |
| 25 | y_J | 0.77558165 | -0.1299432 | 1.681107 | 1 |
| 25 | y_L | 0.58809259 | -1.164887 | 2.341073 | 1 |
| 25 | y_M | 1.2308179 | 0.1057489 | 2.355887 | 1 |
| 25 | y_N | 1.9677647 | 0.9525722 | 2.982957 | 1 |
| 25 | y_O | 0.94747511 | 0.3062326 | 1.588718 | 1 |
| 25 | y_P | 1.2243121 | -0.4717404 | 2.920364 | 1 |
| 27 | y_A | 0.56078639 | -0.9678331 | 2.089406 | 1 |
| 27 | y_B | 2.386366 | 0.0801491 | 4.692583 | 1 |
| 27 | y_C | 1.2776498 | 0.1270754 | 2.428224 | 1 |
| 27 | y_D | 0.85962312 | 0.3917118 | 1.327534 | 1 |
| 27 | y_E | 0.51004537 | -1.019011 | 2.039102 | 1 |
| 27 | y_F | 0.66297494 | -0.412442 | 1.738392 | 1 |
| 27 | y_G | 1.7038885 | 0.1645872 | 3.24319 | 1 |
| 27 | y_H | 0.90323306 | 0.431285 | 1.375181 | 1 |
| 27 | y_I | 1.1421472 | -0.3624781 | 2.646773 | 1 |
| 27 | y_J | 0.76549755 | -0.0883507 | 1.619346 | 1 |
| 27 | y_L | 0.58809259 | -1.062567 | 2.238753 | 1 |
| 27 | y_M | 1.2328117 | 0.1709438 | 2.29468 | 1 |
| 27 | y_N | 1.9607806 | 1.003267 | 2.918294 | 1 |
| 27 | y_O | 0.94498498 | 0.3406731 | 1.549297 | 1 |
| 27 | y_P | 1.2256424 | -0.3808489 | 2.832134 | 1 |
| 30 | y_A | 0.56301261 | -1.440047 | 2.566072 | 1 |
| 30 | y_B | 2.3886129 | -0.597662 | 5.374888 | 1 |
| 30 | y_C | 1.2823274 | -0.2193596 | 2.784014 | 1 |
| 30 | y_D | 0.86089399 | 0.2501879 | 1.4716 | 1 |
| 30 | y_E | 0.51229586 | -1.491092 | 2.515684 | 1 |
| 30 | y_F | 0.66760158 | -0.7313014 | 2.066505 | 1 |
| 30 | y_G | 1.9655767 | -0.0341129 | 3.965266 | 1 |
| 30 | y_H | 1.3218781 | 0.7026953 | 1.941061 | 1 |
| 30 | y_I | 1.1946039 | -0.7630633 | 3.152271 | 1 |
| 30 | y_J | 0.87037011 | -0.2396886 | 1.980429 | 1 |
| 30 | y_L | 0.58809266 | -1.567916 | 2.744102 | 1 |
| 30 | y_M | 1.2339071 | -0.1410489 | 2.608863 | 1 |
| 30 | y_N | 2.0344661 | 0.7911952 | 3.277737 | 1 |
| 30 | y_O | 0.97402073 | 0.1870448 | 1.760997 | 1 |
| 30 | y_P | 1.2249609 | -0.8279483 | 3.27787 | 1 |
| 32 | y_A | 0.5621444 | -1.455725 | 2.580014 | 1 |
| 32 | y_B | 2.3877454 | -0.6198704 | 5.395361 | 1 |
| 32 | y_C | 1.2805964 | -0.2320678 | 2.793261 | 1 |
| 32 | y_D | 0.86008347 | 0.2449123 | 1.475255 | 1 |
| 32 | y_E | 0.51142837 | -1.506767 | 2.529624 | 1 |
| 32 | y_F | 0.66525518 | -0.7437749 | 2.074285 | 1 |
| 32 | y_G | 1.925245 | -0.0888643 | 3.939354 | 1 |
| 32 | y_H | 1.2576543 | 0.6339315 | 1.881377 | 1 |
| 32 | y_I | 1.1863184 | -0.7855352 | 3.158172 | 1 |
| 32 | y_J | 0.85383712 | -0.2642448 | 1.971919 | 1 |
| 32 | y_L | 0.58809281 | -1.583714 | 2.759899 | 1 |
| 32 | y_M | 1.2315365 | -0.1532389 | 2.616312 | 1 |
| 32 | y_N | 2.0227486 | 0.7705235 | 3.274974 | 1 |
| 32 | y_O | 0.96915256 | 0.1764596 | 1.761845 | 1 |
| 32 | y_P | 1.2237438 | -0.8432565 | 3.290744 | 1 |
| 34 | y_A | 0.56305572 | -1.438925 | 2.565037 | 1 |
| 34 | y_B | 2.3886562 | -0.5960641 | 5.373376 | 1 |
| 34 | y_C | 1.2824128 | -0.2184746 | 2.7833 | 1 |
| 34 | y_D | 0.86093418 | 0.2505533 | 1.471315 | 1 |
| 34 | y_E | 0.51233902 | -1.48997 | 2.514648 | 1 |
| 34 | y_F | 0.66771799 | -0.7304476 | 2.065884 | 1 |
| 34 | y_G | 1.9674787 | -0.0312151 | 3.966172 | 1 |
| 34 | y_H | 1.3249057 | 0.7056056 | 1.944206 | 1 |
| 34 | y_I | 1.1949956 | -0.7616404 | 3.151632 | 1 |
| 34 | y_J | 0.87115129 | -0.2383396 | 1.980642 | 1 |
| 34 | y_L | 0.58809278 | -1.566765 | 2.742951 | 1 |
| 34 | y_M | 1.2340265 | -0.1402145 | 2.608268 | 1 |
| 34 | y_N | 2.0350201 | 0.792394 | 3.277646 | 1 |
| 34 | y_O | 0.97425163 | 0.1876901 | 1.760813 | 1 |
| 34 | y_P | 1.2250229 | -0.8268599 | 3.276906 | 1 |
| 35 | y_A | 0.54486027 | -1.457798 | 2.547519 | 1 |
| 35 | y_B | 2.3704639 | -0.6147329 | 5.355661 | 1 |
| 35 | y_C | 1.246048 | -0.2583154 | 2.750412 | 1 |
| 35 | y_D | 0.8442178 | 0.2320334 | 1.456402 | 1 |
| 35 | y_E | 0.49414681 | -1.50884 | 2.497133 | 1 |
| 35 | y_F | 0.61912825 | -0.7856697 | 2.023926 | 1 |
| 35 | y_G | 1.8907423 | -0.1073401 | 3.888825 | 1 |
| 35 | y_H | 1.249898 | 0.6548918 | 1.844904 | 1 |
| 35 | y_I | 1.1556885 | -0.8028786 | 3.114256 | 1 |
| 35 | y_J | 0.79269166 | -0.3301166 | 1.9155 | 1 |
| 35 | y_L | 0.58809264 | -1.566796 | 2.742981 | 1 |
| 35 | y_M | 1.1864574 | -0.1942497 | 2.567164 | 1 |
| 35 | y_N | 1.9439138 | 0.6804125 | 3.207415 | 1 |
| 35 | y_O | 0.86514219 | 0.0212367 | 1.709048 | 1 |
| 35 | y_P | 1.2011262 | -0.8518757 | 3.254128 | 1 |
| 38 | y_A | 0.56345049 | -1.450335 | 2.577236 | 1 |
| 38 | y_B | 2.3890508 | -0.6126691 | 5.39077 | 1 |
| 38 | y_C | 1.2832062 | -0.2264936 | 2.792906 | 1 |
| 38 | y_D | 0.89103994 | 0.2507336 | 1.531346 | 1 |
| 38 | y_E | 0.51273394 | -1.501378 | 2.526846 | 1 |
| 38 | y_F | 0.66875179 | -0.7376162 | 2.07512 | 1 |
| 38 | y_G | 1.9265205 | -0.0807377 | 3.933779 | 1 |
| 38 | y_H | 1.2560941 | 0.6579663 | 1.854222 | 1 |
| 38 | y_I | 1.1883759 | -0.779474 | 3.156226 | 1 |
| 38 | y_J | 0.85794321 | -0.2573679 | 1.973254 | 1 |
| 38 | y_L | 0.58809259 | -1.579343 | 2.755528 | 1 |
| 38 | y_M | 1.2349796 | -0.1472157 | 2.617175 | 1 |
| 38 | y_N | 2.0283551 | 0.7785125 | 3.278198 | 1 |
| 38 | y_O | 0.97689684 | 0.1845399 | 1.769254 | 1 |
| 38 | y_P | 1.2254803 | -0.8376436 | 3.288604 | 1 |
| 39 | y_A | 0.56352109 | -1.414799 | 2.541841 | 1 |
| 39 | y_B | 2.3891203 | -0.5615167 | 5.339757 | 1 |
| 39 | y_C | 1.2833362 | -0.2000152 | 2.766688 | 1 |
| 39 | y_D | 0.86138301 | 0.258135 | 1.464631 | 1 |
| 39 | y_E | 0.51280329 | -1.46585 | 2.491457 | 1 |
| 39 | y_F | 0.6690074 | -0.7129814 | 2.050996 | 1 |
| 39 | y_G | 1.9855677 | 0.0099557 | 3.96118 | 1 |
| 39 | y_H | 1.3536732 | 0.7420281 | 1.965318 | 1 |
| 39 | y_I | 1.1987441 | -0.7352294 | 3.132718 | 1 |
| 39 | y_J | 0.87862369 | -0.2180359 | 1.975283 | 1 |
| 39 | y_L | 0.58809243 | -1.541529 | 2.717714 | 1 |
| 39 | y_M | 1.2354101 | -0.1231472 | 2.593967 | 1 |
| 39 | y_N | 2.0403273 | 0.8120103 | 3.268644 | 1 |
| 39 | y_O | 0.9764867 | 0.1990596 | 1.753914 | 1 |
| 39 | y_P | 1.2257612 | -0.8036255 | 3.255148 | 1 |
| 40 | y_A | 0.56271223 | -1.453546 | 2.57897 | 1 |
| 40 | y_B | 2.3883127 | -0.6169708 | 5.393596 | 1 |
| 40 | y_C | 1.2817316 | -0.2297996 | 2.793263 | 1 |
| 40 | y_D | 0.87378707 | 0.2332809 | 1.514293 | 1 |
| 40 | y_E | 0.51199589 | -1.504588 | 2.52858 | 1 |
| 40 | y_F | 0.66677585 | -0.7412799 | 2.074832 | 1 |
| 40 | y_G | 1.9253378 | -0.0843192 | 3.934995 | 1 |
| 40 | y_H | 1.2562116 | 0.6573975 | 1.855026 | 1 |
| 40 | y_I | 1.1871325 | -0.7830852 | 3.15735 | 1 |
| 40 | y_J | 0.85546224 | -0.2611798 | 1.972104 | 1 |
| 40 | y_L | 0.58809259 | -1.581981 | 2.758166 | 1 |
| 40 | y_M | 1.2330331 | -0.1507986 | 2.616865 | 1 |
| 40 | y_N | 2.0250947 | 0.7737678 | 3.276422 | 1 |
| 40 | y_O | 0.9725259 | 0.1792428 | 1.765809 | 1 |
| 40 | y_P | 1.2244981 | -0.8409786 | 3.289975 | 1 |
| 42 | y_A | 0.55802795 | -1.459153 | 2.575209 | 1 |
| 42 | y_B | 2.3836297 | -0.6224837 | 5.389743 | 1 |
| 42 | y_C | 1.2723688 | -0.2428168 | 2.787555 | 1 |
| 42 | y_D | 0.85630184 | 0.2397073 | 1.472896 | 1 |
| 42 | y_E | 0.50731262 | -1.510194 | 2.524819 | 1 |
| 42 | y_F | 0.65426505 | -0.7605842 | 2.069114 | 1 |
| 42 | y_G | 1.9165835 | -0.0956249 | 3.928792 | 1 |
| 42 | y_H | 1.2550522 | 0.6560171 | 1.854087 | 1 |
| 42 | y_I | 1.1789545 | -0.7935504 | 3.151459 | 1 |
| 42 | y_J | 0.83913723 | -0.2918044 | 1.970079 | 1 |
| 42 | y_L | 0.5880928 | -1.582273 | 2.758459 | 1 |
| 42 | y_M | 1.2207849 | -0.1696689 | 2.611239 | 1 |
| 42 | y_N | 2.0039141 | 0.7312212 | 3.276607 | 1 |
| 42 | y_O | 0.94443262 | 0.0938077 | 1.795058 | 1 |
| 42 | y_P | 1.2183453 | -0.8484831 | 3.285174 | 1 |
| 44 | y_A | 0.56264457 | -1.450608 | 2.575898 | 1 |
| 44 | y_B | 2.3882453 | -0.6127178 | 5.389208 | 1 |
| 44 | y_C | 1.2815947 | -0.2276475 | 2.790837 | 1 |
| 44 | y_D | 0.86054704 | 0.2467678 | 1.474326 | 1 |
| 44 | y_E | 0.51192828 | -1.501651 | 2.525508 | 1 |
| 44 | y_F | 0.66659947 | -0.7392734 | 2.072472 | 1 |
| 44 | y_G | 1.9493336 | -0.0602053 | 3.958873 | 1 |
| 44 | y_H | 1.2960219 | 0.6743336 | 1.91771 | 1 |
| 44 | y_I | 1.1912593 | -0.7761688 | 3.158687 | 1 |
| 44 | y_J | 0.86369797 | -0.2518602 | 1.979256 | 1 |
| 44 | y_L | 0.58809277 | -1.578789 | 2.754975 | 1 |
| 44 | y_M | 1.2328752 | -0.1488388 | 2.614589 | 1 |
| 44 | y_N | 2.0297345 | 0.7803108 | 3.279158 | 1 |
| 44 | y_O | 0.97204758 | 0.1811395 | 1.762956 | 1 |
| 44 | y_P | 1.2244243 | -0.8381827 | 3.287031 | 1 |
| 45 | y_A | 0.56944912 | -1.45013 | 2.589028 | 1 |
| 45 | y_B | 2.3950487 | -0.6126956 | 5.402793 | 1 |
| 45 | y_C | 1.2951953 | -0.2325699 | 2.822961 | 1 |
| 45 | y_D | 0.86010304 | 0.2453333 | 1.474873 | 1 |
| 45 | y_E | 0.51873172 | -1.501172 | 2.538635 | 1 |
| 45 | y_F | 0.68476437 | -0.7540119 | 2.123541 | 1 |
| 45 | y_G | 1.9243347 | -0.0855653 | 3.934235 | 1 |
| 45 | y_H | 1.2561087 | 0.6572138 | 1.855004 | 1 |
| 45 | y_I | 1.1861804 | -0.784282 | 3.156643 | 1 |
| 45 | y_J | 0.85356123 | -0.2629287 | 1.970051 | 1 |
| 45 | y_L | 0.58809279 | -1.582303 | 2.758489 | 1 |
| 45 | y_M | 1.214297 | -0.1938665 | 2.622461 | 1 |
| 45 | y_N | 2.0226236 | 0.7715882 | 3.273659 | 1 |
| 45 | y_O | 0.969243 | 0.1771598 | 1.761326 | 1 |
| 45 | y_P | 1.2150863 | -0.8547893 | 3.284962 | 1 |
| 46 | y_A | 0.56258773 | -1.453952 | 2.579128 | 1 |
| 46 | y_B | 2.3881883 | -0.6175011 | 5.393878 | 1 |
| 46 | y_C | 1.2814828 | -0.2302571 | 2.793223 | 1 |
| 46 | y_D | 0.87086877 | 0.2302836 | 1.511454 | 1 |
| 46 | y_E | 0.51187142 | -1.504994 | 2.528737 | 1 |
| 46 | y_F | 0.66644286 | -0.7418054 | 2.074691 | 1 |
| 46 | y_G | 1.9251273 | -0.0848028 | 3.935057 | 1 |
| 46 | y_H | 1.2562146 | 0.6573225 | 1.855107 | 1 |
| 46 | y_I | 1.1869203 | -0.7835671 | 3.157408 | 1 |
| 46 | y_J | 0.8550387 | -0.2617555 | 1.971833 | 1 |
| 46 | y_L | 0.58809259 | -1.582281 | 2.758466 | 1 |
| 46 | y_M | 1.2327058 | -0.1513126 | 2.616724 | 1 |
| 46 | y_N | 2.0245405 | 0.7730436 | 3.276037 | 1 |
| 46 | y_O | 0.97178561 | 0.1783941 | 1.765177 | 1 |
| 46 | y_P | 1.2243332 | -0.8414115 | 3.290078 | 1 |
| 47 | y_A | 0.54372194 | -1.484098 | 2.571542 | 1 |
| 47 | y_B | 2.369326 | -0.6437799 | 5.382432 | 1 |
| 47 | y_C | 1.2437774 | -0.3279988 | 2.815553 | 1 |
| 47 | y_D | 0.86056716 | 0.2458394 | 1.475295 | 1 |
| 47 | y_E | 0.49300905 | -1.535131 | 2.521149 | 1 |
| 47 | y_F | 0.68813501 | -0.7451755 | 2.121445 | 1 |
| 47 | y_G | 1.9252621 | -0.0844494 | 3.934973 | 1 |
| 47 | y_H | 1.2562095 | 0.6573762 | 1.855043 | 1 |
| 47 | y_I | 1.1870578 | -0.7832155 | 3.157331 | 1 |
| 47 | y_J | 0.85531308 | -0.2612671 | 1.971893 | 1 |
| 47 | y_L | 0.58809259 | -1.582059 | 2.758244 | 1 |
| 47 | y_M | 1.2478469 | -0.1490569 | 2.644751 | 1 |
| 47 | y_N | 2.0249 | 0.7736809 | 3.276119 | 1 |
| 47 | y_O | 0.97226628 | 0.1793759 | 1.765157 | 1 |
| 47 | y_P | 1.2319393 | -0.8358181 | 3.299697 | 1 |
| 49 | y_A | 0.57376686 | -1.437761 | 2.585295 | 1 |
| 49 | y_B | 2.3993652 | -0.5986047 | 5.397335 | 1 |
| 49 | y_C | 1.3038237 | -0.2071561 | 2.814803 | 1 |
| 49 | y_D | 0.87076937 | 0.2558882 | 1.485651 | 1 |
| 49 | y_E | 0.52304834 | -1.488806 | 2.534903 | 1 |
| 49 | y_F | 0.69630637 | -0.7146447 | 2.107257 | 1 |
| 49 | y_G | 1.9456088 | -0.0611039 | 3.952322 | 1 |
| 49 | y_H | 1.2583608 | 0.6608993 | 1.855822 | 1 |
| 49 | y_I | 1.2063403 | -0.7607419 | 3.173423 | 1 |
| 49 | y_J | 0.8938009 | -0.2340047 | 2.021606 | 1 |
| 49 | y_L | 0.58809259 | -1.576247 | 2.752432 | 1 |
| 49 | y_M | 1.2619682 | -0.1247073 | 2.648644 | 1 |
| 49 | y_N | 2.0749182 | 0.8057597 | 3.344077 | 1 |
| 49 | y_O | 1.0387007 | 0.1905747 | 1.886827 | 1 |
| 49 | y_P | 1.2390428 | -0.8224046 | 3.30049 | 1 |
| 50 | y_A | 0.55670007 | -1.413794 | 2.527194 | 1 |
| 50 | y_B | 2.3823007 | -0.5570499 | 5.321651 | 1 |
| 50 | y_C | 1.2697009 | -0.2079415 | 2.747343 | 1 |
| 50 | y_D | 0.8551261 | 0.2541882 | 1.456064 | 1 |
| 50 | y_E | 0.50598342 | -1.464845 | 2.476811 | 1 |
| 50 | y_F | 0.65080536 | -0.7260227 | 2.027633 | 1 |
| 50 | y_G | 1.9808681 | 0.013206 | 3.94853 | 1 |
| 50 | y_H | 1.2584903 | 0.6722267 | 1.844754 | 1 |
| 50 | y_I | 1.2415747 | -0.6869389 | 3.170088 | 1 |
| 50 | y_J | 0.96410742 | -0.1426454 | 2.07086 | 1 |
| 50 | y_L | 0.58809259 | -1.53316 | 2.709346 | 1 |
| 50 | y_M | 1.2176278 | -0.1359279 | 2.571184 | 1 |
| 50 | y_N | 1.8114895 | 0.5412409 | 3.081738 | 1 |
| 50 | y_O | 0.93540477 | 0.1592001 | 1.711609 | 1 |
| 50 | y_P | 1.2168419 | -0.8051223 | 3.238806 | 1 |
| 51 | y_A | 0.5622271 | -1.421621 | 2.546075 | 1 |
| 51 | y_B | 2.3878284 | -0.5707714 | 5.346428 | 1 |
| 51 | y_C | 1.2807482 | -0.2066952 | 2.768192 | 1 |
| 51 | y_D | 0.86018675 | 0.255275 | 1.465099 | 1 |
| 51 | y_E | 0.51151046 | -1.47267 | 2.495691 | 1 |
| 51 | y_F | 0.6655348 | -0.7202229 | 2.051293 | 1 |
| 51 | y_G | 1.9217581 | -0.0564078 | 3.899924 | 1 |
| 51 | y_H | 1.25202 | 0.6623108 | 1.841729 | 1 |
| 51 | y_I | 1.1856673 | -0.7534804 | 3.124815 | 1 |
| 51 | y_J | 0.85252196 | -0.2462839 | 1.951328 | 1 |
| 51 | y_L | 1.2319812 | -0.1302299 | 2.594192 | 1 |
| 51 | y_M | 2.0218426 | 0.7905634 | 3.253122 | 1 |
| 51 | y_N | 0.96884028 | 0.1893812 | 1.748299 | 1 |
| 51 | y_O | 1.2240294 | -0.8106107 | 3.25867 | 1 |
| 55 | y_A | 0.56257209 | -1.452019 | 2.577163 | 1 |
| 55 | y_B | 2.3881726 | -0.6147183 | 5.391064 | 1 |
| 55 | y_C | 1.2814509 | -0.2287828 | 2.791685 | 1 |
| 55 | y_D | 0.86047983 | 0.2462973 | 1.474662 | 1 |
| 55 | y_E | 0.51185572 | -1.503062 | 2.526773 | 1 |
| 55 | y_F | 0.66640459 | -0.7403831 | 2.073192 | 1 |
| 55 | y_G | 1.9460234 | -0.0648136 | 3.956861 | 1 |
| 55 | y_H | 1.2907517 | 0.6686894 | 1.912814 | 1 |
| 55 | y_I | 1.1905786 | -0.7781308 | 3.159288 | 1 |
| 55 | y_J | 0.8623404 | -0.2539412 | 1.978622 | 1 |
| 55 | y_L | 0.58809259 | -1.580216 | 2.756402 | 1 |
| 55 | y_M | 1.2326779 | -0.1499232 | 2.615279 | 1 |
| 55 | y_N | 2.028772 | 0.7785401 | 3.279004 | 1 |
| 55 | y_O | 0.9716474 | 0.180223 | 1.763072 | 1 |
| 55 | y_P | 1.2243226 | -0.8395575 | 3.288203 | 1 |
| 56 | y_A | 0.57620155 | -1.442268 | 2.594671 | 1 |
| 56 | y_B | 2.4017995 | -0.604307 | 5.407906 | 1 |
| 56 | y_C | 1.3086912 | -0.2184852 | 2.835868 | 1 |
| 56 | y_D | 0.86013544 | 0.2457156 | 1.474555 | 1 |
| 56 | y_E | 0.52548274 | -1.493312 | 2.544277 | 1 |
| 56 | y_F | 0.70279965 | -0.7356974 | 2.141297 | 1 |
| 56 | y_G | 1.9243051 | -0.0844689 | 3.933079 | 1 |
| 56 | y_H | 1.2559748 | 0.6574025 | 1.854547 | 1 |
| 56 | y_I | 1.1862184 | -0.7831326 | 3.155569 | 1 |
| 56 | y_J | 0.85363713 | -0.2622254 | 1.9695 | 1 |
| 56 | y_L | 0.58809259 | -1.581066 | 2.757251 | 1 |
| 56 | y_M | 1.1984285 | -0.2093739 | 2.606231 | 1 |
| 56 | y_N | 2.0227419 | 0.7724074 | 3.273076 | 1 |
| 56 | y_O | 0.9694222 | 0.1777866 | 1.761058 | 1 |
| 56 | y_P | 1.2071168 | -0.8617243 | 3.275958 | 1 |
| 63 | y_A | 0.60496769 | -1.320807 | 2.530743 | 1 |
| 63 | y_B | 2.4305573 | -0.4439542 | 5.305069 | 1 |
| 63 | y_C | 1.366147 | -0.0811521 | 2.813446 | 1 |
| 63 | y_D | 0.89946104 | 0.310505 | 1.488417 | 1 |
| 63 | y_E | 0.55423997 | -1.371878 | 2.480358 | 1 |
| 63 | y_F | 0.77980726 | -0.5722534 | 2.131868 | 1 |
| 63 | y_G | 1.9966414 | 0.0731557 | 3.920127 | 1 |
| 63 | y_H | 1.2551539 | 0.6814181 | 1.82889 | 1 |
| 63 | y_I | 1.2590535 | -0.6258642 | 3.143971 | 1 |
| 63 | y_J | 0.99896078 | -0.0818366 | 2.079758 | 1 |
| 63 | y_L | 0.58809259 | -1.484823 | 2.661008 | 1 |
| 63 | y_M | 1.3442268 | 0.0146329 | 2.673821 | 1 |
| 63 | y_N | 2.2129152 | 0.9965328 | 3.429297 | 1 |
| 63 | y_O | 1.2234558 | 0.4111014 | 2.03581 | 1 |
| 63 | y_P | 1.2805484 | -0.6994134 | 3.26051 | 1 |
| 67 | y_A | 0.56206621 | -1.45583 | 2.579962 | 1 |
| 67 | y_B | 2.3876672 | -0.6199757 | 5.39531 | 1 |
| 67 | y_C | 1.2804401 | -0.2323069 | 2.793187 | 1 |
| 67 | y_D | 0.8586351 | 0.2168468 | 1.500423 | 1 |
| 67 | y_E | 0.5113502 | -1.506871 | 2.529572 | 1 |
| 67 | y_F | 0.66504635 | -0.7441335 | 2.074226 | 1 |
| 67 | y_G | 1.9242582 | -0.0869886 | 3.935505 | 1 |
| 67 | y_H | 1.256248 | 0.6569796 | 1.855516 | 1 |
| 67 | y_I | 1.1860335 | -0.7857535 | 3.15782 | 1 |
| 67 | y_J | 0.85326848 | -0.2642685 | 1.970805 | 1 |
| 67 | y_L | 0.58809282 | -1.583727 | 2.759912 | 1 |
| 67 | y_M | 1.2313321 | -0.1535896 | 2.616254 | 1 |
| 67 | y_N | 2.0222212 | 0.7698905 | 3.274552 | 1 |
| 67 | y_O | 0.96868382 | 0.1747278 | 1.76264 | 1 |
| 67 | y_P | 1.223641 | -0.8433944 | 3.290676 | 1 |
| 68 | y_A | 0.56253571 | -1.453087 | 2.578158 | 1 |
| 68 | y_B | 2.3881366 | -0.616241 | 5.392514 | 1 |
| 68 | y_C | 1.2813778 | -0.2296207 | 2.792376 | 1 |
| 68 | y_D | 0.86044497 | 0.2459513 | 1.474939 | 1 |
| 68 | y_E | 0.51181953 | -1.504129 | 2.527768 | 1 |
| 68 | y_F | 0.66630432 | -0.741189 | 2.073798 | 1 |
| 68 | y_G | 1.9443841 | -0.0674967 | 3.956265 | 1 |
| 68 | y_H | 1.2881416 | 0.665441 | 1.910842 | 1 |
| 68 | y_I | 1.1902415 | -0.7794578 | 3.159941 | 1 |
| 68 | y_J | 0.86166736 | -0.255185 | 1.97852 | 1 |
| 68 | y_L | 0.58809279 | -1.581317 | 2.757502 | 1 |
| 68 | y_M | 1.2325746 | -0.1507107 | 2.61586 | 1 |
| 68 | y_N | 2.0282949 | 0.7774339 | 3.279156 | 1 |
| 68 | y_O | 0.97144855 | 0.1796245 | 1.763273 | 1 |
| 68 | y_P | 1.2242691 | -0.8405926 | 3.289131 | 1 |
| 73 | y_A | 0.55825475 | -1.424243 | 2.540752 | 1 |
| 73 | y_B | 2.3838553 | -0.5727876 | 5.340498 | 1 |
| 73 | y_C | 1.272812 | -0.2136973 | 2.759321 | 1 |
| 73 | y_D | 0.7665431 | 0.1359631 | 1.397123 | 1 |
| 73 | y_E | 0.50753818 | -1.475291 | 2.490368 | 1 |
| 73 | y_F | 0.65493445 | -0.730039 | 2.039908 | 1 |
| 73 | y_G | 1.9142107 | -0.0626967 | 3.891118 | 1 |
| 73 | y_H | 1.2508361 | 0.6613806 | 1.840292 | 1 |
| 73 | y_I | 1.1787092 | -0.7591774 | 3.116596 | 1 |
| 73 | y_J | 0.83863543 | -0.2597569 | 1.937028 | 1 |
| 73 | y_L | 0.58809259 | -1.54597 | 2.722155 | 1 |
| 73 | y_M | 1.2216094 | -0.1398421 | 2.583061 | 1 |
| 73 | y_N | 2.0038525 | 0.7729143 | 3.234791 | 1 |
| 73 | y_O | 0.94501045 | 0.1647408 | 1.72528 | 1 |
| 73 | y_P | 1.2188201 | -0.8145467 | 3.252187 | 1 |
| 75 | y_A | 0.56223016 | -1.421624 | 2.546084 | 1 |
| 75 | y_B | 1.280749 | -0.2066984 | 2.768196 | 1 |
| 75 | y_C | 0.86018628 | 0.2552733 | 1.465099 | 1 |
| 75 | y_D | 0.5115214 | -1.472673 | 2.495716 | 1 |
| 75 | y_E | 0.66553415 | -0.7202268 | 2.051295 | 1 |
| 75 | y_F | 1.9217574 | -0.0564123 | 3.899927 | 1 |
| 75 | y_G | 1.2520215 | 0.6623111 | 1.841732 | 1 |
| 75 | y_H | 1.1856654 | -0.7534862 | 3.124817 | 1 |
| 75 | y_I | 0.85252056 | -0.2462874 | 1.951329 | 1 |
| 75 | y_K | 0.58809259 | -1.54743 | 2.723615 | 1 |
| 75 | y_L | 1.23198 | -0.130234 | 2.594194 | 1 |
| 75 | y_M | 2.021841 | 0.7905594 | 3.253123 | 1 |
| 75 | y_N | 0.96883934 | 0.1893787 | 1.7483 | 1 |
| 75 | y_O | 1.2240277 | -0.8106161 | 3.258672 | 1 |
| 76 | y_A | 0.5563812 | -1.379409 | 2.492172 | 1 |
| 76 | y_B | 2.3819813 | -0.507416 | 5.271379 | 1 |
| 76 | y_C | 1.2690513 | -0.1828461 | 2.720949 | 1 |
| 76 | y_D | 0.71927425 | 0.1031936 | 1.335355 | 1 |
| 76 | y_E | 0.50566356 | -1.430468 | 2.441795 | 1 |
| 76 | y_F | 0.65002094 | -0.7030271 | 2.003069 | 1 |
| 76 | y_G | 1.9066868 | -0.0249408 | 3.838314 | 1 |
| 76 | y_H | 1.2442279 | 0.6676682 | 1.820788 | 1 |
| 76 | y_I | 1.1745158 | -0.7186593 | 3.067691 | 1 |
| 76 | y_J | 0.83024732 | -0.2429011 | 1.903396 | 1 |
| 76 | y_L | 0.58809274 | -1.496159 | 2.672344 | 1 |
| 76 | y_M | 1.2170425 | -0.1134628 | 2.547548 | 1 |
| 76 | y_N | 1.9938136 | 0.7910814 | 3.196546 | 1 |
| 76 | y_O | 0.93264154 | 0.1704091 | 1.694874 | 1 |
| 76 | y_P | 1.2166137 | -0.7723956 | 3.205623 | 1 |
| 78 | y_A | 0.56223365 | -1.454528 | 2.578995 | 1 |
| 78 | y_B | 2.3878346 | -0.6181686 | 5.393838 | 1 |
| 78 | y_C | 1.2807744 | -0.2311621 | 2.792711 | 1 |
| 78 | y_D | 0.86016647 | 0.2452789 | 1.475054 | 1 |
| 78 | y_E | 0.51151755 | -1.50557 | 2.528605 | 1 |
| 78 | y_F | 0.66549565 | -0.7429715 | 2.073963 | 1 |
| 78 | y_G | 1.9231705 | -0.089444 | 3.935785 | 1 |
| 78 | y_H | 1.2559722 | 0.6568637 | 1.855081 | 1 |
| 78 | y_I | 1.1850839 | -0.7878018 | 3.157969 | 1 |
| 78 | y_J | 0.85137247 | -0.2808579 | 1.983603 | 1 |
| 78 | y_L | 0.5880928 | -1.58251 | 2.758695 | 1 |
| 78 | y_M | 1.2317775 | -0.1524536 | 2.616009 | 1 |
| 78 | y_N | 2.0263809 | 0.726741 | 3.326021 | 1 |
| 78 | y_O | 0.96966242 | 0.1755441 | 1.763781 | 1 |
| 78 | y_P | 1.2238668 | -0.842094 | 3.289828 | 1 |
| 80 | y_A | 0.55866273 | -1.450334 | 2.567659 | 1 |
| 80 | y_B | 2.384264 | -0.6105303 | 5.379058 | 1 |
| 80 | y_C | 1.2736354 | -0.2326593 | 2.77993 | 1 |
| 80 | y_D | 0.85689211 | 0.2442844 | 1.4695 | 1 |
| 80 | y_E | 0.50794699 | -1.501377 | 2.517271 | 1 |
| 80 | y_F | 0.65597431 | -0.7474192 | 2.059368 | 1 |
| 80 | y_G | 1.9614348 | -0.0465273 | 3.969397 | 1 |
| 80 | y_H | 1.2602944 | 0.6631722 | 1.857417 | 1 |
| 80 | y_I | 1.2212071 | -0.7467935 | 3.189208 | 1 |
| 80 | y_J | 0.9234754 | -0.2222976 | 2.069248 | 1 |
| 80 | y_L | 0.58809271 | -1.5742 | 2.750386 | 1 |
| 80 | y_M | 1.2224951 | -0.1568176 | 2.601808 | 1 |
| 80 | y_N | 2.1704422 | 0.796101 | 3.544783 | 1 |
| 80 | y_O | 0.94806951 | 0.1546968 | 1.741442 | 1 |
| 80 | y_P | 1.2192176 | -0.8393725 | 3.277808 | 1 |
| 84 | y_A | 0.57113328 | -1.458271 | 2.600538 | 1 |
| 84 | y_B | 2.3967322 | -0.6183912 | 5.411856 | 1 |
| 84 | y_C | 1.2985624 | -0.2759156 | 2.87304 | 1 |
| 84 | y_D | 0.85982665 | 0.2447248 | 1.474928 | 1 |
| 84 | y_E | 0.52041545 | -1.509309 | 2.550139 | 1 |
| 84 | y_F | 0.65401202 | -0.7809013 | 2.088925 | 1 |
| 84 | y_G | 1.9238546 | -0.0870596 | 3.934769 | 1 |
| 84 | y_H | 1.2561573 | 0.65698 | 1.855335 | 1 |
| 84 | y_I | 1.1856752 | -0.7857851 | 3.157135 | 1 |
| 84 | y_J | 0.8525537 | -0.2647029 | 1.96981 | 1 |
| 84 | y_L | 0.58809259 | -1.58338 | 2.759565 | 1 |
| 84 | y_M | 1.2235109 | -0.1746069 | 2.621629 | 1 |
| 84 | y_N | 2.0212995 | 0.7693222 | 3.273277 | 1 |
| 84 | y_O | 0.96746815 | 0.1740726 | 1.760864 | 1 |
| 84 | y_P | 1.2197126 | -0.84929 | 3.288715 | 1 |
| 87 | y_A | 0.56346002 | -1.450114 | 2.577034 | 1 |
| 87 | y_B | 2.3890603 | -0.6123545 | 5.390475 | 1 |
| 87 | y_C | 1.2832252 | -0.2263173 | 2.792768 | 1 |
| 87 | y_D | 0.89125141 | 0.2511539 | 1.531349 | 1 |
| 87 | y_E | 0.51274347 | -1.501157 | 2.526644 | 1 |
| 87 | y_F | 0.66877764 | -0.7374448 | 2.075 | 1 |
| 87 | y_G | 1.9265212 | -0.0805315 | 3.933574 | 1 |
| 87 | y_H | 1.2560704 | 0.6580013 | 1.85414 | 1 |
| 87 | y_I | 1.1883886 | -0.7792584 | 3.156036 | 1 |
| 87 | y_J | 0.85796848 | -0.2572266 | 1.973163 | 1 |
| 87 | y_L | 0.58809259 | -1.579117 | 2.755302 | 1 |
| 87 | y_M | 1.235006 | -0.1470482 | 2.61706 | 1 |
| 87 | y_N | 2.0283915 | 0.7786794 | 3.278104 | 1 |
| 87 | y_O | 0.97694916 | 0.1846813 | 1.769217 | 1 |
| 87 | y_P | 1.2254939 | -0.8374285 | 3.288416 | 1 |
| 89 | y_A | 0.52562122 | -1.398896 | 2.450138 | 1 |
| 89 | y_B | 2.351227 | -0.5215908 | 5.224045 | 1 |
| 89 | y_C | 1.2075747 | -0.2380992 | 2.653249 | 1 |
| 89 | y_D | 0.97723143 | 0.3746964 | 1.579767 | 1 |
| 89 | y_E | 0.47490916 | -1.449951 | 2.399769 | 1 |
| 89 | y_F | 0.56789255 | -0.781869 | 1.917654 | 1 |
| 89 | y_G | 1.8480993 | -0.0737969 | 3.769995 | 1 |
| 89 | y_H | 1.2348082 | 0.6613642 | 1.808252 | 1 |
| 89 | y_I | 1.1206202 | -0.7627708 | 3.004011 | 1 |
| 89 | y_J | 0.72268031 | -0.3542608 | 1.799621 | 1 |
| 89 | y_L | 0.58809259 | -1.483643 | 2.659828 | 1 |
| 89 | y_M | 1.1366604 | -0.190686 | 2.464007 | 1 |
| 89 | y_N | 1.8544649 | 0.6436263 | 3.065304 | 1 |
| 89 | y_O | 0.7480697 | -0.0509201 | 1.547059 | 1 |
| 89 | y_P | 1.1762418 | -0.8024123 | 3.154896 | 1 |
| 94 | y_A | 0.56274611 | -1.481367 | 2.606859 | 1 |
| 94 | y_B | 2.3883472 | -0.656999 | 5.433693 | 1 |
| 94 | y_C | 1.2818079 | -0.2508982 | 2.814514 | 1 |
| 94 | y_D | 0.86061631 | 0.2372151 | 1.484017 | 1 |
| 94 | y_E | 0.51203058 | -1.532403 | 2.556464 | 1 |
| 94 | y_F | 0.66681725 | -0.7613837 | 2.095018 | 1 |
| 94 | y_G | 1.9188352 | -0.1328553 | 3.970526 | 1 |
| 94 | y_H | 1.2583982 | 0.6508111 | 1.865985 | 1 |
| 94 | y_I | 1.1795179 | -0.8306503 | 3.189686 | 1 |
| 94 | y_J | 0.84027249 | -0.3821118 | 2.062657 | 1 |
| 94 | y_L | 0.58809259 | -1.611573 | 2.787758 | 1 |
| 94 | y_M | 1.2329394 | -0.1704348 | 2.636314 | 1 |
| 94 | y_N | 2.1183047 | 0.7248299 | 3.511779 | 1 |
| 94 | y_O | 0.9733536 | 0.158927 | 1.78778 | 1 |
| 94 | y_P | 1.2244028 | -0.8676793 | 3.316485 | 1 |
| 96 | disconnected |  |  |  | 0 |
| 100 | y_A | 0.56575757 | -1.450446 | 2.581962 | 1 |
| 100 | y_B | 2.3913578 | -0.6136575 | 5.396373 | 1 |
| 100 | y_C | 1.2878171 | -0.2248045 | 2.800439 | 1 |
| 100 | y_D | 0.86340538 | 0.2480952 | 1.478716 | 1 |
| 100 | y_E | 0.51504081 | -1.501489 | 2.531571 | 1 |
| 100 | y_F | 0.67490745 | -0.7354581 | 2.085273 | 1 |
| 100 | y_G | 1.9641454 | -0.0767504 | 4.005041 | 1 |
| 100 | y_H | 1.2614508 | 0.6607262 | 1.862175 | 1 |
| 100 | y_I | 0.92772897 | -0.3716594 | 2.227117 | 1 |
| 100 | y_K | 0.5880928 | -1.58166 | 2.757845 | 1 |
| 100 | y_L | 1.2409947 | -0.1450893 | 2.627079 | 1 |
| 100 | y_M | 2.0750745 | 0.7386224 | 3.411527 | 1 |
| 100 | y_N | 0.9907796 | 0.1753559 | 1.806203 | 1 |
| 100 | y_O | 1.2284982 | -0.837107 | 3.294103 | 1 |
| 102 | y_A | 0.55208742 | -1.455822 | 2.559997 | 1 |
| 102 | y_B | 2.3776899 | -0.6153924 | 5.370772 | 1 |
| 102 | y_C | 1.2604936 | -0.2458606 | 2.766848 | 1 |
| 102 | y_D | 0.85085179 | 0.2381056 | 1.463598 | 1 |
| 102 | y_E | 0.50137284 | -1.506864 | 2.50961 | 1 |
| 102 | y_F | 0.63841762 | -0.7660306 | 2.042866 | 1 |
| 102 | y_G | 1.3021049 | 0.6883018 | 1.915908 | 1 |
| 102 | y_H | 1.0826248 | -0.9044508 | 3.0697 | 1 |
| 102 | y_I | 0.64685067 | -0.6302388 | 1.92394 | 1 |
| 102 | y_K | 0.58809271 | -1.572839 | 2.749025 | 1 |
| 102 | y_L | 1.2053142 | -0.175035 | 2.585663 | 1 |
| 102 | y_M | 1.8761868 | 0.5533786 | 3.198995 | 1 |
| 102 | y_N | 0.90860763 | 0.0987843 | 1.718431 | 1 |
| 102 | y_O | 1.2105892 | -0.8471059 | 3.268284 | 1 |
| 103 | y_A | 0.56547972 | -1.481591 | 2.61255 | 1 |
| 103 | y_B | 2.3910803 | -0.6585246 | 5.440685 | 1 |
| 103 | y_C | 1.2872724 | -0.2476657 | 2.822211 | 1 |
| 103 | y_D | 0.86312725 | 0.2388131 | 1.487441 | 1 |
| 103 | y_E | 0.51476379 | -1.532628 | 2.562155 | 1 |
| 103 | y_F | 0.67411174 | -0.7561935 | 2.104417 | 1 |
| 103 | y_G | 1.8893941 | -0.1661554 | 3.944944 | 1 |
| 103 | y_H | 1.2547201 | 0.6462355 | 1.863205 | 1 |
| 103 | y_I | 1.1519028 | -0.8619658 | 3.165771 | 1 |
| 103 | y_J | 0.78514713 | -0.444555 | 2.014849 | 1 |
| 103 | y_L | 0.58809259 | -1.61472 | 2.790905 | 1 |
| 103 | y_M | 1.2400647 | -0.1653513 | 2.645481 | 1 |
| 103 | y_N | 2.037926 | 0.6343624 | 3.44149 | 1 |
| 103 | y_O | 0.98981983 | 0.1734941 | 1.806146 | 1 |
| 103 | y_P | 1.2279765 | -0.8669301 | 3.322883 | 1 |
| 104 | y_A | 0.70264205 | -1.307669 | 2.712953 | 1 |
| 104 | y_B | 2.5282149 | -0.4517181 | 5.508148 | 1 |
| 104 | y_C | 1.5613826 | -0.0422699 | 3.165035 | 1 |
| 104 | y_D | 0.87128526 | 0.264533 | 1.478037 | 1 |
| 104 | y_E | 0.65189875 | -1.358732 | 2.662529 | 1 |
| 104 | y_F | 1.040514 | -0.5570597 | 2.638088 | 1 |
| 104 | y_G | 1.9447776 | -0.0384562 | 3.928011 | 1 |
| 104 | y_H | 1.2556855 | 0.6645499 | 1.846821 | 1 |
| 104 | y_I | 1.2068601 | -0.7372133 | 3.150934 | 1 |
| 104 | y_J | 0.89482786 | -0.2100596 | 1.999715 | 1 |
| 104 | y_L | 0.58809251 | -1.552343 | 2.728528 | 1 |
| 104 | y_M | 1.7400385 | 0.0056991 | 3.474378 | 1 |
| 104 | y_N | 2.0766428 | 0.8372051 | 3.316081 | 1 |
| 104 | y_O | 1.0414249 | 0.2453898 | 1.83746 | 1 |
| 106 | y_A | 0.28788188 | -1.743068 | 2.318832 | 1 |
| 106 | y_B | 2.1135346 | -0.8809683 | 5.108037 | 1 |
| 106 | y_C | 0.73245433 | -0.9686816 | 2.43359 | 1 |
| 106 | y_D | 0.88792162 | 0.2800794 | 1.495764 | 1 |
| 106 | y_E | 0.2372158 | -1.794043 | 2.268475 | 1 |
| 106 | y_F | -0.06711557 | -1.835116 | 1.700885 | 1 |
| 106 | y_G | 1.9787616 | -0.0063429 | 3.963866 | 1 |
| 106 | y_H | 1.260398 | 0.6688536 | 1.851942 | 1 |
| 106 | y_I | 1.2385013 | -0.7072983 | 3.184301 | 1 |
| 106 | y_J | 0.95798485 | -0.1540263 | 2.069996 | 1 |
| 106 | y_L | 0.58809259 | -1.5532 | 2.729385 | 1 |
| 106 | y_M | 0.51332312 | -1.213622 | 2.240269 | 1 |
| 106 | y_N | 2.1585584 | 0.9086154 | 3.408501 | 1 |
| 106 | y_O | 1.1500423 | 0.326611 | 1.973474 | 1 |

Table S15. Sensitivity Analysis for BI

| **dropped_id** | **comparison** | **effect size** | **lower confidence interval** | **upper confidence interval** | **Connected Network Status** |
| --- | --- | --- | --- | --- | --- |
| 6 | y_A | 0.49160457 | -0.2350443 | 1.218253 | 1 |
| 6 | y_B | 0.98083576 | 0.2189436 | 1.742728 | 1 |
| 6 | y_C | 1.2973856 | 0.4993815 | 2.09539 | 1 |
| 6 | y_D | 0.60086847 | 0.2109475 | 0.9907894 | 1 |
| 6 | y_E | 0.5999837 | 0.1843966 | 1.015571 | 1 |
| 6 | y_F | 0.54720649 | -0.2305929 | 1.325006 | 1 |
| 6 | y_H | 0.91336079 | 0.1501017 | 1.67662 | 1 |
| 6 | y_I | 0.0109777 | -0.7383908 | 0.7603462 | 1 |
| 6 | y_J | 1.4982019 | 0.3748199 | 2.621584 | 1 |
| 6 | y_K | 0.97822658 | 0.6229511 | 1.333502 | 1 |
| 7 | y_A | 0.49160457 | -0.2352389 | 1.218448 | 1 |
| 7 | y_B | 0.98083576 | 0.2187579 | 1.742914 | 1 |
| 7 | y_C | 1.2973856 | 0.4992042 | 2.095567 | 1 |
| 7 | y_D | 0.60087887 | 0.210862 | 0.9908957 | 1 |
| 7 | y_E | 0.59813539 | 0.183603 | 1.012668 | 1 |
| 7 | y_F | 0.5472065 | -0.2307747 | 1.325188 | 1 |
| 7 | y_H | 0.9133608 | 0.1499164 | 1.676805 | 1 |
| 7 | y_I | 0.01097771 | -0.7385796 | 0.7605349 | 1 |
| 7 | y_J | 1.4982019 | 0.3745681 | 2.621836 | 1 |
| 7 | y_K | 0.97823529 | 0.6228763 | 1.333594 | 1 |
| 8 | y_A | 0.49160457 | 0.0020881 | 0.9811211 | 1 |
| 8 | y_B | 0.98083576 | 0.4403845 | 1.521287 | 1 |
| 8 | y_C | 1.297386 | 0.707116 | 1.887656 | 1 |
| 8 | y_D | 0.57818888 | 0.3022333 | 0.8541445 | 1 |
| 8 | y_E | 0.34822771 | 0.0842034 | 0.6122521 | 1 |
| 8 | y_F | 0.54720726 | -0.0154475 | 1.109862 | 1 |
| 8 | y_H | 0.91336079 | 0.3709843 | 1.455737 | 1 |
| 8 | y_I | 0.0109777 | -0.5116704 | 0.5336258 | 1 |
| 8 | y_J | 1.498203 | 0.6704323 | 2.325974 | 1 |
| 8 | y_K | 0.96092426 | 0.7037368 | 1.218112 | 1 |
| 9 | y_A | 0.49160457 | -0.1965476 | 1.179757 | 1 |
| 9 | y_B | 1.2973857 | 0.53427 | 2.060501 | 1 |
| 9 | y_C | 0.59865765 | 0.2277825 | 0.9695328 | 1 |
| 9 | y_D | 0.53991515 | 0.1989122 | 0.8809181 | 1 |
| 9 | y_E | 0.54720664 | -0.1947549 | 1.289168 | 1 |
| 9 | y_G | 0.91336079 | 0.1866564 | 1.640065 | 1 |
| 9 | y_H | 0.0109777 | -0.7011235 | 0.7230788 | 1 |
| 9 | y_I | 1.4982021 | 0.4243971 | 2.572007 | 1 |
| 9 | y_J | 0.9764051 | 0.6376684 | 1.315142 | 1 |
| 10 | y_A | 0.49160457 | -0.2206924 | 1.203902 | 1 |
| 10 | y_B | 0.98083576 | 0.2326191 | 1.729052 | 1 |
| 10 | y_C | 1.2973857 | 0.5124276 | 2.082344 | 1 |
| 10 | y_D | 0.60008478 | 0.2172272 | 0.9829424 | 1 |
| 10 | y_E | 0.62970766 | 0.2118292 | 1.047586 | 1 |
| 10 | y_F | 0.54720655 | -0.217202 | 1.311615 | 1 |
| 10 | y_H | 0.91336079 | 0.1637523 | 1.662969 | 1 |
| 10 | y_I | 0.0109777 | -0.7244825 | 0.7464379 | 1 |
| 10 | y_J | 1.498202 | 0.3933562 | 2.603048 | 1 |
| 10 | y_K | 0.97757732 | 0.628449 | 1.326706 | 1 |
| 11 | y_A | 0.49160457 | -0.106081 | 1.08929 | 1 |
| 11 | y_B | 0.98083576 | 0.340766 | 1.620906 | 1 |
| 11 | y_C | 1.2973859 | 0.6147291 | 1.980043 | 1 |
| 11 | y_D | 0.557333 | 0.2291018 | 0.8855641 | 1 |
| 11 | y_E | 0.53202559 | 0.2363411 | 0.8277101 | 1 |
| 11 | y_F | 0.54720695 | -0.1117178 | 1.206132 | 1 |
| 11 | y_H | 0.91336079 | 0.2716647 | 1.555057 | 1 |
| 11 | y_I | 0.0109777 | -0.6141325 | 0.6360878 | 1 |
| 11 | y_J | 1.4982026 | 0.538824 | 2.457581 | 1 |
| 11 | y_K | 0.82513556 | 0.4897813 | 1.16049 | 1 |
| 12 | y_A | 0.49160457 | -0.2151269 | 1.198336 | 1 |
| 12 | y_B | 0.98083576 | 0.2379155 | 1.723756 | 1 |
| 12 | y_C | 1.2973857 | 0.5174745 | 2.077297 | 1 |
| 12 | y_D | 0.61811827 | 0.2353362 | 1.0009 | 1 |
| 12 | y_E | 0.54116289 | 0.1907153 | 0.8916105 | 1 |
| 12 | y_F | 0.54720657 | -0.2120185 | 1.306432 | 1 |
| 12 | y_H | 0.91336079 | 0.1690389 | 1.657683 | 1 |
| 12 | y_I | 0.0109777 | -0.7190935 | 0.7410489 | 1 |
| 12 | y_J | 1.498202 | 0.4005277 | 2.595876 | 1 |
| 12 | y_K | 1.0535246 | 0.659771 | 1.447278 | 1 |
| 13 | y_A | 0.49160457 | -0.2294513 | 1.21266 | 1 |
| 13 | y_B | 0.98083576 | 0.224276 | 1.737396 | 1 |
| 13 | y_C | 1.2973857 | 0.504471 | 2.0903 | 1 |
| 13 | y_D | 0.55618155 | 0.1137377 | 0.9986255 | 1 |
| 13 | y_E | 0.54205833 | 0.1843835 | 0.8997332 | 1 |
| 13 | y_F | 0.54720652 | -0.2253702 | 1.319783 | 1 |
| 13 | y_H | 0.9133608 | 0.1554246 | 1.671297 | 1 |
| 13 | y_I | 0.0109777 | -0.7329686 | 0.7549241 | 1 |
| 13 | y_J | 1.4982019 | 0.3820509 | 2.614353 | 1 |
| 13 | y_K | 0.96907845 | 0.6135985 | 1.324558 | 1 |
| 14 | y_A | 0.49160457 | -0.2396626 | 1.222872 | 1 |
| 14 | y_B | 0.98083576 | 0.2145376 | 1.747134 | 1 |
| 14 | y_C | 1.2973856 | 0.4951738 | 2.099597 | 1 |
| 14 | y_D | 0.59043772 | 0.1374597 | 1.043416 | 1 |
| 14 | y_E | 0.54266452 | 0.1798507 | 0.9054784 | 1 |
| 14 | y_F | 0.54720648 | -0.2349092 | 1.329322 | 1 |
| 14 | y_H | 0.91336079 | 0.1457036 | 1.681018 | 1 |
| 14 | y_I | 0.0109777 | -0.74287 | 0.7648254 | 1 |
| 14 | y_J | 1.4982019 | 0.3688421 | 2.627562 | 1 |
| 14 | y_K | 0.97628858 | 0.6160993 | 1.336478 | 1 |
| 15 | y_A | 0.49160457 | -0.1965475 | 1.179757 | 1 |
| 15 | y_B | 0.98083576 | 0.2555673 | 1.706104 | 1 |
| 15 | y_C | 1.2973857 | 0.5342702 | 2.060501 | 1 |
| 15 | y_D | 0.59865764 | 0.2277824 | 0.9695328 | 1 |
| 15 | y_E | 0.53991514 | 0.1989121 | 0.8809182 | 1 |
| 15 | y_F | 0.54720664 | -0.1947547 | 1.289168 | 1 |
| 15 | y_H | 0.0109777 | -0.7011233 | 0.7230787 | 1 |
| 15 | y_I | 1.4982021 | 0.4243973 | 2.572007 | 1 |
| 15 | y_J | 0.97640509 | 0.6376684 | 1.315142 | 1 |
| 19 | y_A | 0.49160457 | -0.1811554 | 1.164364 | 1 |
| 19 | y_B | 0.98083576 | 0.270155 | 1.691517 | 1 |
| 19 | y_C | 1.2973857 | 0.5481207 | 2.046651 | 1 |
| 19 | y_D | 0.62402812 | 0.2581364 | 0.9899199 | 1 |
| 19 | y_E | 0.53880028 | 0.20547 | 0.8721305 | 1 |
| 19 | y_F | 0.54720669 | -0.1805017 | 1.274915 | 1 |
| 19 | y_H | 0.91336079 | 0.2012149 | 1.625507 | 1 |
| 19 | y_I | 0.0109777 | -0.6862601 | 0.7082155 | 1 |
| 19 | y_J | 1.4982022 | 0.4440853 | 2.552319 | 1 |
| 19 | y_K | 1.0856073 | 0.7100484 | 1.461166 | 1 |
| 20 | y_A | 0.49160457 | -0.2137192 | 1.196928 | 1 |
| 20 | y_B | 0.98083576 | 0.2392545 | 1.722417 | 1 |
| 20 | y_C | 1.2973857 | 0.5187499 | 2.076021 | 1 |
| 20 | y_D | 0.58484013 | 0.2032676 | 0.9664127 | 1 |
| 20 | y_E | 0.54107178 | 0.1913187 | 0.8908249 | 1 |
| 20 | y_F | 0.54720658 | -0.2107083 | 1.305121 | 1 |
| 20 | y_H | 0.9133608 | 0.1703753 | 1.656346 | 1 |
| 20 | y_I | 0.0109777 | -0.7177309 | 0.7396863 | 1 |
| 20 | y_J | 1.498202 | 0.4023401 | 2.594064 | 1 |
| 20 | y_K | 0.91557767 | 0.5297381 | 1.301417 | 1 |
| 21 | y_A | 0.98083576 | 0.2555672 | 1.706104 | 1 |
| 21 | y_B | 1.2973857 | 0.53427 | 2.060501 | 1 |
| 21 | y_C | 0.59865764 | 0.2277824 | 0.9695329 | 1 |
| 21 | y_D | 0.53991514 | 0.198912 | 0.8809183 | 1 |
| 21 | y_E | 0.54720664 | -0.1947548 | 1.289168 | 1 |
| 21 | y_G | 0.91336079 | 0.1866565 | 1.640065 | 1 |
| 21 | y_H | 0.0109777 | -0.7011234 | 0.7230788 | 1 |
| 21 | y_I | 1.4982021 | 0.4243971 | 2.572007 | 1 |
| 21 | y_J | 0.9764051 | 0.6376683 | 1.315142 | 1 |
| 22 | y_A | 0.49160457 | -0.1965476 | 1.179757 | 1 |
| 22 | y_B | 0.98083576 | 0.2555672 | 1.706104 | 1 |
| 22 | y_C | 1.2973857 | 0.53427 | 2.060501 | 1 |
| 22 | y_D | 0.59865764 | 0.2277825 | 0.9695327 | 1 |
| 22 | y_E | 0.53991514 | 0.1989122 | 0.8809181 | 1 |
| 22 | y_F | 0.54720664 | -0.1947548 | 1.289168 | 1 |
| 22 | y_H | 0.91336079 | 0.1866565 | 1.640065 | 1 |
| 22 | y_I | 1.4982021 | 0.4243971 | 2.572007 | 1 |
| 22 | y_J | 0.9764051 | 0.6376684 | 1.315142 | 1 |
| 23 | y_A | 0.49160457 | -0.1965476 | 1.179757 | 1 |
| 23 | y_B | 0.98083576 | 0.2555671 | 1.706104 | 1 |
| 23 | y_C | 1.2973865 | 0.5342706 | 2.060502 | 1 |
| 23 | y_D | 0.59865766 | 0.2277826 | 0.9695327 | 1 |
| 23 | y_E | 0.53991516 | 0.1989123 | 0.880918 | 1 |
| 23 | y_F | 0.5472081 | -0.1947539 | 1.28917 | 1 |
| 23 | y_H | 0.91336079 | 0.1866564 | 1.640065 | 1 |
| 23 | y_I | 0.0109777 | -0.7011234 | 0.7230788 | 1 |
| 23 | y_J | 0.97640511 | 0.6376685 | 1.315142 | 1 |
| 27 | disconnected |  |  |  | 0 |

Table S16. Sensitivity Analysis for TER

| **Dropped_id** | **comparison** | **log_eff** | **log_lci** | **log_uci** | **OR** | **OR_lci** | **OR_uci** | **connected** |
| --- | --- | --- | --- | --- | --- | --- | --- | --- |
| 4 | y_A | 1.856298 | 0.535417 | 3.177179 | 6.4 | 1.70816 | 23.97901 | 1 |
| 4 | y_B | 3.5302684 | 1.357341 | 5.703196 | 34.133126 | 3.885848 | 299.824 | 1 |
| 4 | y_C | 1.5533484 | -0.0640305 | 3.170727 | 4.7272727 | 0.9379764 | 23.82481 | 1 |
| 4 | y_D | 1.1773254 | 0.7200882 | 1.634563 | 3.2456818 | 2.054615 | 5.127215 | 1 |
| 4 | y_E | 2.315069 | 0.5298631 | 4.100275 | 10.125622 | 1.6987 | 60.35688 | 1 |
| 4 | y_F | 3.8489981 | 1.510292 | 6.187705 | 46.946004 | 4.528051 | 486.7276 | 1 |
| 4 | y_G | 3.5850621 | 1.659306 | 5.510818 | 36.055595 | 5.255663 | 247.3533 | 1 |
| 4 | y_H | 1.5901355 | 0.8654588 | 2.314812 | 4.9044132 | 2.376096 | 10.12302 | 1 |
| 4 | y_I | 2.8540647 | 0.709987 | 4.998143 | 17.358195 | 2.033965 | 148.1377 | 1 |
| 4 | y_K | 1.4136933 | 0.0365319 | 2.790855 | 4.1111111 | 1.037207 | 16.29494 | 1 |
| 4 | y_L | 1.6436293 | 0.5162063 | 2.771052 | 5.173913 | 1.675659 | 15.97544 | 1 |
| 4 | y_M | 3.0183782 | 1.581565 | 4.455192 | 20.458085 | 4.862559 | 86.07263 | 1 |
| 4 | y_N | 4.9609058 | 2.229316 | 7.692496 | 142.72301 | 9.293505 | 2191.838 | 1 |
| 4 | y_O | 1.6684657 | 0.9336178 | 2.403314 | 5.3040235 | 2.543695 | 11.05976 | 1 |
| 6 | y_A | 1.856298 | 0.535417 | 3.177179 | 6.4 | 1.70816 | 23.97901 | 1 |
| 6 | y_B | 1.5533484 | -0.0640305 | 3.170727 | 4.7272727 | 0.9379764 | 23.82481 | 1 |
| 6 | y_C | 1.2356999 | 0.7957069 | 1.675693 | 3.440786 | 2.216007 | 5.342496 | 1 |
| 6 | y_D | 2.3294648 | 0.5445213 | 4.114408 | 10.272442 | 1.723783 | 61.21598 | 1 |

| 6 | y_E | 3.8633939 | 1.524888 | 6.2019 | 47.626718 | 4.594628 | 493.6862 | 1 |
| --- | --- | --- | --- | --- | --- | --- | --- | --- |
| 6 | y_F | 3.5994579 | 1.673945 | 5.524971 | 36.578399 | 5.333168 | 250.8789 | 1 |
| 6 | y_G | 1.5901355 | 0.8654588 | 2.314812 | 4.9044132 | 2.376096 | 10.12302 | 1 |
| 6 | y_H | 2.8684605 | 0.7246013 | 5.01232 | 17.609888 | 2.063908 | 150.2529 | 1 |
| 6 | y_J | 1.4136933 | 0.0365319 | 2.790855 | 4.1111111 | 1.037207 | 16.29494 | 1 |
| 6 | y_K | 1.6436293 | 0.5162063 | 2.771052 | 5.173913 | 1.675659 | 15.97544 | 1 |
| 6 | y_L | 3.0327728 | 1.596286 | 4.469259 | 20.754702 | 4.934672 | 87.29205 | 1 |
| 6 | y_M | 4.9753016 | 2.243883 | 7.70672 | 144.79249 | 9.429876 | 2223.239 | 1 |
| 6 | y_N | 1.6828588 | 0.9486511 | 2.417067 | 5.3809168 | 2.582224 | 11.21292 | 1 |
| 9 | y_A | 1.856298 | 0.535417 | 3.177179 | 6.4 | 1.70816 | 23.97901 | 1 |
| 9 | y_B | 3.5446607 | 1.37195 | 5.717371 | 34.627935 | 3.943032 | 304.1045 | 1 |
| 9 | y_C | 1.5533484 | -0.0640305 | 3.170727 | 4.7272727 | 0.9379764 | 23.82481 | 1 |
| 9 | y_D | 1.2356999 | 0.7957068 | 1.675693 | 3.4407858 | 2.216007 | 5.342496 | 1 |
| 9 | y_E | 2.3294614 | 0.5445189 | 4.114404 | 10.272407 | 1.723779 | 61.21571 | 1 |
| 9 | y_F | 3.8633905 | 1.524885 | 6.201896 | 47.626554 | 4.594616 | 493.6841 | 1 |
| 9 | y_G | 3.5994544 | 1.673943 | 5.524966 | 36.578273 | 5.333155 | 250.8778 | 1 |
| 9 | y_H | 1.751062 | 0.4368526 | 3.065271 | 5.7607171 | 1.547828 | 21.44028 | 1 |
| 9 | y_I | 2.8684571 | 0.7245988 | 5.012315 | 17.609827 | 2.063903 | 150.2522 | 1 |
| 9 | y_K | 1.4136933 | 0.0365319 | 2.790855 | 4.1111111 | 1.037207 | 16.29494 | 1 |
| 9 | y_L | 1.6436293 | 0.5162063 | 2.771052 | 5.173913 | 1.675659 | 15.97544 | 1 |
| 9 | y_M | 3.0327706 | 1.596285 | 4.469256 | 20.754656 | 4.934664 | 87.2918 | 1 |
| 9 | y_N | 4.9752982 | 2.24388 | 7.706716 | 144.79199 | 9.429851 | 2223.229 | 1 |
| 9 | y_O | 1.6828582 | 0.9486506 | 2.417066 | 5.3809137 | 2.582223 | 11.21291 | 1 |
| 12 | y_A | 1.856298 | 0.535417 | 3.177179 | 6.4 | 1.70816 | 23.97901 | 1 |
| 12 | y_B | 3.3612521 | 1.151852 | 5.570652 | 28.82526 | 3.164046 | 262.6054 | 1 |
| 12 | y_C | 1.5533484 | -0.0640305 | 3.170727 | 4.7272727 | 0.9379764 | 23.82481 | 1 |
| 12 | y_D | 1.2901495 | 0.8343381 | 1.745961 | 3.6333298 | 2.303289 | 5.731407 | 1 |
| 12 | y_E | 2.1460527 | 0.3166272 | 3.975478 | 8.5510383 | 1.372491 | 53.27559 | 1 |
| 12 | y_F | 3.6799819 | 1.307349 | 6.052614 | 39.645676 | 3.696363 | 425.2233 | 1 |
| 12 | y_G | 3.4160458 | 1.449228 | 5.382864 | 30.448776 | 4.259824 | 217.6447 | 1 |
| 12 | y_H | 1.5901355 | 0.8654588 | 2.314812 | 4.9044132 | 2.376096 | 10.12302 | 1 |
| 12 | y_I | 2.6850485 | 0.5040151 | 4.866082 | 14.658912 | 1.655354 | 129.8113 | 1 |
| 12 | y_K | 1.4136933 | 0.0365319 | 2.790855 | 4.1111111 | 1.037207 | 16.29494 | 1 |
| 12 | y_L | 1.6436293 | 0.5162063 | 2.771052 | 5.173913 | 1.675659 | 15.97544 | 1 |
| 12 | y_M | 2.8493613 | 1.357962 | 4.34076 | 17.276744 | 3.888262 | 76.76589 | 1 |
| 12 | y_N | 4.7918895 | 2.031197 | 7.552582 | 120.52889 | 7.623208 | 1905.656 | 1 |
| 12 | y_O | 1.499448 | 0.6628825 | 2.336014 | 4.4792157 | 1.940377 | 10.33993 | 1 |
| 13 | disconnected |  |  |  |  |  |  | 0 |
| 14 | y_A | 1.856298 | 0.535417 | 3.177179 | 6.4 | 1.70816 | 23.97901 | 1 |
| 14 | y_B | 3.5446607 | 1.37195 | 5.717371 | 34.627935 | 3.943032 | 304.1045 | 1 |
| 14 | y_C | 1.5533484 | -0.0640305 | 3.170727 | 4.7272727 | 0.9379764 | 23.82481 | 1 |
| 14 | y_D | 1.2356999 | 0.7957068 | 1.675693 | 3.4407858 | 2.216007 | 5.342496 | 1 |
| 14 | y_E | 2.3294614 | 0.5445189 | 4.114404 | 10.272407 | 1.723779 | 61.21571 | 1 |
| 14 | y_F | 3.8633905 | 1.524885 | 6.201896 | 47.626554 | 4.594616 | 493.6841 | 1 |
| 14 | y_G | 3.5994544 | 1.673943 | 5.524966 | 36.578273 | 5.333154 | 250.8778 | 1 |
| 14 | y_H | 1.5811764 | 0.7741932 | 2.38816 | 4.8606707 | 2.168842 | 10.89343 | 1 |
| 14 | y_I | 2.8684571 | 0.7245988 | 5.012315 | 17.609827 | 2.063903 | 150.2522 | 1 |
| 14 | y_K | 1.4136933 | 0.0365319 | 2.790855 | 4.1111111 | 1.037207 | 16.29494 | 1 |
| 14 | y_L | 1.6436293 | 0.5162063 | 2.771052 | 5.173913 | 1.675659 | 15.97544 | 1 |
| 19 | y_A | 1.856298 | 0.535417 | 3.177179 | 6.4 | 1.70816 | 23.97901 | 1 |
| 19 | y_B | 3.5552605 | 1.382158 | 5.728363 | 34.996936 | 3.98349 | 307.4654 | 1 |
| 19 | y_C | 1.5533484 | -0.0640305 | 3.170727 | 4.7272727 | 0.9379764 | 23.82481 | 1 |
| 19 | y_D | 1.2786919 | 0.8079724 | 1.749411 | 3.591938 | 2.243355 | 5.751217 | 1 |
| 19 | y_E | 2.3400612 | 0.5546423 | 4.12548 | 10.381872 | 1.741318 | 61.89752 | 1 |
| 19 | y_F | 3.8739903 | 1.535121 | 6.212859 | 48.134071 | 4.641888 | 499.1263 | 1 |
| 19 | y_G | 3.6100542 | 1.684101 | 5.536007 | 36.968058 | 5.387606 | 253.6632 | 1 |
| 19 | y_H | 1.5901355 | 0.8654588 | 2.314812 | 4.9044132 | 2.376096 | 10.12302 | 1 |
| 19 | y_I | 2.8790569 | 0.7348018 | 5.023312 | 17.79748 | 2.085069 | 151.9136 | 1 |
| 19 | y_K | 1.4136933 | 0.0365319 | 2.790855 | 4.1111111 | 1.037207 | 16.29494 | 1 |
| 19 | y_L | 1.6436293 | 0.5162063 | 2.771052 | 5.173913 | 1.675659 | 15.97544 | 1 |
| 19 | y_M | 3.0433704 | 1.606292 | 4.480448 | 20.975822 | 4.984297 | 88.27425 | 1 |
| 19 | y_N | 4.9858979 | 2.254169 | 7.717627 | 146.33492 | 9.527369 | 2247.62 | 1 |
| 19 | y_O | 1.6934581 | 0.9580929 | 2.428823 | 5.4382541 | 2.606721 | 11.34552 | 1 |
| 21 | y_A | 1.856298 | 0.535417 | 3.177179 | 6.4 | 1.70816 | 23.97901 | 1 |
| 21 | y_B | 3.5446641 | 1.371952 | 5.717376 | 34.62805 | 3.943042 | 304.1058 | 1 |
| 21 | y_C | 1.5533484 | -0.0640305 | 3.170727 | 4.7272727 | 0.9379764 | 23.82481 | 1 |
| 21 | y_D | 1.2356999 | 0.7957069 | 1.675693 | 3.440786 | 2.216007 | 5.342496 | 1 |
| 21 | y_E | 2.3294647 | 0.5445212 | 4.114408 | 10.272441 | 1.723783 | 61.21598 | 1 |
| 21 | y_F | 3.8633938 | 1.524888 | 6.2019 | 47.626713 | 4.594627 | 493.6861 | 1 |
| 21 | y_G | 3.480987 | 1.538894 | 5.42308 | 32.491776 | 4.659435 | 226.5759 | 1 |
| 21 | y_H | 1.5901355 | 0.8654588 | 2.314812 | 4.9044132 | 2.376096 | 10.12302 | 1 |
| 21 | y_I | 2.8684604 | 0.7246011 | 5.01232 | 17.609886 | 2.063908 | 150.2529 | 1 |
| 21 | y_K | 1.4136933 | 0.0365319 | 2.790855 | 4.1111111 | 1.037207 | 16.29494 | 1 |
| 21 | y_L | 1.6436293 | 0.5162063 | 2.771052 | 5.173913 | 1.675659 | 15.97544 | 1 |
| 21 | y_M | 3.0327727 | 1.596286 | 4.469259 | 20.7547 | 4.934672 | 87.29205 | 1 |
| 22 | y_C | 1.5533484 | -0.0640305 | 3.170727 | 4.7272727 | 0.9379764 | 23.82481 | 1 |
| 22 | y_D | 1.2356999 | 0.7957068 | 1.675693 | 3.4407858 | 2.216007 | 5.342496 | 1 |
| 22 | y_E | 2.3294614 | 0.5445188 | 4.114404 | 10.272407 | 1.723779 | 61.21571 | 1 |
| 22 | y_F | 3.8633905 | 1.524885 | 6.201896 | 47.626554 | 4.594615 | 493.6841 | 1 |
| 22 | y_G | 3.5994544 | 1.673943 | 5.524966 | 36.578273 | 5.333154 | 250.8778 | 1 |
| 22 | y_H | 1.543249 | 0.7748874 | 2.31161 | 4.67977 | 2.170348 | 10.09066 | 1 |
| 22 | y_I | 2.8684571 | 0.7245987 | 5.012315 | 17.609827 | 2.063903 | 150.2522 | 1 |
| 22 | y_K | 1.4136933 | 0.0365319 | 2.790855 | 4.1111111 | 1.037207 | 16.29494 | 1 |
| 22 | y_L | 1.6436293 | 0.5162063 | 2.771052 | 5.173913 | 1.675659 | 15.97544 | 1 |
| 22 | y_M | 3.0327706 | 1.596285 | 4.469256 | 20.754656 | 4.934664 | 87.29181 | 1 |
| 22 | y_N | 4.9752982 | 2.24388 | 7.706716 | 144.79199 | 9.429851 | 2223.229 | 1 |
| 22 | y_O | 1.6828582 | 0.9486506 | 2.417066 | 5.3809137 | 2.582223 | 11.21291 | 1 |
| 26 | y_A | 1.856298 | 0.535417 | 3.177179 | 6.4 | 1.70816 | 23.97901 | 1 |
| 26 | y_B | 3.5446651 | 1.371953 | 5.717377 | 34.628087 | 3.943044 | 304.1062 | 1 |
| 26 | y_C | 1.5533484 | -0.0640305 | 3.170727 | 4.7272727 | 0.9379764 | 23.82481 | 1 |
| 26 | y_D | 1.2356999 | 0.7957069 | 1.675693 | 3.440786 | 2.216007 | 5.342496 | 1 |
| 26 | y_E | 2.3294658 | 0.5445218 | 4.11441 | 10.272452 | 1.723784 | 61.21607 | 1 |
| 26 | y_F | 3.8633949 | 1.524888 | 6.201901 | 47.626763 | 4.594631 | 493.6868 | 1 |
| 26 | y_G | 3.5931784 | 1.551396 | 5.634961 | 36.349425 | 4.718053 | 280.0479 | 1 |
| 26 | y_H | 1.5901355 | 0.8654588 | 2.314812 | 4.9044132 | 2.376096 | 10.12302 | 1 |
| 26 | y_I | 2.8684615 | 0.7246019 | 5.012321 | 17.609904 | 2.063909 | 150.2531 | 1 |
| 26 | y_K | 1.4136933 | 0.0365319 | 2.790855 | 4.1111111 | 1.037207 | 16.29494 | 1 |
| 26 | y_L | 1.6436293 | 0.5162063 | 2.771052 | 5.173913 | 1.675659 | 15.97544 | 1 |
| 26 | y_M | 3.0327734 | 1.596287 | 4.46926 | 20.754714 | 4.934674 | 87.29212 | 1 |
| 26 | y_N | 4.9753025 | 2.243884 | 7.706721 | 144.79262 | 9.429883 | 2223.241 | 1 |
| 26 | y_O | 1.6828589 | 0.9486512 | 2.417067 | 5.3809177 | 2.582224 | 11.21292 | 1 |
| 27 | y_A | 1.856298 | 0.535417 | 3.177179 | 6.4 | 1.70816 | 23.97901 | 1 |
| 27 | y_B | 3.5446635 | 1.371952 | 5.717375 | 34.628029 | 3.94304 | 304.1056 | 1 |
| 27 | y_C | 1.5533484 | -0.0640305 | 3.170727 | 4.7272727 | 0.9379764 | 23.82481 | 1 |
| 27 | y_D | 1.2356999 | 0.7957069 | 1.675693 | 3.440786 | 2.216007 | 5.342496 | 1 |
| 27 | y_E | 2.3294641 | 0.5445207 | 4.114408 | 10.272435 | 1.723782 | 61.21593 | 1 |
| 27 | y_F | 3.8633932 | 1.524887 | 6.201899 | 47.626684 | 4.594625 | 493.6858 | 1 |
| 27 | y_G | 3.7874864 | 1.765818 | 5.809155 | 44.145296 | 5.846353 | 333.3372 | 1 |
| 27 | y_H | 1.5901355 | 0.8654588 | 2.314812 | 4.9044132 | 2.376096 | 10.12302 | 1 |
| 27 | y_I | 2.8684598 | 0.7246007 | 5.012319 | 17.609875 | 2.063907 | 150.2528 | 1 |
| 27 | y_K | 1.4136933 | 0.0365319 | 2.790855 | 4.1111111 | 1.037207 | 16.29494 | 1 |
| 27 | y_L | 1.6436293 | 0.5162063 | 2.771052 | 5.173913 | 1.675659 | 15.97544 | 1 |
| 27 | y_M | 3.0327724 | 1.596286 | 4.469259 | 20.754692 | 4.93467 | 87.292 | 1 |
| 27 | y_N | 4.9753009 | 2.243882 | 7.706719 | 144.79238 | 9.429871 | 2223.237 | 1 |
| 27 | y_O | 1.6828587 | 0.948651 | 2.417066 | 5.3809162 | 2.582224 | 11.21292 | 1 |
| 28 | y_A | 1.856298 | 0.535417 | 3.177179 | 6.4 | 1.70816 | 23.97901 | 1 |
| 28 | y_B | 3.5446607 | 1.37195 | 5.717371 | 34.627935 | 3.943032 | 304.1046 | 1 |
| 28 | y_C | 1.2356999 | 0.7957068 | 1.675693 | 3.4407858 | 2.216007 | 5.342496 | 1 |
| 28 | y_D | 2.3294614 | 0.5445188 | 4.114404 | 10.272407 | 1.723779 | 61.21572 | 1 |
| 28 | y_E | 3.8633905 | 1.524885 | 6.201896 | 47.626554 | 4.594615 | 493.6841 | 1 |
| 28 | y_F | 3.5994544 | 1.673943 | 5.524966 | 36.578273 | 5.333154 | 250.8778 | 1 |
| 28 | y_G | 1.5901355 | 0.8654588 | 2.314812 | 4.9044132 | 2.376096 | 10.12302 | 1 |
| 28 | y_H | 2.8684571 | 0.7245986 | 5.012316 | 17.609827 | 2.063903 | 150.2523 | 1 |
| 28 | y_J | 1.4136933 | 0.0365319 | 2.790855 | 4.1111111 | 1.037207 | 16.29494 | 1 |
| 28 | y_K | 1.6436293 | 0.5162063 | 2.771052 | 5.173913 | 1.675659 | 15.97544 | 1 |
| 28 | y_L | 3.0327706 | 1.596285 | 4.469257 | 20.754656 | 4.934664 | 87.29181 | 1 |
| 28 | y_M | 4.9752982 | 2.24388 | 7.706716 | 144.79199 | 9.42985 | 2223.229 | 1 |
| 28 | y_N | 1.6828582 | 0.9486506 | 2.417066 | 5.3809137 | 2.582223 | 11.21291 | 1 |
| 29 | y_A | 1.856298 | 0.535417 | 3.177179 | 6.4 | 1.70816 | 23.97901 | 1 |
| 29 | y_B | 3.5594741 | 1.385957 | 5.732991 | 35.144708 | 3.998651 | 308.8918 | 1 |
| 29 | y_C | 1.5533484 | -0.0640305 | 3.170727 | 4.7272727 | 0.9379764 | 23.82481 | 1 |
| 29 | y_D | 1.2957817 | 0.7945518 | 1.797012 | 3.6538511 | 2.213449 | 6.031596 | 1 |
| 29 | y_E | 2.3442747 | 0.558351 | 4.130198 | 10.425708 | 1.747788 | 62.19026 | 1 |
| 29 | y_F | 3.8782038 | 1.538949 | 6.217458 | 48.337313 | 4.659693 | 501.427 | 1 |
| 29 | y_G | 3.6142678 | 1.687847 | 5.540689 | 37.124152 | 5.407823 | 254.8535 | 1 |
| 29 | y_H | 1.5901355 | 0.8654588 | 2.314812 | 4.9044132 | 2.376096 | 10.12302 | 1 |
| 29 | y_I | 2.8832704 | 0.7385951 | 5.027946 | 17.872629 | 2.092993 | 152.6192 | 1 |
| 29 | y_K | 1.4136933 | 0.0365319 | 2.790855 | 4.1111111 | 1.037207 | 16.29494 | 1 |
| 29 | y_L | 1.6436293 | 0.5162063 | 2.771052 | 5.173913 | 1.675659 | 15.97544 | 1 |
| 29 | y_M | 3.047584 | 1.609879 | 4.485289 | 21.064391 | 5.002205 | 88.70258 | 1 |
| 29 | y_N | 4.9901115 | 2.258052 | 7.722171 | 146.95281 | 9.564443 | 2257.855 | 1 |
| 29 | y_O | 1.6976717 | 0.9610818 | 2.434262 | 5.461217 | 2.614523 | 11.40739 | 1 |
| 30 | y_A | 1.856298 | 0.535417 | 3.177179 | 6.4 | 1.70816 | 23.97901 | 1 |
| 30 | y_B | 3.5247978 | 1.351868 | 5.697727 | 33.94691 | 3.864639 | 298.189 | 1 |
| 30 | y_C | 1.5533484 | -0.0640305 | 3.170727 | 4.7272727 | 0.9379764 | 23.82481 | 1 |
| 30 | y_D | 1.1551374 | 0.6977281 | 1.612547 | 3.1744594 | 2.009183 | 5.015568 | 1 |
| 30 | y_E | 2.3095985 | 0.5243896 | 4.094807 | 10.07038 | 1.689427 | 60.02777 | 1 |
| 30 | y_F | 3.8435276 | 1.504819 | 6.182236 | 46.689886 | 4.503338 | 484.0732 | 1 |
| 30 | y_G | 3.5795915 | 1.653833 | 5.50535 | 35.858891 | 5.226977 | 246.0045 | 1 |
| 30 | y_H | 1.5901355 | 0.8654588 | 2.314812 | 4.9044132 | 2.376096 | 10.12302 | 1 |
| 30 | y_I | 2.8485942 | 0.7045141 | 4.992674 | 17.263496 | 2.022863 | 147.3299 | 1 |
| 30 | y_K | 1.4136933 | 0.0365319 | 2.790855 | 4.1111111 | 1.037207 | 16.29494 | 1 |
| 30 | y_L | 1.6436293 | 0.5162063 | 2.771052 | 5.173913 | 1.675659 | 15.97544 | 1 |
| 30 | y_M | 3.0129076 | 1.576091 | 4.449724 | 20.346474 | 4.836014 | 85.60335 | 1 |
| 30 | y_N | 4.9554352 | 2.223843 | 7.687027 | 141.94437 | 9.242785 | 2179.885 | 1 |
| 30 | y_O | 1.6629951 | 0.9281408 | 2.39785 | 5.2750867 | 2.529801 | 10.9995 | 1 |
| 36 | y_A | 1.856298 | 0.535417 | 3.177179 | 6.4 | 1.70816 | 23.97901 | 1 |
| 36 | y_B | 3.5417448 | 1.368676 | 5.714813 | 34.527109 | 3.930146 | 303.3275 | 1 |
| 36 | y_C | 1.5533484 | -0.0640305 | 3.170727 | 4.7272727 | 0.9379764 | 23.82481 | 1 |
| 36 | y_D | 1.223873 | 0.7557285 | 1.692018 | 3.4003318 | 2.129162 | 5.430426 | 1 |
| 36 | y_E | 2.3265454 | 0.5411677 | 4.111923 | 10.242497 | 1.718012 | 61.06404 | 1 |
| 36 | y_F | 3.8604745 | 1.521637 | 6.199312 | 47.48788 | 4.579716 | 492.4102 | 1 |
| 36 | y_G | 3.5965385 | 1.670623 | 5.522454 | 36.471769 | 5.315481 | 250.2483 | 1 |
| 36 | y_H | 1.5901355 | 0.8654588 | 2.314812 | 4.9044132 | 2.376096 | 10.12302 | 1 |
| 36 | y_I | 2.8655412 | 0.7213204 | 5.009762 | 17.558553 | 2.057148 | 149.869 | 1 |
| 36 | y_K | 1.4136933 | 0.0365319 | 2.790855 | 4.1111111 | 1.037207 | 16.29494 | 1 |
| 36 | y_L | 1.6436293 | 0.5162063 | 2.771052 | 5.173913 | 1.675659 | 15.97544 | 1 |
| 36 | y_M | 3.0298546 | 1.592828 | 4.466881 | 20.694224 | 4.917635 | 87.08472 | 1 |
| 36 | y_N | 4.9723822 | 2.24068 | 7.704084 | 144.3704 | 9.399719 | 2217.386 | 1 |
| 36 | y_O | 1.6799422 | 0.944677 | 2.415207 | 5.365246 | 2.571983 | 11.19209 | 1 |
| 37 | y_A | 1.856298 | 0.535417 | 3.177179 | 6.4 | 1.70816 | 23.97901 | 1 |
| 37 | y_B | 3.9622229 | 1.693064 | 6.231382 | 52.574063 | 5.436109 | 508.4578 | 1 |
| 37 | y_C | 1.5533484 | -0.0640305 | 3.170727 | 4.7272727 | 0.9379764 | 23.82481 | 1 |
| 37 | y_D | 1.2726734 | 0.8288797 | 1.716467 | 3.5703849 | 2.290751 | 5.564834 | 1 |
| 37 | y_E | 2.7470236 | 0.8458577 | 4.64819 | 15.596143 | 2.329975 | 104.3958 | 1 |
| 37 | y_F | 4.2809525 | 1.852575 | 6.709331 | 72.309284 | 6.376215 | 820.0214 | 1 |
| 37 | y_G | 4.0170165 | 1.983299 | 6.050734 | 55.535168 | 7.266676 | 424.4244 | 1 |
| 37 | y_H | 1.5901354 | 0.8654587 | 2.314812 | 4.9044127 | 2.376096 | 10.12302 | 1 |
| 37 | y_I | 3.2860192 | 1.044471 | 5.527568 | 26.736221 | 2.841893 | 251.5314 | 1 |
| 37 | y_K | 1.4136933 | 0.0365319 | 2.790855 | 4.1111111 | 1.037207 | 16.29494 | 1 |
| 37 | y_L | 1.6436293 | 0.5162063 | 2.771052 | 5.173913 | 1.675659 | 15.97544 | 1 |
| 37 | y_M | 3.4503343 | 1.871757 | 5.028912 | 31.510923 | 6.499705 | 152.7667 | 1 |
| 37 | y_N | 5.3928604 | 2.584114 | 8.201607 | 219.83128 | 13.25154 | 3646.805 | 1 |
| 37 | y_O | 2.100424 | 1.116819 | 3.084029 | 8.1696331 | 3.05512 | 21.84625 | 1 |
| 38 | y_A | 1.856298 | 0.535417 | 3.177179 | 6.4 | 1.70816 | 23.97901 | 1 |
| 38 | y_B | 3.5446607 | 1.37195 | 5.717371 | 34.627935 | 3.943032 | 304.1046 | 1 |
| 38 | y_C | 1.5533484 | -0.0640305 | 3.170727 | 4.7272727 | 0.9379764 | 23.82481 | 1 |
| 38 | y_D | 1.2356999 | 0.7957068 | 1.675693 | 3.4407858 | 2.216007 | 5.342496 | 1 |
| 38 | y_E | 2.3294614 | 0.5445188 | 4.114404 | 10.272407 | 1.723779 | 61.21571 | 1 |
| 38 | y_F | 3.8633905 | 1.524885 | 6.201896 | 47.626554 | 4.594615 | 493.6841 | 1 |
| 38 | y_G | 3.5994544 | 1.673943 | 5.524966 | 36.578273 | 5.333154 | 250.8778 | 1 |
| 38 | y_H | 1.5901355 | 0.8654588 | 2.314812 | 4.9044132 | 2.376096 | 10.12302 | 1 |
| 38 | y_I | 2.8684571 | 0.7245986 | 5.012316 | 17.609827 | 2.063903 | 150.2522 | 1 |
| 38 | y_K | 1.4136933 | 0.0365319 | 2.790855 | 4.1111111 | 1.037207 | 16.29494 | 1 |
| 38 | y_L | 3.0327706 | 1.596285 | 4.469257 | 20.754656 | 4.934664 | 87.29181 | 1 |
| 38 | y_M | 4.9752982 | 2.24388 | 7.706716 | 144.79199 | 9.42985 | 2223.229 | 1 |
| 38 | y_N | 1.6828582 | 0.9486506 | 2.417066 | 5.3809137 | 2.582223 | 11.21291 | 1 |
| 42 | disconnected |  |  |  |  |  |  | 0 |
| 46 | y_A | 1.856298 | 0.535417 | 3.177179 | 6.4 | 1.70816 | 23.97901 | 1 |
| 46 | y_B | 3.5446647 | 1.371953 | 5.717377 | 34.628074 | 3.943043 | 304.1061 | 1 |
| 46 | y_C | 1.5533484 | -0.0640305 | 3.170727 | 4.7272727 | 0.9379764 | 23.82481 | 1 |
| 46 | y_D | 1.2356999 | 0.7957069 | 1.675693 | 3.440786 | 2.216007 | 5.342496 | 1 |
| 46 | y_E | 2.3294654 | 0.5445215 | 4.114409 | 10.272448 | 1.723783 | 61.21605 | 1 |
| 46 | y_F | 3.8633945 | 1.524888 | 6.201901 | 47.626746 | 4.594629 | 493.6866 | 1 |
| 46 | y_G | 3.5994585 | 1.673946 | 5.524971 | 36.57842 | 5.333169 | 250.8792 | 1 |
| 46 | y_H | 1.5901355 | 0.8654588 | 2.314812 | 4.9044132 | 2.376096 | 10.12302 | 1 |
| 46 | y_J | 1.4136933 | 0.0365319 | 2.790855 | 4.1111111 | 1.037207 | 16.29494 | 1 |
| 46 | y_K | 1.6436293 | 0.5162063 | 2.771052 | 5.173913 | 1.675659 | 15.97544 | 1 |
| 46 | y_L | 3.0327732 | 1.596286 | 4.46926 | 20.75471 | 4.934673 | 87.2921 | 1 |
| 46 | y_M | 4.9753022 | 2.243883 | 7.706721 | 144.79257 | 9.429879 | 2223.24 | 1 |
| 46 | y_N | 1.6828589 | 0.9486511 | 2.417067 | 5.3809174 | 2.582224 | 11.21292 | 1 |
| 47 | y_A | 1.856298 | 0.535417 | 3.177179 | 6.4 | 1.70816 | 23.97901 | 1 |
| 47 | y_B | 3.4969473 | 1.291215 | 5.702679 | 33.014515 | 3.637204 | 299.6693 | 1 |
| 47 | y_C | 1.5533484 | -0.0640305 | 3.170727 | 4.7272727 | 0.9379764 | 23.82481 | 1 |
| 47 | y_D | 1.231475 | 0.7901956 | 1.672754 | 3.4262797 | 2.203828 | 5.32682 | 1 |
| 47 | y_E | 2.2817479 | 0.4567544 | 4.106741 | 9.7937844 | 1.578941 | 60.74844 | 1 |
| 47 | y_F | 3.8156771 | 1.44646 | 6.184894 | 45.40749 | 4.24805 | 485.3615 | 1 |
| 47 | y_G | 3.551741 | 1.589045 | 5.514437 | 34.873981 | 4.899067 | 248.2502 | 1 |
| 47 | y_H | 1.5901355 | 0.8654588 | 2.314812 | 4.9044132 | 2.376096 | 10.12302 | 1 |
| 47 | y_I | 2.8207437 | 0.6434264 | 4.998061 | 16.789332 | 1.90299 | 148.1257 | 1 |
| 47 | y_K | 1.4136933 | 0.0365319 | 2.790855 | 4.1111111 | 1.037207 | 16.29494 | 1 |
| 47 | y_L | 1.6436293 | 0.5162063 | 2.771052 | 5.173913 | 1.675659 | 15.97544 | 1 |
| 47 | y_M | 2.985057 | 1.499098 | 4.471016 | 19.78763 | 4.477647 | 87.44554 | 1 |
| 47 | y_N | 4.9275847 | 2.169827 | 7.685342 | 138.04569 | 8.756772 | 2176.214 | 1 |
| 47 | y_O | 1.6351444 | 0.8083161 | 2.461972 | 5.1301985 | 2.244126 | 11.72792 | 1 |
| 48 | y_A | 1.856298 | 0.535417 | 3.177179 | 6.4 | 1.70816 | 23.97901 | 1 |
| 48 | y_B | 3.4838406 | 1.29492 | 5.672761 | 32.584628 | 3.650705 | 290.8365 | 1 |
| 48 | y_C | 1.5533484 | -0.0640305 | 3.170727 | 4.7272727 | 0.9379764 | 23.82481 | 1 |
| 48 | y_D | 1.2303145 | 0.7896919 | 1.670937 | 3.4223056 | 2.202718 | 5.317148 | 1 |
| 48 | y_E | 2.2686413 | 0.4640027 | 4.07328 | 9.6662582 | 1.590427 | 58.74934 | 1 |
| 48 | y_F | 3.8025704 | 1.448997 | 6.156144 | 44.816232 | 4.258841 | 471.606 | 1 |
| 48 | y_G | 3.5386344 | 1.594851 | 5.482418 | 34.419882 | 4.927593 | 240.4274 | 1 |
| 48 | y_H | 1.5901355 | 0.8654588 | 2.314812 | 4.9044132 | 2.376096 | 10.12302 | 1 |
| 48 | y_I | 2.807637 | 0.6473525 | 4.967922 | 16.570716 | 1.910476 | 143.7278 | 1 |
| 48 | y_K | 1.4136933 | 0.0365319 | 2.790855 | 4.1111111 | 1.037207 | 16.29494 | 1 |
| 48 | y_L | 1.6436293 | 0.5162063 | 2.771052 | 5.173913 | 1.675659 | 15.97544 | 1 |
| 48 | y_M | 2.9719503 | 1.511063 | 4.432838 | 19.529972 | 4.531543 | 84.16996 | 1 |
| 48 | y_N | 4.9144781 | 2.170149 | 7.658808 | 136.24818 | 8.759585 | 2119.229 | 1 |
| 48 | y_O | 1.6220376 | 0.8411646 | 2.40291 | 5.0633969 | 2.319066 | 11.05531 | 1 |
| 50 | y_A | 3.5446607 | 1.37195 | 5.717371 | 34.627935 | 3.943032 | 304.1046 | 1 |
| 50 | y_B | 1.5533484 | -0.0640305 | 3.170727 | 4.7272727 | 0.9379764 | 23.82481 | 1 |
| 50 | y_C | 1.2356999 | 0.7957068 | 1.675693 | 3.4407858 | 2.216007 | 5.342496 | 1 |
| 50 | y_D | 2.3294614 | 0.5445188 | 4.114404 | 10.272407 | 1.723779 | 61.21571 | 1 |
| 50 | y_E | 3.8633905 | 1.524885 | 6.201896 | 47.626554 | 4.594615 | 493.6841 | 1 |
| 50 | y_F | 3.5994544 | 1.673943 | 5.524966 | 36.578273 | 5.333154 | 250.8778 | 1 |
| 50 | y_G | 1.5901355 | 0.8654588 | 2.314812 | 4.9044132 | 2.376096 | 10.12302 | 1 |
| 50 | y_H | 2.8684571 | 0.7245987 | 5.012316 | 17.609827 | 2.063903 | 150.2522 | 1 |
| 50 | y_J | 1.4136933 | 0.0365319 | 2.790855 | 4.1111111 | 1.037207 | 16.29494 | 1 |
| 50 | y_K | 1.6436293 | 0.5162063 | 2.771052 | 5.173913 | 1.675659 | 15.97544 | 1 |
| 50 | y_L | 3.0327706 | 1.596285 | 4.469256 | 20.754656 | 4.934664 | 87.29181 | 1 |
| 50 | y_M | 4.9752982 | 2.24388 | 7.706716 | 144.79199 | 9.429851 | 2223.229 | 1 |
| 50 | y_N | 1.6828582 | 0.9486506 | 2.417066 | 5.3809137 | 2.582223 | 11.21291 | 1 |
| 51 | y_A | 1.856298 | 0.535417 | 3.177179 | 6.4 | 1.70816 | 23.97901 | 1 |
| 51 | y_B | 3.5416577 | 1.368623 | 5.714693 | 34.524102 | 3.929935 | 303.2909 | 1 |
| 51 | y_C | 1.5533484 | -0.0640305 | 3.170727 | 4.7272727 | 0.9379764 | 23.82481 | 1 |
| 51 | y_D | 1.2235197 | 0.7579491 | 1.68909 | 3.3991307 | 2.133895 | 5.414553 | 1 |
| 51 | y_E | 2.3264583 | 0.5411214 | 4.111795 | 10.241605 | 1.717932 | 61.05623 | 1 |
| 51 | y_F | 3.8603874 | 1.521581 | 6.199194 | 47.483744 | 4.57946 | 492.352 | 1 |
| 51 | y_G | 3.5964514 | 1.670574 | 5.522328 | 36.468592 | 5.315219 | 250.217 | 1 |
| 51 | y_H | 1.5901355 | 0.8654588 | 2.314812 | 4.9044132 | 2.376096 | 10.12302 | 1 |
| 51 | y_I | 2.8654541 | 0.7212673 | 5.009641 | 17.557023 | 2.057038 | 149.8509 | 1 |
| 51 | y_K | 1.4136933 | 0.0365319 | 2.790855 | 4.1111111 | 1.037207 | 16.29494 | 1 |
| 51 | y_L | 1.6436293 | 0.5162063 | 2.771052 | 5.173913 | 1.675659 | 15.97544 | 1 |
| 51 | y_M | 3.0297675 | 1.592791 | 4.466743 | 20.692422 | 4.917457 | 87.07272 | 1 |
| 51 | y_N | 4.9722951 | 2.240619 | 7.703971 | 144.35782 | 9.399152 | 2217.134 | 1 |
| 51 | y_O | 1.6798551 | 0.9446893 | 2.415021 | 5.3647786 | 2.572014 | 11.19001 | 1 |
| 52 | y_A | 1.856298 | 0.535417 | 3.177179 | 6.4 | 1.70816 | 23.97901 | 1 |
| 52 | y_B | 3.5446659 | 1.371954 | 5.717378 | 34.628115 | 3.943047 | 304.1065 | 1 |
| 52 | y_C | 1.5533484 | -0.0640305 | 3.170727 | 4.7272727 | 0.9379764 | 23.82481 | 1 |
| 52 | y_D | 1.2357 | 0.7957069 | 1.675693 | 3.4407861 | 2.216007 | 5.342496 | 1 |
| 52 | y_E | 2.3294666 | 0.5445225 | 4.114411 | 10.27246 | 1.723785 | 61.21613 | 1 |
| 52 | y_F | 3.5994596 | 1.673946 | 5.524973 | 36.578463 | 5.333174 | 250.8795 | 1 |
| 52 | y_G | 1.5901355 | 0.8654588 | 2.314812 | 4.9044132 | 2.376096 | 10.12302 | 1 |
| 52 | y_H | 2.8684623 | 0.7246026 | 5.012322 | 17.609918 | 2.063911 | 150.2532 | 1 |
| 52 | y_J | 1.4136933 | 0.0365319 | 2.790855 | 4.1111111 | 1.037207 | 16.29494 | 1 |
| 52 | y_K | 1.6436293 | 0.5162063 | 2.771052 | 5.173913 | 1.675659 | 15.97544 | 1 |
| 52 | y_L | 3.0327739 | 1.596287 | 4.469261 | 20.754725 | 4.934676 | 87.29218 | 1 |
| 52 | y_M | 4.9753033 | 2.243884 | 7.706722 | 144.79274 | 9.429889 | 2223.243 | 1 |
| 52 | y_N | 1.6828591 | 0.9486513 | 2.417067 | 5.3809184 | 2.582225 | 11.21292 | 1 |
| 53 | y_A | 1.856298 | 0.535417 | 3.177179 | 6.4 | 1.70816 | 23.97901 | 1 |
| 53 | y_B | 3.5446607 | 1.37195 | 5.717371 | 34.627935 | 3.943032 | 304.1045 | 1 |
| 53 | y_C | 1.5533484 | -0.0640305 | 3.170727 | 4.7272727 | 0.9379764 | 23.82481 | 1 |
| 53 | y_D | 1.2356999 | 0.7957068 | 1.675693 | 3.4407858 | 2.216007 | 5.342496 | 1 |
| 53 | y_E | 2.3294614 | 0.544519 | 4.114404 | 10.272407 | 1.723779 | 61.2157 | 1 |
| 53 | y_F | 3.8633905 | 1.524885 | 6.201896 | 47.626554 | 4.594616 | 493.684 | 1 |
| 53 | y_G | 3.5994544 | 1.673943 | 5.524966 | 36.578273 | 5.333155 | 250.8778 | 1 |
| 53 | y_H | 1.5901355 | 0.8654588 | 2.314812 | 4.9044132 | 2.376096 | 10.12302 | 1 |
| 53 | y_I | 2.8684571 | 0.7245989 | 5.012315 | 17.609827 | 2.063903 | 150.2522 | 1 |
| 53 | y_K | 1.6436293 | 0.5162063 | 2.771052 | 5.173913 | 1.675659 | 15.97544 | 1 |
| 53 | y_L | 3.0327706 | 1.596285 | 4.469256 | 20.754656 | 4.934664 | 87.2918 | 1 |
| 53 | y_M | 4.9752982 | 2.24388 | 7.706716 | 144.79199 | 9.429852 | 2223.229 | 1 |
| 53 | y_N | 1.6828582 | 0.9486506 | 2.417066 | 5.3809137 | 2.582223 | 11.21291 | 1 |
| 54 | y_A | 1.856298 | 0.535417 | 3.177179 | 6.4 | 1.70816 | 23.97901 | 1 |
| 54 | y_B | 3.5446627 | 1.371951 | 5.717374 | 34.628004 | 3.943038 | 304.1053 | 1 |
| 54 | y_C | 1.5533484 | -0.0640305 | 3.170727 | 4.7272727 | 0.9379764 | 23.82481 | 1 |
| 54 | y_D | 1.2356999 | 0.7957069 | 1.675693 | 3.4407859 | 2.216007 | 5.342496 | 1 |
| 54 | y_E | 2.3294634 | 0.5445202 | 4.114407 | 10.272427 | 1.723781 | 61.21587 | 1 |
| 54 | y_F | 3.8633925 | 1.524887 | 6.201898 | 47.626649 | 4.594623 | 493.6853 | 1 |
| 54 | y_G | 3.5994564 | 1.673944 | 5.524969 | 36.578346 | 5.333162 | 250.8785 | 1 |
| 54 | y_H | 1.5901355 | 0.8654588 | 2.314812 | 4.9044132 | 2.376096 | 10.12302 | 1 |
| 54 | y_I | 2.8684591 | 0.7246001 | 5.012318 | 17.609862 | 2.063906 | 150.2526 | 1 |
| 54 | y_K | 1.4136933 | 0.0365319 | 2.790855 | 4.1111111 | 1.037207 | 16.29494 | 1 |
| 54 | y_L | 1.6436293 | 0.5162063 | 2.771052 | 5.173913 | 1.675659 | 15.97544 | 1 |
| 54 | y_M | 3.0327719 | 1.596285 | 4.469258 | 20.754682 | 4.934669 | 87.29195 | 1 |
| 54 | y_N | 1.6828585 | 0.9486509 | 2.417066 | 5.3809155 | 2.582224 | 11.21291 | 1 |
| 56 | y_A | 1.856298 | 0.535417 | 3.177179 | 6.4 | 1.70816 | 23.97901 | 1 |
| 56 | y_B | 3.5494077 | 1.376003 | 5.722812 | 34.792703 | 3.959047 | 305.7635 | 1 |
| 56 | y_C | 1.5533484 | -0.0640305 | 3.170727 | 4.7272727 | 0.9379764 | 23.82481 | 1 |
| 56 | y_D | 1.2549533 | 0.7618322 | 1.748074 | 3.5076744 | 2.142198 | 5.743532 | 1 |
| 56 | y_E | 2.3342083 | 0.5484217 | 4.119995 | 10.321286 | 1.73052 | 61.55893 | 1 |
| 56 | y_F | 3.8681374 | 1.528988 | 6.207287 | 47.853173 | 4.613504 | 496.3529 | 1 |
| 56 | y_G | 3.6042014 | 1.677907 | 5.530496 | 36.752321 | 5.354339 | 252.2689 | 1 |
| 56 | y_H | 1.5901354 | 0.8654588 | 2.314812 | 4.9044132 | 2.376096 | 10.12302 | 1 |
| 56 | y_I | 2.8732041 | 0.7286429 | 5.017765 | 17.693619 | 2.072266 | 151.0733 | 1 |
| 56 | y_K | 1.4136933 | 0.0365319 | 2.790855 | 4.1111111 | 1.037207 | 16.29494 | 1 |
| 56 | y_L | 1.6436293 | 0.5162063 | 2.771052 | 5.173913 | 1.675659 | 15.97544 | 1 |
| 56 | y_M | 3.0375176 | 1.599983 | 4.475052 | 20.853411 | 4.952948 | 87.79919 | 1 |
| 56 | y_N | 4.9800451 | 2.248075 | 7.712015 | 145.48094 | 9.469495 | 2235.041 | 1 |
| 56 | y_O | 1.6876052 | 0.9513481 | 2.423862 | 5.4065176 | 2.589198 | 11.28938 | 1 |

Table S17. Meta-Regression Analysis of MoCA by Region

| **Intervention** | **Covariate (Region)** | **Coefficient** | **Standard Error** | **Z-statistic** | **P>z** | **lower confidence interval** | **upper confidence interval** |
| --- | --- | --- | --- | --- | --- | --- | --- |
| _y_B | cov1 | 0.0943378 | 129.3435 | 0 | 0.999 | -253.4143 | 253.6029 |
| _y_B | _cons | 0.3665912 | 0.4425313 | 0.83 | 0.407 | -0.5007542 | 1.233937 |
| _y_C | _cons | 1.303821 | 0.5480123 | 2.38 | 0.017 | 0.2297363 | 2.377905 |
| _y_D | cov1 | -0.0324432 | 0.3316258 | -0.1 | 0.922 | -0.6824179 | 0.6175314 |
| _y_D | _cons | 0.4957414 | 0.5073815 | 0.98 | 0.329 | -0.4987081 | 1.490191 |
| _y_E | _cons | -0.0100547 | 0.442575 | -0.02 | 0.982 | -0.8774859 | 0.8573765 |
| _y_F | cov1 | 0.0918019 | 129.3438 | 0 | 0.999 | -253.4173 | 253.6009 |
| _y_F | _cons | -0.5851387 | 0.6114276 | -0.96 | 0.339 | -1.783515 | 0.6132374 |
| _y_G | _cons | -0.0921644 | 0.6223411 | -0.15 | 0.882 | -1.311931 | 1.127602 |
| _y_H | _cons | 1.68553 | 0.6274615 | 2.69 | 0.007 | 0.4557285 | 2.915332 |
| _y_I | cov1 | 0.1832195 | 0.3318489 | 0.55 | 0.581 | -0.4671924 | 0.8336315 |
| _y_I | _cons | 0.3490716 | 0.4768461 | 0.73 | 0.464 | -0.5855297 | 1.283673 |
| _y_J | _cons | 0.944222 | 0.5201521 | 1.82 | 0.069 | -0.0752574 | 1.963701 |
| _y_K | cov1 | 0.2645408 | 0.3974814 | 0.67 | 0.506 | -0.5145084 | 1.04359 |
| _y_K | _cons | 0.5225638 | 0.5471812 | 0.96 | 0.34 | -0.5498916 | 1.595019 |
| _y_L | cov1 | -0.0894102 | 0.305597 | -0.29 | 0.77 | -0.6883692 | 0.5095489 |

| _y_L | _cons | -0.2527931 | 0.4436526 | -0.57 | 0.569 | -1.122336 | 0.6167501 |
| --- | --- | --- | --- | --- | --- | --- | --- |
| _y_M | cov1 | -1.520986 | 129.3452 | -0.01 | 0.991 | -255.0329 | 251.9909 |
| _y_M | _cons | 1.738775 | 0.6188169 | 2.81 | 0.005 | 0.5259157 | 2.951633 |
| _y_N | cov1 | 0.0837869 | 0.4395979 | 0.19 | 0.849 | -0.7778092 | 0.9453831 |
| _y_N | _cons | 1.741927 | 0.5731279 | 3.04 | 0.002 | 0.6186166 | 2.865237 |
| _y_O | cov1 | -0.0261433 | 0.3897919 | -0.07 | 0.947 | -0.7901213 | 0.7378348 |
| _y_O | _cons | 1.291587 | 0.5384243 | 2.4 | 0.016 | 0.2362946 | 2.346879 |

Table S18.Meta-Regression Analysis of MOCA by Follow-Up Duration

| **Intervention** | **Covariate (Follow-up Duration)** | **Coefficient** | **Standard Error** | **Z-statistic** | **P>z** | **lower confidence interval** | **upper confidence interval** |
| --- | --- | --- | --- | --- | --- | --- | --- |
| _y_B | cov2 | -1.593929 | 1.126532 | -1.41 | 0.157 | -3.801891 | 0.6140321 |
| _y_B | _cons | 1.960497 | 1.20166 | 1.63 | 0.103 | -0.3947139 | 4.315708 |
| _y_C | _cons | 1.380836 | 0.9869423 | 1.4 | 0.162 | -0.5535353 | 3.315208 |
| _y_D | cov2 | -0.1951911 | 0.5760322 | -0.34 | 0.735 | -1.324193 | 0.9338112 |
| _y_D | _cons | 0.6993275 | 0.9579035 | 0.73 | 0.465 | -1.178129 | 2.576784 |
| _y_E | _cons | -0.0100779 | 0.4202834 | -0.02 | 0.981 | -0.8338183 | 0.8136625 |
| _y_F | cov2 | -1.107525 | 1.17541 | -0.94 | 0.346 | -3.411286 | 1.196237 |
| _y_F | _cons | 0.8536049 | 1.162247 | 0.73 | 0.463 | -1.424357 | 3.131567 |
| _y_G | _cons | -0.092211 | 0.5906152 | -0.16 | 0.876 | -1.249796 | 1.065374 |
| _y_H | _cons | 1.4647 | 1.030763 | 1.42 | 0.155 | -0.5555583 | 3.484958 |
| _y_I | cov2 | -0.2654564 | 0.6447864 | -0.41 | 0.681 | -1.529215 | 0.9983018 |
| _y_I | _cons | 0.6789295 | 0.9531134 | 0.71 | 0.476 | -1.189139 | 2.546997 |
| _y_J | _cons | 0.9488491 | 1.023552 | 0.93 | 0.354 | -1.057275 | 2.954973 |
| _y_K | _cons | 0.6515197 | 0.9672699 | 0.67 | 0.501 | -1.244294 | 2.547334 |
| _y_L | cov2 | -0.0560531 | 0.5923891 | -0.09 | 0.925 | -1.217114 | 1.105008 |
| _y_L | _cons | -0.1967864 | 0.9400087 | -0.21 | 0.834 | -2.03917 | 1.645597 |
| _y_M | cov2 | 0.6500077 | 0.9270389 | 0.7 | 0.483 | -1.166955 | 2.466971 |
| _y_M | _cons | 1.564737 | 1.076876 | 1.45 | 0.146 | -0.5459012 | 3.675375 |
| _y_N | _cons | 1.663952 | 0.9858526 | 1.69 | 0.091 | -0.268284 | 3.596187 |
| _y_O | cov2 | 0.9219754 | 0.7219976 | 1.28 | 0.202 | -0.4931139 | 2.337065 |
| _y_O | _cons | 0.9663143 | 0.9846263 | 0.98 | 0.326 | -0.9635178 | 2.896146 |

Table S19.Meta-Regression Analysis of MMSE by Region

| **Intervention** | **Covariate (Region)** | **Coefficient** | **Standard Error** | **Z-statistic** | **P>z** | **lower confidence interval** | **upper confidence interval** |
| --- | --- | --- | --- | --- | --- | --- | --- |
| _y_B | _cons | 1.825602 | 1.677222 | 1.09 | 0.276 | -1.461694 | 5.112897 |
| _y_C | cov1 | -0.4244942 | 31.48814 | -0.01 | 0.989 | -62.14012 | 61.29113 |
| _y_C | _cons | 1.344108 | 62.96479 | 0.02 | 0.983 | -122.0646 | 124.7528 |
| _y_D | cov1 | -0.0187087 | 31.48223 | 0 | 1 | -61.72274 | 61.68532 |
| _y_D | _cons | 0.2326157 | 62.94958 | 0 | 0.997 | -123.1463 | 123.6115 |
| _y_E | _cons | -0.050718 | 1.168962 | -0.04 | 0.965 | -2.341841 | 2.240405 |
| _y_F | cov1 | -0.2595779 | 31.48567 | -0.01 | 0.993 | -61.97035 | 61.45119 |
| _y_F | _cons | 0.3583794 | 62.95425 | 0.01 | 0.995 | -123.0297 | 123.7464 |
| _y_G | _cons | 2.020489 | 1.659518 | 1.22 | 0.223 | -1.232107 | 5.273086 |
| _y_H | cov1 | 0.6734009 | 31.48178 | 0.02 | 0.983 | -61.02975 | 62.37656 |
| _y_H | _cons | 0.0028279 | 62.94987 | 0 | 1 | -123.3767 | 123.3823 |
| _y_I | _cons | 0.2951337 | 62.95692 | 0 | 0.996 | -123.0982 | 123.6884 |
| _y_J | cov1 | 0.507151 | 31.48526 | 0.02 | 0.987 | -61.20282 | 62.21712 |
| _y_J | _cons | -0.0620656 | 62.95175 | 0 | 0.999 | -123.4452 | 123.3211 |
| _y_K | cov1 | 0.2637862 | 31.48019 | 0.01 | 0.993 | -61.43625 | 61.96382 |
| _y_K | _cons | -0.8662058 | 62.94855 | -0.01 | 0.989 | -124.2431 | 122.5107 |
| _y_L | _cons | -0.2781843 | 62.95908 | 0 | 0.996 | -123.6757 | 123.1193 |
| _y_M | cov1 | -0.2167788 | 31.48961 | -0.01 | 0.995 | -61.93529 | 61.50173 |
| _y_M | _cons | 0.785532 | 62.95248 | 0.01 | 0.99 | -122.5991 | 124.1701 |
| _y_N | cov1 | 0.0854434 | 31.48782 | 0 | 0.998 | -61.62955 | 61.80044 |
| _y_N | _cons | 1.349756 | 62.95206 | 0.02 | 0.983 | -122.034 | 124.7335 |
| _y_O | cov1 | -0.188709 | 31.48242 | -0.01 | 0.995 | -61.89313 | 61.51571 |
| _y_O | _cons | 0.5559568 | 62.9496 | 0.01 | 0.993 | -122.823 | 123.9349 |
| _y_P | _cons | 0.5680326 | 62.95752 | 0.01 | 0.993 | -122.8264 | 123.9625 |

Table S20.Meta-Regression Analysis of MMSE by Follow-Up Duration

| **Intervention** | **Covariate (Follow-up Duration)** | **Coefficient** | **Standard Error** | **Z-statistic** | **P>z** | **lower confidence interval** | **upper confidence interval** |
| --- | --- | --- | --- | --- | --- | --- | --- |
| _y_B | _cons | 0.9381197 | 315.0608 | 0 | 0.998 | -616.5697 | 618.4459 |
| _y_C | cov2 | -0.3838286 | 27.43093 | -0.01 | 0.989 | -54.14747 | 53.37981 |
|  | _cons | 1.262835 | 54.84905 | 0.02 | 0.982 | -106.2393 | 108.765 |
| _y_D | cov2 | -0.1102963 | 27.43323 | 0 | 0.997 | -53.87844 | 53.65784 |
|  | _cons | 0.0842079 | 54.83813 | 0 | 0.999 | -107.3965 | 107.565 |
| _y_E | cov2 | 0.8875489 | 315.0627 | 0 | 0.998 | -616.6241 | 618.3992 |
|  | _cons | -1.825755 | 630.1225 | 0 | 0.998 | -1236.843 | 1233.192 |
| _y_F | cov2 | -1.110026 | 27.4978 | -0.04 | 0.968 | -55.00472 | 52.78467 |
|  | _cons | 0.4972575 | 54.84556 | 0.01 | 0.993 | -106.9981 | 107.9926 |
| _y_G | _cons | 1.178458 | 54.84544 | 0.02 | 0.983 | -106.3166 | 108.6736 |
| _y_H | cov2 | -0.8622539 | 27.43334 | -0.03 | 0.975 | -54.63061 | 52.9061 |
|  | _cons | 0.6152563 | 54.83743 | 0.01 | 0.991 | -106.8641 | 108.0946 |
| _y_I | _cons | 0.2574032 | 54.84518 | 0 | 0.996 | -107.2372 | 107.752 |
| _y_J | _cons | 0.0029627 | 54.83869 | 0 | 1 | -107.4789 | 107.4848 |
| _y_K | cov2 | 0.3342921 | 27.42556 | 0.01 | 0.99 | -53.41881 | 54.08739 |
|  | _cons | -1.007119 | 54.83683 | -0.02 | 0.985 | -108.4853 | 106.4711 |
| _y_L | _cons | -0.0848068 | 27.44547 | 0 | 0.998 | -53.87693 | 53.70732 |
| _y_M | cov2 | -1.461869 | 27.47496 | -0.05 | 0.958 | -55.3118 | 52.38806 |
|  | _cons | 1.192858 | 54.84592 | 0.02 | 0.983 | -106.3032 | 108.6889 |
| _y_N | cov2 | -0.1801611 | 27.46468 | -0.01 | 0.995 | -54.00995 | 53.64963 |
|  | _cons | 1.226142 | 54.84026 | 0.02 | 0.982 | -106.2588 | 108.7111 |
| _y_O | cov2 | 0.5273759 | 27.43921 | 0.02 | 0.985 | -53.25248 | 54.30723 |
|  | _cons | -0.0557205 | 54.83908 | 0 | 0.999 | -107.5383 | 107.4269 |
| _y_P | _cons | 0.1347952 | 27.44689 | 0 | 0.996 | -53.66013 | 53.92972 |

Table S21.Meta-Regression Analysis of BI by Region

| **Intervention** | **Covariate (Region)** | **Coefficient** | **Standard Error** | **Z-statistic** | **P>z** | **lower confidence interval** | **upper confidence interval** |
| --- | --- | --- | --- | --- | --- | --- | --- |
| _y_B | _cons | 0.4891132 | 0.4316586 | 1.13 | 0.257 | -0.3569221 | 1.335148 |
| _y_C | _cons | 0.8740462 | 65.54921 | 0.01 | 0.989 | -127.6 | 129.3481 |
| _y_D | cov1 | 0.1106962 | 65.54879 | 0 | 0.999 | -128.3626 | 128.584 |
| _y_D | _cons | 0.0396745 | 0.4400985 | 0.09 | 0.928 | -0.8229027 | 0.9022516 |
| _y_E | cov1 | 0.4927849 | 65.54866 | 0.01 | 0.994 | -127.9802 | 128.9658 |
| _y_E | _cons | -0.1421121 | 0.3355856 | -0.42 | 0.672 | -0.7998479 | 0.5156237 |
| _y_F | cov1 | 0.5992652 | 328.5882 | 0 | 0.999 | -643.4219 | 644.6204 |
| _y_F | _cons | -0.4753286 | 322.0754 | 0 | 0.999 | -631.7316 | 630.7809 |
| _y_G | cov1 | 0.0683828 | 65.54845 | 0 | 0.999 | -128.4042 | 128.541 |
| _y_G | _cons | -0.4916039 | 0.2937639 | -1.67 | 0.094 | -1.067371 | 0.0841628 |
| _y_H | _cons | 0.4216401 | 0.4322788 | 0.98 | 0.329 | -0.4256109 | 1.268891 |
| _y_I | _cons | -0.3438618 | 131.0968 | 0 | 0.998 | -257.2889 | 256.6011 |
| _y_J | _cons | 0.4756677 | 322.0754 | 0 | 0.999 | -630.7806 | 631.7319 |
| _y_K | cov1 | 0.1011873 | 65.54871 | 0 | 0.999 | -128.3719 | 128.5743 |
| _y_K | _cons | 0.4467776 | 0.3592984 | 1.24 | 0.214 | -0.2574344 | 1.15099 |

Table S22.Meta-Regression Analysis of BI by Follow-Up Duration

| **Intervention** | **Covariate (Follow-up Duration)** | **Coefficient** | **Standard Error** | **Z-statistic** | **P>z** | **lower confidence interval** | **upper confidence interval** |
| --- | --- | --- | --- | --- | --- | --- | --- |
| _y_B | _cons | 0.4891126 | 0.4091012 | 1.2 | 0.232 | -0.3127111 | 1.290936 |
| _y_C | _cons | 0.8056626 | 0.4266653 | 1.89 | 0.059 | -0.030586 | 1.641911 |
| _y_D | cov2 | 0.3848578 | 104.8125 | 0 | 0.997 | -205.044 | 205.8137 |
| _y_D | _cons | -0.0078793 | 0.3411243 | -0.02 | 0.982 | -0.6764706 | 0.660712 |
| _y_E | cov2 | -0.725627 | 104.8124 | -0.01 | 0.994 | -206.1541 | 204.7028 |
| _y_E | _cons | 0.7066916 | 0.4064778 | 1.74 | 0.082 | -0.0899903 | 1.503374 |
| _y_F | _cons | 0.0555528 | 0.416747 | 0.13 | 0.894 | -0.7612562 | 0.8723619 |
| _y_G | cov2 | 0.1235759 | 104.8119 | 0 | 0.999 | -205.3039 | 205.5511 |
| _y_G | _cons | -0.4916045 | 0.2771546 | -1.77 | 0.076 | -1.034817 | 0.0516085 |
| _y_H | _cons | 0.5452154 | 104.8119 | 0.01 | 0.996 | -204.8824 | 205.9728 |
| _y_I | _cons | -0.480628 | 0.4029381 | -1.19 | 0.233 | -1.270372 | 0.3091161 |
| _y_J | _cons | 1.006549 | 0.5329842 | 1.89 | 0.059 | -0.0380811 | 2.051179 |
| _y_K | cov2 | 0.3574379 | 104.8126 | 0 | 0.997 | -205.0714 | 205.7863 |
| _y_K | _cons | 0.266957 | 0.4597064 | 0.58 | 0.561 | -0.634051 | 1.167965 |
| _y_B | _cons | 0.4891126 | 0.4091012 | 1.2 | 0.232 | -0.3127111 | 1.290936 |
| _y_C | _cons | 0.8056626 | 0.4266653 | 1.89 | 0.059 | -0.030586 | 1.641911 |

Table S23.Meta-Regression Analysis of TER by Region

| **Intervention** | **Covariate (Region)** | **Coefficient** | **Standard Error** | **Z-statistic** | **P>z** | **lower confidence interval** | **upper confidence interval** |
| --- | --- | --- | --- | --- | --- | --- | --- |
| _y_B | _cons | 0.0804445 | 93.68325 | 0 | 0.999 | -183.5354 | 183.6962 |
| _y_C | _cons | -0.1087927 | 0.1695559 | -0.64 | 0.521 | -0.4411162 | 0.2235309 |
| _y_D | cov1 | 0.1002307 | 27.52274 | 0 | 0.997 | -53.84335 | 54.04381 |
| _y_D | _cons | -0.0837521 | 0.1473172 | -0.57 | 0.57 | -0.3724886 | 0.2049844 |
| _y_E | cov1 | 0.0136262 | 57.30678 | 0 | 1 | -112.3056 | 112.3328 |
| _y_E | _cons | -0.1231544 | 93.68321 | 0 | 0.999 | -183.7389 | 183.4926 |
| _y_F | _cons | 0.3121635 | 93.68342 | 0 | 0.997 | -183.304 | 183.9283 |
| _y_G | _cons | 0.1199807 | 62.38637 | 0 | 0.998 | -122.1551 | 122.395 |
| _y_H | cov1 | -0.0821149 | 27.52305 | 0 | 0.998 | -54.0263 | 53.86207 |
| _y_H | _cons | 0.1987948 | 0.237809 | 0.84 | 0.403 | -0.2673023 | 0.6648919 |
| _y_I | _cons | 0.010377 | 93.68332 | 0 | 1 | -183.6056 | 183.6263 |
| _y_J | cov1 | 0.0827744 | 27.52268 | 0 | 0.998 | -53.8607 | 54.02624 |
| _y_J | _cons | -0.2758469 | 0.1244475 | -2.22 | 0.027 | -0.5197594 | -0.0319343 |
| _y_K | _cons | 0.0166481 | 27.52291 | 0 | 1 | -53.92727 | 53.96057 |
| _y_L | _cons | 0.144007 | 0.2088907 | 0.69 | 0.491 | -0.2654113 | 0.5534253 |
| _y_M | cov1 | -0.1368891 | 55.23009 | 0 | 0.998 | -108.3859 | 108.1121 |
| _y_M | _cons | 0.1916639 | 0.3322879 | 0.58 | 0.564 | -0.4596084 | 0.8429362 |
| _y_N | _cons | 0.0650283 | 62.38642 | 0 | 0.999 | -122.2101 | 122.3402 |
| _y_O | cov1 | 0.1178924 | 27.52308 | 0 | 0.997 | -53.82636 | 54.06214 |
| _y_O | _cons | -0.0596505 | 0.3013715 | -0.2 | 0.843 | -0.6503278 | 0.5310268 |

Table S24.Meta-Regression Analysis of TER by Follow-Up Duration

| **Intervention** | **Covariate (Follow-up Duration)** | **Coefficient** | **Standard Error** | **Z-statistic** | **P>z** | **lower confidence interval** | **upper confidence interval** |
| --- | --- | --- | --- | --- | --- | --- | --- |
| _y_B | _cons | 0.3249138 | 0.2703403 | 1.2 | 0.229 | -0.2049434 | 0.8547709 |
| _y_C | _cons | -0.1087934 | 0.1286555 | -0.85 | 0.398 | -0.3609536 | 0.1433667 |
| _y_D | cov2 | 0.1928584 | 52.33063 | 0 | 0.997 | -102.3733 | 102.759 |
| _y_D | _cons | -0.1213939 | 0.1038306 | -1.17 | 0.242 | -0.3248981 | 0.0821104 |
| _y_E | cov2 | -0.2671691 | 136.9886 | 0 | 0.998 | -268.7598 | 268.2255 |
| _y_E | _cons | 0.1213149 | 0.2492571 | 0.49 | 0.626 | -0.36722 | 0.6098498 |
| _y_F | _cons | 0.2894637 | 136.9884 | 0 | 0.998 | -268.2029 | 268.7818 |
| _y_G | _cons | 0.3212814 | 0.2559034 | 1.26 | 0.209 | -0.18028 | 0.8228428 |
| _y_H | cov2 | 0.6996738 | 52.33121 | 0.01 | 0.989 | -101.8676 | 103.267 |
| _y_H | _cons | -0.0763449 | 0.1209745 | -0.63 | 0.528 | -0.3134506 | 0.1607607 |
| _y_I | _cons | -0.0123228 | 136.9884 | 0 | 1 | -268.5046 | 268.48 |
| _y_J | cov2 | 0.1150577 | 52.33061 | 0 | 0.998 | -102.451 | 102.6812 |
| _y_J | _cons | -0.2758476 | 0.0968952 | -2.85 | 0.004 | -0.4657587 | -0.0859365 |
| _y_K | _cons | 0.0489307 | 52.33062 | 0 | 0.999 | -102.5172 | 102.6151 |
| _y_L | _cons | 0.2590639 | 52.33073 | 0 | 0.996 | -102.3073 | 102.8254 |
| _y_M | _cons | 0.285619 | 0.2147378 | 1.33 | 0.183 | -0.1352593 | 0.7064973 |
| _y_N | _cons | 0.2822452 | 0.2546545 | 1.11 | 0.268 | -0.2168685 | 0.7813589 |
| _y_O | cov2 | 0.0713247 | 52.3308 | 0 | 0.999 | -102.4952 | 102.6378 |
| _y_O | _cons | 0.0343056 | 0.1806118 | 0.19 | 0.849 | -0.319687 | 0.3882982 |

Table S25. GRADE Assessment for MoCA

| **Comparison** | **Number of studies** | **Within-study bias** | **Reporting bias** | **Indirectness** | **Imprecision** | **Heterogeneity** | **Incoherence** |
| --- | --- | --- | --- | --- | --- | --- | --- |
| BA:BA-C-SOC | 1 | No concerns | Low risk | No concerns | Some concerns | No concerns | No concerns |
| BA:BA-SOC | 1 | No concerns | Low risk | No concerns | Some concerns | Some concerns | No concerns |
| BA:C | 1 | No concerns | Low risk | No concerns | Some concerns | No concerns | No concerns |
| BA:SOC | 2 | No concerns | Low risk | No concerns | Some concerns | Some concerns | No concerns |
| BA-C-SOC:C | 1 | No concerns | Low risk | No concerns | Some concerns | Some concerns | Some concerns |
| BA-C-SOC:C-SOC | 3 | No concerns | Low risk | No concerns | No concerns | Some concerns | Some concerns |
| BA-C-SOC:SOC | 1 | No concerns | Low risk | No concerns | No concerns | No concerns | Some concerns |
| BA-N-SOC:N-SOC | 1 | No concerns | Low risk | No concerns | Some concerns | No concerns | No concerns |
| BA-N-SOC:SOC | 2 | No concerns | Low risk | No concerns | No concerns | No concerns | No concerns |
| BA-SOC:SOC | 6 | No concerns | Low risk | No concerns | No concerns | Some concerns | No concerns |
| BA-SOC:ScA-SOC | 2 | No concerns | Low risk | No concerns | No concerns | Some concerns | No concerns |
| C:SOC | 1 | No concerns | Low risk | No concerns | Some concerns | Some concerns | No concerns |
| C-SOC:ScA-C-SOC | 1 | No concerns | Low risk | No concerns | No concerns | No concerns | Some concerns |
| EA:SOC | 1 | No concerns | Low risk | No concerns | Some concerns | No concerns | Some concerns |
| EA-N-SOC:EA-SOC | 1 | No concerns | Low risk | No concerns | No concerns | Some concerns | No concerns |
| EA-N-SOC:N-SOC | 1 | No concerns | Low risk | No concerns | Some concerns | No concerns | No concerns |
| EA-SOC:N-SOC | 1 | No concerns | Low risk | No concerns | Some concerns | Some concerns | No concerns |
| EA-SOC:SOC | 12 | No concerns | Low risk | No concerns | No concerns | Some concerns | No concerns |
| EyeAcu-N-SOC:N-SOC | 1 | No concerns | Low risk | No concerns | Some concerns | No concerns | No concerns |
| EyeAcu-N-SOC:SOC | 1 | No concerns | Low risk | No concerns | No concerns | No concerns | No concerns |
| N-SOC:SOC | 2 | No concerns | Low risk | No concerns | No concerns | No concerns | Some concerns |
| N-SOC:ScA-N-SOC | 4 | No concerns | Low risk | No concerns | No concerns | No concerns | No concerns |
| N-SOC:ScA-SOC | 2 | No concerns | Low risk | No concerns | No concerns | Some concerns | Some concerns |
| ScA-SOC:SOC | 1 | No concerns | Low risk | No concerns | No concerns | No concerns | No concerns |
| ScA-C-SOC:ScA-SOC | 2 | No concerns | Low risk | No concerns | Some concerns | Some concerns | Some concerns |
| ScA-N-SOC:ScA-SOC | 3 | No concerns | Low risk | No concerns | No concerns | No concerns | No concerns |
| BA:BA-N-SOC | 0 | No concerns | Low risk | No concerns | No concerns | Some concerns | Some concerns |
| BA:C-SOC | 0 | No concerns | Low risk | No concerns | Some concerns | No concerns | Some concerns |
| BA:EA | 0 | No concerns | Low risk | No concerns | Some concerns | No concerns | Some concerns |
| BA:EA-N-SOC | 0 | No concerns | Low risk | No concerns | No concerns | Some concerns | Some concerns |
| BA:EA-SOC | 0 | No concerns | Low risk | No concerns | Some concerns | Some concerns | Some concerns |
| BA:EyeAcu-N-SOC | 0 | No concerns | Low risk | No concerns | Some concerns | Some concerns | Some concerns |
| BA:N-SOC | 0 | No concerns | Low risk | No concerns | Some concerns | Some concerns | Some concerns |
| BA:ScA-C-SOC | 0 | No concerns | Low risk | No concerns | No concerns | No concerns | Some concerns |
| BA:ScA-N-SOC | 0 | No concerns | Low risk | No concerns | No concerns | No concerns | Some concerns |
| BA:ScA-SOC | 0 | No concerns | Low risk | No concerns | No concerns | Some concerns | Some concerns |
| BA-C-SOC:BA-N-SOC | 0 | No concerns | Low risk | No concerns | Some concerns | Some concerns | Some concerns |
| BA-C-SOC:BA-SOC | 0 | No concerns | Low risk | No concerns | Some concerns | Some concerns | Some concerns |
| BA-C-SOC:EA | 0 | No concerns | Low risk | No concerns | Some concerns | No concerns | Some concerns |
| BA-C-SOC:EA-N-SOC | 0 | No concerns | Low risk | No concerns | Some concerns | No concerns | Some concerns |
| BA-C-SOC:EA-SOC | 0 | No concerns | Low risk | No concerns | Some concerns | Some concerns | Some concerns |
| BA-C-SOC:EyeAcu-N-SOC | 0 | No concerns | Low risk | No concerns | Major concerns | No concerns | Some concerns |
| BA-C-SOC:N-SOC | 0 | No concerns | Low risk | No concerns | Some concerns | No concerns | Some concerns |
| BA-C-SOC:ScA-C-SOC | 0 | No concerns | Low risk | No concerns | Some concerns | Some concerns | Some concerns |
| BA-C-SOC:ScA-N-SOC | 0 | No concerns | Low risk | No concerns | No concerns | Some concerns | Some concerns |
| BA-C-SOC:ScA-SOC | 0 | No concerns | Low risk | No concerns | Some concerns | Some concerns | Some concerns |
| BA-N-SOC:BA-SOC | 0 | No concerns | Low risk | No concerns | No concerns | Some concerns | Some concerns |
| BA-N-SOC:C | 0 | No concerns | Low risk | No concerns | No concerns | Some concerns | Some concerns |
| BA-N-SOC:C-SOC | 0 | No concerns | Low risk | No concerns | No concerns | Some concerns | Some concerns |
| BA-N-SOC:EA | 0 | No concerns | Low risk | No concerns | No concerns | No concerns | Some concerns |
| BA-N-SOC:EA-N-SOC | 0 | No concerns | Low risk | No concerns | Major concerns | No concerns | Some concerns |
| BA-N-SOC:EA-SOC | 0 | No concerns | Low risk | No concerns | No concerns | Some concerns | Some concerns |
| BA-N-SOC:EyeAcu-N-SOC | 0 | No concerns | Low risk | No concerns | Some concerns | No concerns | Some concerns |
| BA-N-SOC:ScA-C-SOC | 0 | No concerns | Low risk | No concerns | Some concerns | No concerns | Some concerns |
| BA-N-SOC:ScA-N-SOC | 0 | No concerns | Low risk | No concerns | Some concerns | Some concerns | Some concerns |
| BA-N-SOC:ScA-SOC | 0 | No concerns | Low risk | No concerns | Some concerns | No concerns | Some concerns |
| BA-SOC:C | 0 | No concerns | Low risk | No concerns | Some concerns | No concerns | Some concerns |
| BA-SOC:C-SOC | 0 | No concerns | Low risk | No concerns | Some concerns | Some concerns | Some concerns |
| BA-SOC:EA | 0 | No concerns | Low risk | No concerns | Some concerns | Some concerns | Some concerns |
| BA-SOC:EA-N-SOC | 0 | No concerns | Low risk | No concerns | No concerns | Some concerns | Some concerns |
| BA-SOC:EA-SOC | 0 | No concerns | Low risk | No concerns | No concerns | Some concerns | Some concerns |
| BA-SOC:EyeAcu-N-SOC | 0 | No concerns | Low risk | No concerns | Some concerns | Some concerns | Some concerns |
| BA-SOC:N-SOC | 0 | No concerns | Low risk | No concerns | Some concerns | Some concerns | Some concerns |
| BA-SOC:ScA-C-SOC | 0 | No concerns | Low risk | No concerns | No concerns | Some concerns | Some concerns |
| BA-SOC:ScA-N-SOC | 0 | No concerns | Low risk | No concerns | No concerns | No concerns | Some concerns |
| C:C-SOC | 0 | No concerns | Low risk | No concerns | Some concerns | No concerns | Some concerns |
| C:EA | 0 | No concerns | Low risk | No concerns | Major concerns | No concerns | Some concerns |
| C:EA-N-SOC | 0 | No concerns | Low risk | No concerns | No concerns | Some concerns | Some concerns |
| C:EA-SOC | 0 | No concerns | Low risk | No concerns | Some concerns | Some concerns | Some concerns |
| C:EyeAcu-N-SOC | 0 | No concerns | Low risk | No concerns | Some concerns | Some concerns | Some concerns |
| C:N-SOC | 0 | No concerns | Low risk | No concerns | Some concerns | Some concerns | Some concerns |
| C:ScA-C-SOC | 0 | No concerns | Low risk | No concerns | No concerns | Some concerns | Some concerns |
| C:ScA-N-SOC | 0 | No concerns | Low risk | No concerns | No concerns | No concerns | Some concerns |
| C:ScA-SOC | 0 | No concerns | Low risk | No concerns | No concerns | Some concerns | Some concerns |
| C-SOC:EA | 0 | No concerns | Low risk | No concerns | Some concerns | No concerns | Some concerns |
| C-SOC:EA-N-SOC | 0 | No concerns | Low risk | No concerns | No concerns | Some concerns | Some concerns |
| C-SOC:EA-SOC | 0 | No concerns | Low risk | No concerns | Some concerns | Some concerns | Some concerns |
| C-SOC:EyeAcu-N-SOC | 0 | No concerns | Low risk | No concerns | Some concerns | Some concerns | Some concerns |
| C-SOC:N-SOC | 0 | No concerns | Low risk | No concerns | Some concerns | Some concerns | Some concerns |
| C-SOC:SOC | 0 | No concerns | Low risk | No concerns | Some concerns | Some concerns | Some concerns |
| C-SOC:ScA-N-SOC | 0 | No concerns | Low risk | No concerns | No concerns | No concerns | Some concerns |
| C-SOC:ScA-SOC | 0 | No concerns | Low risk | No concerns | No concerns | Some concerns | Some concerns |
| EA:EA-N-SOC | 0 | No concerns | Low risk | No concerns | No concerns | No concerns | Some concerns |
| EA:EA-SOC | 0 | No concerns | Low risk | No concerns | Some concerns | Some concerns | Some concerns |
| EA:EyeAcu-N-SOC | 0 | No concerns | Low risk | No concerns | Some concerns | No concerns | Some concerns |
| EA:N-SOC | 0 | No concerns | Low risk | No concerns | Some concerns | Some concerns | Some concerns |
| EA:ScA-C-SOC | 0 | No concerns | Low risk | No concerns | No concerns | No concerns | Some concerns |
| EA:ScA-N-SOC | 0 | No concerns | Low risk | No concerns | No concerns | No concerns | Some concerns |
| EA:ScA-SOC | 0 | No concerns | Low risk | No concerns | No concerns | Some concerns | Some concerns |
| EA-N-SOC:EyeAcu-N-SOC | 0 | No concerns | Low risk | No concerns | Some concerns | No concerns | Some concerns |
| EA-N-SOC:SOC | 0 | No concerns | Low risk | No concerns | No concerns | No concerns | Some concerns |
| EA-N-SOC:ScA-C-SOC | 0 | No concerns | Low risk | No concerns | Major concerns | No concerns | Some concerns |
| EA-N-SOC:ScA-N-SOC | 0 | No concerns | Low risk | No concerns | Major concerns | No concerns | Some concerns |
| EA-N-SOC:ScA-SOC | 0 | No concerns | Low risk | No concerns | Some concerns | No concerns | Some concerns |
| EA-SOC:EyeAcu-N-SOC | 0 | No concerns | Low risk | No concerns | Some concerns | Some concerns | Some concerns |
| EA-SOC:ScA-C-SOC | 0 | No concerns | Low risk | No concerns | No concerns | Some concerns | Some concerns |
| EA-SOC:ScA-N-SOC | 0 | No concerns | Low risk | No concerns | No concerns | No concerns | Some concerns |
| EA-SOC:ScA-SOC | 0 | No concerns | Low risk | No concerns | No concerns | Some concerns | Some concerns |
| EyeAcu-N-SOC:ScA-C-SOC | 0 | No concerns | Low risk | No concerns | Some concerns | No concerns | Some concerns |
| EyeAcu-N-SOC:ScA-N-SOC | 0 | No concerns | Low risk | No concerns | Some concerns | No concerns | Some concerns |
| EyeAcu-N-SOC:ScA-SOC | 0 | No concerns | Low risk | No concerns | Some concerns | No concerns | Some concerns |
| N-SOC:ScA-C-SOC | 0 | No concerns | Low risk | No concerns | Some concerns | No concerns | Some concerns |
| ScA-C-SOC:SOC | 0 | No concerns | Low risk | No concerns | No concerns | No concerns | Some concerns |
| ScA-N-SOC:SOC | 0 | No concerns | Low risk | No concerns | No concerns | No concerns | Some concerns |
| ScA-C-SOC:ScA-N-SOC | 0 | No concerns | Low risk | No concerns | Some concerns | Some concerns | Some concerns |

Table S26. GRADE Assessment for MMSE

| **Comparison** | **Number of studies** | **Within-study bias** | **Reporting bias** | **Indirectness** | **Imprecision** | **Heterogeneity** | **Incoherence** | **Confidence rating** |
| --- | --- | --- | --- | --- | --- | --- | --- | --- |
| BA:BA-C-SOC | 1 | No concerns | Low risk | No concerns | Major concerns | No concerns | No concerns | Low |
| BA:C | 1 | No concerns | Low risk | No concerns | Some concerns | No concerns | No concerns | Moderate |
| BA:SOC | 1 | No concerns | Low risk | No concerns | Major concerns | No concerns | No concerns | Low |
| BA-C:C | 1 | No concerns | Low risk | No concerns | No concerns | Some concerns | No concerns | Moderate |
| BA-C-SOC:C | 1 | No concerns | Low risk | No concerns | Major concerns | No concerns | No concerns | Low |
| BA-C-SOC:C-SOC | 3 | No concerns | Low risk | No concerns | Some concerns | Some concerns | No concerns | Low |
| BA-C-SOC:SOC | 1 | No concerns | Low risk | No concerns | No concerns | Major concerns | No concerns | Low |
| BA-SOC:SOC | 11 | No concerns | Low risk | No concerns | No concerns | Major concerns | Major concerns | Very low |
| BA-SOC:ScA-SOC | 2 | No concerns | Low risk | No concerns | Some concerns | No concerns | Major concerns | Very low |
| C:SOC | 1 | No concerns | Low risk | No concerns | Major concerns | No concerns | No concerns | Low |
| C-SOC:ScA-C-SOC | 4 | No concerns | Low risk | No concerns | Some concerns | Some concerns | No concerns | Low |
| C-SOC:ScA-SOC | 1 | No concerns | Low risk | No concerns | Major concerns | No concerns | No concerns | Low |
| EA-N-SOC:EA-SOC | 1 | No concerns | Low risk | No concerns | Major concerns | No concerns | No concerns | Low |
| EA-N-SOC:N-SOC | 1 | No concerns | Low risk | No concerns | Some concerns | Some concerns | No concerns | Low |
| EA-SOC:N-SOC | 1 | No concerns | Low risk | No concerns | Major concerns | No concerns | No concerns | Low |
| EA-SOC:SOC | 13 | No concerns | Low risk | No concerns | No concerns | Some concerns | No concerns | Moderate |
| EyeAcu-N-SOC:N-SOC | 1 | No concerns | Low risk | No concerns | Some concerns | No concerns | No concerns | Moderate |
| EyeAcu-N-SOC:SOC | 1 | No concerns | Low risk | No concerns | Some concerns | Some concerns | No concerns | Low |
| N-SOC:SOC | 1 | No concerns | Low risk | No concerns | No concerns | Major concerns | No concerns | Low |
| N-SOC:ScA-N-SOC | 4 | No concerns | Low risk | No concerns | No concerns | Major concerns | No concerns | Low |
| N-SOC:ScA-SOC | 2 | No concerns | Low risk | No concerns | Some concerns | No concerns | No concerns | Moderate |
| ScA-BA-SOC:SOC | 1 | Some concerns | Low risk | No concerns | Major concerns | No concerns | No concerns | Very low |
| ScA-C-SOC:SOC | 1 | Some concerns | Low risk | No concerns | No concerns | Major concerns | No concerns | Very low |
| ScA-SOC:SOC | 4 | No concerns | Low risk | No concerns | No concerns | Major concerns | No concerns | Low |
| ScAsim-C-SOC:SOC | 1 | Some concerns | Low risk | No concerns | Some concerns | Some concerns | No concerns | Very low |
| ScA-C-SOC:ScA-SOC | 1 | No concerns | Low risk | No concerns | Major concerns | No concerns | No concerns | Low |
| ScA-C-SOC:ScAsim-C-SOC | 1 | Some concerns | Low risk | No concerns | Some concerns | No concerns | No concerns | Low |
| ScA-N-SOC:ScA-SOC | 3 | No concerns | Low risk | No concerns | No concerns | Major concerns | No concerns | Low |
| BA:BA-C | 0 | No concerns | Low risk | No concerns | Some concerns | Some concerns | No concerns | Low |
| BA:BA-SOC | 0 | No concerns | Low risk | No concerns | Some concerns | No concerns | No concerns | Moderate |
| BA:C-SOC | 0 | No concerns | Low risk | No concerns | Major concerns | No concerns | No concerns | Low |
| BA:EA-N-SOC | 0 | No concerns | Low risk | No concerns | Major concerns | No concerns | No concerns | Low |
| BA:EA-SOC | 0 | No concerns | Low risk | No concerns | Some concerns | No concerns | No concerns | Moderate |
| BA:EyeAcu-N-SOC | 0 | No concerns | Low risk | No concerns | Some concerns | No concerns | No concerns | Moderate |
| BA:N-SOC | 0 | No concerns | Low risk | No concerns | Major concerns | No concerns | No concerns | Low |
| BA:ScA-BA-SOC | 0 | No concerns | Low risk | No concerns | Some concerns | No concerns | No concerns | Moderate |
| BA:ScA-C-SOC | 0 | No concerns | Low risk | No concerns | Major concerns | No concerns | No concerns | Low |
| BA:ScA-N-SOC | 0 | No concerns | Low risk | No concerns | Some concerns | Some concerns | No concerns | Low |
| BA:ScA-SOC | 0 | No concerns | Low risk | No concerns | Some concerns | No concerns | No concerns | Moderate |
| BA:ScAsim-C-SOC | 0 | No concerns | Low risk | No concerns | Major concerns | No concerns | No concerns | Low |
| BA-C:BA-C-SOC | 0 | No concerns | Low risk | No concerns | Some concerns | No concerns | No concerns | Moderate |
| BA-C:BA-SOC | 0 | No concerns | Low risk | No concerns | Major concerns | No concerns | No concerns | Low |
| BA-C:C-SOC | 0 | No concerns | Low risk | No concerns | Some concerns | No concerns | No concerns | Moderate |
| BA-C:EA-N-SOC | 0 | No concerns | Low risk | No concerns | Major concerns | No concerns | No concerns | Low |
| BA-C:EA-SOC | 0 | No concerns | Low risk | No concerns | Major concerns | No concerns | No concerns | Low |
| BA-C:EyeAcu-N-SOC | 0 | No concerns | Low risk | No concerns | Some concerns | No concerns | No concerns | Moderate |
| BA-C:N-SOC | 0 | No concerns | Low risk | No concerns | Major concerns | No concerns | No concerns | Low |
| BA-C:SOC | 0 | No concerns | Low risk | No concerns | No concerns | Some concerns | No concerns | Moderate |
| BA-C:ScA-BA-SOC | 0 | No concerns | Low risk | No concerns | Some concerns | No concerns | No concerns | Moderate |
| BA-C:ScA-C-SOC | 0 | No concerns | Low risk | No concerns | Major concerns | No concerns | No concerns | Low |
| BA-C:ScA-N-SOC | 0 | No concerns | Low risk | No concerns | Some concerns | No concerns | No concerns | Moderate |
| BA-C:ScA-SOC | 0 | No concerns | Low risk | No concerns | Major concerns | No concerns | No concerns | Low |
| BA-C:ScAsim-C-SOC | 0 | No concerns | Low risk | No concerns | Some concerns | No concerns | No concerns | Moderate |
| BA-C-SOC:BA-SOC | 0 | No concerns | Low risk | No concerns | Some concerns | No concerns | No concerns | Moderate |
| BA-C-SOC:EA-N-SOC | 0 | No concerns | Low risk | No concerns | Major concerns | No concerns | No concerns | Low |
| BA-C-SOC:EA-SOC | 0 | No concerns | Low risk | No concerns | Some concerns | No concerns | No concerns | Moderate |
| BA-C-SOC:EyeAcu-N-SOC | 0 | No concerns | Low risk | No concerns | Some concerns | No concerns | No concerns | Moderate |
| BA-C-SOC:N-SOC | 0 | No concerns | Low risk | No concerns | Major concerns | No concerns | No concerns | Low |
| BA-C-SOC:ScA-BA-SOC | 0 | No concerns | Low risk | No concerns | Some concerns | No concerns | No concerns | Moderate |
| BA-C-SOC:ScA-C-SOC | 0 | No concerns | Low risk | No concerns | Major concerns | No concerns | No concerns | Low |
| BA-C-SOC:ScA-N-SOC | 0 | No concerns | Low risk | No concerns | Some concerns | No concerns | No concerns | Moderate |
| BA-C-SOC:ScA-SOC | 0 | No concerns | Low risk | No concerns | Some concerns | No concerns | No concerns | Moderate |
| BA-C-SOC:ScAsim-C-SOC | 0 | No concerns | Low risk | No concerns | Major concerns | No concerns | No concerns | Low |
| BA-SOC:C | 0 | No concerns | Low risk | No concerns | Some concerns | No concerns | No concerns | Moderate |
| BA-SOC:C-SOC | 0 | No concerns | Low risk | No concerns | Some concerns | No concerns | No concerns | Moderate |
| BA-SOC:EA-N-SOC | 0 | No concerns | Low risk | No concerns | Major concerns | No concerns | No concerns | Low |
| BA-SOC:EA-SOC | 0 | No concerns | Low risk | No concerns | Some concerns | Some concerns | No concerns | Low |
| BA-SOC:EyeAcu-N-SOC | 0 | No concerns | Low risk | No concerns | Major concerns | No concerns | No concerns | Low |
| BA-SOC:N-SOC | 0 | No concerns | Low risk | No concerns | Some concerns | No concerns | No concerns | Moderate |
| BA-SOC:ScA-BA-SOC | 0 | Some concerns | Low risk | No concerns | Some concerns | No concerns | No concerns | Low |
| BA-SOC:ScA-C-SOC | 0 | No concerns | Low risk | No concerns | Major concerns | No concerns | No concerns | Low |
| BA-SOC:ScA-N-SOC | 0 | No concerns | Low risk | No concerns | No concerns | Major concerns | No concerns | Low |
| BA-SOC:ScAsim-C-SOC | 0 | Some concerns | Low risk | No concerns | Some concerns | No concerns | No concerns | Low |
| C:C-SOC | 0 | No concerns | Low risk | No concerns | Major concerns | No concerns | No concerns | Low |
| C:EA-N-SOC | 0 | No concerns | Low risk | No concerns | Some concerns | No concerns | No concerns | Moderate |
| C:EA-SOC | 0 | No concerns | Low risk | No concerns | Major concerns | No concerns | No concerns | Low |
| C:EyeAcu-N-SOC | 0 | No concerns | Low risk | No concerns | Major concerns | No concerns | No concerns | Low |
| C:N-SOC | 0 | No concerns | Low risk | No concerns | Some concerns | No concerns | No concerns | Moderate |
| C:ScA-BA-SOC | 0 | No concerns | Low risk | No concerns | Major concerns | No concerns | No concerns | Low |
| C:ScA-C-SOC | 0 | No concerns | Low risk | No concerns | Some concerns | No concerns | No concerns | Moderate |
| C:ScA-N-SOC | 0 | No concerns | Low risk | No concerns | Some concerns | Some concerns | No concerns | Low |
| C:ScA-SOC | 0 | No concerns | Low risk | No concerns | Major concerns | No concerns | No concerns | Low |
| C:ScAsim-C-SOC | 0 | No concerns | Low risk | No concerns | Some concerns | No concerns | No concerns | Moderate |
| C-SOC:EA-N-SOC | 0 | No concerns | Low risk | No concerns | Major concerns | No concerns | No concerns | Low |
| C-SOC:EA-SOC | 0 | No concerns | Low risk | No concerns | Some concerns | No concerns | No concerns | Moderate |
| C-SOC:EyeAcu-N-SOC | 0 | No concerns | Low risk | No concerns | Some concerns | No concerns | No concerns | Moderate |
| C-SOC:N-SOC | 0 | No concerns | Low risk | No concerns | Major concerns | No concerns | No concerns | Low |
| C-SOC:SOC | 0 | No concerns | Low risk | No concerns | Some concerns | Some concerns | No concerns | Low |
| C-SOC:ScA-BA-SOC | 0 | Some concerns | Low risk | No concerns | Major concerns | No concerns | No concerns | Very low |
| C-SOC:ScA-N-SOC | 0 | No concerns | Low risk | No concerns | No concerns | Major concerns | No concerns | Low |
| C-SOC:ScAsim-C-SOC | 0 | Some concerns | Low risk | No concerns | Some concerns | No concerns | No concerns | Low |
| EA-N-SOC:EyeAcu-N-SOC | 0 | No concerns | Low risk | No concerns | Major concerns | No concerns | No concerns | Low |
| EA-N-SOC:SOC | 0 | No concerns | Low risk | No concerns | No concerns | Some concerns | No concerns | Moderate |
| EA-N-SOC:ScA-BA-SOC | 0 | No concerns | Low risk | No concerns | Major concerns | No concerns | No concerns | Low |
| EA-N-SOC:ScA-C-SOC | 0 | No concerns | Low risk | No concerns | Some concerns | No concerns | No concerns | Moderate |
| EA-N-SOC:ScA-N-SOC | 0 | No concerns | Low risk | No concerns | Major concerns | No concerns | No concerns | Low |
| EA-N-SOC:ScA-SOC | 0 | No concerns | Low risk | No concerns | Major concerns | No concerns | No concerns | Low |
| EA-N-SOC:ScAsim-C-SOC | 0 | No concerns | Low risk | No concerns | Some concerns | No concerns | No concerns | Moderate |
| EA-SOC:EyeAcu-N-SOC | 0 | No concerns | Low risk | No concerns | Major concerns | No concerns | No concerns | Low |
| EA-SOC:ScA-BA-SOC | 0 | Some concerns | Low risk | No concerns | Some concerns | No concerns | No concerns | Low |
| EA-SOC:ScA-C-SOC | 0 | No concerns | Low risk | No concerns | Some concerns | No concerns | No concerns | Moderate |
| EA-SOC:ScA-N-SOC | 0 | No concerns | Low risk | No concerns | Some concerns | Some concerns | No concerns | Low |
| EA-SOC:ScA-SOC | 0 | No concerns | Low risk | No concerns | Some concerns | No concerns | No concerns | Moderate |
| EA-SOC:ScAsim-C-SOC | 0 | Some concerns | Low risk | No concerns | Major concerns | No concerns | No concerns | Very low |
| EyeAcu-N-SOC:ScA-BA-SOC | 0 | No concerns | Low risk | No concerns | Major concerns | No concerns | No concerns | Low |
| EyeAcu-N-SOC:ScA-C-SOC | 0 | No concerns | Low risk | No concerns | Some concerns | No concerns | No concerns | Moderate |
| EyeAcu-N-SOC:ScA-N-SOC | 0 | No concerns | Low risk | No concerns | Major concerns | No concerns | No concerns | Low |
| EyeAcu-N-SOC:ScA-SOC | 0 | No concerns | Low risk | No concerns | Some concerns | No concerns | No concerns | Moderate |
| EyeAcu-N-SOC:ScAsim-C-SOC | 0 | No concerns | Low risk | No concerns | Some concerns | No concerns | No concerns | Moderate |
| N-SOC:ScA-BA-SOC | 0 | No concerns | Low risk | No concerns | Major concerns | No concerns | No concerns | Low |
| N-SOC:ScA-C-SOC | 0 | No concerns | Low risk | No concerns | Some concerns | No concerns | No concerns | Moderate |
| N-SOC:ScAsim-C-SOC | 0 | No concerns | Low risk | No concerns | Some concerns | No concerns | No concerns | Moderate |
| ScA-N-SOC:SOC | 0 | No concerns | Low risk | No concerns | No concerns | No concerns | No concerns | High |
| ScA-BA-SOC:ScA-C-SOC | 0 | Some concerns | Low risk | No concerns | Major concerns | No concerns | No concerns | Very low |
| ScA-BA-SOC:ScA-N-SOC | 0 | No concerns | Low risk | No concerns | Some concerns | Some concerns | No concerns | Low |
| ScA-BA-SOC:ScA-SOC | 0 | Some concerns | Low risk | No concerns | Major concerns | No concerns | No concerns | Very low |
| ScA-BA-SOC:ScAsim-C-SOC | 0 | Some concerns | Low risk | No concerns | Some concerns | No concerns | No concerns | Low |
| ScA-C-SOC:ScA-N-SOC | 0 | No concerns | Low risk | No concerns | Some concerns | No concerns | No concerns | Moderate |
| ScA-N-SOC:ScAsim-C-SOC | 0 | No concerns | Low risk | No concerns | Some concerns | No concerns | No concerns | Moderate |
| ScAsim-C-SOC:ScA-SOC | 0 | Some concerns | Low risk | No concerns | Major concerns | No concerns | No concerns | Very low |

Table S27. GRADE Assessment for BI

| **Comparison** | **Number of studies** | **Within-study bias** | **Reporting bias** | **Indirectness** | **Imprecision** | **Heterogeneity** | **Incoherence** | **Confidence rating** |
| --- | --- | --- | --- | --- | --- | --- | --- | --- |
| B-SOC:SOC | 1 | No concerns | Low risk | No concerns | Some concerns | Some concerns | No concerns | Low |
| BA-M-SOC:SOC | 1 | No concerns | Low risk | No concerns | No concerns | Some concerns | No concerns | Moderate |
| BA-N-SOC:N-SOC | 1 | No concerns | Low risk | No concerns | No concerns | Some concerns | No concerns | Moderate |
| BA-N-SOC:SOC | 1 | No concerns | Low risk | No concerns | No concerns | No concerns | No concerns | High |
| BA-SOC:SOC | 3 | Some concerns | Low risk | No concerns | No concerns | Some concerns | No concerns | Low |
| BA-SOC:ScA-SOC | 1 | No concerns | Low risk | No concerns | Some concerns | No concerns | No concerns | Moderate |
| EA-SOC:SOC | 4 | No concerns | Low risk | No concerns | No concerns | Some concerns | No concerns | Moderate |
| N-SOC:SOC | 1 | No concerns | Low risk | No concerns | Some concerns | Some concerns | No concerns | Low |
| N-SOC:ScA-N-SOC | 1 | No concerns | Low risk | No concerns | No concerns | Some concerns | No concerns | Moderate |
| ScA-BA-SOC:SOC | 1 | Some concerns | Low risk | No concerns | No concerns | Some concerns | No concerns | Low |
| ScA-E-SOC:SOC | 1 | No concerns | Low risk | No concerns | Major concerns | No concerns | No concerns | Low |
| ScA-SOC:SOC | 4 | No concerns | Low risk | No concerns | No concerns | No concerns | No concerns | High |
| BA-M-SOC:B-SOC | 0 | No concerns | Low risk | No concerns | Some concerns | Some concerns | No concerns | Low |
| BA-N-SOC:B-SOC | 0 | No concerns | Low risk | No concerns | Some concerns | Some concerns | No concerns | Low |
| BA-SOC:B-SOC | 0 | No concerns | Low risk | No concerns | Major concerns | No concerns | No concerns | Low |
| B-SOC:EA-SOC | 0 | No concerns | Low risk | No concerns | Major concerns | No concerns | No concerns | Low |
| B-SOC:N-SOC | 0 | No concerns | Low risk | No concerns | Major concerns | No concerns | No concerns | Low |
| B-SOC:ScA-BA-SOC | 0 | No concerns | Low risk | No concerns | Major concerns | No concerns | No concerns | Low |
| B-SOC:ScA-E-SOC | 0 | No concerns | Low risk | No concerns | Some concerns | Some concerns | No concerns | Low |
| B-SOC:ScA-N-SOC | 0 | No concerns | Low risk | No concerns | Some concerns | Some concerns | No concerns | Low |
| B-SOC:ScA-SOC | 0 | No concerns | Low risk | No concerns | Some concerns | Some concerns | No concerns | Low |
| BA-M-SOC:BA-N-SOC | 0 | No concerns | Low risk | No concerns | Major concerns | No concerns | No concerns | Low |
| BA-M-SOC:BA-SOC | 0 | No concerns | Low risk | No concerns | Some concerns | Some concerns | No concerns | Low |
| BA-M-SOC:EA-SOC | 0 | No concerns | Low risk | No concerns | Some concerns | Some concerns | No concerns | Low |
| BA-M-SOC:N-SOC | 0 | No concerns | Low risk | No concerns | Major concerns | No concerns | No concerns | Low |
| BA-M-SOC:ScA-BA-SOC | 0 | No concerns | Low risk | No concerns | Major concerns | No concerns | No concerns | Low |
| BA-M-SOC:ScA-E-SOC | 0 | No concerns | Low risk | No concerns | Some concerns | No concerns | No concerns | Moderate |
| BA-M-SOC:ScA-N-SOC | 0 | No concerns | Low risk | No concerns | Major concerns | No concerns | No concerns | Low |
| BA-M-SOC:ScA-SOC | 0 | No concerns | Low risk | No concerns | Major concerns | No concerns | No concerns | Low |
| BA-N-SOC:BA-SOC | 0 | No concerns | Low risk | No concerns | Some concerns | No concerns | No concerns | Moderate |
| BA-N-SOC:EA-SOC | 0 | No concerns | Low risk | No concerns | Some concerns | No concerns | No concerns | Moderate |
| BA-N-SOC:ScA-BA-SOC | 0 | No concerns | Low risk | No concerns | Major concerns | No concerns | No concerns | Low |
| BA-N-SOC:ScA-E-SOC | 0 | No concerns | Low risk | No concerns | No concerns | Some concerns | No concerns | Moderate |
| BA-N-SOC:ScA-N-SOC | 0 | No concerns | Low risk | No concerns | Major concerns | No concerns | No concerns | Low |
| BA-N-SOC:ScA-SOC | 0 | No concerns | Low risk | No concerns | Major concerns | No concerns | No concerns | Low |
| BA-SOC:EA-SOC | 0 | No concerns | Low risk | No concerns | Some concerns | Some concerns | No concerns | Low |
| BA-SOC:N-SOC | 0 | No concerns | Low risk | No concerns | Major concerns | No concerns | No concerns | Low |
| BA-SOC:ScA-BA-SOC | 0 | Some concerns | Low risk | No concerns | Some concerns | Some concerns | No concerns | Very low |
| BA-SOC:ScA-E-SOC | 0 | No concerns | Low risk | No concerns | Some concerns | Some concerns | No concerns | Low |
| BA-SOC:ScA-N-SOC | 0 | No concerns | Low risk | No concerns | Some concerns | Some concerns | No concerns | Low |
| EA-SOC:N-SOC | 0 | No concerns | Low risk | No concerns | Major concerns | No concerns | No concerns | Low |
| EA-SOC:ScA-BA-SOC | 0 | Some concerns | Low risk | No concerns | Some concerns | Some concerns | No concerns | Very low |
| EA-SOC:ScA-E-SOC | 0 | No concerns | Low risk | No concerns | Some concerns | Some concerns | No concerns | Low |
| EA-SOC:ScA-N-SOC | 0 | No concerns | Low risk | No concerns | Some concerns | Some concerns | No concerns | Low |
| EA-SOC:ScA-SOC | 0 | No concerns | Low risk | No concerns | Some concerns | No concerns | No concerns | Moderate |
| N-SOC:ScA-BA-SOC | 0 | No concerns | Low risk | No concerns | Major concerns | No concerns | No concerns | Low |
| N-SOC:ScA-E-SOC | 0 | No concerns | Low risk | No concerns | Some concerns | Some concerns | No concerns | Low |
| N-SOC:ScA-SOC | 0 | No concerns | Low risk | No concerns | Some concerns | Some concerns | No concerns | Low |
| ScA-N-SOC:SOC | 0 | No concerns | Low risk | No concerns | No concerns | No concerns | No concerns | High |
| ScA-BA-SOC:ScA-E-SOC | 0 | No concerns | Low risk | No concerns | Some concerns | No concerns | No concerns | Moderate |
| ScA-BA-SOC:ScA-N-SOC | 0 | No concerns | Low risk | No concerns | Major concerns | No concerns | No concerns | Low |
| ScA-BA-SOC:ScA-SOC | 0 | Some concerns | Low risk | No concerns | Major concerns | No concerns | No concerns | Very low |
| ScA-E-SOC:ScA-N-SOC | 0 | No concerns | Low risk | No concerns | No concerns | Some concerns | No concerns | Moderate |
| ScA-E-SOC:ScA-SOC | 0 | No concerns | Low risk | No concerns | No concerns | Some concerns | No concerns | Moderate |
| ScA-N-SOC:ScA-SOC | 0 | No concerns | Low risk | No concerns | Major concerns | No concerns | No concerns | Low |

Table S28. GRADE Assessment for TER

| **Comparison** | **Number of studies** | **Within-study bias** | **Reporting bias** | **Indirectness** | **Imprecision** | **Heterogeneity** | **Incoherence** | **Confidence rating** |
| --- | --- | --- | --- | --- | --- | --- | --- | --- |
| B-SOC:SOC | 1 | No concerns | Low risk | No concerns | No concerns | No concerns | No concerns | High |
| BA-C-SOC:C-SOC | 1 | Some concerns | Low risk | No concerns | Some concerns | No concerns | No concerns | Low |
| BA-M-SOC:SOC | 1 | No concerns | Low risk | No concerns | Some concerns | Some concerns | No concerns | Low |
| BA-SOC:SOC | 7 | No concerns | Low risk | No concerns | No concerns | No concerns | No concerns | High |
| BA-SOC:ScA-SOC | 1 | Some concerns | Low risk | No concerns | Major concerns | No concerns | No concerns | Very low |
| C-SOC:E-C-SOC | 1 | No concerns | Low risk | No concerns | No concerns | Some concerns | No concerns | Moderate |
| C-SOC:EA-C-SOC | 3 | No concerns | Low risk | No concerns | No concerns | No concerns | No concerns | High |
| C-SOC:M-C-SOC | 1 | No concerns | Low risk | No concerns | Major concerns | No concerns | No concerns | Low |
| C-SOC:ScA-C-SOC | 1 | No concerns | Low risk | No concerns | Major concerns | No concerns | No concerns | Low |
| C-SOC:ScA-E-C-SOC | 1 | No concerns | Low risk | No concerns | No concerns | No concerns | No concerns | High |
| EA-SOC:SOC | 3 | No concerns | Low risk | No concerns | No concerns | No concerns | No concerns | High |
| ScA-B-SOC:SOC | 1 | No concerns | Low risk | No concerns | No concerns | Some concerns | No concerns | Moderate |
| ScA-BA-SOC:SOC | 1 | Some concerns | Low risk | No concerns | No concerns | No concerns | No concerns | Moderate |
| ScA-SOC:SOC | 3 | Some concerns | Low risk | No concerns | No concerns | No concerns | No concerns | Moderate |
| ScA-C-SOC:ScA-SOC | 1 | No concerns | Low risk | No concerns | No concerns | Some concerns | No concerns | Moderate |
| BA-C-SOC:B-SOC | 0 | No concerns | Low risk | No concerns | Major concerns | No concerns | No concerns | Low |
| BA-M-SOC:B-SOC | 0 | No concerns | Low risk | No concerns | Some concerns | No concerns | No concerns | Moderate |
| BA-SOC:B-SOC | 0 | No concerns | Low risk | No concerns | Major concerns | No concerns | No concerns | Low |
| B-SOC:C-SOC | 0 | No concerns | Low risk | No concerns | Major concerns | No concerns | No concerns | Low |
| B-SOC:E-C-SOC | 0 | No concerns | Low risk | No concerns | Some concerns | No concerns | No concerns | Moderate |
| B-SOC:EA-C-SOC | 0 | No concerns | Low risk | No concerns | Major concerns | No concerns | No concerns | Low |
| B-SOC:EA-SOC | 0 | No concerns | Low risk | No concerns | Some concerns | No concerns | No concerns | Moderate |
| B-SOC:M-C-SOC | 0 | No concerns | Low risk | No concerns | Major concerns | No concerns | No concerns | Low |
| B-SOC:ScA-B-SOC | 0 | No concerns | Low risk | No concerns | Major concerns | No concerns | No concerns | Low |
| B-SOC:ScA-BA-SOC | 0 | No concerns | Low risk | No concerns | Some concerns | No concerns | No concerns | Moderate |
| B-SOC:ScA-C-SOC | 0 | No concerns | Low risk | No concerns | Major concerns | No concerns | No concerns | Low |
| B-SOC:ScA-E-C-SOC | 0 | No concerns | Low risk | No concerns | No concerns | Major concerns | No concerns | Low |
| B-SOC:ScA-SOC | 0 | No concerns | Low risk | No concerns | Some concerns | No concerns | No concerns | Moderate |
| BA-C-SOC:BA-M-SOC | 0 | No concerns | Low risk | No concerns | Major concerns | No concerns | No concerns | Low |
| BA-C-SOC:BA-SOC | 0 | No concerns | Low risk | No concerns | No concerns | Some concerns | No concerns | Moderate |
| BA-C-SOC:E-C-SOC | 0 | No concerns | Low risk | No concerns | Major concerns | No concerns | No concerns | Low |
| BA-C-SOC:EA-C-SOC | 0 | No concerns | Low risk | No concerns | Some concerns | No concerns | No concerns | Moderate |
| BA-C-SOC:EA-SOC | 0 | No concerns | Low risk | No concerns | Some concerns | No concerns | No concerns | Moderate |
| BA-C-SOC:M-C-SOC | 0 | No concerns | Low risk | No concerns | Major concerns | No concerns | No concerns | Low |
| BA-C-SOC:SOC | 0 | No concerns | Low risk | No concerns | No concerns | No concerns | No concerns | High |
| BA-C-SOC:ScA-B-SOC | 0 | No concerns | Low risk | No concerns | Major concerns | No concerns | No concerns | Low |
| BA-C-SOC:ScA-BA-SOC | 0 | Some concerns | Low risk | No concerns | Major concerns | No concerns | No concerns | Very low |
| BA-C-SOC:ScA-C-SOC | 0 | No concerns | Low risk | No concerns | Some concerns | No concerns | No concerns | Moderate |
| BA-C-SOC:ScA-E-C-SOC | 0 | No concerns | Low risk | No concerns | Major concerns | No concerns | No concerns | Low |
| BA-C-SOC:ScA-SOC | 0 | No concerns | Low risk | No concerns | Some concerns | Some concerns | No concerns | Low |
| BA-M-SOC:BA-SOC | 0 | No concerns | Low risk | No concerns | Some concerns | No concerns | No concerns | Moderate |
| BA-M-SOC:C-SOC | 0 | No concerns | Low risk | No concerns | Major concerns | No concerns | No concerns | Low |
| BA-M-SOC:E-C-SOC | 0 | No concerns | Low risk | No concerns | Major concerns | No concerns | No concerns | Low |
| BA-M-SOC:EA-C-SOC | 0 | No concerns | Low risk | No concerns | Some concerns | No concerns | No concerns | Moderate |
| BA-M-SOC:EA-SOC | 0 | No concerns | Low risk | No concerns | Major concerns | No concerns | No concerns | Low |
| BA-M-SOC:M-C-SOC | 0 | No concerns | Low risk | No concerns | Some concerns | No concerns | No concerns | Moderate |
| BA-M-SOC:ScA-B-SOC | 0 | No concerns | Low risk | No concerns | Major concerns | No concerns | No concerns | Low |
| BA-M-SOC:ScA-BA-SOC | 0 | No concerns | Low risk | No concerns | Major concerns | No concerns | No concerns | Low |
| BA-M-SOC:ScA-C-SOC | 0 | No concerns | Low risk | No concerns | Some concerns | No concerns | No concerns | Moderate |
| BA-M-SOC:ScA-E-C-SOC | 0 | No concerns | Low risk | No concerns | No concerns | Some concerns | No concerns | Moderate |
| BA-M-SOC:ScA-SOC | 0 | No concerns | Low risk | No concerns | Major concerns | No concerns | No concerns | Low |
| BA-SOC:C-SOC | 0 | No concerns | Low risk | No concerns | Some concerns | No concerns | No concerns | Moderate |
| BA-SOC:E-C-SOC | 0 | No concerns | Low risk | No concerns | No concerns | No concerns | No concerns | High |
| BA-SOC:EA-C-SOC | 0 | No concerns | Low risk | No concerns | No concerns | No concerns | No concerns | High |
| BA-SOC:EA-SOC | 0 | No concerns | Low risk | No concerns | Major concerns | No concerns | No concerns | Low |
| BA-SOC:M-C-SOC | 0 | No concerns | Low risk | No concerns | Major concerns | No concerns | No concerns | Low |
| BA-SOC:ScA-B-SOC | 0 | No concerns | Low risk | No concerns | Some concerns | No concerns | No concerns | Moderate |
| BA-SOC:ScA-BA-SOC | 0 | Some concerns | Low risk | No concerns | Major concerns | No concerns | No concerns | Very low |
| BA-SOC:ScA-C-SOC | 0 | No concerns | Low risk | No concerns | No concerns | No concerns | No concerns | High |
| BA-SOC:ScA-E-C-SOC | 0 | No concerns | Low risk | No concerns | No concerns | No concerns | No concerns | High |
| C-SOC:EA-SOC | 0 | No concerns | Low risk | No concerns | Major concerns | No concerns | No concerns | Low |
| C-SOC:SOC | 0 | No concerns | Low risk | No concerns | No concerns | No concerns | No concerns | High |
| C-SOC:ScA-B-SOC | 0 | No concerns | Low risk | No concerns | Major concerns | No concerns | No concerns | Low |
| C-SOC:ScA-BA-SOC | 0 | No concerns | Low risk | No concerns | Major concerns | No concerns | No concerns | Low |
| C-SOC:ScA-SOC | 0 | No concerns | Low risk | No concerns | Some concerns | No concerns | No concerns | Moderate |
| EA-C-SOC:E-C-SOC | 0 | No concerns | Low risk | No concerns | Major concerns | No concerns | No concerns | Low |
| EA-SOC:E-C-SOC | 0 | No concerns | Low risk | No concerns | Some concerns | Some concerns | No concerns | Low |
| E-C-SOC:M-C-SOC | 0 | No concerns | Low risk | No concerns | Major concerns | No concerns | No concerns | Low |
| E-C-SOC:SOC | 0 | No concerns | Low risk | No concerns | No concerns | No concerns | No concerns | High |
| E-C-SOC:ScA-B-SOC | 0 | No concerns | Low risk | No concerns | Major concerns | No concerns | No concerns | Low |
| E-C-SOC:ScA-BA-SOC | 0 | No concerns | Low risk | No concerns | Some concerns | No concerns | No concerns | Moderate |
| E-C-SOC:ScA-C-SOC | 0 | No concerns | Low risk | No concerns | Major concerns | No concerns | No concerns | Low |
| E-C-SOC:ScA-E-C-SOC | 0 | No concerns | Low risk | No concerns | Major concerns | No concerns | No concerns | Low |
| E-C-SOC:ScA-SOC | 0 | No concerns | Low risk | No concerns | Some concerns | Some concerns | No concerns | Low |
| EA-C-SOC:EA-SOC | 0 | No concerns | Low risk | No concerns | Some concerns | Some concerns | No concerns | Low |
| EA-C-SOC:M-C-SOC | 0 | No concerns | Low risk | No concerns | Some concerns | No concerns | No concerns | Moderate |
| EA-C-SOC:SOC | 0 | No concerns | Low risk | No concerns | No concerns | No concerns | No concerns | High |
| EA-C-SOC:ScA-B-SOC | 0 | No concerns | Low risk | No concerns | Some concerns | Some concerns | No concerns | Low |
| EA-C-SOC:ScA-BA-SOC | 0 | No concerns | Low risk | No concerns | Some concerns | No concerns | No concerns | Moderate |
| EA-C-SOC:ScA-C-SOC | 0 | No concerns | Low risk | No concerns | Major concerns | No concerns | No concerns | Low |
| EA-C-SOC:ScA-E-C-SOC | 0 | No concerns | Low risk | No concerns | Major concerns | No concerns | No concerns | Low |
| EA-C-SOC:ScA-SOC | 0 | No concerns | Low risk | No concerns | No concerns | Some concerns | No concerns | Moderate |
| EA-SOC:M-C-SOC | 0 | No concerns | Low risk | No concerns | Major concerns | No concerns | No concerns | Low |
| EA-SOC:ScA-B-SOC | 0 | No concerns | Low risk | No concerns | Some concerns | No concerns | No concerns | Moderate |
| EA-SOC:ScA-BA-SOC | 0 | No concerns | Low risk | No concerns | Some concerns | No concerns | No concerns | Moderate |
| EA-SOC:ScA-C-SOC | 0 | No concerns | Low risk | No concerns | Some concerns | Some concerns | No concerns | Low |
| EA-SOC:ScA-E-C-SOC | 0 | No concerns | Low risk | No concerns | No concerns | No concerns | No concerns | High |
| EA-SOC:ScA-SOC | 0 | No concerns | Low risk | No concerns | Major concerns | No concerns | No concerns | Low |
| M-C-SOC:SOC | 0 | No concerns | Low risk | No concerns | No concerns | No concerns | No concerns | High |
| M-C-SOC:ScA-B-SOC | 0 | No concerns | Low risk | No concerns | Major concerns | No concerns | No concerns | Low |
| M-C-SOC:ScA-BA-SOC | 0 | No concerns | Low risk | No concerns | Some concerns | No concerns | No concerns | Moderate |
| M-C-SOC:ScA-C-SOC | 0 | No concerns | Low risk | No concerns | Major concerns | No concerns | No concerns | Low |
| M-C-SOC:ScA-E-C-SOC | 0 | No concerns | Low risk | No concerns | Some concerns | No concerns | No concerns | Moderate |
| M-C-SOC:ScA-SOC | 0 | No concerns | Low risk | No concerns | Major concerns | No concerns | No concerns | Low |
| ScA-C-SOC:SOC | 0 | No concerns | Low risk | No concerns | No concerns | No concerns | No concerns | High |
| ScA-E-C-SOC:SOC | 0 | No concerns | Low risk | No concerns | No concerns | No concerns | No concerns | High |
| ScA-BA-SOC:ScA-B-SOC | 0 | No concerns | Low risk | No concerns | Major concerns | No concerns | No concerns | Low |
| ScA-B-SOC:ScA-C-SOC | 0 | No concerns | Low risk | No concerns | Major concerns | No concerns | No concerns | Low |
| ScA-B-SOC:ScA-E-C-SOC | 0 | No concerns | Low risk | No concerns | No concerns | No concerns | No concerns | High |
| ScA-B-SOC:ScA-SOC | 0 | No concerns | Low risk | No concerns | Major concerns | No concerns | No concerns | Low |
| ScA-BA-SOC:ScA-C-SOC | 0 | Some concerns | Low risk | No concerns | Major concerns | No concerns | No concerns | Very low |
| ScA-BA-SOC:ScA-E-C-SOC | 0 | No concerns | Low risk | No concerns | No concerns | No concerns | No concerns | High |
| ScA-BA-SOC:ScA-SOC | 0 | Some concerns | Low risk | No concerns | Major concerns | No concerns | No concerns | Very low |
| ScA-C-SOC:ScA-E-C-SOC | 0 | No concerns | Low risk | No concerns | Some concerns | No concerns | No concerns | Moderate |
| ScA-E-C-SOC:ScA-SOC | 0 | No concerns | Low risk | No concerns | No concerns | No concerns | No concerns | High |


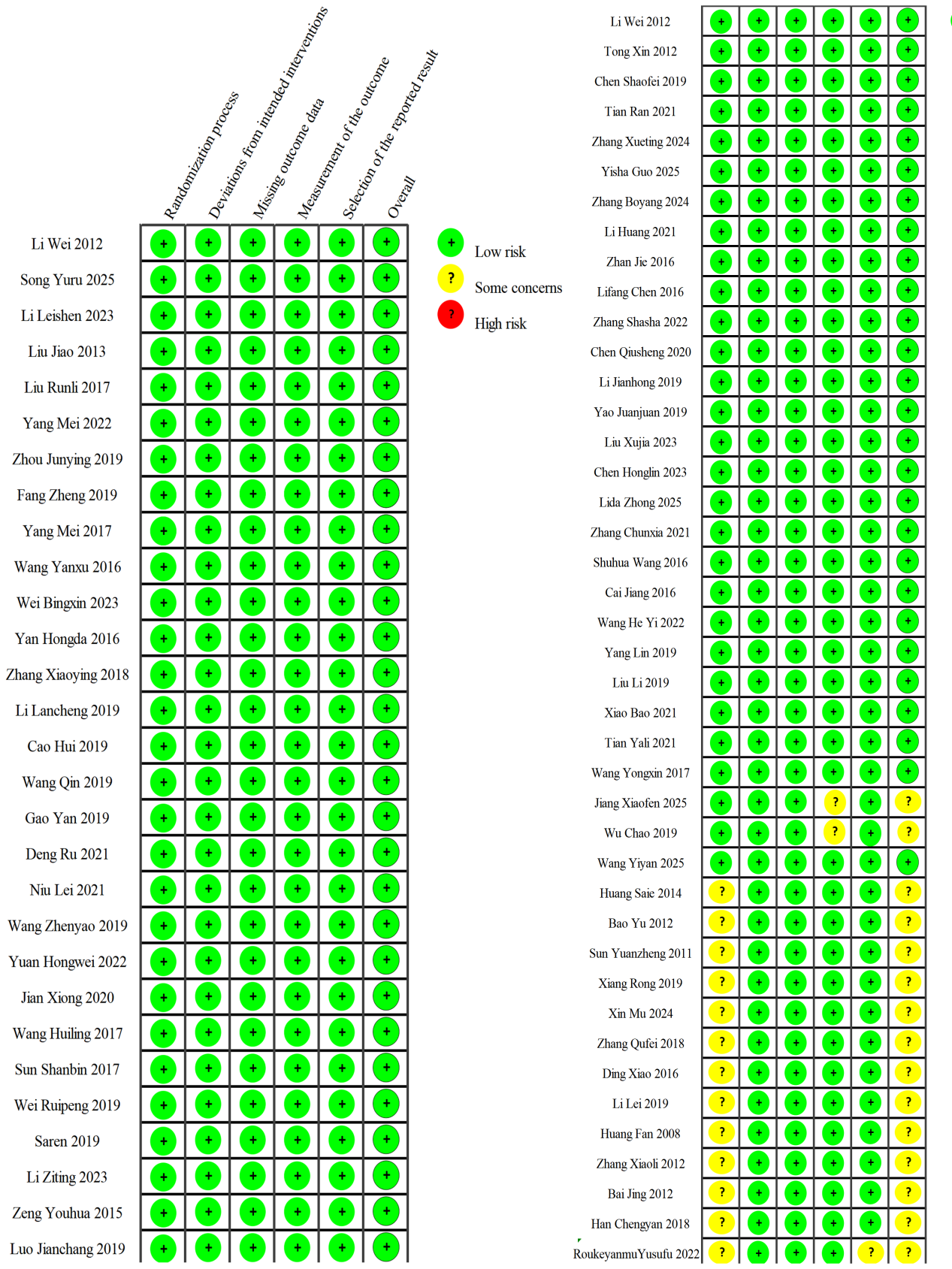


SFIG.1. Risk of Bias Assessment for Randomized Controlled Trials


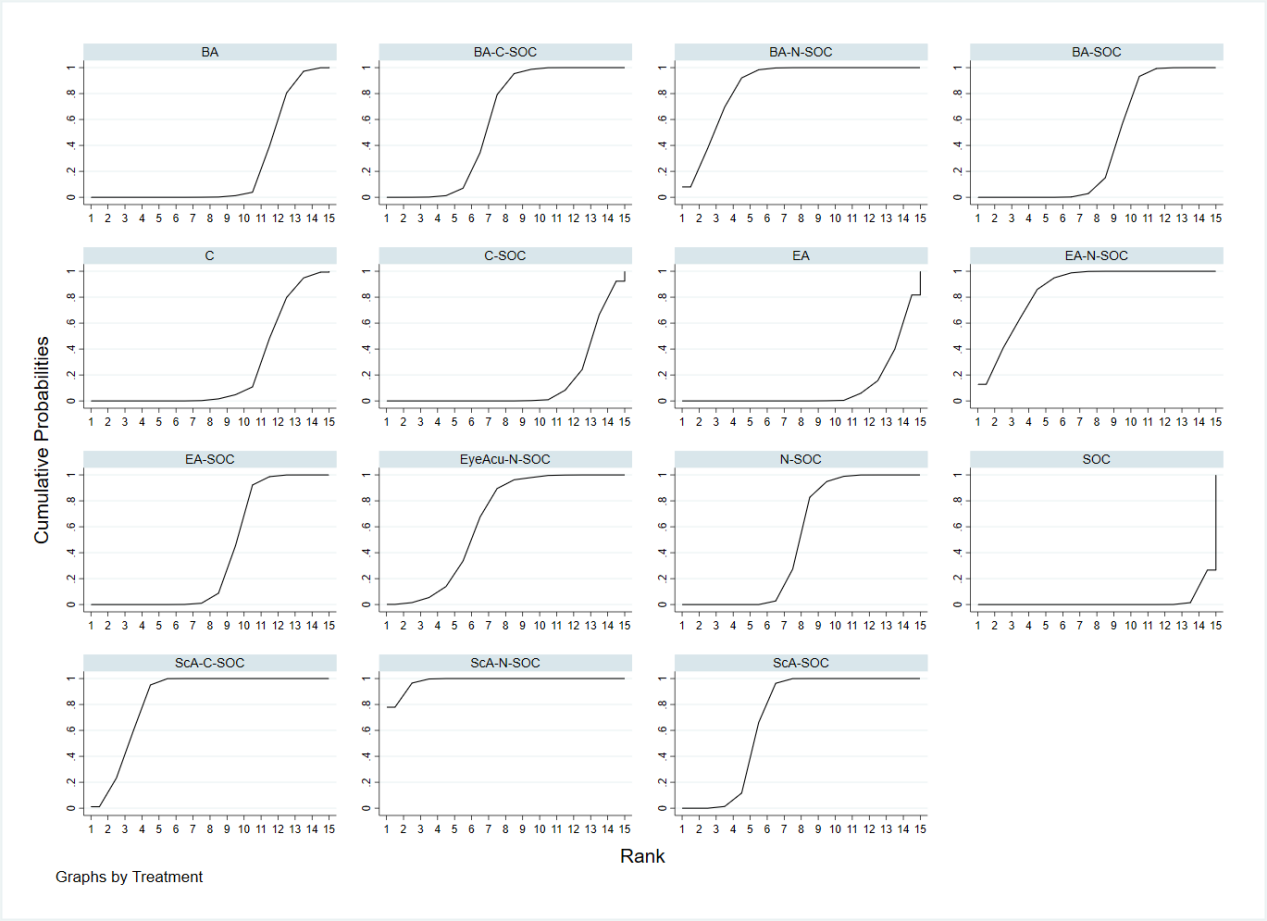


SFIG.2 SCURA Diagram of MOCA


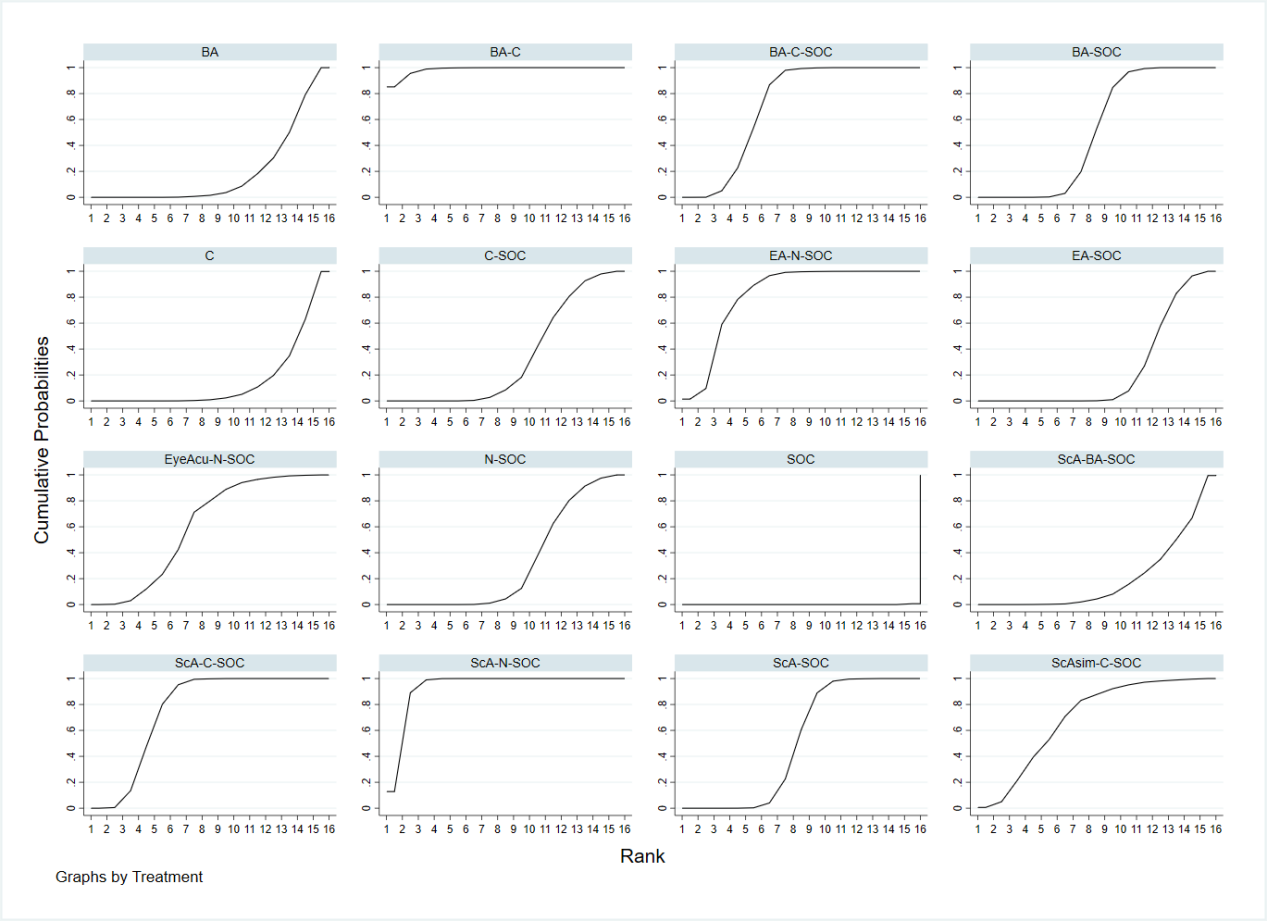


SFIG.3 SCURA Diagram of MMSE


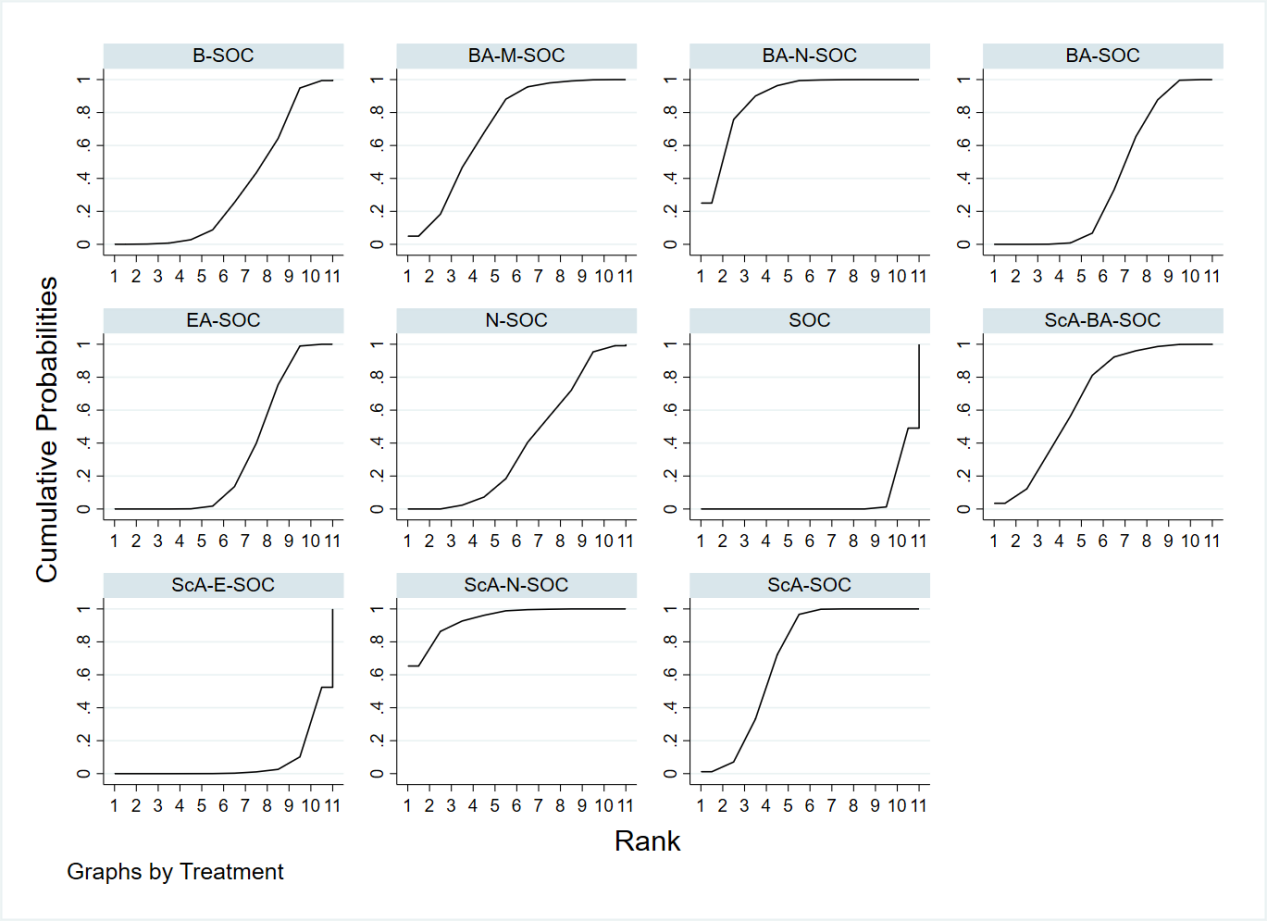


SFIG.4 SCURA Diagram of BI


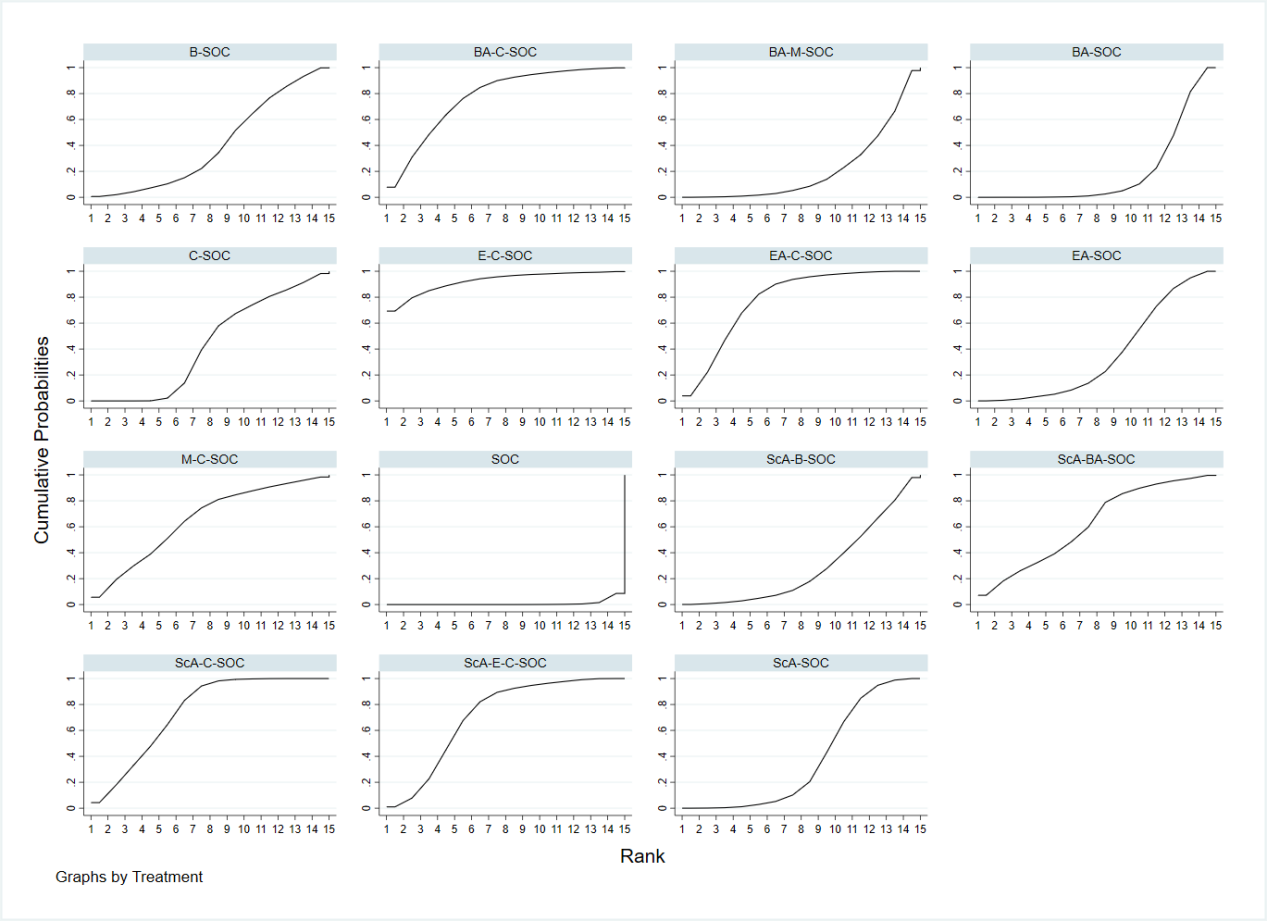


SFIG.5 SCURA Diagram of TER


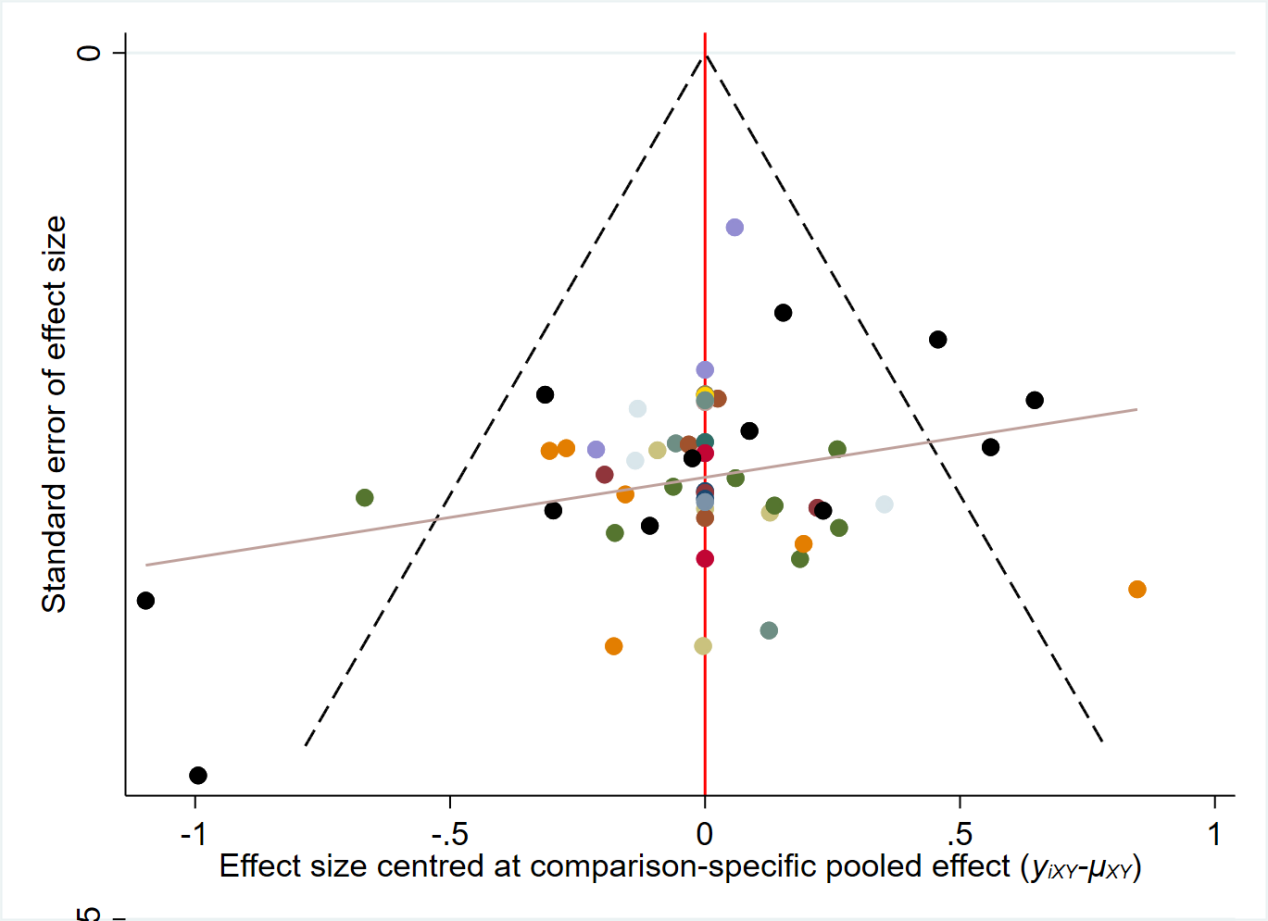


SFIG.6 The funnel plot of MOCA


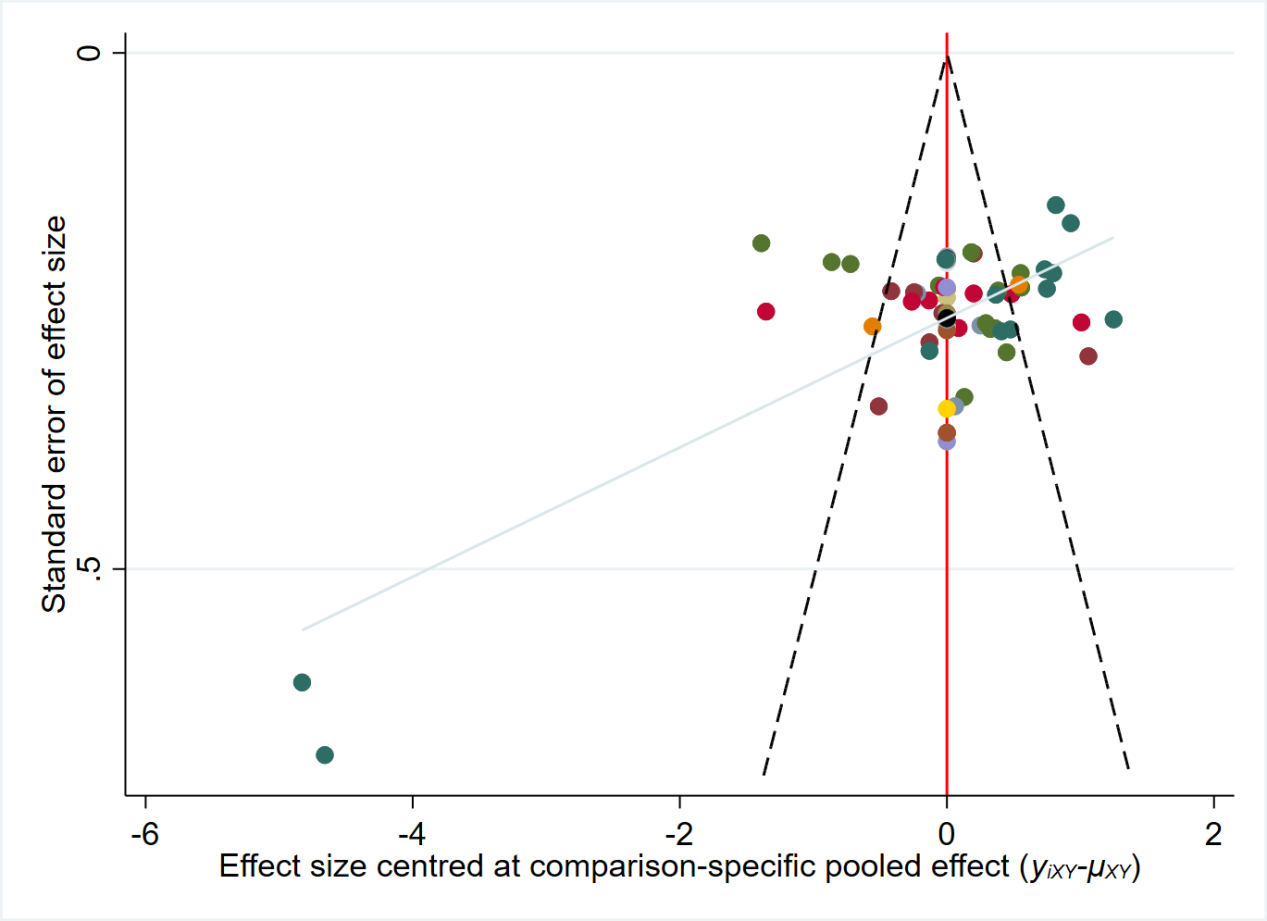


SFIG.7 The funnel plot of MMSE


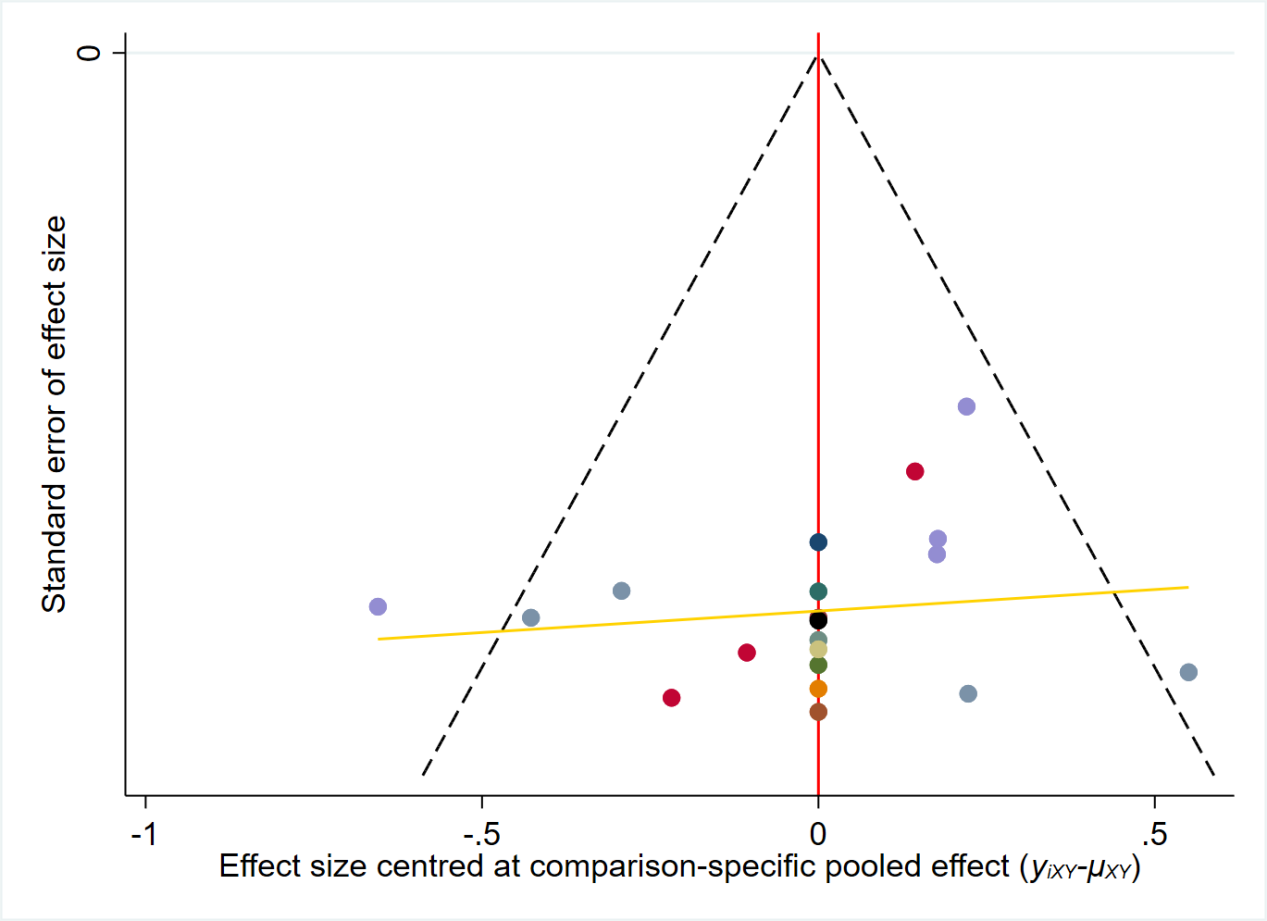


SFIG.8 The funnel plot of BI


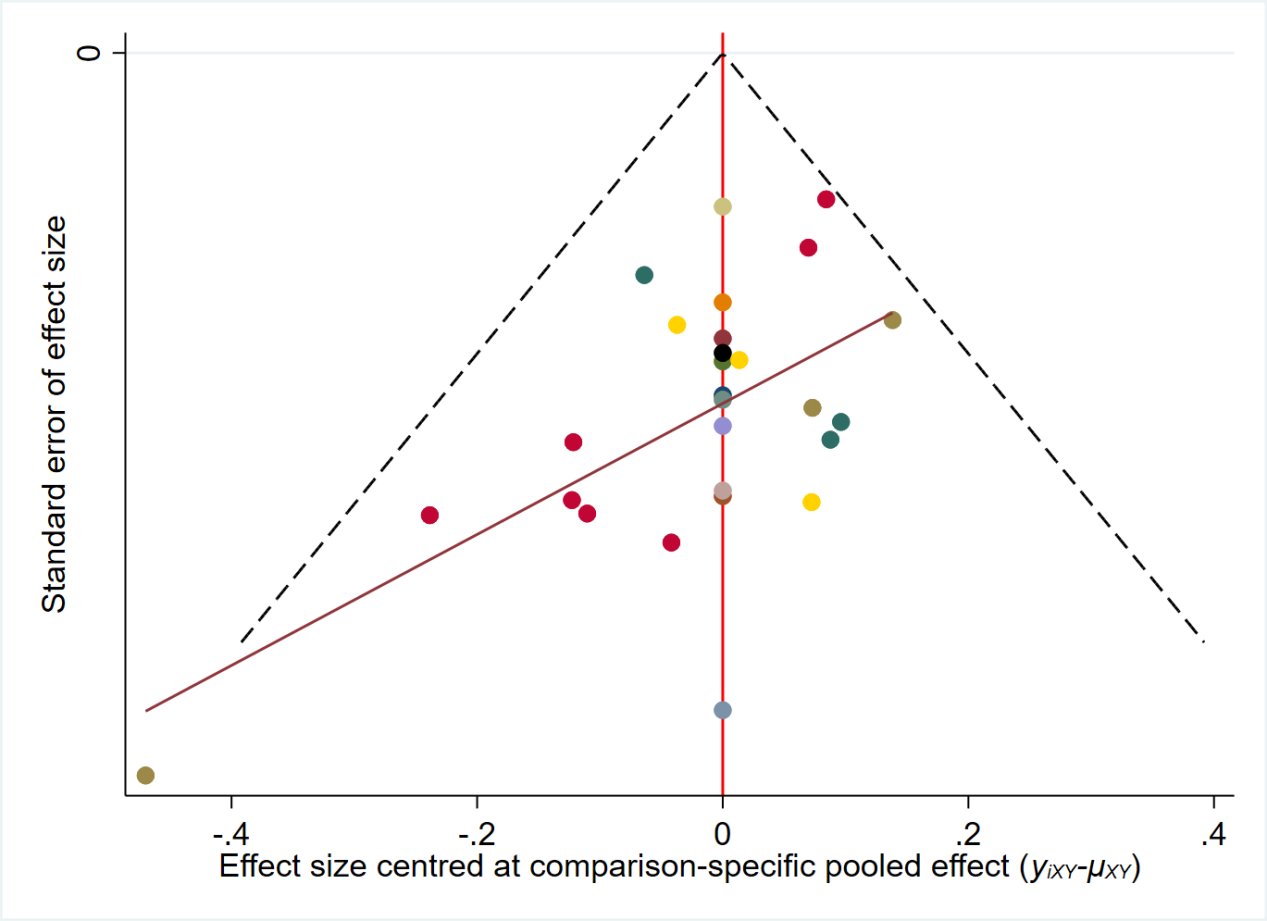


SFIG.9 The funnel plot of TER

[1] Roukeyanmu Yusufu, Mayila Tuerxun, Rena Abudusalamu, Shi W. (2022). Effects of acupuncture on cerebral blood flow, serum S100β, and alkaline phosphatase in patients with mild cognitive impairment during stroke recovery. World Journal of Acupuncture-Moxibustion, 32, 305–309.

[2] Jiang X, Xu Q, Jin Y. (2025). Acupuncture combined with cognitive training for mild-to-moderate post-stroke cognitive impairment: A clinical study. Journal of Acupuncture and Tuina Science, 23, 144–150. [https://doi.org/10.1007/s11726-025-1493-1](https://doi.org/10.1007/s11726-025-1493-1)

[3] Li W, Cheng YH, Yu XG. (2012). Effects of combined acupuncture and medication on mild cognitive impairment after stroke. Chinese Acupuncture & Moxibustion, 32, 3–7. [https://doi.org/10.13703/j.0255-2930.2012.01.005](https://doi.org/10.13703/j.0255-2930.2012.01.005)

[4] Wang YY, Wang DY, Li SW, Dong X, Liu Y, Zhao DX, Xing JJ. (2025). Repetitive transcranial acupuncture for mild cognitive impairment after stroke: A randomized controlled trial. Journal of Clinical Acupuncture, 41, 11–16. [https://doi.org/10.19917/j.cnki.1005-0779.025085](https://doi.org/10.19917/j.cnki.1005-0779.025085)

[5] Song YR, Yang S, Qi M, Pan WH. (2025). Effects of scalp acupuncture combined with Liuzijue exercise on cognition, event-related potential P300, and serum NPAS4 in patients with post-stroke cognitive impairment. Laboratory Medicine and Clinic, 22, 742–746.

[6] Li LS, Qi JJ, Sun YJ, Huang GR. (2023). Effects of cognitive rehabilitation training combined with acupoint electrostimulation on cognitive impairment after cerebral infarction. Henan Medical Research, 32, 3508–3512.

[7] Huang SE, Xue XH, Lin ZC, Wu ZK, Liao SQ, Zhuo LP. (2014). Effects of Governor Vessel–targeted acupuncture on event-related potentials in post-stroke cognitive impairment. Asia-Pacific Traditional Medicine, 10, 44–46.

[8] Liu J, Feng XD. (2013). Electroacupuncture at Baihui (GV20) and Shenting (GV24) combined with rehabilitation training for post-stroke cognitive impairment: A clinical study. Journal of Traditional Chinese Medicine, 28, 608–610.
[https://doi.org/10.16368/j.issn.1674-8999.2013.04.020](https://doi.org/10.16368/j.issn.1674-8999.2013.04.020)

[9] Liu RL. (2017). Clinical study on electroacupuncture at Shenting (GV24) and Baihui (GV20) for mild cognitive impairment after stroke. Clinical Research and Practice, 2, 101–102.[https://doi.org/10.19347/j.cnki.2096-1413.201729049](https://doi.org/10.19347/j.cnki.2096-1413.201729049)

[10] Yang M, Zhu XH, Zhu RH, Dai JL, Sheng G. (2022). Effects of acupuncture combined with cognitive training on vascular cognitive impairment after stroke and on serum CRP, homocysteine, and neuron-specific enolase. Modern Journal of Integrated Traditional Chinese and Western Medicine, 31, 1981–1984.

[11] Zhou JY, Zuo JJ, Chen B, Lü JL. (2019). Efficacy of electroacupuncture at Baihui (GV20) and Shenting (GV24) for mild cognitive impairment after stroke. World Chinese Medicine, 14, 486–489.

[12] Fang Z. (2019). Effects of warming acupuncture on mild cognitive impairment after stroke and vascular endothelial function. Modern Journal of Integrated Traditional Chinese and Western Medicine, 28, 424–426, 433.

[13] Yang M, Zhang B. (2017). Clinical observation of acupuncture combined with cognitive training for 47 cases of post-stroke cognitive impairment. Journal of Yunnan Traditional Chinese Medicine and Materia Medica, 38, 64–66.
[https://doi.org/10.16254/j.cnki.53-1120/r.2017.02.028](https://doi.org/10.16254/j.cnki.53-1120/r.2017.02.028)

[14] Wang YX, Liu JZ, Li SJ, Liang H, Gao QR. (2016). Effects of acupuncture on serum IL-6 and TNF-α in post-stroke mild cognitive impairment with phlegm–blood stasis pattern. Chinese Journal of Rehabilitation, 26, 12–16.

[15] Bao Y, Zou W, Sun XW. (2012). Clinical observation of combined acupuncture and medication for mild cognitive impairment after cerebral infarction. Shanghai Journal of Acupuncture and Moxibustion, 31, 470–472.

[16] Sun YZ, Wu WP. (2011). Cluster needling of scalp points for cognitive impairment after ischemic stroke: A study of 36 cases. Journal of Clinical Acupuncture, 27, 11–13.

[17] Xiang R, Han LL, Liu WG, Han J, Gao W. (2019). Clinical observation of scalp acupuncture combined with neck acupuncture and body acupuncture for post-stroke cognitive impairment. Modern Journal of Integrated Traditional Chinese and Western Medicine, 28, 3391–3394.

[18] Wei BX, Liu GC, Zeng JY. (2023). Scalp motor-acupuncture combined with cognitive training for post-stroke cognitive impairment: A clinical observation. Journal of Guangzhou University of Chinese Medicine, 40, 368–374. https://doi.org/10.13359/j.cnki.gzxbtcm.2023.02.016

[19] Yan HD, Yang N, Zhao MH, Zheng LQ. (2016). Thunder-fire moxibustion at Dazhui (GV14) and Shenshu (BL23) combined with cognitive training for mild cognitive impairment after ischemic stroke. Shanghai Journal of Acupuncture and Moxibustion, 35, 1410–1413. https://doi.org/10.13460/j.issn.1005-0957.2016.12.1410

[20] Zhang XY, An JM. (2018). Clinical efficacy of Fang’s scalp acupuncture combined with idebenone for post-stroke cognitive impairment. Journal of Practical Cardio-Cerebral-Pulmonary and Vascular Diseases, 26, 146–148.

[21] Li LC, Jin XY, Zeng XQ. (2019). Scalp acupuncture combined with nimodipine for mild cognitive impairment after stroke: A clinical study. Journal of Traditional Chinese Medicine, 25, 49–52.https://doi.org/10.13862/j.cnki.cn43-1446/r.2019.22.015

[22] Cao H, Huang ZD. (2019). Effects of Jing-well point bloodletting on cognitive recovery and prognosis in post-stroke cognitive impairment. Shanghai Journal of Acupuncture and Moxibustion, 38, 164–168. https://doi.org/10.13460/j.issn.1005-0957.2019.02.0164

[23] Wang Q, Dong JC, Sun LY. (2019). Effects of acupuncture combined with atorvastatin on hemorheology and cognitive status in elderly patients with ischemic stroke and mild cognitive impairment. Chinese Journal of Gerontology, 39, 5180–5183.

[24] Gao Y, Yu B, Wang YS, Wang W, Xu HW. (2019). Effects of auricular acupressure combined with cognitive training on post-stroke cognitive impairment. Chinese Folk Therapy, 27, 33–35.https://doi.org/10.19621/j.cnki.11-3555/r.2019.2117

[25] Deng R, Lei ZQ, Yang Q, Wang XQ, Han B. (2021). Effects of scalp acupuncture combined with Jing-well bloodletting on post-stroke cognitive impairment. Shanghai Journal of Acupuncture and Moxibustion, 40, 536–540. https://doi.org/10.13460/j.issn.1005-0957.2021.05.0536

[26] Niu L, Qin HW, Kong YF. (2021). Scalp cluster-needling with long-retained needles combined with cognitive training for cognitive impairment after cerebral infarction: A clinical study. Integrative Medicine Research, 13, 377–380, 388.

[27] Wang ZY, Zhang H, Wang XW, Zhang DW. (2019). Effects of acupuncture combined with cognitive rehabilitation training on cognitive function and cytokines in post-stroke cognitive impairment. Shanghai Journal of Acupuncture and Moxibustion, 38, 1098–1102. https://doi.org/10.13460/j.issn.1005-0957.2019.10.1098

[28] Yuan HW, Liu YX, Zhang H, Liu Y, Li XL, Ni JX. (2022). “Tongdu Xingshen” acupuncture combined with cognitive training for mild cognitive impairment after stroke: A randomized controlled trial. Chinese Acupuncture & Moxibustion, 42, 839–843. https://doi.org/10.13703/j.0255-2930.20210811-0005

[29] Xiong J, Zhang Z, Ma Y, Li Z, Zhou F, Qiao N, Liu Q, Liao W. (2020). Effects of combined scalp acupuncture and cognitive training on cognitive and motor functions in stroke patients. NeuroRehabilitation, 46, 75–82. https://doi.org/10.3233/NRE-192942

[30] Wang HL, Feng XD, Chen Z. (2017). Clinical efficacy of electroacupuncture at Baihui (GV20) and Zusanli (ST36) combined with rehabilitation training for post-stroke cognitive impairment. Journal of Clinical Research in Traditional Chinese Medicine, 9, 67–70.

[31] Sun SB, Cao EM, Chen C, Hao PF, Sun XL, Yang YJ, Li YJ, Tian HW, Liu G. (2017). Electroacupuncture at Shenting (GV24) and Sishencong (EX-HN1) combined with syndrome differentiation acupoint selection for post-stroke cognitive impairment: A clinical observation. Journal of Traditional Chinese Medicine, 29, 1864–1866. https://doi.org/10.16448/j.cjtcm.2017.0619

[32] Wei RP, Zhang JB, Cong WD. (2019). Efficacy of acupuncture for cognitive function after cerebral infarction and analysis of NIHSS and ADL scores. Jilin Journal of Traditional Chinese Medicine, 39, 1373–1376. https://doi.org/10.13463/j.cnki.jlzyy.2019.10.029

[33] Wu C, Chang C. (2019). Clinical observation of Baicong acupuncture method for post-stroke cognitive impairment. Journal of Integrated Traditional Chinese and Western Cardiovascular Diseases, 17, 3493–3496.

[34] Saren, Li CJ, Cai HX. (2019). Effects of scalp cluster-needling on cognition and serum HIF-1α in vascular dementia after stroke. Shaanxi Journal of Traditional Chinese Medicine, 40, 1622–1626.

[35] Zhang SY, Pi M, Chen PD, Gou YH, Zhou R, Peng Y. (2015). Clinical study of Governor Vessel–regulating acupuncture for mild cognitive impairment after ischemic stroke. New Chinese Medicine, 47, 255–257. https://doi.org/10.13457/j.cnki.jncm.2015.07.114

[36] Li ZT, Ban LQ, Chen F. (2023). Peri-cranial acupoint–tailored acupuncture for mild cognitive impairment after stroke: A randomized controlled trial. Chinese Acupuncture & Moxibustion, 43, 1104–1108. https://doi.org/10.13703/j.0255-2930.20221231-0002

[37] Mu X, Guo L, Yang Y, Qin Y, Jing X, Mao M. (2024). A randomized controlled clinical trial of Xingnao Kaiqiao acupuncture combined with modern rehabilitation in treating cognitive dysfunction after acute ischemic stroke. Topics in Geriatric Rehabilitation, 40, 196–202. https://doi.org/10.1097/TGR.0000000000000444

[38] Zeng YH, Bao YH, Zhu M, Chen SX, Fang JQ. (2015). Acupuncture for mild cognitive impairment in subacute stroke: A randomized controlled study. Chinese Acupuncture & Moxibustion, 35, 979–982. https://doi.org/10.13703/j.0255-2930.2015.10.001

[39] Luo JC, Chen AW, Lang BX. (2019). Clinical study of Xiangba Zhen (Eight-Needle of the Neck) for mild cognitive impairment after stroke. Shanghai Journal of Acupuncture and Moxibustion, 38, 169–173. https://doi.org/10.13460/j.issn.1005-0957.2019.02.0169

[40] Zhang QF, Li LX. (2018). Bagua scalp acupuncture for mild cognitive impairment after ischemic stroke: A clinical study. Chinese Journal of Emergency in Traditional Chinese Medicine, 27, 1232–1234.

[41] Ding X, Zhang H. (2016). Effects of scalp acupuncture combined with motor relearning on post-stroke cognitive impairment. Hebei Journal of Traditional Chinese Medicine, 38, 586–588.

[42] Tong X, Liu DD, Kou JY, Wei Y, Yang TS, Qiao LD. (2012). Through-needling combined with cognitive training for mild cognitive impairment after ischemic stroke. Chinese Folk Therapy, 20, 28–29. https://doi.org/10.19621/j.cnki.11-3555/r.2012.03.024

[43] Chen SF, Zhu CQ, Wang Y, Jiang LS, Sheng HM. (2019). Scalp embedding needles combined with auricular acupressure seeds for post-stroke cognitive impairment: A study of 40 cases. Journal of Anhui University of Traditional Chinese Medicine, 38, 52–54.

[44] Tian R, Zhang L, Ma T, Zhou N, Bai M. (2021). Clinical effects of scalp acupuncture combined with hyperbaric oxygen on post-stroke cognitive impairment. Guangxi Medical Journal, 43, 1829–1831, 1857.

[45] Guo, Y., Sun, T., Qiu, F., Li, X., Cui, W., Liao, Z., Yao, J. (2025). Electroacupuncture combined with cognitive rehabilitation outperforms cognitive rehabilitation alone in treating post-stroke cognitive impairment: A randomized controlled trial. Frontiers in Neurology, 16, 1507475. https://doi.org/10.3389/fneur.2025.1507475

[46] Boyang, Z., Yang, Z., Liyuan, F., Dan, S., Lei, H., Dan, T., Ruoyu, W., Xue, S., Jing, S., Dongyan, W. (2024). Neural regulation mechanism of head electroacupuncture on brain network of patients with stroke-related sleep disorders. Journal of Traditional Chinese Medicine.

[47] Huang, L., Yin, X., Li, W., Cao, Y., Chen, Y., Lao, L., Zhang, Z., Mi, Y., Xu, S. (2021). Effects of acupuncture on vascular cognitive impairment without dementia: A randomized controlled trial. Journal of Alzheimer’s Disease, 81, 1391–1401. https://doi.org/10.3233/JAD-201353

[48] Zhan J, Pan RH, Guo YH, Zhan LC, He MF, Wang QC, Chen HX. (2016). Acupuncture at Baihui and Shenting combined with basic treatment and routine rehabilitation for post-stroke cognitive impairment: A randomized controlled study. Chinese Acupuncture & Moxibustion, 36, 803–806. https://doi.org/10.13703/j.0255-2930.2016.08.007

[49] Chen, L., Fang, J., Ma, R., Gu, X., Chen, L., Li, J., Xu, S. (2016). Additional effects of acupuncture on early comprehensive rehabilitation in patients with mild to moderate acute ischemic stroke: A multicenter randomized controlled trial. BMC Complementary and Alternative Medicine, 16, 226. https://doi.org/10.1186/s12906-016-1193-y

[50] Zhang SS, Shen J. (2022). Randomized controlled study on acupuncture combined with tDCS for post-stroke cognitive impairment. Laboratory Medicine and Clinic, 19, 884–889.

[51] Chen QS. (2020). Clinical observation of scalp acupuncture combined with repetitive TMS for cognitive impairment in stroke patients. Yunnan Journal of Traditional Chinese Medicine, 41, 60–62. https://doi.org/10.16254/j.cnki.53-1120/r.2020.04.019

[52] Li JH, Yang ZH, Yao JF, Lü JC, Zhou H. (2019). Acupuncture combined with computer-assisted cognitive training for post-stroke cognitive impairment. Ningxia Medical Journal, 41, 848–850. https://doi.org/10.13621/j.1001-5949.2019.09.0848

[53] Yao JJ, Lin SF, Yu JF. (2019). Acupuncture combined with cognitive rehabilitation training for cognitive impairment after ischemic stroke. Asia-Pacific Traditional Medicine, 15, 119–122.

[54] Liu XJ, Zhu L, Xu Y, Liu XY. (2023). Effects of repetitive TMS combined with scalp acupuncture on cognitive impairment after stroke. Journal of China Medical University, 52, 115–119.

[55] Chen HL, Guan F. (2023). Clinical effects of acupuncture combined with cognitive rehabilitation for mild cognitive impairment after stroke. Journal of Integrated Traditional Chinese and Western Cardiovascular Diseases, 21, 361–363.

[56] Li L, Xiao P, Chen Q, Tang L. (2019). Effects of acupuncture combined with donepezil on cognitive impairment after stroke in the elderly. Chinese Journal of Prevention and Control of Chronic Diseases, 27, 617–620. https://doi.org/10.16386/j.cjpccd.issn.1004-6194.2019.08.015

[57] Zhong, L., Luo, J., Ma, X., Yan, J., Tang, Q., X., Lan, S. (2025). fNIRS-based evaluation of scalp acupuncture combined with iTBS for cognitive impairment after stroke. NeuroRehabilitation, 56, 152–163. https://doi.org/10.1177/10538135241303348

[58] Zhang CX, Zhang SH, Wang YL, Zhang CP, Li QF, Pan WY, Liang WR. (2021). Interactive scalp acupuncture for post-stroke cognitive impairment: A randomized controlled study. Chinese Acupuncture & Moxibustion, 41, 252–256. https://doi.org/10.13703/j.0255-2930.20200212-k0003

[59] Wang, S., Yang, H., Zhang, J., Zhang, B., Liu, T., Gan, L., Zheng, J. (2016). Efficacy and safety assessment of acupuncture combined with nimodipine for mild cognitive impairment after cerebral infarction: A randomized controlled trial. BMC Complementary and Alternative Medicine, 16, 361. https://doi.org/10.1186/s12906-016-1337-0

[60] Jiang, C., Yang, S., Tao, J., Huang, J., Li, Y., Ye, H., Chen, S., Hong, W., Chen, L. (2016). Acupuncture combined with RehaCom cognitive training for cognitive improvement after stroke: A 2×2 factorial randomized controlled trial. Journal of the American Medical Directors Association, 17, 1114–1122. https://doi.org/10.1016/j.jamda.2016.07.021

[61] Wang HY, Wang PQ. (2022). Efficacy of eye acupuncture combined with tDCS for post-stroke cognitive impairment. Journal of Liaoning University of Traditional Chinese Medicine, 24, 59–62. https://doi.org/10.13194/j.issn.1673-842x.2022.04.014

[62] Yang L, Tan J, Chen LZ. (2019). Clinical observation of scalp acupuncture combined with hyperbaric oxygen for post-stroke cognitive impairment. Journal of Traditional Chinese Medicine Guide, 25, 36–38. https://doi.org/10.13862/j.cnki.cn43-1446/r.2019.24.012

[63] Liu L, Xia WG, Xu T. (2019). Clinical observation of scalp acupuncture combined with repetitive TMS for cognitive impairment after cerebral infarction. Chinese Rehabilitation, 34, 123–126.

[64] Huang F, Liu Y, Zhou FX, Yao GX, He QT. (2008). Effects of acupuncture on vascular cognitive impairment after cerebral infarction. Guangdong Medical Journal, 1918–1920. https://doi.org/10.13820/j.cnki.gdyx.2008.11.007

[65] Zhang Xiaoli, Luo Yang, Chen Yiliang, Feng Weiju, Lu Hui. (2012). Clinical study of acupuncture combined with herbal medicine for acute cerebral infarction with cognitive impairment. Jiangsu Traditional Chinese Medicine, 44, 57–59.

[66] Bai Jing, Li Baodong, Wang Qinghai. (2012). Cluster needling of scalp points combined with cognitive training for post-stroke cognitive impairment: A clinical observation. Shanghai Journal of Acupuncture and Moxibustion, 31, 711–713.

[67] X., Li, L., Liu, H., Shi, H., Xu, S., Wang, M., Xie, J. (2021). Effect of acupuncture combined with rehabilitation on cognitive and motor functions in post-stroke patients. The Anatomical Record, 304, 2531–2537. https://doi.org/10.1002/ar.24700

[68] Tian Yali, Su Yuting, Feng Xiaoyang. (2021). Clinical observation of acupuncture combined with cognitive rehabilitation training for post-stroke cognitive impairment. Clinical Medicine, 41, 116–117. https://doi.org/10.19528/j.issn.1003-3548.2021.01.049

[69] Han Chengyan, Wen Wanshun, Yao Baolong, Yang Ting, Ye Xiangming. (2018). Effects of cognition training during scalp needle retention on cognitive function after stroke. Modern Practical Medicine, 30, 1592–1594.

[70] Wang Yongxin, Zhang Hongshun, Wang Lichun, Miao Wei, Xu Zhiguang, Liu Mingqing. (2017). Effects of scalp cluster needling combined with cognitive rehabilitation on UA, ACA, and VEGF levels in patients with cognitive impairment after cerebral infarction. Hebei Journal of Traditional Chinese Medicine, 32, 36–38. https://doi.org/10.16370/j.cnki.13-1214/r.2017.05.012
